# The coelurosaur theropods of the Romualdo formation, early Cretaceous (Aptian) of Brazil: *Santanaraptor placidus* meets *Mirischia asymmetrica*

**DOI:** 10.1002/ar.70085

**Published:** 2025-11-18

**Authors:** Rafael Delcourt, Orlando Nelson Grillo, Christophe Hendrickx, Maximilian Kellermann, Max Cardoso Langer

**Affiliations:** ^1^ Laboratório de Paleontologia de Ribeirão Preto FFCLRP, Universidade de São Paulo Ribeirão Preto Brazil; ^2^ Laboratório de Processamento de Imagem Digital, Departamento de Geologia e Paleontologia, Museu Nacional Universidade Federal do Rio de Janeiro Rio de Janeiro Brazil; ^3^ Dinosauria Lab, Fundación Miguel Lillo Tucumán Argentina; ^4^ Unidad Ejecutora Lillo, CONICET‐Fundación Miguel Lillo Tucumán Argentina; ^5^ Staatliche Naturwissenschaftliche Sammlungen Bayerns–Bayerische Staatssammlung für Paläontologie und Geologie Munich Germany; ^6^ Department für Geo‐ und Umweltwissenschaften Sektion Paläontologie und Geobiologie, Ludwig‐Maximilians‐Universität Munich Germany

**Keywords:** Araripe, Coelurosauria, Dinosauria, Gondwana, Theropoda

## Abstract

The upper carbonate concretion levels of the Romualdo Formation (Aptian, Brazil) have yielded several theropod dinosaur remains, including spinosaurids and the coelurosaurs *Santanaraptor placidus* and *Mirischia asymmetrica*, the phylogenetic affinities of which are controversial. Here, we present a comprehensive anatomical reassessment of the holotypes of both species (MN 4802‐V and SMNK 2349 PAL, respectively), integrating newly observed osteological features and a detailed comparison of the pelvic and hind limb elements. Our preferred phylogenetic hypothesis places *S. placidus* and *M. asymmetrica* in the earliest‐branching maniraptoromorph clade, along with *Juratyrant langhami* and *Tanycolagreus topwilsoni* from the Late Jurassic of Laurasia, suggesting an early diversification of coelurosaurs in that area, followed by Early Cretaceous dispersal events towards Gondwana. The comparative analysis of the two Romualdo taxa refutes their synonymy, given consistent differences in ischial (position and shape of the obturator plate and foramen) and tibial (condylar configuration) morphology. The observed morphological variation in the ischial obturator plate across early coelurosaurs further highlights a significant degree of homoplasy in this structure during the early radiation of the group. This revision underscores the need for additional research to further resolve the early evolutionary history of coelurosaur theropods.

## 1 INTRODUCTION

**FIGURE 1:**
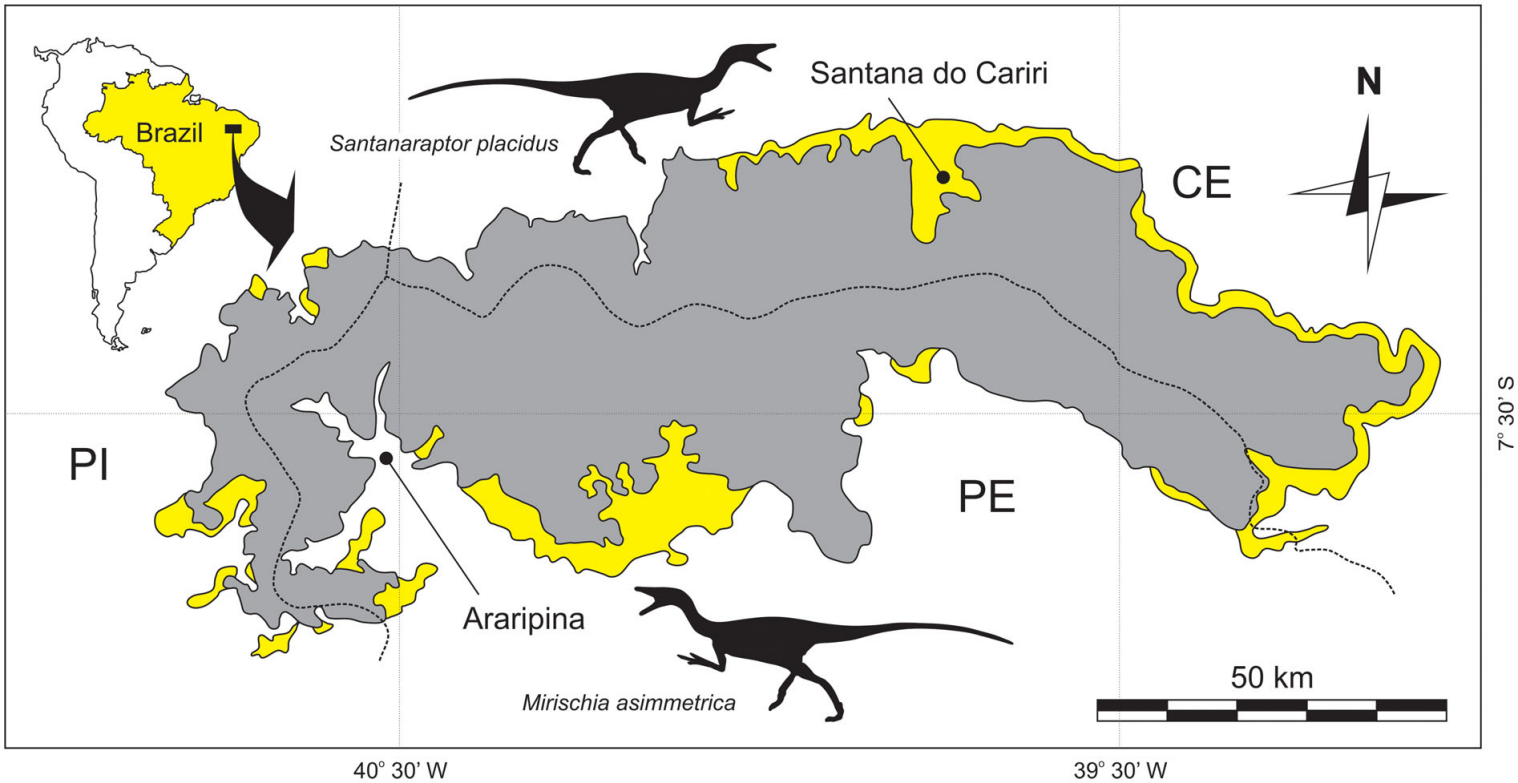
Map depicting the Araripe Plateau (gray) and its borders where the Crato, Ipubi, and Romualdo formations are exposed (yellow); dotted lines indicate borders between Ceará (CE), Pernambuco (PE), and Piauí (PI) states; from Assine (2007). Silhouettes representing *Santanaraptor placidus* and *Mirischia asymmetrica* are positioned close to the towns that may have yielded the type‐specimens.

The Romualdo Formation is a world‐famous Early Cretaceous (Aptian) fossil *Konservat Lagerstätte* that crops out along the margins of the Araripe Plateau, in northwestern Brazil (Figure 1; Custódio et al., 2017; Arai & Assine, 2020; Melo et al., 2020). Its macrofossil record includes one of the most diverse and well‐preserved ichthyofaunas of the Mesozoic (Maisey, 1991), as well as plants (de Lima et al., 2012), “invertebrates” (de Araújo Gomes et al., 2023), turtles (Scheyer et al., 2023), crocodilians (Price, 1959), pterosaurs (Kellner, 2013), and theropod dinosaurs. Spinosaurs are arguably the best‐known Romualdo theropods, with many referred specimens (Aureliano et al., 2018; Sales & Schultz, 2017), including the well‐preserved skull of *Irritator challengeri* (Schade et al., 2023; Sues et al., 2002) and the snout of *Angaturama limai* (Kellner & Campos, 1996). Among coelurosaurs, *Santanaraptor placidus* (Kellner, 1999) and *Mirischia asymmetrica* (Naish et al., 2004) come from the same deposits that yielded the spinosaurs, that is, the better‐known upper carbonate concretion levels of the Romualdo Formation, whereas *Aratasaurus museunacionali* was recovered from the lower levels of that unit (Sayão et al., 2020). Finally, depending on the phylogenetic position of *Megaraptora* (Novas et al., 2013), a possible member of that group (Aranciaga Rolando et al., 2018) completes the coelurosaur record of the Romualdo Formation.

*Santanaraptor placidus* was initially referred to *Maniraptoriformes* by Kellner (1996, 1999) who later classified it as a tyrannoraptoran (Kellner, 2001). Its tyrannosauroid affinity was hinted at by Holtz Jr (2004) and later supported by most phylogenetic analyses that included this taxon (Delcourt & Grillo, 2018; Novas et al., 2013; Porfiri et al., 2014; Sayão et al., 2020), although this remains debated (e.g., Brownstein, 2021; Cau, 2024). As for *M. asymmetrica*, it was first assigned to Compsognathidae (Martill et al., 2000; Naish et al., 2004; Rauhut, 2003), an affinity not firmly contested since. Some later studies confirmed this hypothesis (Brusatte et al., 2014; Novas et al., 2012; Pei et al., 2020; Peyer, 2006; Pol & Goloboff, 2020), although some uncertainty remains (Cau, 2024; Göhlich & Chiappe, 2006; Naish & Cau, 2022). More recently, Qiu et al. (2025) proposed that *M. asymmetrica* may correspond to a member of the early branching coelurosaur clade Sinosauropterygidae, otherwise restricted to the early Aptian of China. Alternatively, the clade might also include the European *Compsognathus longipes* and *Juravenator starki*, in which case it would correspond to Compsognathidae.

Here, we revise and compare the anatomy of *S. placidus* and *M. asymmetrica*, with the goal of investigating their phylogenetic affinities and possible synonymy. The senior author is, to our knowledge, the only person to have granted first‐hand access to the holotypes of both taxa, respectively MN 4802‐V and SMNK 2349 PAL. Given that the former was badly damaged (the extent of the damage still needs to be properly documented) by the Museu Nacional fire of 2018 (Mendes et al., 2022), this contribution provides a unique and needed perspective on this issue.

## 2 MATERIALS AND METHODS

### 2.1 Materials

MN 4802‐V, the holotype of *S. placidus* includes three mid‐distal tail vertebrae, both ischia, femora, and tibiae (partial), left fibula (fragment), right astragalus, calcaneum, and distal tarsals III and IV, left distal tarsal IV, and both pes (partial). SMNK 2349 PAL, the holotype of *M. asymmetrica*, preserves the last two trunk and the first three sacral vertebrae as well as a few gastral elements, both ilia (partial), both pubes and ischia, right femur, left femur (partial), and right tibia (partial).

### 2.2 Terminology

Here, we follow the Nomina Anatomica Avium (Clark Jr., 1993) and the International Committee on Veterinary Gross Anatomical Nomenclature (Nomina Anatomica Veterinaria, 2005) to describe the body parts of *S. placidus* and *M. asymmetrica*. The vertebral laminae and fossae were described following the terminology of Wilson (1999, 2012) and Wilson et al. (2011), whereas that of Hendrickx et al. (2022) was used to describe the soft tissues. Under the renewed aftermath of the PhyloCode (Cantino & De Queiroz, 2020) and Phylonyms (De Queiroz et al., 2020) and for future systematized use, Table 1 provides definitions for the following clades: *Coelurosauria*, *Eutyrannosauria*, *Megaraptora*, *Maniraptoriformes*, *Maniraptoromorpha*, *Neocoelurosauria*, *Ornithomimosauria*, *Tyrannoraptora*, and *Tyrannosauroidea*. Following PhyloCode article 6, recommendation 6.1A (Cantino & De Queiroz, 2020), all clades established under this code are italicized.

**TABLE 1:** Phylogenetic definitions of clade names used in this study.

Coelurosauria von Huene, 1914 [this work], converted clade name Registration Number: 551	Phylogenetic definition: The largest clade containing *Vultur gryphus* Linnaeus 1758, but not *Allosaurus fragilis* March 1877. This is a maximum clade definition. Reference phylogeny: Phylogenetic hypothesis depicted in Figure 2 of Brusatte et al. (2014). Composition: based on the reference phylogeny, *Coelurosauria* includes Tyrannosauroidea, Compsognathidae, Ornithomimosauria, Alvarezsauroidea, Therizinosauroidea, Oviraptorosauria, Troodontidae, Dromaeosauridae, and Avialae.
Tyrannoraptora Sereno, 1999 [this work], converted clade name Registration Number: 1169	Phylogenetic definition: The least inclusive clade containing *Vultur gryphus* Linnaeus 1758 and *Tyrannosaurus rex* Osborn 1905. This is a minimum clade definition. Reference phylogeny: Phylogenetic hypothesis depicted in Figure 2 of Brusatte et al. (2014). Composition: based on the reference phylogeny, *Tyrannoraptora* includes Tyrannosauroidea, Compsognathidae, Ornithomimosauria, Alvarezsauroidea, Therizinosauroidea, Oviraptorosauria, Troodontidae, Dromaeosauridae, and Avialae.
Maniraptoromorpha Cau, 2018 [this work], converted clade name Registration Number: 1170	Phylogenetic definition: The largest clade containing *Vultur gryphus* Carolus Linnaeus 1758, but not *Tyrannosaurus rex* Osborn 1905. This is a maximum clade definition. Reference phylogeny: Phylogenetic hypothesis depicted in Figure 2 of Brusatte et al. (2014). Composition: based on the reference phylogeny, *Maniraptoromorpha* includes Compsognathidae, Ornithomimosauria, Alvarezsauroidea, Therizinosauroidea, Oviraptorosauria, Troodontidae, Dromaeosauridae, and Avialae.
Neocoelurosauria Hendrickx et al., 2019 [this work], converted clade name Registration Number: 1171	Phylogenetic definition: The least inclusive clade containing *Vultur gryphus* Linnaeus 1758 and *Compsognathus longipes* Wagner 1859. This is a minimum clade definition. Reference phylogeny: Phylogenetic hypothesis depicted in Figure 2 of Brusatte et al. (2014). Composition: based on the reference phylogeny, Neocoelurosauria includes Compsognathidae, Ornithomimosauria, Alvarezsauroidea, Therizinosauroidea, Oviraptorosauria, Troodontidae, Dromaeosauridae, and Avialae.
Maniraptoriformes Holtz 1996 [this work], converted clade name Registration Number: 1172	Phylogenetic definition: The least inclusive clade containing *Vultur gryphus* Linnaeus 1758 and *Ornithomimus velox* Marsh 1890. This is a minimum clade definition. Reference phylogeny: Phylogenetic hypothesis depicted in Figure 2 of Brusatte et al. (2014). Composition: based on the reference phylogeny, Maniraptoriformes includes Ornithomimosauria, Alvarezsauroidea, Therizinosauroidea, Oviraptorosauria, Troodontidae, Dromaeosauridae, and Avialae.
Ornithomimosauria Barsbold, 1976 [this work], converted clade name Registration Number: 1173	Phylogenetic definition: The largest clade containing *Ornithomimus velox* Marsh 1890, but not *Vultur gryphus* Linnaeus 1758, *Tyrannosaurus rex* Osborn 1905, *Nothronychus mckinleyi* Kirkland & Wolfe 2001 or *Alvarezsaurus calvoi* Bonaparte 1991. This is a maximum clade definition. Reference phylogeny: Phylogenetic hypothesis depicted in Figure 23 of Serrano‐Brañas et al. (2020). Composition: based on the reference phylogeny, *Ornithomimosauria* includes *Nqwebasaurus thwazi*, *Beishanlong grandis*, *Pelecanimimus polyodont*, *Shenzhousaurus orientalis*, Deinocheiridae, and Ornithomimidae.
Megaraptora Benson, Carrano & Brusatte 2010b [this work], converted clade name Registration Number: 1174	Phylogenetic definition: The largest clade containing *Megaraptor namunhuaiquii* Novas 1998, but not *Tyrannosaurus rex* Osborn 1905 or *Allosaurus fragilis* March 1877. This is a maximum clade definition. Reference phylogeny: Phylogenetic hypothesis depicted in Figure 12 of Porfiri et al. (2014). Composition: based on the reference phylogeny, *Megaraptora* includes *Fukuiraptor* and Megaraptoridae.
Tyrannosauroidea Osborn, 1906 [this work], converted clade name Registration Number: 1175	Phylogenetic definition: The largest clade containing *Tyrannosaurus rex* Osborn 1905, but not *Vultur gryphus* Linnaeus 1758 or *Ornithomimus velox* Marsh 1890. This is a maximum clade definition. Reference phylogeny: Phylogenetic hypothesis depicted in Figure S1 of Brusatte et al. (2014). Composition: based on the reference phylogeny, Tyrannosauroidea includes Proceratosauridae and Pantyrannosauria.
Eutyrannosauria Delcourt & Grillo, 2018 [this work], converted clade name Registration Number: 1176	Phylogenetic definition: The largest clade containing *Tyrannosaurus rex* Osborn 1905, but not *Megaraptor namunhuaiquii* Novas 1998. This is a maximum clade definition. Reference phylogeny: Phylogenetic hypothesis depicted in Figure 3 of Delcourt and Grillo (2018). Composition: Based on the refence phylogeny, Eutyrannosauria includes *Dryptosaurus*, *Appalachiosaurus*, *Bistahieversor*, and Tyrannosauridae.

### 2.3 Phylogenetic analysis

The phylogenetic affinities of *S. placidus* and *M. asymmetrica* were explored based on the comprehensive and continuously updated (Isasmendi et al., 2024; Kellermann et al., 2025) taxon‐character matrix of the “Mesozoic Tetrapod Work Group” (MTWG) from the Bayerische Staatssammlung für Paläontologie und Geologie, accessed on August 26, 2025. The MTWG matrix is a modified version of that first published by Rauhut and Pol (2021), which was in turn modified from the data matrix of Wang et al. (2017) and previous works. As a part of the continuous revisions by the members of the MTWG, the matrix has undergone major restructuring since its last published version (Kellermann et al., 2025). To facilitate both a better comparison between characters, as well as making it easier to score new taxa into the dataset, the characters were shuffled to fit the anatomical regions. Multiple new taxa (e.g., *Buriolestes schultzi, Kiyacursor longipes*, *Gualicho shinyae*, *Deltadromeus agilis*) and multiple new characters were added; others were modified, reworded or removed and many of the scorings have since been reviewed. For this manuscript, *S. placidus* and *M. asymmetrica* were added based on detailed examinations of MN 4802‐V and SMNK 2349 PAL. Two new characters (see Data S1) were added specifically for this manuscript to account for the variations observed between *S. placidus* and *M. asymmetrica* in the morphology of the tibia. These new characters are: 883—Tibia, proximal articulation, medial condyle, caudal margin in proximal view: (0) broadly rounded or subrectangular; (1) arcuate and posteriorly angular; 885—Tibia, proximal articulation, lateral condyle, lateromedial width relative to medial condyle [inapplicable for taxa with poorly defined posterior condyles]: (0) significantly smaller; (1) subequal; (2) significantly larger. To improve overall analysis performance, several phylogenetically far removed and/or very fragmentary taxa (e.g., *Veterupristisaurus milneri*, *Datanglong guangxiensis*, *Poekiliopleuron bucklandii*) were removed prior to the analysis. The final matrix therefore encompasses 188 OTUs and 967 morphological characters, many of which were treated as ordered (see Data S1).

The analysis was performed via a “New Technology Search” in TNT (Goloboff & Morales, 2023), combining sectorial searches and tree‐fusing as searching algorithms, in their default configurations (Goloboff, 1999), with 10 starting trees from a “driven search”, finding a minimum length 30 times, and collapsing the trees after the search. This initial step was followed up with a “Traditional search”, using the tree bisection and reconnection (TBR) swapping algorithm with trees from RAM generated by the previous “New Technology Search”. All of these steps were done twice, once under equal weights, as well as once with an implied weight of *k* = 16. This value was chosen in order to best reduce the effects of homoplasy for our sample size (Ezcurra 2024). The resulting most parsimonious trees were then summarized using a strict consensus (Wilkinson 1994).

### 2.4 Institutional abbreviations

AM, Albany Museum, Grahamstown, South Africa; AMNH, American Museum of Natural History, New York, USA; BSPG, Bayerische Staatssammlung für Paläontologie und historische Geologie, Munich, Germany; BYU, Brigham Young University, Provo, USA; FPDM, Fukui Prefectural Museum, Fukui, Japan; HMN, Humboldt Museum für Naturkunde, Berlin, Germany; IVPP, Institute of Vertebrate Paleontology and Paleoanthropology, Peking, China; MN, Museu Nacional/Universidade Federal do Rio de Janeiro, Rio de Janeiro, Brazil; MPCA, Museo Provincial Carlos Ameghino, Cipolletti, Argentina; MPD, Mongolian Paleontology Dinosaur, Paleontological Center of Mongolia, Ulaan Baatar, Mongolia; MPEF‐PV, Museo Paleontológico Egidio Feruglio, Trelew, Argentina; NCSM, North Carolina Museum of Natural Science, Raleigh, North Carolina, USA; SGM, Service Géologique du Maroc, Rabat, Morocco; SMNK, Staatliches Museum für Naturkunde Karlsruhe, Karlsruhe, Germany; UA, Université d'Antananarivo, Antananarivo, Madagascar; UC, University of Chicago, Chicago, USA; UMNH, Utah Museum of Natural History, Salt Lake City, USA.

## 3 RESULTS

### 3.1 Santanaraptor placidus and Mirischia asymmetrica in the spotlight

#### 3.1.1 Axial skeleton

**FIGURE 2:**
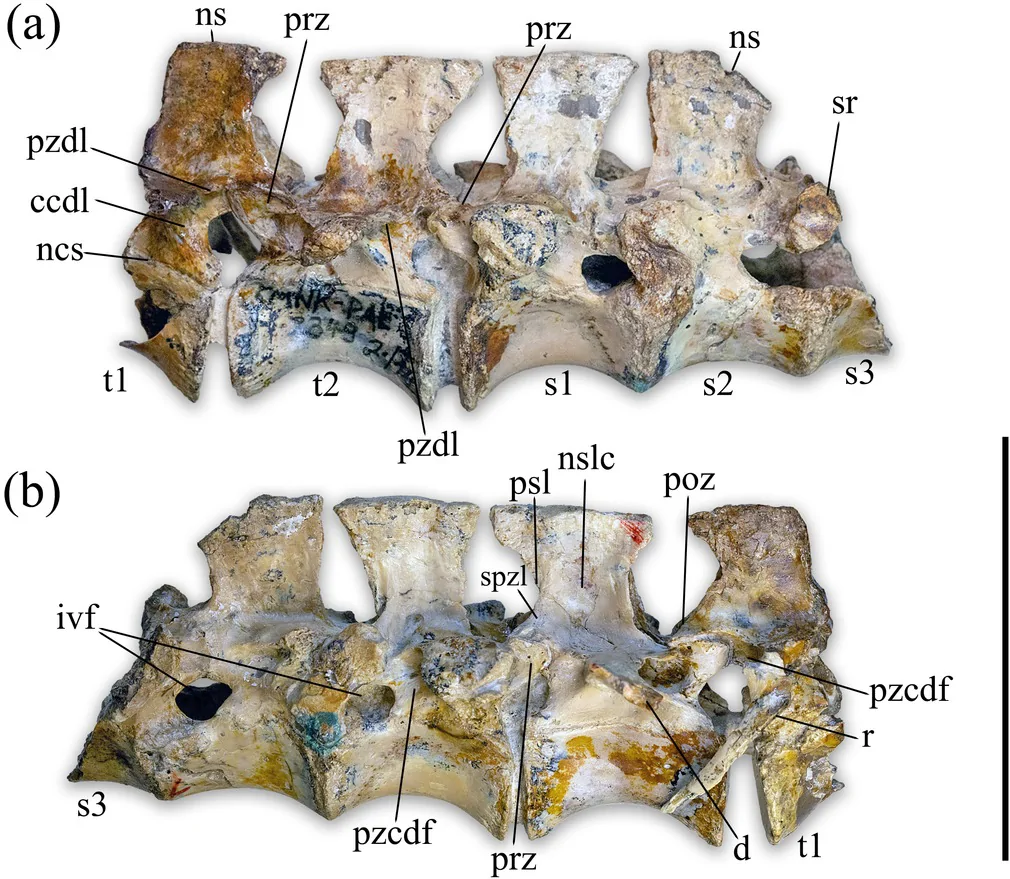
*Mirischia asymmetrica* (SMNK 2349). Vertebral series in (a) left lateral and (b) right lateral views. ccdl, caudal centrodiapophyseal lamina; d, diapophysis; ivf, intervertebral foramen; ncs, neurocentral suture; ns, neural spine; nslc, neural spine longitudinal concavity; poz, postzygapophysis; psl, postspinal lamina; prz, prezygapophysis; pzcdf, postzygapophyseal centrodiapophyseal fossa; pzdl postzygodiapophyseal lamina; r, rib; s, sacral vertebra; spzl, spinopostzygapophyseal lamina; sr, sacral rib; t, trunk vertebra. Scale bar equals 5 cm.

**FIGURE 3:**
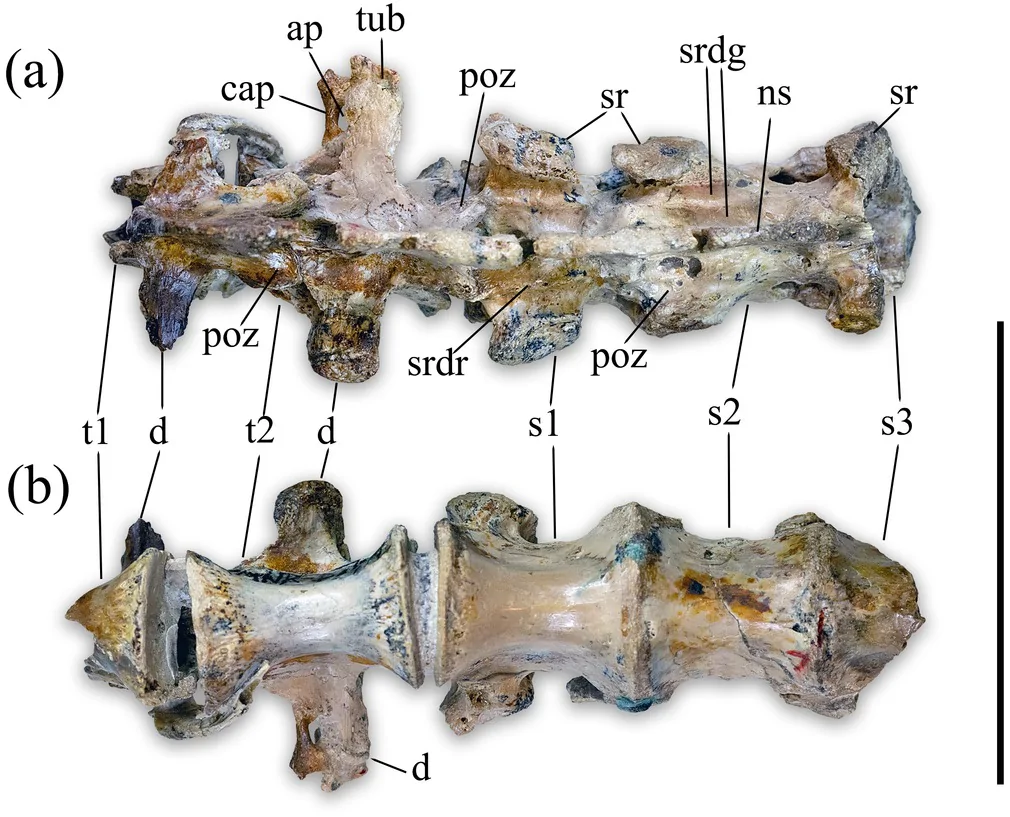
*Mirischia asymmetrica* (SMNK 2349). Vertebral series in (a) dorsal and (b) ventral views. ap, aperture; cap, capitulum; d, diapophysis; ns, neural spine; poz, postzygapophysis; s, sacral vertebra; sr, sacral rib; srdg, sacral rib dorsal groove; srdr, srdg, sacral rib dorsal ridge; t, trunk vertebra; tub, tuberculum. Scale bar equals 5 cm.

**FIGURE 4:**
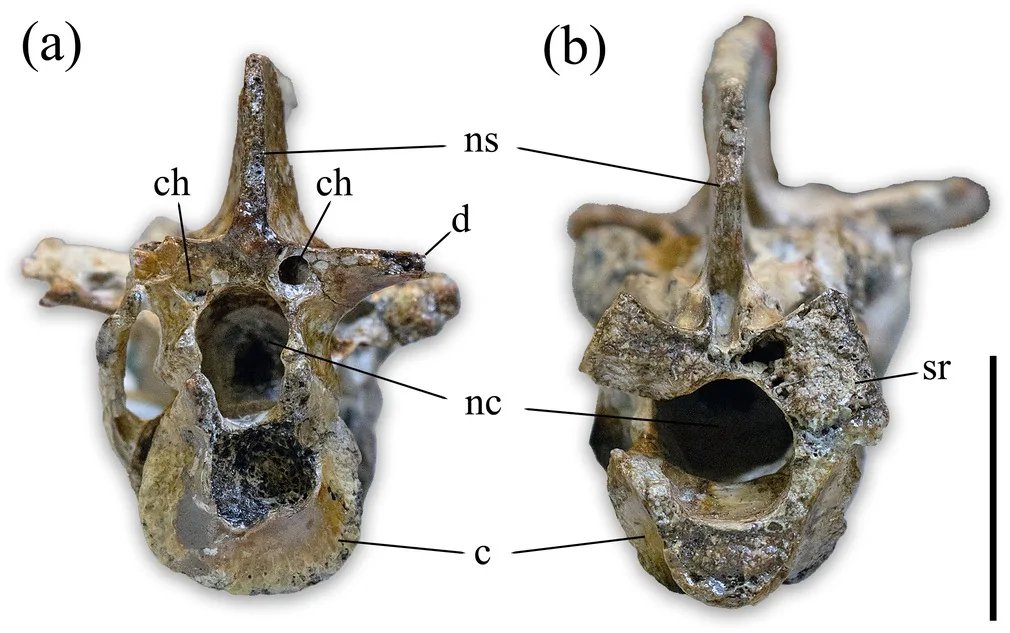
*Mirischia asymmetrica* (SMNK 2349). Trunk vertebra in cranial view (a) and sacral vertebra 3 in caudal view (b). c, vertebral centrum; ch, channel; d, diapophysis; nc, neural canal; ns, neural spine; sr, sacral rib. Scale bar equals 2 cm.

**FIGURE 5:**
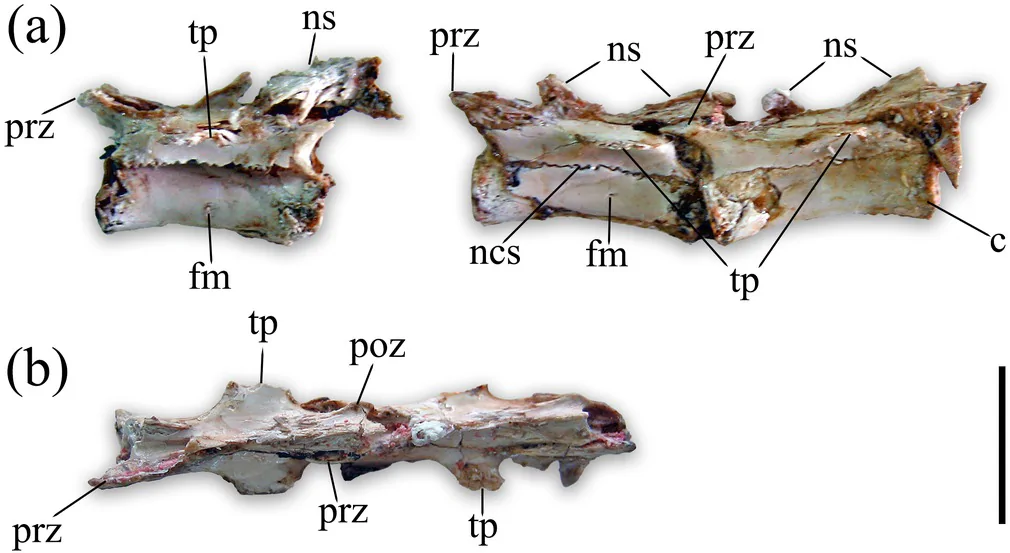
*Santanaraptor placidus* (MN 4802‐V). Caudal vertebrae in (a) left lateral; (b) dorsal views. c, vertebral centrum; fm, foramen; ns, neural spine; ncs, neurocentral suture; poz, postzygapophysis; prz, prezygapophysis; tp, transverse process. Scale bar equals 2 cm.

**FIGURE 6:**
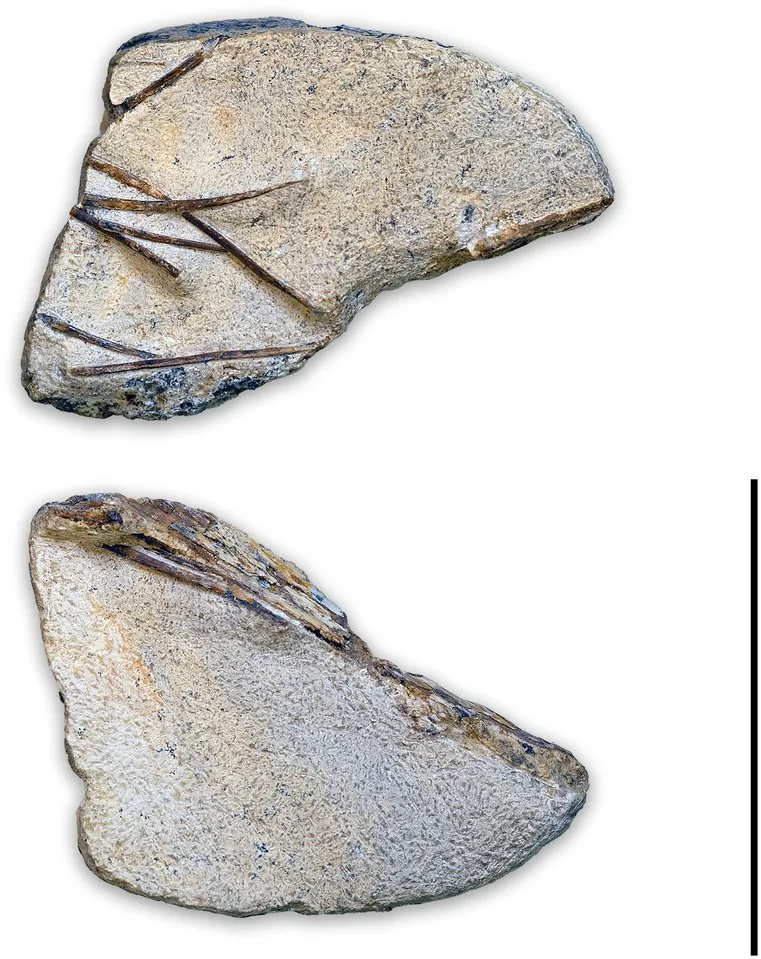
*Mirischia asymmetrica* (SMNK 2349 PAL). Gastral elements still on the rock matrix. Scale bar equals 5 cm.

*Mirischia asymmetrica* preserves the last two trunk and the first three sacral vertebrae, but the first and last of this series are incomplete (Figures 2, 3, 4 and S1). Based on circumstantial evidence, Martill et al. (2000) and Naish et al. (2004) considered the last trunk vertebra to be incorporated into the sacrum (= truncosacral). Yet, in the original articulation of the specimen, its transverse processes are clearly positioned at the level of the cranial edge of the iliac preacetabular ala, with no space for a proper articulation. Moreover, its right transverse process is articulated with a typically dichocephalous trunk rib, so that we consider it to represent the last trunk element, that is, a free element, not incorporated into the sacrum. *Santanaraptor placidus*, on the other hand, preserves four mid‐distal tail vertebrae (Figure 5). Hence, there is no overlap of axial elements between the two specimens. Also, elongated and tapering gastral elements are preserved close to the pubis of *M. asymmetrica*, some of which are curved (Figure 6).

The trunk vertebrae of *M. asymmetrica* have clear neurocentral sutures (“ncs” in Figures 2 and S1). The centrum of the last one (“t2” in Figures 2, 3, and S1) is spool‐shaped, amphicoelous, much longer than tall, with ventral longitudinal striations near the articular surfaces, matching what is preserved (caudal end) of the previous centrum. The centrum of the penultimate trunk vertebra (“t1” in Figures 2, 3, 4, and S1) has a concave caudal articular facet, with a circular caudal outline. Its neural arch is tall, about twice the centrum high. The cranial margin of the neural spine is missing, so that its shape is elusive. Its caudal margin is concave in lateral view, with a thick process expanding caudally from its dorsal margin. Only about half of the neural arch is preserved, missing the spinoprezygapophyseal laminae and prezygapophyses. The breakage exposes the channels leading to both laminopeduncular foramina (“ch” in Figure 4a), the right of which is positioned slightly more ventrally, closer to the neural canal, which is circular at the breakage and nearly half the diameter of the centrum. The left one is deeper as preserved, but it is unclear if this may be due to different degrees of preparation. The diapophyses are laterally directed and horizontally held (forming a 90° angle with the neural spine), with a proximal rib portion (“r” in Figures 2b and S1), with close capitulum and tuberculum, attached to the right element. Although incomplete distally, the diapophyses seem to narrow craniocaudally towards their distal ends. Each diapophysis is connected to the postzygapophysis and the caudoventral corner of the neural arch respectively by the postzygodiapophyseal (“pzdl” in Figure 2b,s) and caudal centrodiapophyseal (“ccdl” in Figures 2a and S1) laminae. The former of which is more marked, roofing a deep postzygapophyseal centrodiapophyseal fossa (“pzcdf” in Figures 2 and S1). Given the lack of the cranial part of the neural arch, other diapophyseal laminae are not preserved in that vertebra. Its postzygapophyses are caudolaterally projected, with the articular facets lateroventrally oriented; a small, rounded tubercle is seen on the dorsal surface of the right element. The centropostzygapophyseal (“cpzl” in Figures 2 and S1) and spinopostzygapophyseal (“spzl” in Figures 2 and S1) laminae form strongly concave caudal margins in lateral view, the pair of the latter forming the lateral margins of a deep spinopostzygapophyseal fossa, triangular in caudal view.

The last trunk vertebra of *M. asymmetrica* is like the preceding element in several aspects. Its centrum is longer than deep, spool‐shaped, with concave lateral and ventral margins. Ventral striations are seen near the articular surfaces and the right surface is pierced by a small foramen, set at the mid‐height of its cranial half. The neural spine expands dorsally, following a fan‐shaped lateral outline, with concave cranial and caudal margins. The spinoprezygapophseal and spinopostzygapophseal fossae are both deep, ventrally broader, and triangular‐shaped in cranial/caudal views. The lateral surfaces of the neural spine bear a dorsoventrally elongated subtle concavity at their central part (“nslc” in Figures 2 and S1). This is caudally bordered by a vertical ridge that continues along the dorsal surface of the postzygapophysis in the form of the spinopostzygapophyseal lamina, the pair of which delimit the spinopostzygapophyseal fossa. Medial to those laminae, the postspinal lamina (“psl” in Figures 2 and S1) divides the dorsal part of the spinopostzygapophyseal fossa, extending dorsally to form the caudally projected caudodorsal expansion of the neural spine, the lateral surface of which is also depressed relative to the dorsal portion of the spinopostzygapophyseal lamina. The dorsal margin of the neural spine is lateromedially broader at its caudal portion. The prezygapophyses are triangular in lateral view, with rounded apices that do not extend beyond the cranial edge of the centrum. The postzygapophyses are dorsoventrally shallower than those of the previous vertebra and project caudolaterally forming a higher (almost 45°) angle to the sagittal plane. Although mostly covered by the next prezygapophysis, their articular facets seem elongated rather than circular, and more vertically oriented than those of the previous trunk vertebra.

The diapophyses of the last trunk vertebra expand strictly laterally and horizontally, but are slightly twisted at their distal ends, becoming cranioventrally to caudodorsally oriented in lateral outline (Figures 2 and S1). The distal margin of the left element is slightly deeper dorsoventrally, whereas that of the right side is articulated with its respective rib, which led Martill et al. (2000) to erroneously suggest that the diapophysis was longer on that side. That apophysis is surrounded by four well‐developed laminae. The pre‐ and postzygodiapophyseal laminae are subhorizontal, respectively leading to the zygapophyses, whereas the caudal centrodiapophyseal lamina extends towards the caudoventral corner of the neural arch. Opposite to the latter, a ridge connects the diapophysis to the cranioventral corner of the neural arch, bearing an articular facet as it approaches the diapophysis. This is interpreted here as the parapophysis, so that most of that lamina corresponds to the centroparapophyseal lamina (“cpl” in Figures 2 and S1). Those laminae form the boundaries of three fossae: the prezygapophyseal centroparapophyseal fossa, which is much deeper, and the centrodiapophyseal and postzygapophyseal centrodiapophyseal fossae (Figures 2b and S1). As in the preceding vertebra, the centropostzygapophyseal lamina forms a concave caudal margin in lateral view, but the part of the pedicel below it is shallower. The proximal articulation of the associated right rib is composed of a craniocaudally wider tuberculum, connected but unfused to the diapophysis, and a more gracile capitulum that expands more medially to reach the parapophysis. An aperture (“ap” in Figures 2 and S1) is formed between the capitulum and the tuberculum/diapophysis. Martill et al. (2000) identified this rib as possibly parts of an ossified iliosacral ligament.

The sacral centra of *M. asymmetrica* are co‐ossified to one another, although suture lines are visible between the elements, as are the neurocentral sutures. They are amphicoelous, as is the last trunk vertebra, with ventral transverse striations near the articular facets. The first two elements are nearly of the same size and stouter (craniocaudally shorter and lateromedially broader) than the last trunk centrum. They are spool‐shaped like that element, but much less lateromedially compressed at the middle (especially the second centrum). The cranial articulation of the first sacral centrum is lateromedially narrower and dorsoventrally deeper than the caudal, whereas that of the following centrum is both deeper and broader compared to its caudal facet. This caudal reduction of centrum depth leads to a dorsal sloping of the ventral surface of the cranial (as preserved) part of the sacrum. The ventral surfaces of the sacral centra are slightly flattened and covered by faint longitudinal striations. Each also bears a shallow midline sulcus, restricted to the caudal portion in the first centrum, but extending along the entire surface of the second element and entering the third centrum. Not much else can be said about the latter element, which is represented only by its cranial third, except that its shape mostly matches that of the previous vertebra.

The sacral neural spines are craniocaudally expanded towards their dorsal margins, but not as abruptly as in the preserved trunk vertebrae. The base of the second neural spine is craniocaudally broader than that of the first and its caudodorsal corner is missing. As in the last trunk vertebra, the lateral surface of the first sacral neural spine has a central, dorsoventrally elongated concavity (“nslc” in Figures 2 and S1), caudally bound by a vertical ridge that forms the spinopostzygapophyseal lamina. The base of that concavity is particularly excavated in that vertebra, whereas the second sacral element lacks the concavity altogether, its neural spine bearing a flat lateral surface instead. As in the last trunk vertebra, but not in the second sacral element, a postspinal lamina divides the dorsal part of the spinopostzygapophyseal fossa in the first sacral vertebra. Unlike those of the trunk vertebrae, the preserved sacral neural arches do not cover the entire dorsal surface of the respective centrum, but only about their cranial two‐fifths. Their caudal fifth is occupied by the rib of the following vertebra, with the space between that and the arch filled by the subcircular intervertebral foramen (“ivf” in Figures 2 and S1), which is larger in the second element. These craniocaudally shortened neural arches have less conspicuous laminae and fossae surrounding the ribs. The very deep prezygapophyseal centrodiapophyseal fossa—that is, the foramen opening into the lumen sensu Martill et al. (2000)—of the first element is floored by the base of its rib and roofed by a short prezygodiapophyseal lamina. Given the position of their ribs, those lamina and fossa are obliterated in the next two vertebrae. Inconspicuous caudal centrodiapophyseal and postzygodiapophyseal laminae are seen in the two first sacral vertebrae, bounding the postzygapophyseal centrodiapophyseal fossa. These are shallower compared to those of the trunk vertebrae, but slightly deeper in the first sacral element. As for the centrodiapophyseal fossa, this is only visible as a subtle subtriangular depression below the rib of the first sacral vertebra. The prezygapophyses of the first sacral vertebra expand craniodorsally beyond the cranial articulation of the respective centrum. Their facets face dorsomedially and are not fused to the last trunk postzygapophyses. On the contrary, the prezygapophyses of the second and third sacral vertebrae are respectively fused to the postzygapophyses of the first and second elements, although their suture lines are still visible. These are set just above the intervertebral foramen, showing that these zygapophyseal articulations are more cranially placed compared to those of the preserved trunk vertebrae.

The sacral ribs expand strictly laterally in dorsal view and bear laterodorsally oriented articular facets. The first ones are more laterally expanded than those of the second sacral vertebra, whereas the caudally incomplete third sacral ribs are again more expanded. The iliac articulation of the first sacral rib is ovoid, with the long axis mainly craniocaudally oriented. That of the second rib is more elongated, with the long axis craniodorsally to caudoventrally oriented. The sacral ribs are all positioned towards the cranial portion of the respective vertebra, but the first of those is the only one that does not also articulate to the previous element. On the contrary, the ribs of the second and third sacral vertebrae are more cranially positioned, with their bases shared with the centra of the preceding vertebrae. Yet, *contra* Martill et al. (2000), those ribs are not fused to the neural arch of the preceding vertebrae, but restricted to their respective arches. In all these cases, despite their fusion, it is possible to identify the suture lines between the rib bases and the centra. Equivalent sutures, formed between the ribs and the respective neural arches, are harder to discern in dorsal view. Instead, the dorsal surfaces of each preserved sacral rib harbor a longitudinal ridge (“srdr” in Figures 2 and S1), which divides such surfaces into two elongated concavities (“srdg” in Figures 2 and S1), which have been interpreted as housing tendons (Martill et al., 2000). They form a nearly continuous structure, separated only by depressions positioned above the postzygapophyses of the two first sacral vertebrae.

*Santanaraptor placidus* includes four tail vertebrae from the mid‐distal portion of the series (Figure 5), one isolated and three articulated, the last of which is represented only by a portion of the left neural arch leading to the prezygapophysis. They have similar sizes, but the isolated vertebra is stouter, with a slightly higher neural arch, suggesting a more proximal position in the tail. Like the *M. asymmetrica* vertebrae, the neural arches and centra of the *S. placidus* tail are not fused, with the sutures clearly visible in the articulated set and slightly displaced in the isolated element. The tail centra are amphicoelous and about 2.5 times longer than deep; there are no pleurocoels, but a few foramina pierce their lateral surfaces (“fm” in Figure 5). Their ventral surfaces are smooth, without ridges or grooves, and slightly concave in lateral view. Kellner (1999) described haemal arches associated with the tail of *S. placidus* as half the length of the centra, with craniocaudally expanded distal ends. Yet, these were not identified among the specimens studied by the senior author.

The tail neural arches are low, slightly shallower than the respective centrum, as are the neural spines. In the articulated set, those latter structures are composed of raised proximal and distal tips, separated by a concave margin, as seen in lateral view. The shorter proximal tip expends proximodorsally, barely entering the interprezygapophyseal space. Although not fully preserved in either vertebra, the distal tip is further elongated, expanding distal to the postzygapophyses. The proximal tip is not preserved, perhaps absent or much reduced in the isolated vertebra. Yet, the portion of its spine that corresponds to the distal tip in the articulated set is better developed, raising dorsally since the proximal half of the neural arch and expanding further dorsal to the postzygapophyses; what also suggests a more proximal position in the tail. The transverse processes form horizontally held laminae, the lateral margins of which are not complete in any of the specimens. Yet, those of the isolated element seem longer and are more proximally placed in the centrum, whereas those of the articulated set are more distally placed and angled. There are no fossae or large pneumatic perforations in the neural arches, but a relatively large foramen is seen below the left transverse process of the first articulated vertebra. The prezygapophyses expand proximally, forming angles of, respectively, ca. 30° and 20° relative to the horizontal and sagittal planes. When articulated, they expand proximal to the level of the postzygapophyseal articular facets, but overlap less than one‐fourth of the previous centrum length. They laterally conceal the postzygapophyses that, consequently, are not as laterally expanded, although forming a steeper angle to the sagittal plane. In fact, those zygapophyses are held on stalks along with the distal tip of the neural spine, with their distal margins barely reaching those of the centrum and neural spine.

#### 3.1.2 Pelvic girdle

**FIGURE 7:**
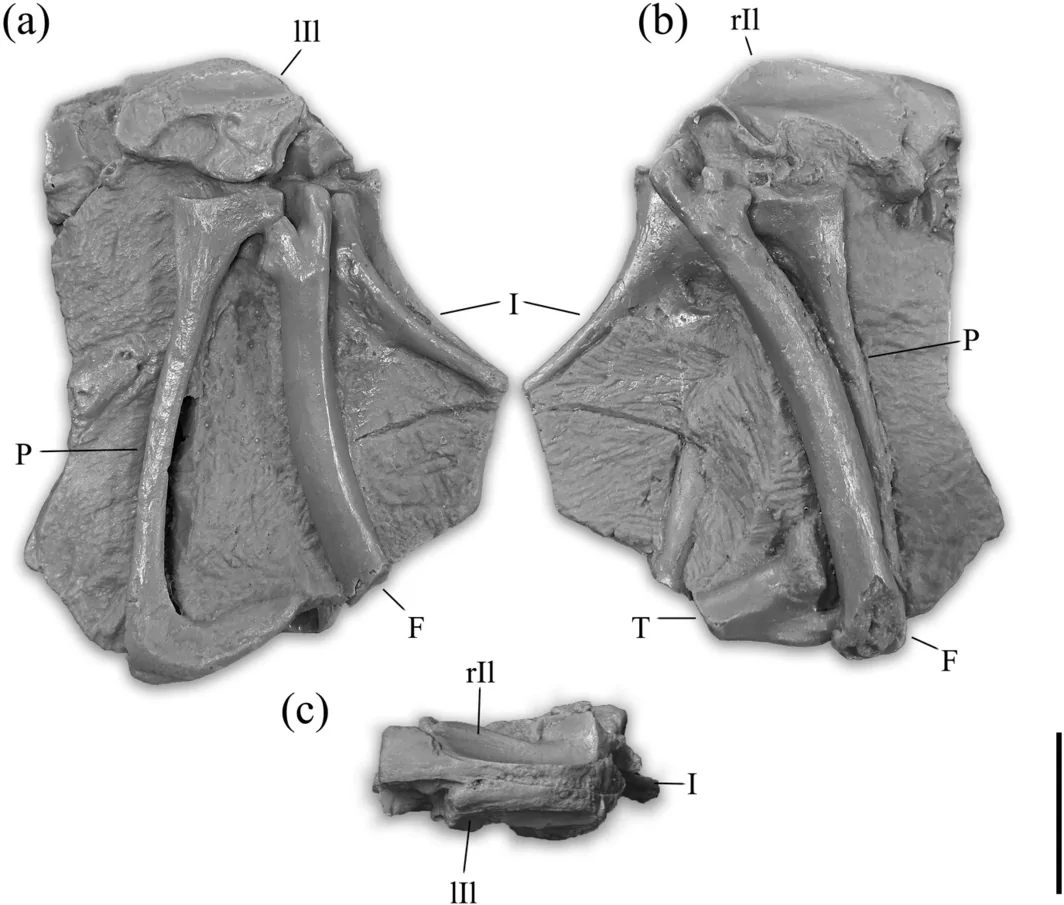
*Mirischia asymmetrica* (SMNK 2349 PAL). Cast before the preparation in (a) right lateral, (b) left lateral, and (c) dorsal views. F, femur; I, ischium; lIl, left ilium; P, pubis; rIl, right ilium; T, tibia. Scale bar equals 5 cm.

**FIGURE 8:**
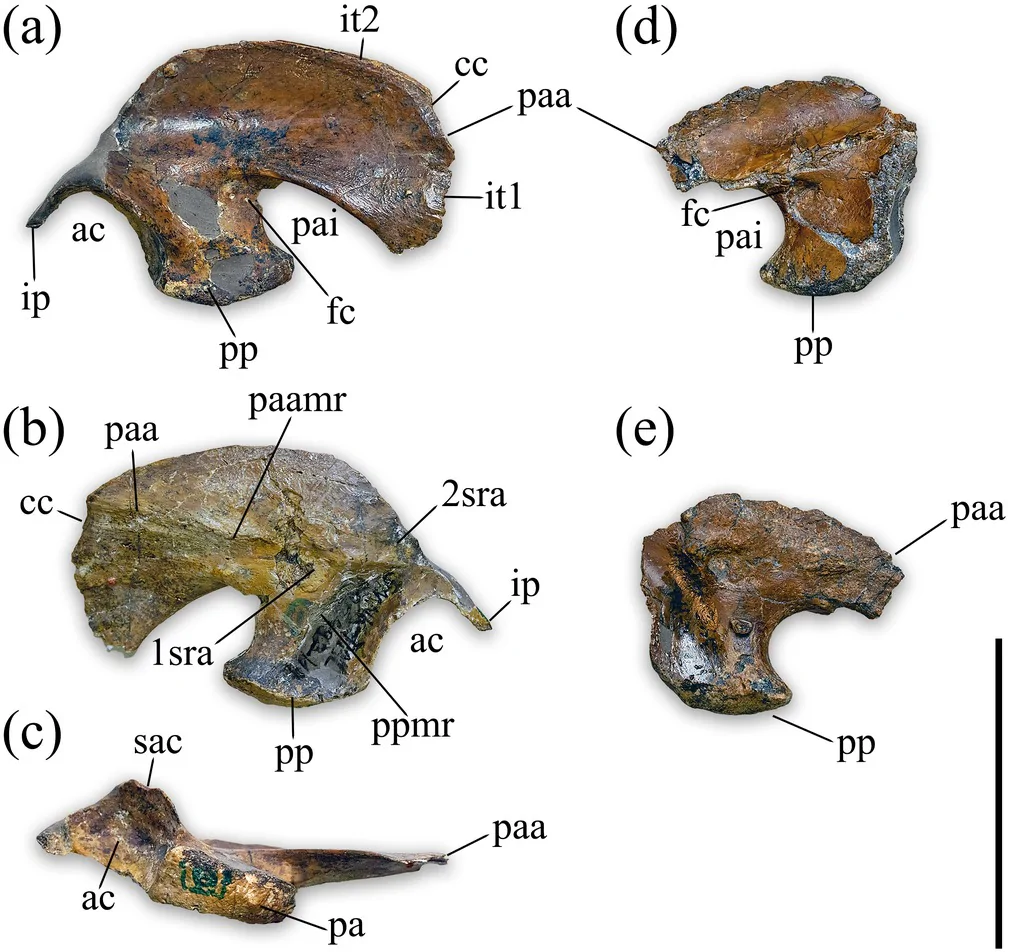
*Mirischia asymmetrica* (SMNK 2349 PAL). Right (a–c) and left (d–e) ilia in (a, d) lateral, (b, e) medial, and (c) medial views. 2sra; ac, acetabulum; cc, concavity; ip, ischiadic peduncle; it1‐2 origins of *M. iliotibialis* 1 and 2; fc, fossa cuppedicus; pa, pubic articulation; paa, preacetabular ala; paamr, preacetabular ala medial ridge; pai, preacetabular incisure; pp, pubic peduncle; ppmr, pubic peduncle medial ridge; sac, supraacetabular crest; sra, articulation facets for sacral ribs 1 and 2. Scale bar equals 5 cm.

**FIGURE 9:**
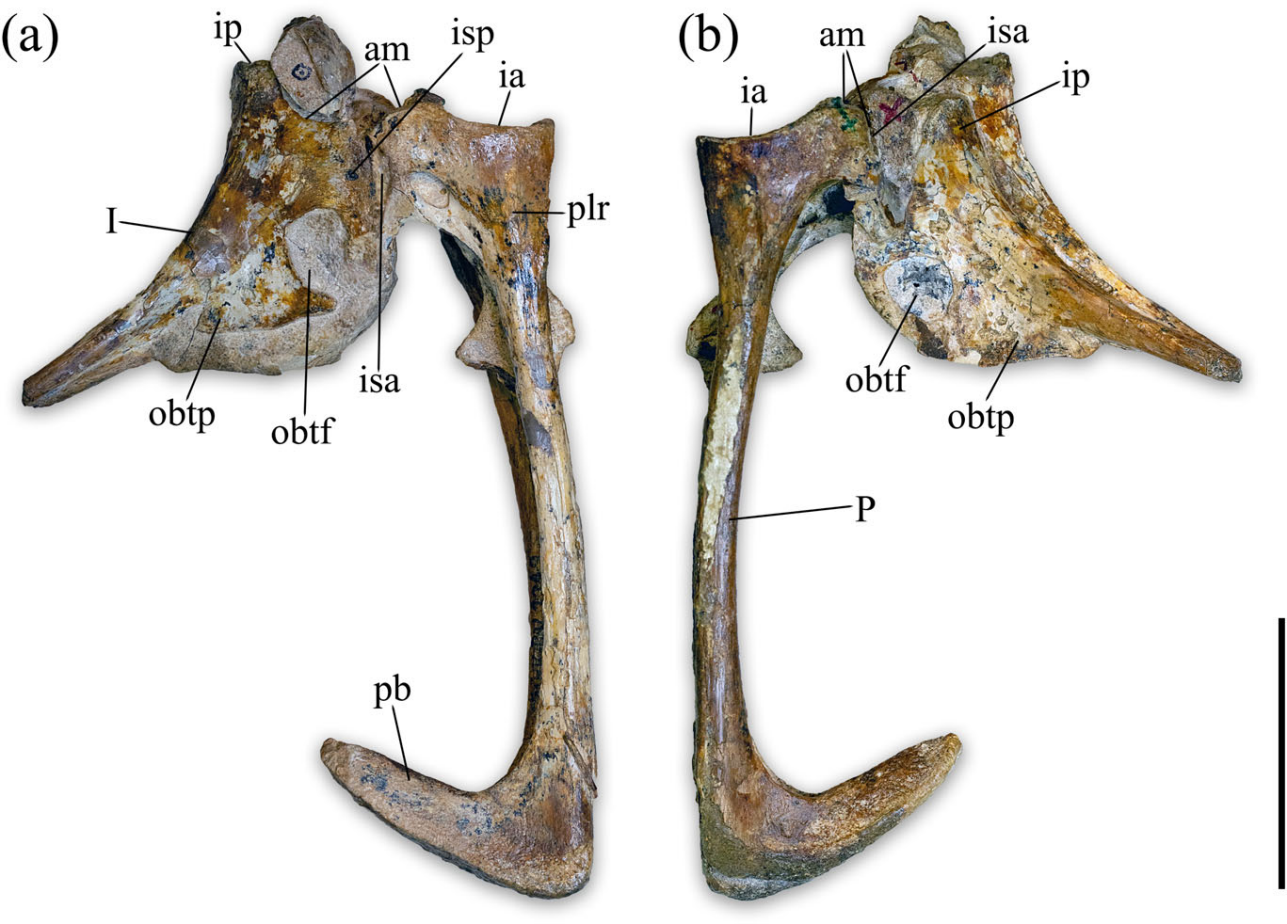
*Mirischia asymmetrica* (SMNK 2349 PAL). Articulated pubes and ischia in (a) right and (b) left lateral views. am, acetabular margin; I, ischium; ia, iliac articulation; ip, iliac peduncle; isa, ischiadic articulation; isp, ischiadic peduncle; obtf, obtp, obturator foramen; obtp, obturator plate; of; P, pubis; pb, pubic boot; plr, pubic lateral ridge. Scale bar equals 5 cm.

**FIGURE 10:**
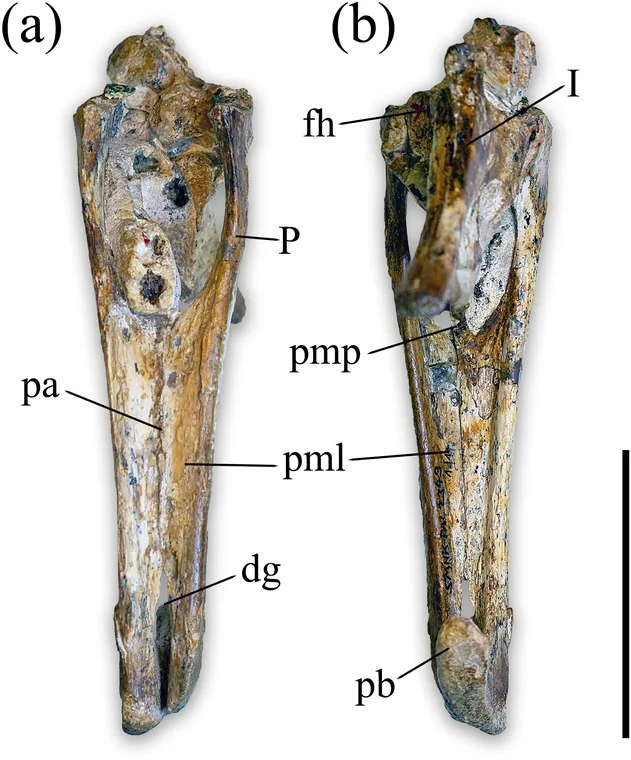
*Mirischia asymmetrica* (SMNK 2349 PAL). Articulated pubes and ischia in (a) cranial and (b) caudal views. dg, distal gap; fh, femoral head; I, ischium; P, pubis; pa, pubic apron; pb, pubic boot; pml, pubis medial lamina; pmp, pubic midline process. Scale bar equals 5 cm.

**FIGURE 11:**
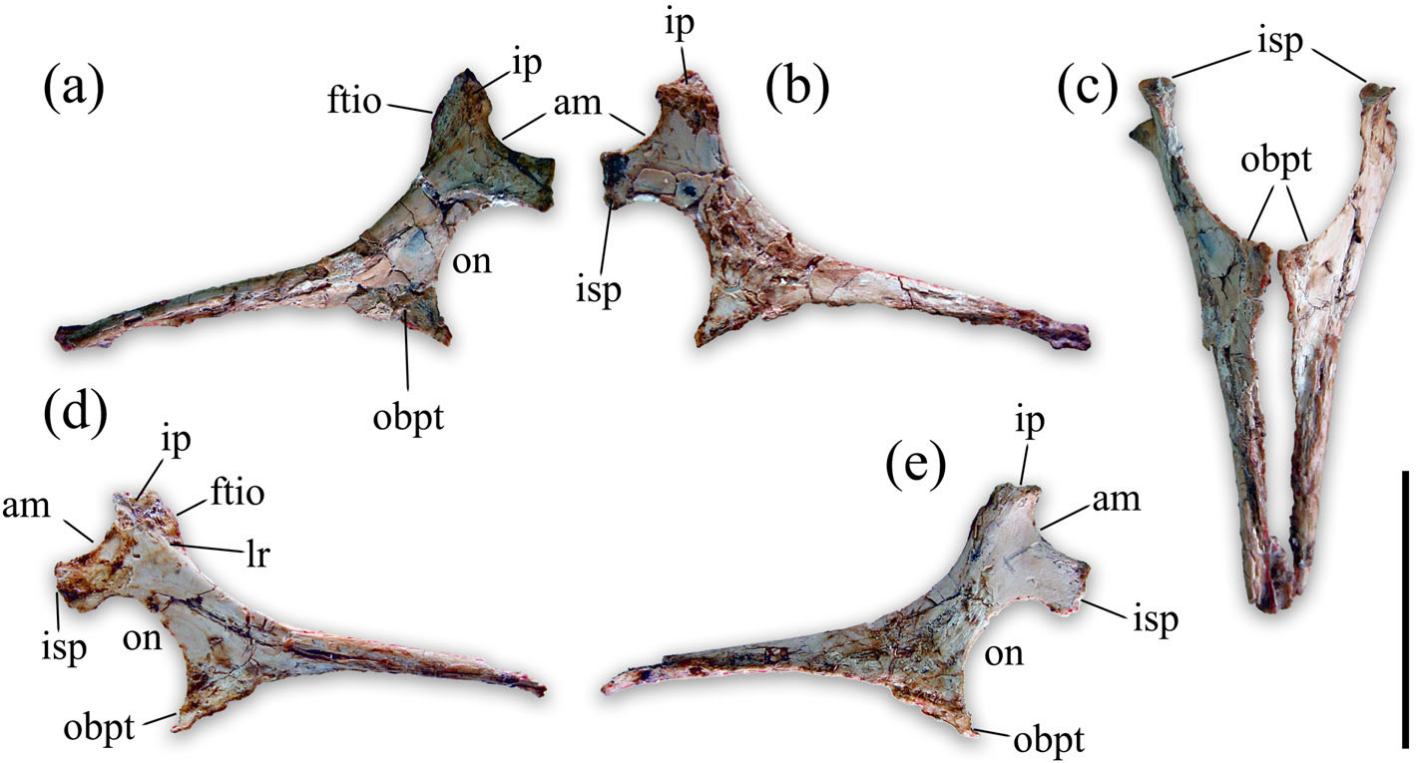
*Santanaraptor placidus* (MN 4802‐V). Right (a–c) and left (c–e) ischia in (a, d) lateral, (b, e) medial, and (c) ventral views. am, acetabular margin; ftio, origin of M. flexor tibialis internus 3; ip, iliac peduncle; isp, ischiatic peduncle; lr, longitudinal ridge; obpt, obturator plate; on, obturator notch. Scale bar equals 5 cm.

The pelvic girdle is more complete in *M. asymmetrica* than in *S. placidus*. The former includes both pubes and ischia, as well as the cranial portions of both ilia (Figures 7, 8, 9, 10). Only the ischia are preserved in *S. placidus* (Figure 11), which nonetheless allows comparing both specimens. The right ilium of *M. asymmetrica* is more complete and forms the basis of this description. It includes part of the central body, leading to most of the preacetabular ala, the entire pubic peduncle, and a small portion of the acetabular margin of the ischiadic peduncle. The dorsal margin of the ilium is complete until about the level of the caudal margin of the preacetabular incisure. Caudal to that, for about the craniocaudal length of the pubic peduncle, the preserved margin seems to roughly follow the original contour of the bone. Yet, from that point, a fracture extends until the caudodorsal margin of the acetabulum, missing all iliac parts caudal to that. The preserved dorsal margin of the ilium is slightly convex, with a markedly striated lateral border interpreted as the origin of m. *iliotibialis 2* (Bates et al., 2012; Carrano & Hutchinson, 2002). That origin extends cranioventrally along the dorsal half of the cranial margin of the ala, whereas the more verticalized and convex margin ventral to that harbored the origin of m. *iliotibialis 1* (Bates et al., 2012; Carrano & Hutchinson, 2002), which is broader and marked by coarser striations. Indeed, the boundary separating the two muscles corresponds to subtle concavity seen in the craniodorsal corner of the ala (“cc” in Figure 8). This is not a breakage, unlike suggested by Brusatte et al. (2014), and is set in the same position as the notch common to tyrannosauroids (Brochu, 2003; Xu et al., 2006). The lateral surface of the preserved supracetabular portion of the ilium is dorsoventrally concave, hosting the origin of m. *iliofemoralis externus*, but becomes flatter towards the cranioventral tip of the preacetabular ala. Yet, the vertical ridge that marks the caudal end of that muscle attachment (Carrano & Hutchinson, 2002) is not seen, probably because that part of the bone is missing. The cranial portion of the ala is also slightly deeper dorsoventrally and its ventral margin is concave, accompanying the convexity of the dorsal margin, so that the ala as a whole is hook‐shaped, curving ventrally towards its cranial end to nearly reach the ventral level of the pubic articulation. The ventral margin of the preacetabular ala extends caudally as a ridge along the lateral surface of the ilium. This marks the dorsal edge of the m. *iliofemoralis internus* origin (i.e., *fossa cuppedicus*) on the pubic peduncle (“fc” in Figure 8), which hosts two large foramina on the right and one on the left side. Similarly, the cranial margin of the peduncle extends craniodorsally as a ridge along the medial surface of the ala. In dorsal view, the preacetabular ala curves laterally towards its cranial end. In articulation, the dorsal margins of the ilia are rather close to one another in the center of the bone (Figure 7), separated by a space equivalent to the lateral expansion of both the supracetabular crest and cranioventral tip of the ala.

The pubic peduncle is longer than broad in lateral/medial views, with a convex ventral margin. Its ventral end expands cranially, accentuating its already concave cranial margin. The external margin of the peduncle is craniocaudally convex, whereas its articular facet is slightly excavated caudally, but flat cranially. Its ventral outline is subrectangular, with the cranial portion narrower than the caudal. As preserved, the acetabulum has the shape of a pointed dome and is almost fully opened, except for a narrow wall on its cranial margin. Its dorsal margin is marked by a very projected supracetabular crest, almost as expanded as the acetabular is dorsoventrally deep, reaching more laterally than the cranioventral tip of the preacetabular ala. The breakage through the supracetabular crest shows that it is wedge‐shaped in cross‐section, and its dorsal/ventral outline is rounded. Of the ischial peduncle, only part of its acetabular margin is preserved. Yet, if this corresponds to most of its ventral expansion, that element would be significantly shorter than the pubic peduncle. The medial surface of the preacetabular ala is almost flat and covered with thin striations, mostly craniocaudally oriented. For the rest of the medial surface of the bone, a ridge (“ppmr” in Figure 8) longitudinally crosses the center of the pubic peduncle, on a cranioventral to caudodorsal direction, dorsal to which it meets a subhorizontal ridge (“paamr” in Figure 8) coming from the preacetabular ala in the direction of the dorsal end of the acetabulum, so that they form a triradiate set of ridges. The articulation areas of the two first sacral ribs are clearly identified, based on both their scars on the medial surface of the ilium and relative positions inferred from the comparison of the articulated vertebra and the original position of the bones. The facet for the first sacral rib is rounded and positioned right caudal to the ventral part of the preacetabular ala and above the cranial part of the pubic peduncle. Only the cranial part of the second sacral rib articulation is preserved in the right ilium, above the part of the horizontal ridge that is itself positioned above the acetabulum.

Both pubes of *M. asymmetrica* (Figures 9 and 10) are slender elements that form angles of about 55° to the long axis of the sacrum and 70° to that of the ischium. In lateral view, the cranial margin of the pubic body is straight, with the shaft distal to that arching slightly cranially. In cranial view, the proximal portion of the pubis arches outwards, whereas the shaft is straight, but gets closer to its pair as it extends distally. The proximal margin of the pubis bears a concave (in lateral/medial views) cranial portion that matches the size of the distal articulation of the pubic peduncle of the ilium, representing the articulation between these two bones (“ia” in Figure 9). Caudal to that, the pubic body is subrectangular, almost hatch‐shaped. Its dorsal margin takes part in the acetabulum and faces slightly caudally (“am” in Figure 9), whereas the caudal margin forms a sigmoid (in lateral/medial views) articular facet for the ischium (“isa” in Figure 9). The obturator flange expands ventrally from the pubic body, forming a laminate structure that merges distally to the ventral margin of the pubic shaft. An ovoid obturator foramen, with the long axis subparallel to that of the shaft, perforates the flange on the right side, whereas the left flange bears a notch. This part of the left flange is here considered lost (*contra* Naish et al., 2004) possibly due to preparation, so that it would also have originally delimited an obturator foramen (Figure S2). The lateral surface of the pubic body is traversed by a subtle ridge (“plr” in Figure 9), the rugose proximal portion of which likely received the origin of m. *ambiens* (Bates et al., 2012; Carrano & Hutchinson, 2002). It extends distally, entering the proximal portion of the shaft, which is lateromedially flattened. Distal to that, the pubes are conjoined by their medial laminae (“pml” in Figure 10), which extend from the dorsal margin of the rod‐like shafts to meet one another at the midline, forming the pubic apron; consequently, most of the distal two‐thirds of the pubis is flat cranially and concave caudally. The proximal margin of the apron is w‐shaped, due to the presence of a proximally expanded midline process (“pmp” in Figure 10). Because the shafts come closer distally, the laminae get lateromedially narrower in the same direction, not reaching the distal end of the bone. At this point, they form the proximal margin of a proximodistally elongated gap (“dg” in Figure 10), set at the midline to separate the cranial portion of the distal ends of the pubes, possibly accommodating a duct leading to a post‐pubic air sac (Martill et al., 2000). Yet, caudal to that, the distal end of the pubis extends caudally to meet and co‐ossify with its pair, forming a large pubic boot. The lateral surface of the boot is depressed, whereas its dorsal surface bears a pair of ridges that extend along the caudal surface of the distal portion of the shafts. The distal margin of the boot is flat in lateral view, with a subtriangular distal outline, broader cranially than caudally.

The ischium is the only pelvic element common to both *M. asymmetrica* and *S. placidus* (Figures 9, 10, 11). They are isolated in the latter and articulated to the pelvis in the former, but neither has a complete distal end, although those of *S. placidus* are more so. The ischium is also the common skeletal part that differs the most between the two specimens, providing evidence that they belong to different species. As with the pubis, the ischium is composed of a main body, the laminar obturator plate, and a rod‐like shaft. In lateral/medial views, the acetabular margin is strongly concave, with its caudodorsal margin formed by a subrectangular iliac peduncle. The laterodorsal surface of the peduncle is marked by a longitudinal crest, that extends distally, forming the laterodorsal edge of the shaft; this is more marked in *S. placidus* (especially in the left side; “lr” in Figure 11) than in *M. asymmetrica*. Also, in lateral/medial views, the dorsal margin of the peduncle is mostly concave in *M. asymmetrica*, whereas *S. placidu*s bears a rugose protuberance (“ftio” in Figure 11) for the origin of m. *flexor tibialis internus 3* (Carrano & Hutchinson, 2002; Rhodes et al., 2021). As such, the peduncle of the latter specimen is somewhat cranially bent, with a more verticalized iliac articular facet. The ischial peduncle of *S. placidus* is subrectangular, whereas that of *M. asymmetrica* is dorsoventrally deeper and hatchet‐shaped. This is because its cranial portion projects more ventrally, forming more extensive pubic articulation and cranial edge of a relatively small obturator foramen. In fact, the ischium of *S. placidus* is completely devoid of such extension, so that it bears a wide obturator notch, rather than a foramen. Indeed, we agree with Brusatte et al. (Brusatte et al., 2014); (*contra* Martill et al., 2000, and Naish et al., 2004) that the right obturator plate of *M. asymmetrica* is broken, suggesting the presence of a fully enclosed obturator foramen, as in the left side. In fact, in the later side, the bone surface of the plate that closes the foramen was lost, probably due to overpreparation with chemical agents. The same, but more extensively, seems to have happened on the right side, as indicated by the ventrally truncated part of the plate cranial to the aperture, which was surely more extensive, reaching the part ventral to the aperture and enclosing the foramen. Hence, the obturator plate of *S. placidus* could be described as subtriangular/spur‐like and placed more distally along the ventral margin of the ischium. Yet, it clearly corresponds to the part of the plate that, in *M. asymmetrica*, is placed distal to the foramen. This part of the plate is more proximally placed on the *M. asymmetrica* ischium than in that of *S. placidus*. More distally, the plate merges smoothly onto the ventral surface of the shaft in *S. placidus*, extending as a thin, medioventral flange for about half the length of its preserved portion. Instead, the plate is not so distally extensive in *M. asymmetrica* and its distal margin ends more abruptly onto the shaft. Yet, it is important to note that, in the right ischium, the “deep notch” between the obturator plate and the shaft is actually a breakage in the bone. The shaft itself extends caudoventrally in both specimens. It is more lateromedially compressed in *S. placidus*, forming a low angle to the dorsal margin of the ischial body in lateral/medial views. That of *M. asymmetrica* is more rod‐like, its dorsal margin following a more even concavity with that of the ischial body.

#### 3.1.3 Hindlimb

**FIGURE 12:**
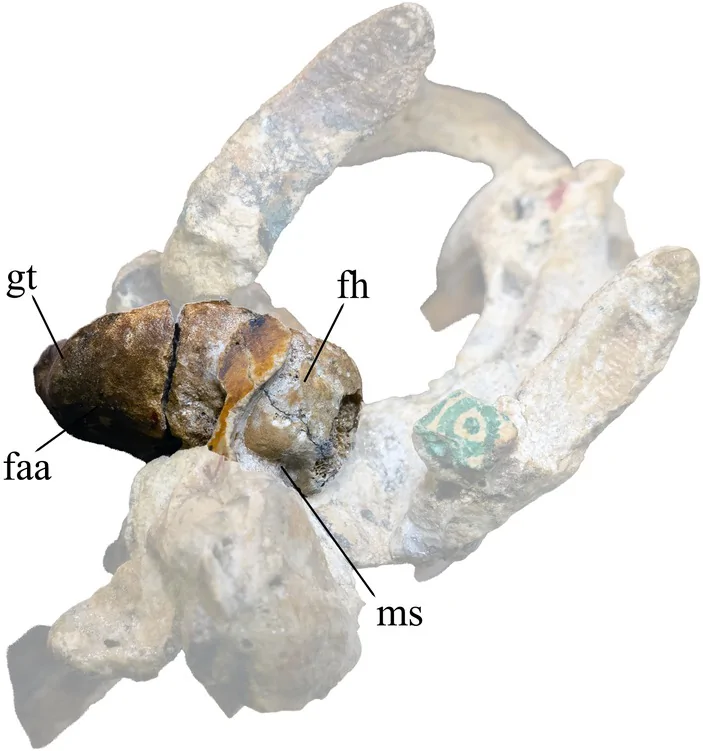
*Mirischia asymmetrica* (SMNK 2349 PAL). Detail of the left femoral head articulated with the pelvic elements. faa, facies articularis antitrochanterica; fh, femoral head; gt, greater trochanter; ms, medial sulcus for the capitate ligament. Without scale.

**FIGURE 13:**
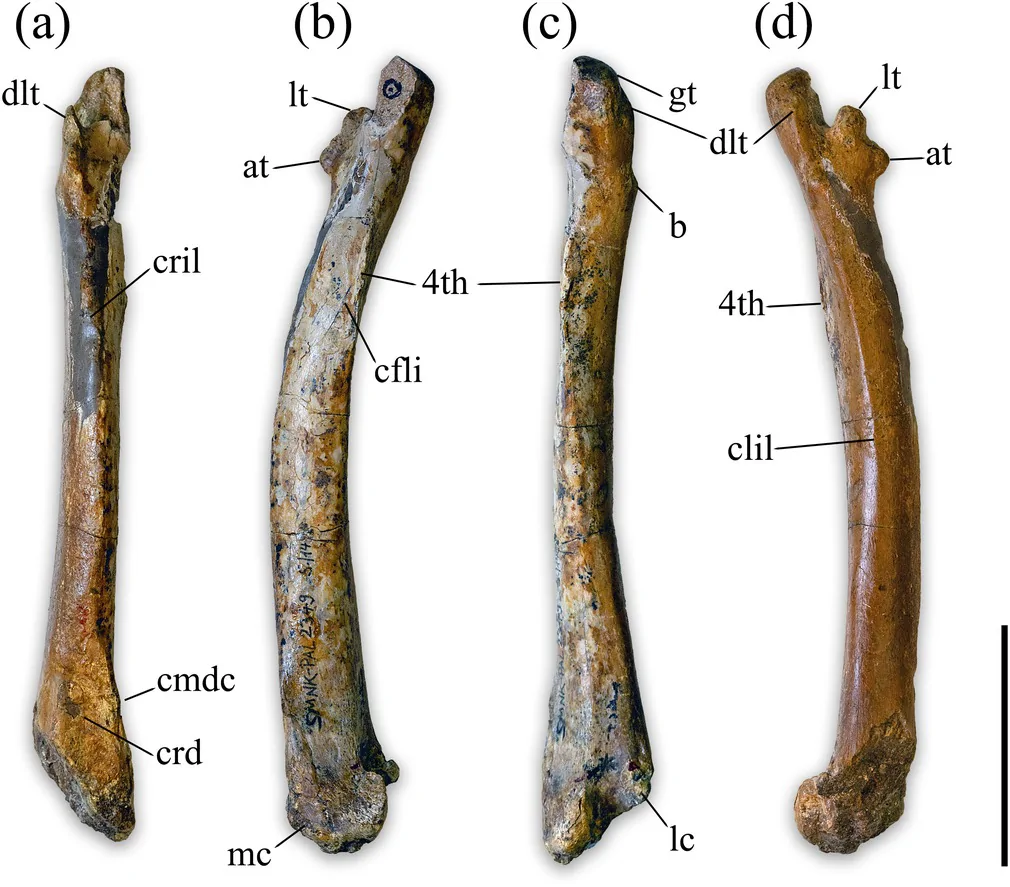
*Mirischia asymmetrica* (SMNK 2349 PAL). Right femur in (a) cranial, (b) medial, (c) caudal, and (d) lateral views. 4th, fourth trochanter; at, accessory trochanter; b, lateral bulge; cfli, insertion of M. caudofemoralis longus; clil, caudolateral intermuscular line; cmdc, craniomedial distal crest; crd, cranial depression; cril, cranial intermuscular line; dlt, dorsolateral trochanter; gt, greater trochanter; lc, lateral condyle; lt, lesser trochanter; mc, medial condyle. Scale bar equals 5 cm.

**FIGURE 14:**
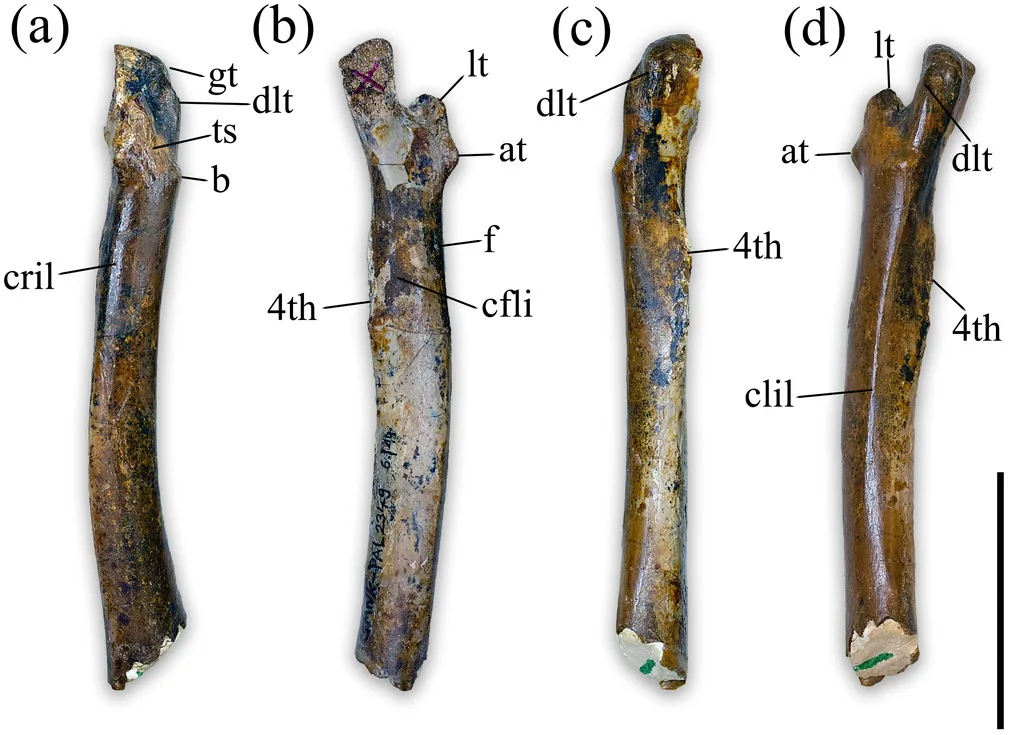
*Mirischia asymmetrica* (SMNK 2349 PAL). Left femur in (a) cranial, (b) medial, (c) caudal, and (d) lateral views. 4th, fourth trochanter; at, accessory trochanter; b, lateral bulge; cfli, insertion of M. caudofemoralis longus; clil, caudolateral intermuscular line; cril, cranial intermuscular line; dlt, dorsolateral trochanter; f, foramen; gt, greater trochanter; lt, lesser trochanter; ts, trochanteric shelf. Scale bar equals 5 cm.

**FIGURE 15:**
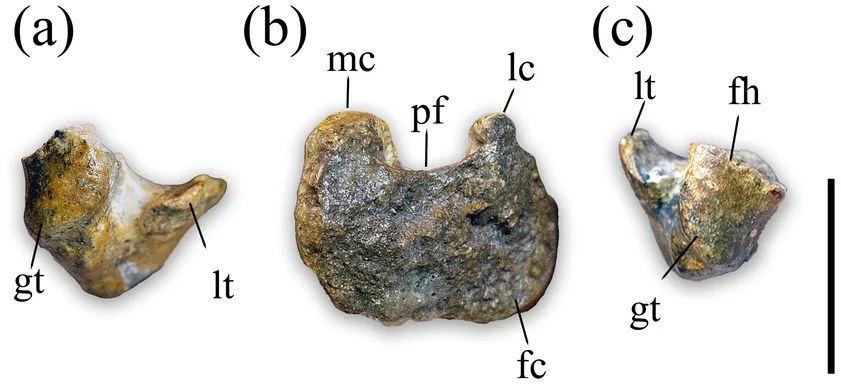
*Mirischia asymmetrica* (SMNK 2349 PAL). Right (a, b) and left (c) femora in (a, c) proximal and (b) distal views. fc, fibular condyle; fh, femoral head; gt, greater trochanter; lc, lateral condyle; lt, lesser trochanter; mc, medial condyle; pf, popliteal fossa. Scale bar equals 2 cm.

**FIGURE 16:**
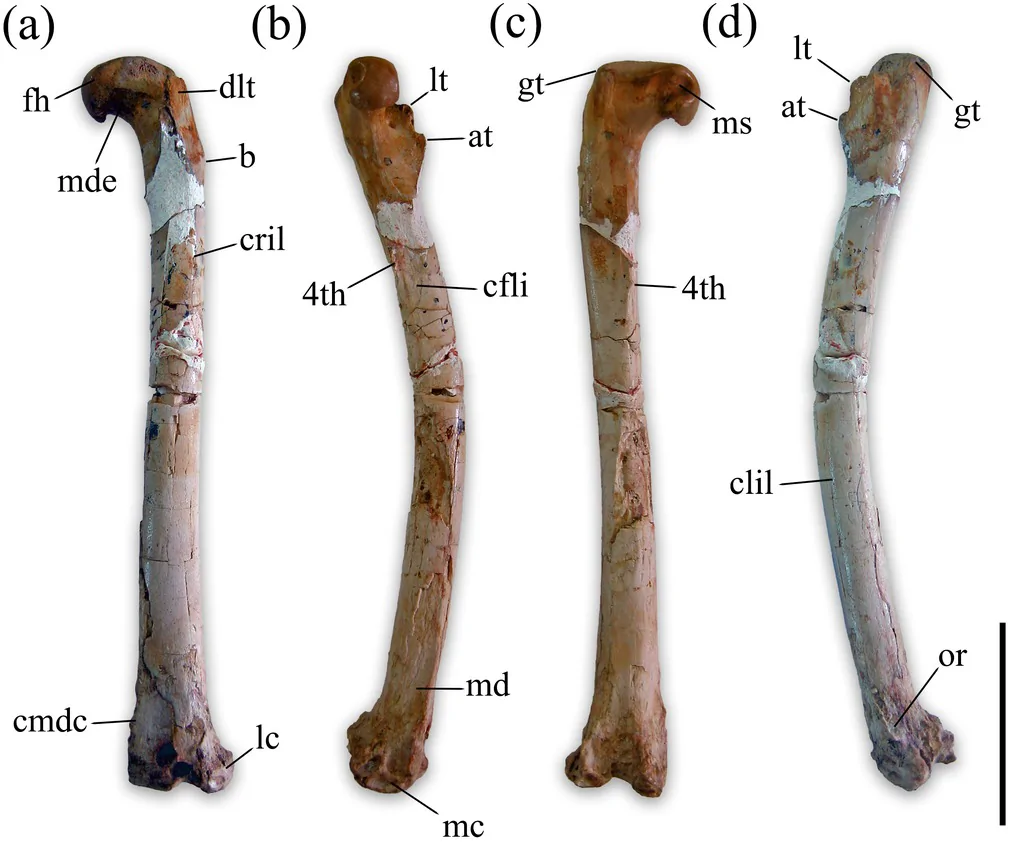
*Santanaraptor placidus* (MN 4802‐V). Left femur in (a) cranial, (b) medial, (c) caudal, and (d) lateral views. 4th, fourth trochanter; at, accessory trochanter; b, lateral bulge; cmdc, craniomedial distal crest; cfli, insertion of M. caudofemoralis longus; clil, caudolateral intermuscular line; cril, cranial intermuscular line; dlt, dorsolateral trochanter; fh, femoral head; gt, greater trochanter; lc, lateral condyle; lt, lesser trochanter; mc, medial condyle; md, medial depression; mde, mediodistal emargination; ms, medial sulcus for the capitate ligament; or, oval rugosity. Scale bar equals 5 cm.

**FIGURE 17:**
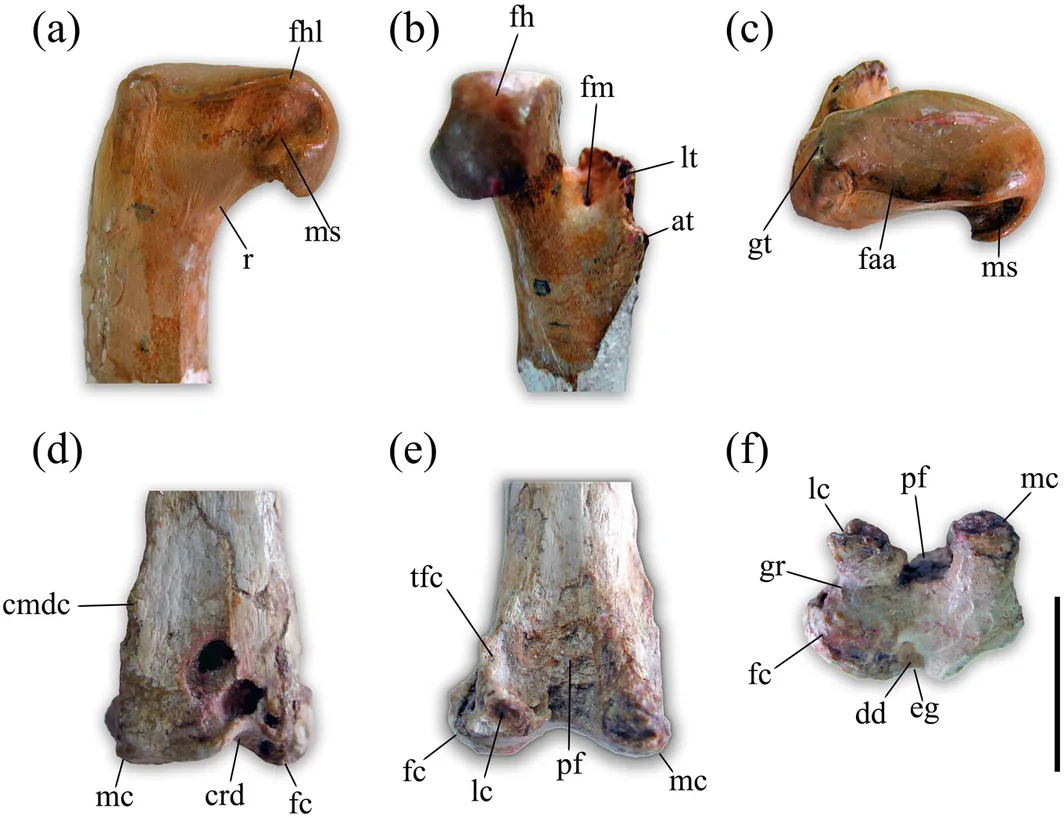
*Santanaraptor placidus* (MN 4802‐V). Proximal (a–c) and distal (d–f) ends of the left femur in (a, c, e) caudal, (b) medial, (d) cranial, and (f) distal views. at, accessory trochanter; cmdc, craniomedial distal crest; crd, cranial depression; dd, distal depression; eg, extensor groove; faa, facies articularis antitrochanterica; fc, fibular condyle; fh, femoral head; fhl, femoral head lip; fm, foramen; gr, groove; gt, greater trochanter; lc, lateral condyle; lt, lesser trochanter; mc, medial condyle; ms, medial sulcus for the capitate ligament; pf, popliteal fossa; r, ridge; tfc, crista tibiofibularis. Scale bar equals 5 cm.

**FIGURE 18:**
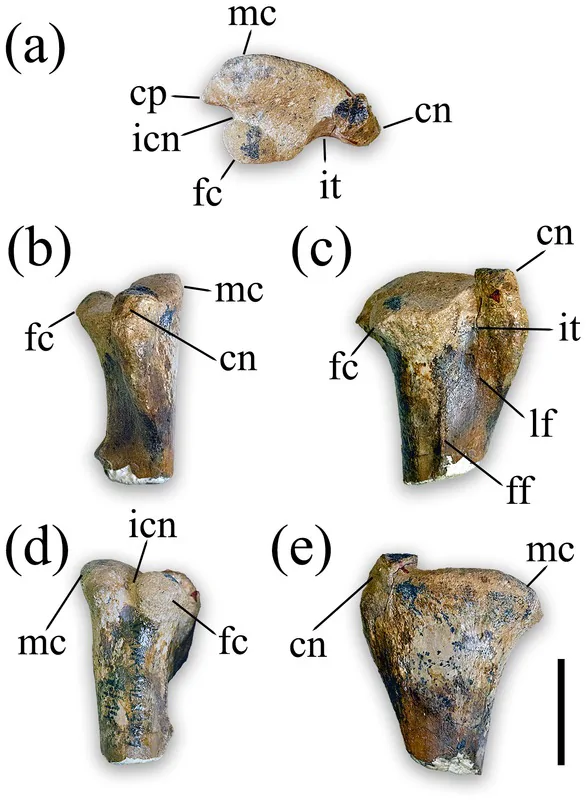
*Mirischia asymmetrica* (SMNK 2349 PAL). Proximal end of right tibia in (a) proximal, (b) cranial, (c) lateral, (d) caudal, and (e) medial views. cn, cnemial crest; cp, caudal process; fc, fibular condyle; ff, fibular flange; icn, intercondylar notch; it, incisura tibialis; lf, lateral fossa; mc, medial condyle. Scale bar equals 2 cm.

**FIGURE 19:**
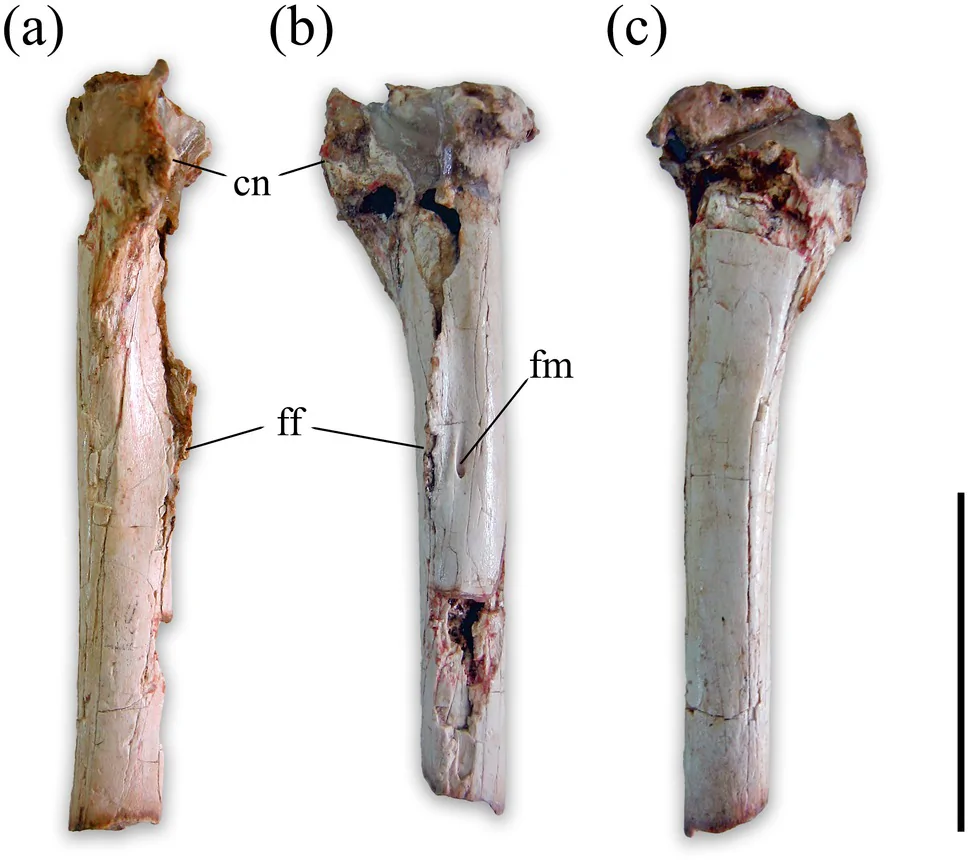
*Santanaraptor placidus* (MN 4802‐V). Left tibia in (a) cranial, (b) lateral, and (c) medial views. cn, cnemial crest; ff, fibular flange; fm, foramen. Scale bar equals 5 cm.

**FIGURE 20:**
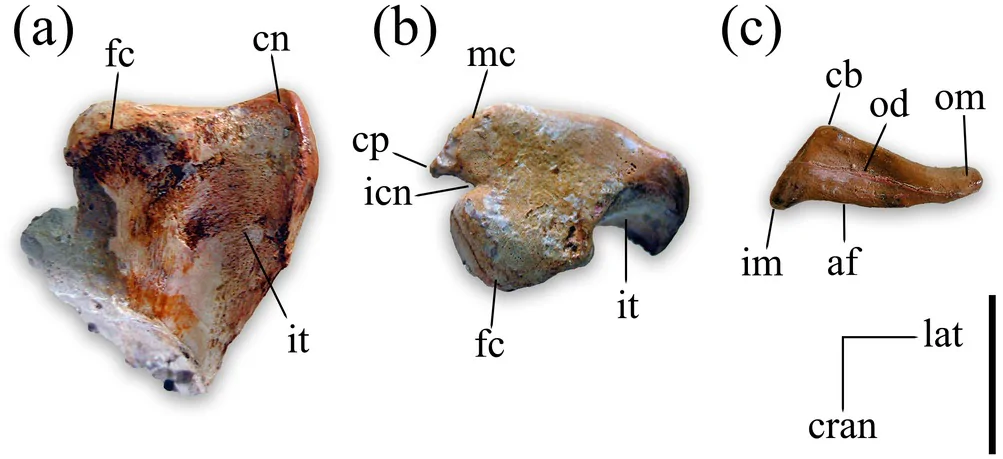
*Santanaraptor placidus* (MN 4802‐V). Proximal (a, b) and distal (c) ends of the right tibia in (a) lateral, (b) proximal, and (c) distal views. af, astragalar facet; cb, caudal buttress; cn, cnemial crest; cp, caudal process; cran, cranial; fc, fibular condyle; icn, intercondylar notch; im, inner malleolus; it, incisura tibialis; lat, lateral; mc, medial condyle; od, ovoid depression; om, outer malleolus. Scale bar equals 2 cm.

**FIGURE 21:**
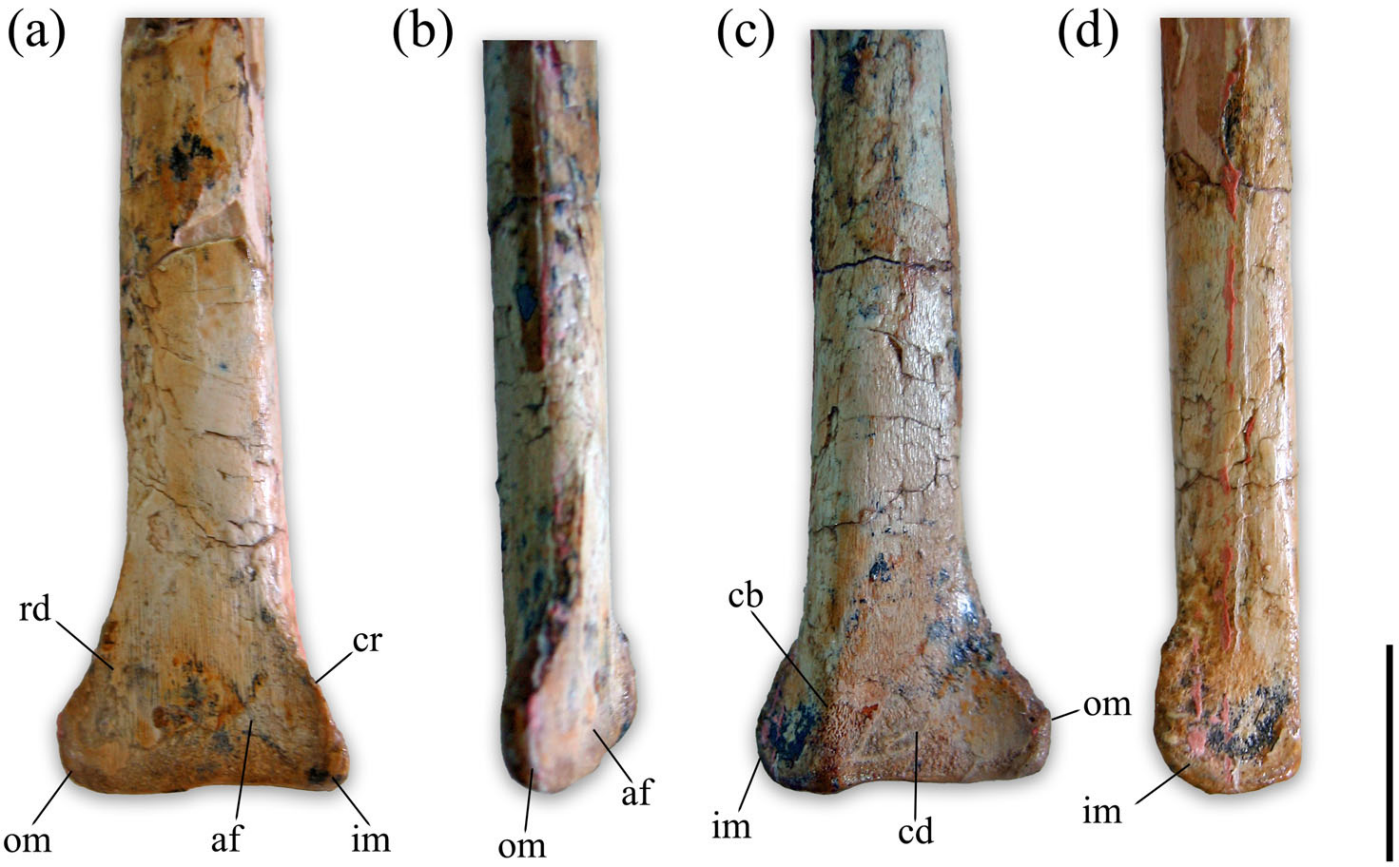
*Santanaraptor placidus* (MN 4802‐V). Distal end of right tibia in (a) cranial, (b) lateral, (c) caudal, and (d) medial views. af, astragalar facet; cb, caudal buttress; cd, caudal depression; cr, crest; im, inner malleolus; om, outer malleolus; rd, ridge. Scale bar equals 2 cm.

**FIGURE 22:**
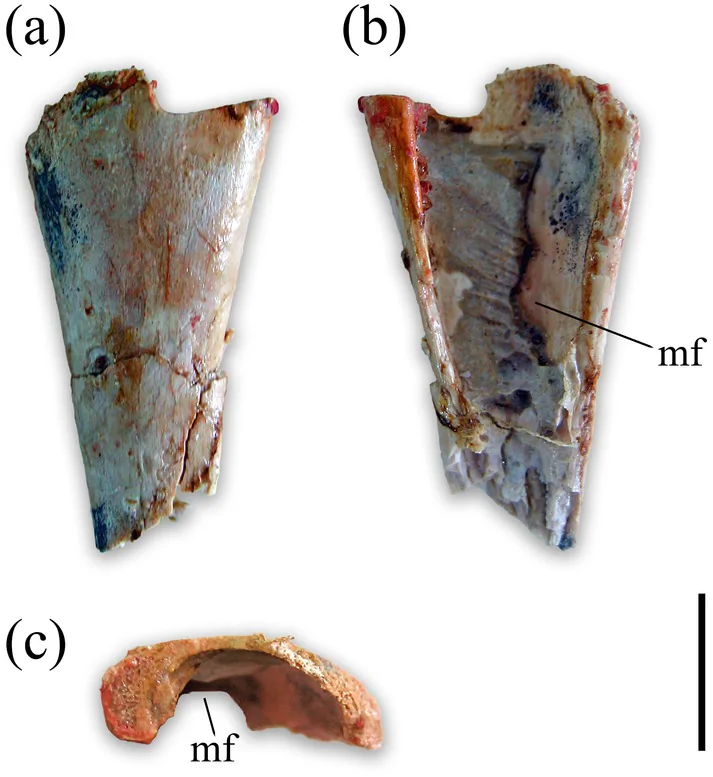
*Santanaraptor placidus* (MN 4802‐V). Possible proximal portion of left fibula in (a) lateral, (b) medial, and (c) proximal views. mf, medial fossa. Scale bar equals 1 cm.

**FIGURE 23:**
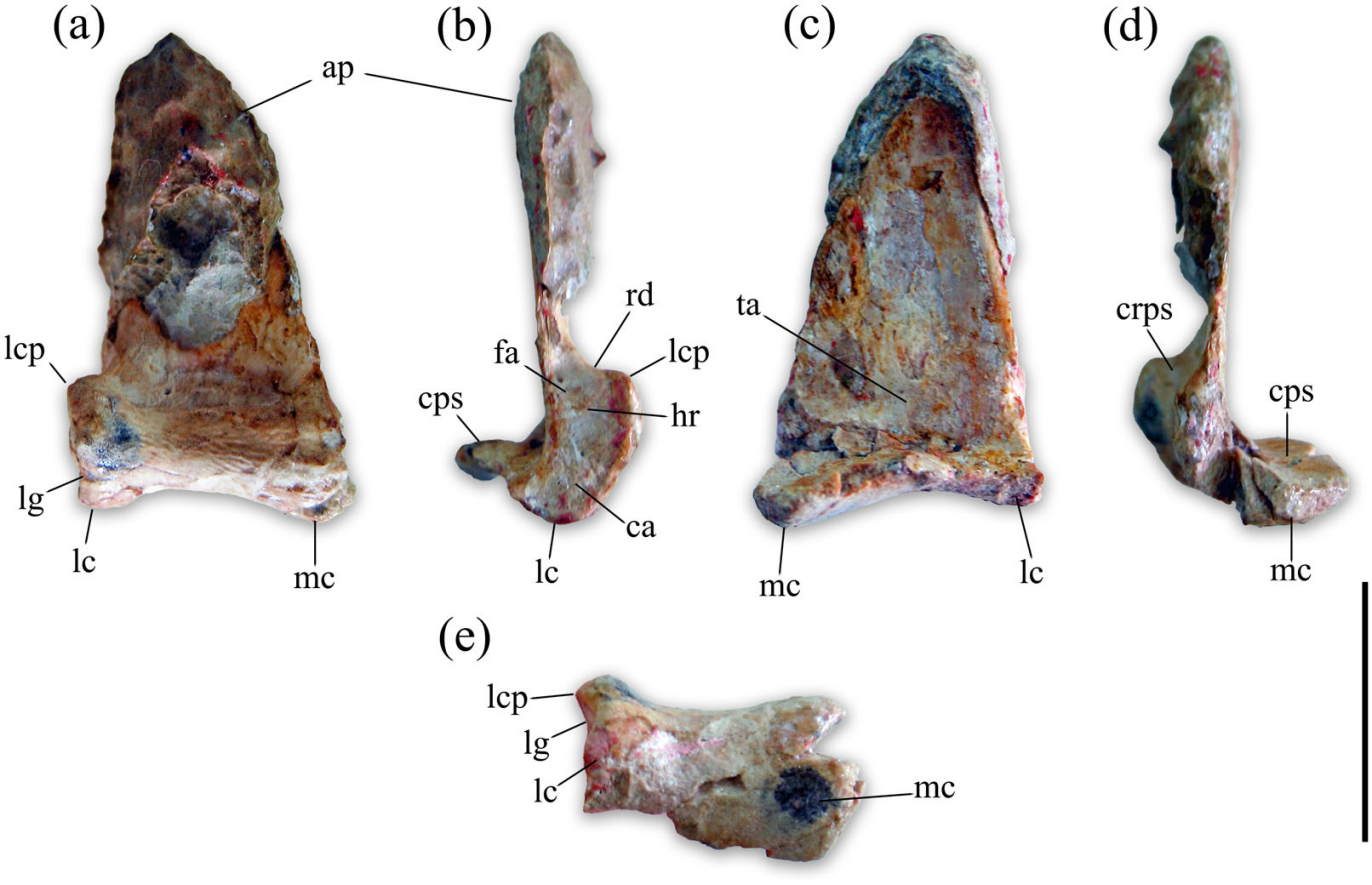
*Santanaraptor placidus* (MN 4802‐V). Right astragalus in (a) cranial, (b) lateral, (c) caudal, (d) medial, and (e) distal views. ap, ascending process; ca, calcaneum articulation; cps, caudal proximal surface; crps, cranial proximal surface; fa, fibula articulation; hr., horizontal ridge; lc, lateral condyle; lcp, laterocranial process; lg, lateral groove; mc, medial condyle; rd, ridge; ta, tibia articulation. Scale bar equals 2 cm.

**FIGURE 24:**
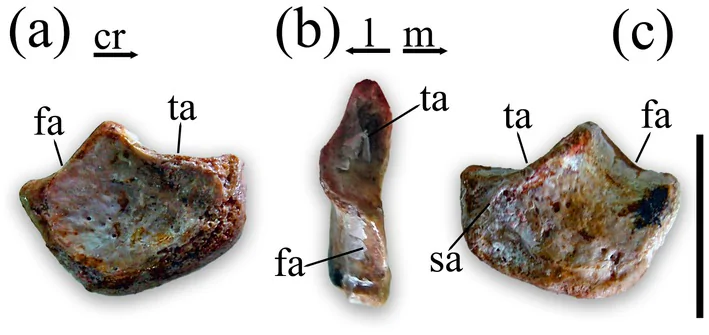
*Santanaraptor placidus* (MN 4802‐V). Right calcaneum in (a) lateral, (b) proximal, and (c) medial views. fa, fibula articulation; sa, scarred area; ta, tibia articulation. Scale bar equals 1 cm.

**FIGURE 25:**
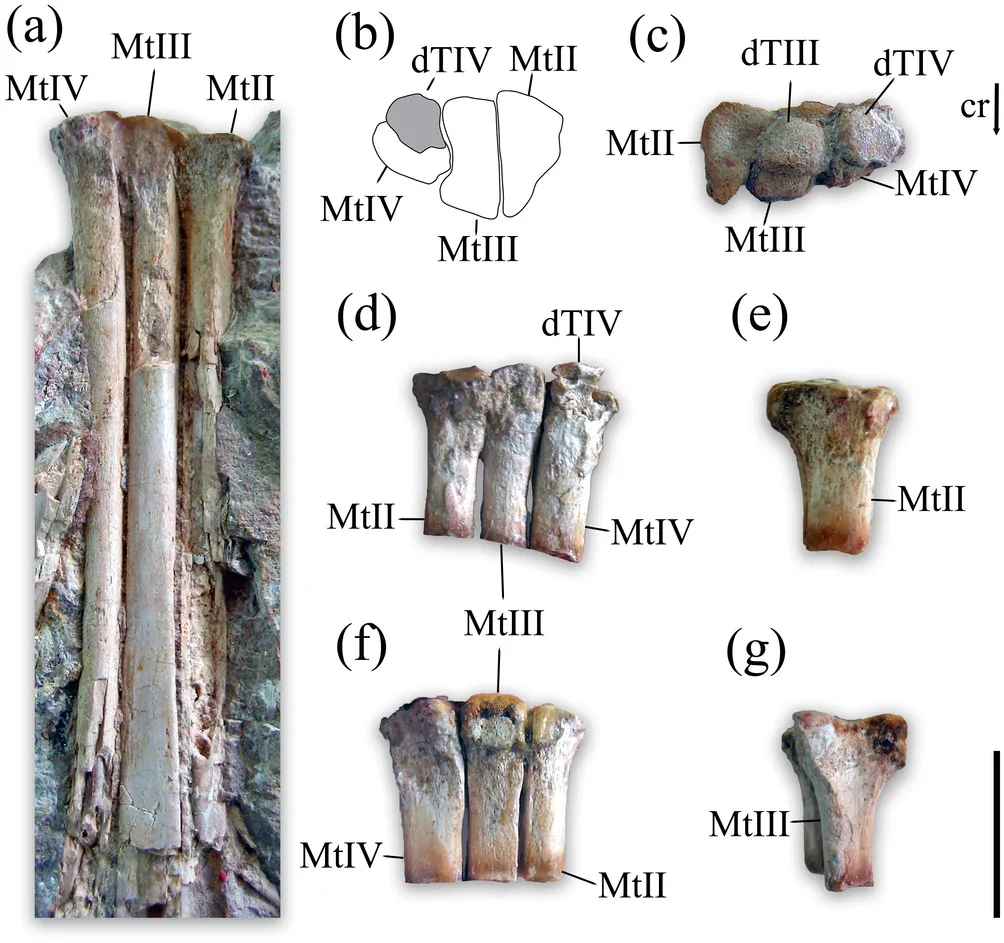
*Santanaraptor placidus* (MN 4802‐V). Right (a, c) and proximal portion of left (b, d–g) metatarsi in (a, d) cranial, (b, c) proximal, (e) medial, (f) caudal, and (g) lateral views. cran, cranial; dT, distal tarsal; Mt., metatarsal. Scale bar equals 2 cm.

**FIGURE 26:**
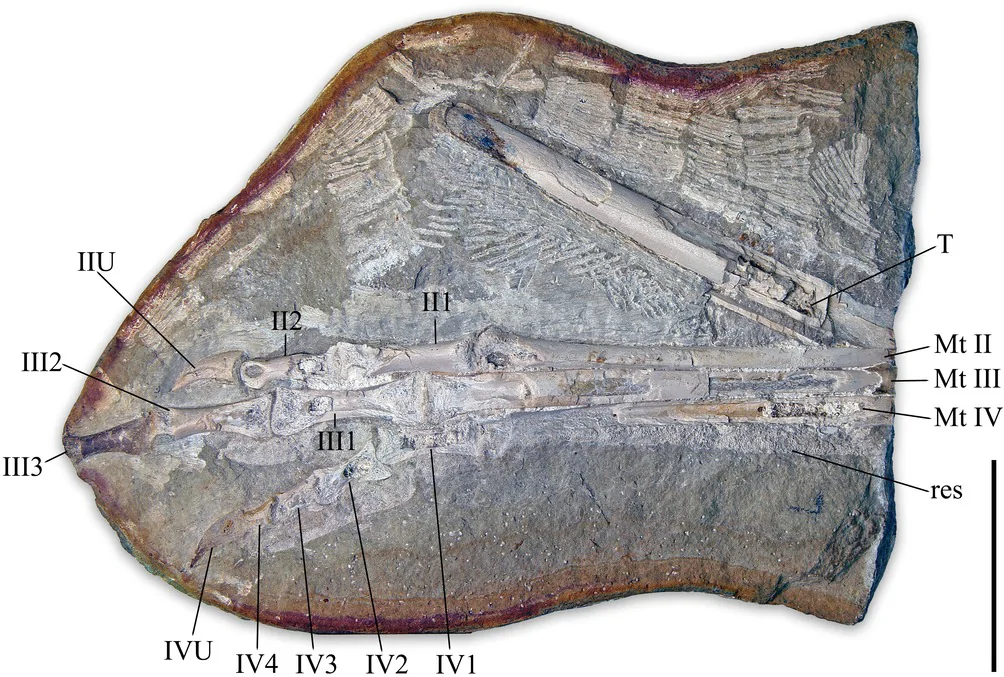
*Santanaraptor placidus* (MN 4802‐V). Articulated left pes. Mt, metatarsals; res, reticulated scales; T, left tibia; U, ungual phalanx. Roman numbers refer to digit numbers; Arabic numbers refer to phalanx numbers. Scale bar equals 5 cm.

**FIGURE 27:**
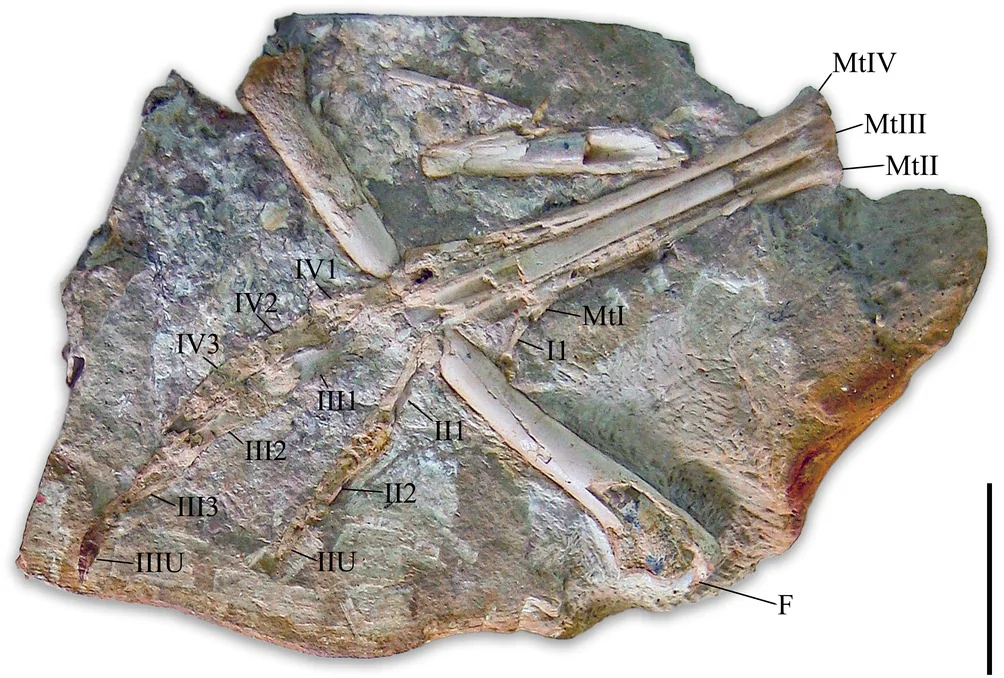
*Santanaraptor placidus* (MN 4802‐V). Articulated right pes. Mt, metatarsals; F, femur; U, ungual phalanx. Roman numbers refer to digit numbers; Arabic numbers refer to phalanx numbers. Scale bar equals 5 cm.

The hindlimb is partially preserved in both *M. asymmetrica* and *S. placidus*, with significant overlap among parts. The femora of *M. asymmetrica* were originally preserved articulated to the pelvis (Figures 7 and 12). Most of both bones (Figures 13, 14, 15) were prepared out, but separated from the medial part of their heads in the process, which were kept within the acetabulum. Apart from that, the right element is nearly complete, missing only the lateral portion of its distal end, whereas only about the proximal three‐fourths of the left femur is preserved. As for *S. placidus*, its left femur is nearly complete and prepared out of the matrix (Figures 16 and 17), whereas the right element was still partially within the bearing rock when last seen by the authors. Only the proximal end of the right tibia is preserved in *M. asymmetrica* (Figure 18), whereas *S. placidus* preserved both elements (Figures 19, 20, 21). This includes the isolated distal half of the right element and its proximal end still within the bearing rock, plus the isolated proximal half of the left element, but with a badly damaged proximal articulation. The fibula is represented only by a proximal fragment of the left bone in *S. placidus* (Figure 22; but see below). The tarsus and pes are preserved only in *S. placidus*, including isolated right astragalus and calcaneum (Figures 23 and 24), as well as distal tarsals III and IV of that side appressed to the metatarsals, as is also the case of the left distal tarsal IV (Figure 25). Both *pes* are partially complete, the left missing only digit I and the ungual phalanx of digit III (Figure 26), whereas the right has the ungual phalanx of digit I covered by the right femur, and the distal end of digit IV covered by digit III (Figure 27).

The femora of *M. asymmetrica* and *S. placidus* are rather similar, with the latter slightly smaller and marginally narrower at midshaft, compared to the length of the bone. The shaft is nearly straight in cranial/caudal views, but strongly bowed cranially in lateral/medial views. As best seen in the left element of *S. placidus*, the femoral head expands medially, its long axis forming an angle of about 15° to the distal intercondylar line; in *M. asymmetrica*, this angle is about 30°. The articular surface is smooth and separated from the striated surfaces that surround it by a slightly raised lip (“fhl” in Figure 17). It covers the proximal margin, but also expands distally along the entire medial margin of the head. The proximal outline of the femur is ovoid, with the lateral portion (greater trochanter) more compressed craniocaudally and separated from the main medial portion by a subtle groove on the proximal surface. The caudolateral corner of the head projects slightly caudally, bordering the facies articularis antitrochanterica (“faa” in Figures 12 and 17). On its turn, the craniolateral corner is more rounded in *M. asymmetrica* than in *S. placidus*, in which it forms a subsquared lateral margin. As for the medial margin, the articular facet forms a caudal loop, as it extends medially, surrounding a very deep, caudally positioned, sulcus (“ms” in Figures 12, 16, and 17) for the capitate ligament (= ligamentum capitis femoris). This is clearly seen in the left side of *S. placidus* (Figures 16 and 17), but also (*contra* Naish et al., 2004) in the medial portion of the right femoral head of *M. asymmetrica* (Figure 12). The head has a subsquared medial outline (Figure 17b), with flat proximal, cranial, and distal margins, and a pointed caudal margin that represents the maximal extension of the loop. The caudal surface is mostly excavated, with the distal margin of its medial expansion formed by a subtle ridge (“r” in Figure 17) that extends distally towards the shaft. The cranial surface of the head is divided into a striated proximolateral area and a smoother mediodistal emargination (“mde” in Figure 16). In addition, as best seen in *M. asymmetrica*, the craniolateral corner of the head is distally sloped, extending distally as a column subtly separated from the rest of the head contour (“dlt” in Figures 13, 14, and 16). This corresponds to the “dorsolateral trochanter” of early dinosaurs, which probably served as an insertion point for m. *puboischiofemorales externi* (Hutchinson, 2001) or part of the iliotrocanteric musculature (Langer, 2003).

The flange composed of the lesser and accessory trochanters (Hutchinson, 2001) projects from the craniolateral margin of the proximal fifth of the femur, right distal to the head, and is lateromedially compressed. In *M. asymmetrica*, it is vertically oriented in cranial view and its main axis is craniocaudally oriented in proximal view. Instead, it is more oblique in *S. placidus*, curving slightly more medially and having its proximal portion more laterally positioned, relative to the distal. It has a bilobate shape, with a cranioproximal expansion (lesser trochanter s.s.), which is set apart from the shaft by the intertrochanteric fossa, and a cranially expanded base (accessory trochanter; Hutchinson, 2001). This shape is best seen in *M. asymmetrica*, but also hinted at by its broken margin in *S. placidus*. The lesser and accessory trochanters can be respectively interpreted as the insertion areas of mm. *iliotrochantericus caudalis* and *pubo‐ischiofemoralis internus 2* (Hutchinson, 2001), although a correlation of the lesser trochanter to the m. *iliofememoralis cranialis* has also been proposed (Walker, 1977; Langer, 2003). In *M. asymmetrica*, a rugose area extends caudally from the accessory trochanter, along the lateral surface of the bone, leading to a bulge (“b” in Figures 13 and 14) on its caudolateral corner, the latter of which is also seen in *S. placidus* (Figure 16). This corresponds to the trochanteric shelf of early saurischians and correlates to the insertion of m. *iliofemoralis externus* (= *gluteus medius et minimus*, in McGowan, 1979; Hutchinson, 2001; Carrano & Hutchinson, 2002; Langer, 2003; Brochu, 2003; Naish et al., 2004). The lesser trochanter does not reach the proximal margin of the head in either *M. asymmetrica* or *S. placidus*, but gets significantly closer to that in the latter. The proximal surface of the intertrochanteric fossa of *S. placidus* is pierced by a large nutrient foramen (“fm” in Figure 17), in the same position in which *M. asymmetrica* bears a large pit. Yet, it is not clear if that pit pierced the bone, and several other pits are seen in various positions around the lesser trochanter on both femora of that specimen.

The fourth trochanter forms a low crest on the caudomedial border of the shaft. Its apex is positioned at the middle of the proximal half of the *S. placidus* femur, but somewhat more distally in *M. asymmetrica*. Cranial to the fourth trochanter, on the lateral surface of the femur, a depressed rugose area (“cfli” in Figures 13, 14, and 16) represents the insertion of m. *caudofemoralis longus* (Carrano & Hutchinson, 2002; Langer, 2003). In the right side of *M. asymmetrica*, an ovoid scar is seen at the proximocaudal portion of that area, which could be part of the m. *caudofemoralis longus* insertion or that of m. *puboischiofemoralis internus* (Brochu, 2003). Slightly cranial to that, but in the left side, the femur of *S. placidus* is pierced by a nutrient foramen (“f” in Figure 14), which could not be identified in the right bone, or in *S. placidus*, due to broken surfaces in the area. On the caudal margin of the lateral surface of the shaft, the caudolateral intermuscular line (“clil” in Figures 13, 14, and 16) extends distally from the trochanteric shelf bulge until the fibular condyle, setting the caudal limit of m. *femorotibialis lateralis* (Carrano & Hutchinson, 2002; Langer, 2003). Near the distal end of that line, on the caudal surface of the femur, an oval rugosity (“or” in Figure 16) marks the insertion of muscles of the digital flexor group (Carrano & Hutchinson, 2002; Langer, 2003). Likewise, on the cranial surface of the shaft, the cranial intermuscular line (“cril” in Figures 13, 14, and 16) extends from the accessory trochanter until the craniomedial corner of the distal end, setting apart the *lateralis* and *mediallis* (= *externus* and *internus*) parts of m. *femorotibialis* (Carrano & Hutchinson, 2002; Lacerda et al., 2023). That line leads to a subtle craniomedial distal crest (Hutchinson 2021; = “medial epicondylar flange” of Carrano & Sampson 2008; = “medial‐distal crest” of Hocknull et al., 2009), slightly more marked in *S. placidus* than in *M. asymmetrica* (“cmdc” in Figures 13 and 16). It expands medially, forming a sharp corner that borders a broad depression (“crd” in Figures 13 and 16) on the cranial surface of the bone and a deeper proximodistally elongated depression on its medial surface (“md” in Figure 16). The shaft, broken at its distal third in the right femur of *S. placidus*, reveals an ovoid cross‐section, with the long axis oriented craniomedially to caudolaterally. At this point, the bone wall corresponds to about 10% of the femoral thickness.

The distal articulation of the femur is more complete in *S. placidus* than in *M. asymmetrica*, which preserved only the medial portion of the right bone. Hence, the following accounts are based on the former specimen, with notes on the latter at the end. In distal view, the well‐developed extensor groove (“eg” in Figure 17) is seen as a concavity in the cranial margin of the bone. This is slightly displaced laterally and leads to depressions on both the distal and cranial surfaces of the bone; the former (“dd” in Figure 17) separated from the popliteal fossa by a raised area of the distal articulation. The cranial depression (“crd” in Figures 13 and 17) is rather reduced and separated from the (shallower, broader, and more proximal) depression set laterally to the craniomedial distal crest by artificial perforations on the bone surface. In distal view, the cranial outline of the bone adjacent to the extensor groove is composed of slightly concave, oblique lateral and medial margins. Caudal to that, the medial and, especially, lateral margins of the bone are excavated; the cranial margin of the latter marked by the strong lateral expansion of the fibular condyle. The distal surface of that condyle is separated from that of the lateral condyle by a broad, subtle groove (“gr” in Figure 17). Both the medial and fibular condyles have broadly globular distal surfaces. The lateral condyle and the caudal portion of the medial condyle are subsquared in distal view, subequal and equally projected caudally, although the caudolateral corner of the former forms a sharper angle compared to the caudomedial corner of the latter. The popliteal fossa (= intercondylar fossa; = flexor groove) is deep and also subsquared in shape, excavating about one‐third of the craniocaudal breadth of the distal articulation. In caudal view, the distal femoral margin is concave at the level of the popliteal fossa, which extends proximally excavating the caudal surface of the bone for about 15% of its total length. For most of its length, this fossa is bordered laterally by the sharp, crest‐like proximal extension of the lateral condyle (= crista tibiofibularis; “tfc” in Figure 17). Regarding the preserved part of the distal femoral end of *M. asymmetrica*, it differs from that of *S. placidus* by having a slightly convex, rather than concave medial margin.

The proximal end of the tibia is best preserved in the right side of both *M. asymmetrica* and *S. placidus*. It includes three main projections, the medial and fibular condyles and the cnemial crest, which respectively expand caudomedially, caudolaterally and cranially, giving the proximal outline a subtriangular shape. The proximal surface is broadly flat, but subtly excavated between the caudal condyles, as well as between these and the cnemial crest. The cranial expansion of the cnemial crest is also evident in lateral/medial views. Its cranial tip is distally departed from its proximal end, whereas its distal margin merges smoothly into the shaft. In *S. placidus*, the crest expands proximal to the level of the caudal condyles to form a subtriangular tip (Figure 20a). That expansion is larger and subrectangular in *M. asymmetrica* (Figure 18c,e), with a more rugose proximolateral margin, but the tip is partially broken, hampering a reliable assessment of its shape. In caudal view, the fibular condyle slopes distally in *M. asymmetrica*, but is set at the same proximal level as the medial condyle in *S. placidus*. In proximal view, the cnemial crest is relatively narrow, and curves medially as it expands cranially. Its medial margin is continuous with that of the medial condyle, forming a soft convexity. On the contrary, its lateral margin is strongly excavated by the incisura tibialis (“it” in Figure 20), which also forms the cranial margin of the fibular condyle. The medial condyle is ovoid, bears a marked spine‐like caudal process (“cp” in Figures 18 and 20), and is separated from the fibular condyle by a deep intercondylar notch. Both the condyle and the caudal process are more voluminous relative to the fibular condyle in *M. asymmetrica*, although the condyles are subequal in lateromedial breadth. On the contrary, the fibular condyle is significantly more voluminous and almost twice lateromedially broader than the medial condyle in *S. placidus*. It has a marked subrectangular contour, with flat lateral and caudal margins, whereas this is less clear in *M. asymmetrica*.

The fibular condyle gives rise to a straight, distally extending ridge. This distally leads to the fibular flange (“ff” in Figures 18 and 19), the proximal margin of which is at the level of the distal end of the cnemial crest. As best preserved in the left tibia of *S. placidus* (Figure 19), the flange is straight, rugose, and well‐marked, but not much laterally expanded, extending proximodistally along the estimated proximal third of the bone. That same element reveals that the proximal half of the tibial shaft is nearly straight in cranial/caudal views, but caudally arched in lateral/medial views. This is enhanced by the cranial expansion of the cnemial crest, so that the preserved portion of the tibia has a concave cranial margin in lateral/medial views. Right caudal to the fibular flange, a foramen (“fm” in Figure 20) enters the lateral surface of the tibia in a distomedial direction. As revealed by the distal breakages, the cross section of the tibia is subtriangular near the cnemial crest (*M. asymmetrica*), becoming ovoid (craniocaudally compressed) more distally (*S. placidus*).

Preserved isolated from its proximal portion, the distal half of the right tibia of *S. placidus* has a sub‐rounded cross section at the shaft. Its distal end expands well lateromedially to form the outer and inner malleoli, the former of which is more projected. As such, its proximal margin is less verticalized, but merges as smoothly as that of the inner malleolus into the shaft in cranial/caudal views. The flattened medial margin of the distal tibial end faces slightly caudally and is bound by marked cranio‐ and caudomedial corners. The former expands cranially to form the inner malleolus and extends proximolaterally onto the cranial surface of the tibia, forming a crest (“cr” in Figure 21) that sets the medial limits of the articular facet for the ascending process of the astragalus. That articular facet is subtriangular in cranial view and marked by strong striations. Its lateral edge mirrors the medial in cranial view, but is formed by a more subtle, although sharper ridge (“rd” in Figure 21) that extends laterodistally towards the tip of the outer malleolus. In caudal view, the proximodistally oriented buttress (“cb” in Figures 20 and 21) that forms the caudomedial corner of the tibia also marks the medial edge of a subtle depression (“cd” in Figure 21). This occupies most of the caudal surface of the tibial end, expanding towards the outer malleolus. In cranial/caudal views, the distal margin of the tibia expands slightly at the level of the malleoli, and is subtly concave between them, right lateral to the vertical buttress. The tibial distal outline is subtriangular (Figure 20c), with an ovoid depression at its centre. From a craniocaudally broad medial margin, the outline tapers laterally to reach a caudally curving lateral tip (i.e., outer malleolus).

The proximal portion of the left fibula of *S. placidus* (Figure 22) was not originally described by Kellner (1999). It lacks its proximal margin and tapers distally along its entire preserved length. A broad fossa excavates the medial surface of the bone and also tapers distally. Its cranial margin is thicker than the caudal, so that the shaft is “coma‐shaped” in cross section. The lateral surface is smooth, with no preserved fibular tubercle. Martill et al. (2000) and Naish et al. (2004) described, but not figured, the proximal portion of a right fibula for *M. asymmetrica*. As seen by the first author in 2024, the material catalogued under includes a single piece that could possibly correspond to that element. It is laminar bone twisted along its long axis, probably due to deformation. It does not fit the description of those authors, neither the anatomy of a proximal fibular end (unless highly distorted). So, we refrain from ascribing a fibula to *M. asymmetrica*.

Neither of the tarsal bones of *S. placidus* is fused to the epipodium, to the metatarsi, nor to one another. The astragalus is a complex bone, with a proximodistally flattened body and a very large laminar (craniocaudally compressed) ascending process. As seen in caudal view (Figure 23c), because its cranial surface was still covered in matrix when last analyzed by the first author, the ascending process has a symmetrically triangular shape and is more than twice the maximal height (at its craniolateral portion) of the astragalar body. Its medial margin is confluent with that of the astragalar body, whereas the lateral margin is offset medially from that of the body. The distal surface of the astragalar body is saddle‐shaped, with expanded (both distally and craniocaudally) lateral and medial condyles and a compressed central part. The distal surface is smooth, lateromedially concave and craniocaudally convex. The medial condyle expands slightly more distally than the lateral and is craniocaudally broader. The lateral surface of the astragalus is mostly excavated, with raised distal and cranial borders and a more subtle caudal edge framing the tibial articulation (“ta” in Figure 23). The excavated area is divided by a subtle sub horizontal ridge (“hr” in Figure 23), that extends caudally from the astragalar laterocranial process (“lcp” in Figure 23). Distal to that, the bone surface is more rugose, corresponding to the calcaneal articulation (“ca” in Figure 23). Proximally, the surface is smoother and corresponds to the fibular articulation (“fa” in Figure 23). That surface is continuous with that of the lateral margin of the ascending process, which is craniocaudally broader compared to its main proximal portion. The lateral surface of the astragalus is pierced by foramina, one of them near the base of the ascending process. The medial margin of the astragalar body has an “L‐shaped” outline (Figure 23d), with a proximodistally flattened base and a proximally extensive cranial part that leads to the ascending process. Partially broken on its cranial portion, the medial surface is relatively featureless forming a convex outline in proximal/distal views. The proximal surface of the astragalus is divided into subhorizontal cranial (“crps” in Figure 23), and caudal (“cps” in Figure 23) areas by the transversally extensive ascending process. This is displaced cranially, so that the area caudal to the process is craniocaudally broader compared to that cranial to it. The latter is mostly occupied by a curved (following the concave cranial margin of the bone) continuous depression that gets craniocaudally narrower towards its medial portion. Its lateral margin is formed by a subtle ridge (“rd” in Figure 23) that extends craniolaterally from the lateral edge of the ascending process towards the laterocranial process, setting the craniomedial limit of the fibular articulation. The surface caudal to the ascending process is convex lateromedially and entirely occupied by the tibial articulation. Distal to that, the astragalar body is proximodistally shallow, with a smooth caudal surface. On the contrary, the cranial portion of the body is much deeper, and its cranial surface is marked by longitudinal striations (Figure 23a). Its lateral end expands proximodistally, with the proximal portion of its lateral margin giving rise to the astragalar laterocranial process. In distal view, the caudal limit of that process is marked by a subtle groove (“lg” in Figure 23), expanding medially from the lateral margin of the bone.

The calcaneum is a small bone, strongly flattened lateromedially. Its lateral surface is overall depressed (Figure 24a), with raised outer borders, and pierced by several foramina, especially on its cranial portion. The medial surface is more rugose and equally bears several foramina, some of which are significantly larger. Most of that surface is also depressed, with raised outer borders, but not near its cranial margin, where a scared area (“sa” in Figure 24c) represents the articulation with the laterocranial process of the astragalus. The bone has an irregular shape in lateral/medial views, with a large concave proximal articulation for the fibula and a smaller concave caudodorsal articulation for the outer malleolus of the tibia. Ventral to this the calcaneal contour is formed by three nearly straight, but slightly convex margins: two larger ones facing cranio‐ and caudoventrally, and a smaller one facing cranially. The tibial articulation is bound by a raised lateral margin, which is cranially continuous with the medial margin of the fibular articular facet. The lateral margin of that facet is marked by a fainter corner, which extends caudolaterally from the proximocranial tip of the bone.

Distal tarsals are preserved in both *pedes* of *S. placidus*. They correspond to proximodistally flattened disk‐like bones placed over the proximal surface of the metatarsals (Figure 25b). Distal tarsal III has a slightly convex proximal surface and is trapezoidal in proximal/distal outline, with the caudal margin larger than the cranial. It fits in a central concavity on the proximal surface of metatarsal III, but it also seems to articulate with a lateral excavation of the proximal surface of metatarsal II. Distal tarsal IV has a flatter proximal surface. Its proximal/distal outline is irregular, with a flat medial margin, a caudal concavity, and a lateral pointed projection that is more cranially placed on the right side and more caudally placed on the left side.

To preserve soft tissues, the feet of *S. placidus* were not fully prepared out of the calcareous matrix. The exposed surface of the more distal elements is mostly abraded, especially on the right side, so that most details are unavailable. The gracile and elongated weight‐bearing metatarsals II–IV are closely packed (Figures 25, 26, 27), but not fused to one another. Metatarsal III is the widest and longest, nearly 80% the femoral length, whereas metatarsals II and IV are about 5% shorter and also subequal in width. Only the distal end of metatarsal I is exposed (Figure 27), and it is unclear how much of its laminar body extended along the lateral surface of metatarsal II, but it most certainly did not reach its proximal end. The more robust distal part diverges laterodistally from that metatarsal and bears two well‐developed condyles (the medial slightly larger), with a deep extensor pit set on the dorsal surface between them. The distal margin of metatarsal I barely reaches the distal fifth of metatarsal II.

The proximal end of metatarsal II expands medially, cranially, and especially caudally relative to the shaft (Figure 25e). Its outline is somewhat “D‐shaped” (Figure 25b,c), with a flat lateral margin and a general convex contour including the cranial, lateral, and caudal margins. The cranial portion of the outline is, however, more medially expanded, whereas the caudal part tapers towards its caudolateral corner. The proximal surface is excavated at the center of its lateral half, likely for articulation with the third distal tarsal. The caudal projection of the articular facet extends distally for a short distance as a broad ridge along the caudal surface of the bone. Indeed, it does not reach the distal two‐thirds of the metatarsal, as revealed by the sub‐squared cross section of the shaft at its proximal third. This matches its flat dorsal surface (Figure 26), bound by prominent medio‐ and laterocranial corners, which are more conspicuous proximally. The laterocranial corner extends as a ridge towards the ovoid lateral collateral pit. The distal end of the metatarsal lacks a deep extensor pit, and the medial condyle expands more distally than the lateral.

The proximal end of metatarsal III is slightly shorter craniocaudally than that of metatarsal II, expanding cranially and, especially, caudally relative to the shaft (Figure 25g). Its proximal articular facet has flat cranial, caudal, and medial margins, whereas the lateral is concave for the reception of metatarsal IV. This produces a sub‐rectangular outline, but slightly craniolaterally to caudomedially sloped (somewhat forming a parallelogram). In this configuration, its caudomedial corner meets the caudolateral corner of metatarsal II in the form of a caudal subtriangular projection (Figure 25b). The proximal surface is excavated at its center for the articulation with the third distal tarsal, as revealed by its concave proximal margin in lateral view (Figure 25g). In that view, the proximal portion of the bone is T‐shaped, following the craniocaudal expansion of its proximal end, and bears a subtriangular, distally tapering facet for the articulation of metatarsal IV. Right distal to the expanded proximal end, the caudomedial corner of the shaft forms a subtle flange that expands towards metatarsal II (“fl” in Figure 25f). Although the cross section of its proximal portion is basically circular, the entire dorsal surface of the shaft is flat (Figures 25a and 27). Yet, this is enhanced at its distal half, where the shaft gets gradually broader, forming a subtle medial flange for the contact with metatarsal II at the level of its condyles. Distal to that flange, the metatarsal narrows down, widening lateromedially again to form its distal articulation. The medial flange and the constriction distal to that are reminiscent of the condition seen in tyrannosauroids, ornithomimosaurs, and some other coelurosaurs (Choiniere et al., 2012; Sayão et al., 2020).

The proximal end of metatarsal IV is also “D‐shaped”, with a flat medial margin, which contacts metatarsal III, and a convex lateral border. As in metatarsal III, the caudomedial corner of the proximalmost portion of the shaft forms a subtle flange that expands towards that same element. Distal to that, but still in the proximal third of the shaft, a breakage reveals a subsquare cross section. The distal portion of the bone curves slightly laterally (Figure 25a), but is very damaged on both sides, revealing nothing but the expansion of its distal end.

The phalangeal formula of *S. placidus* is 2‐3‐4‐5 (Figures 26 and 27). Digit II is almost as long as digit IV, whereas digit III is about 40% longer than those. The left foot is significantly best preserved and forms the basis of this account for overlapping elements. Despite the preservation of soft tissue in *S. placidus* (Hendrickx et al., 2022), there is no evidence of corneous tissue in the ungual phalanges. Phalanx I‐1 is the smallest (shortest and thinnest) of the foot (Figure 27). It was not weight‐bearing, projecting laterodistally as a “spur” from the packed metatarsus. It tapers distally until it expands significantly to form the distal condyles, the exposed of which (likely the medial) bears a large (considering the phalanx size) and deep collateral pit. Phalanx II‐1 is more than three times longer than wide at its proximal end, with a subtriangular shaft cross‐section. The medial condyle is more developed than the lateral and the lateral collateral pit is deep and ovoid shaped. Phalanx II‐2 and II‐3 are exposed on their lateral sides. The former is about two thirds the length of the preceding phalanx and three times longer than the dorsoventral depth of its distal end. Its proximal end is not well‐preserved, but it expands dorsal to the level of the shaft, suggesting the presence of an extensor tubercle. In the distal end, the lateral condyle bears a well‐developed and proximodistally elongated collateral pit. Because the condyle is ventrally expanded, the pit is positioned towards its dorsal portion. The ventral length of phalanx II‐3 is nearly two‐thirds of that of phalanx II‐2. In lateral view, its dorsal margin is convex, the contrary being the case for the proximal and ventral margins. Indeed, this is the most strongly recurved ungual of the pes, with a tapering distal tip. Small longitudinal striations cover the lateral surface near the proximal articulation and a nutrient foramen is seen distal to these striations, towards the dorsal part of the phalanx. The extensor tuber expands proximally as a curved tapering process, corresponding to about one‐fifth of the total length of the phalanx. A single lateral groove extends along the distal half of the bone, somewhat following its curved contour. It enters the dorsal surface of the phalanx as it extends distally, but does not reach its distal end.

Phalanx III‐1 is the largest of the foot, slightly surpassing the length of phalanx II‐1, but is well‐preserved only at midshaft. Its general morphology is conservative, with a mediolaterally constricted mid‐shaft and equally enlarged proximal and distal ends. The lateral collateral pit seems well‐developed and large as in the previously described phalanges. Phalanx III‐2 is better preserved than the previous one and about four‐fifths of its total length. It is constricted at the mid‐shaft, with a relatively flat dorsal surface, and a distal end not as lateromedially expanded as the proximal. The extensor pit is broad, subtriangular, and distally deeper, whereas the lateral collateral pit is large and proximodistally elongated. Phalanx III‐3 lacks its distal end on the left side and most of its surface on the right. It is almost as long as phalanx III‐2, but more gracile, that is, narrower both at mid‐shaft and at the distal end. The ungula phalanx of digit III is shorter and less curved than that of digit II, with the lateral groove more ventrally positioned at its distal end. The phalanges of digit IV are reduced compared to those of the other digits. Phalanx IV‐1 is the longest, with the others shortening to about three‐quarters to two‐thirds of the length of the previous one. The ungual is, however, longer than the two preceding elements. The extensor tubercle and flexor platform are well‐developed in IV‐2, IV‐3, and IV‐4, and the preserved lateral collateral pits are relatively smaller compared to those of more medial digits. The ungual phalanx is less recurved and slightly shorter than that of digit II, with the lateral groove also ending more dorsally, unlike that of digit III.

#### 3.1.4 Soft tissues

Both *S. placidus* and *M. asymmetrica* exceptionally preserve soft tissues. Kellner (1996) briefly described those of *S. placidus*, mentioning the preservation of skin, muscle fibers, and possibly blood vessels. The latter two were identified under scanning electron microscopy and our naked‐eye revaluation of the specimen obviously provided no further detail about these elements. More recently, Hendrickx et al. (2022; appendix S3.5) provided a comprehensive treatment of the scaly skin of *S. placidus* (i.e., squamous integument in the metatarsals, crus, and pes, more precisely lateral to metatarsal IV, and lateral/ventrolateral to pedal digits III and IV), a summary of which is given here. Two types of scales are preserved in negative relief form the squamous integument. Relatively large (~0.3–0.6 mm maximum dimension) hexagonal scales visible lateral to the proximal portion of metatarsal IV represent the first type. They are larger and more elliptical than the rest of the scales and oriented parallel to the metatarsus. Owing to their position lateral to the metatarsus, large size, hexagonal shape, and the fact that they increase in size towards the metatarsal, they were interpreted by Hendrickx et al. (2022) as scutellate scales. Minute diamond‐shaped scales (<0.1 mm) ventral to the distal portion of metatarsal IV and pedal digits III and IV and forming quadrangular pits between the raised interstitial tissue form the second integumentary type. Their presence in the ventral part of the foot makes them reticulate scales, which form a regular network, the external margin of which ventral to the pedal digit defines plantar pads. A single plantar pad is visible in line with the joint between the first and second phalanx of digit III. Conversely, four points invaginate the straight external margin of the integument, approximately at the mid‐shaft of each phalanx. Although the associated soft tissues on the digits do not necessarily reflect the shape and arrangement of the plantar pads, the position of the invaginations ventral to pedal digits III and IV supports an arthral condition. The association of scutellate and reticulate scales seen in *S. placidus* forms a podotheca which, in the carcharodontosaurid *Concavenator corcovatus*, also includes large polygonal scutate scales lateral to the proximal portion of the metatarsals (Cuesta et al., 2018; Hendrickx et al., 2022). These scutate scales are, however, absent in *S. placidus*, possibly due to the fact of not having the proximalmost portions of the metatarsals preserved. The distal portions of the *S. placidus* digits resemble those of early Avialae, which have minute reticulate scales covering the plantar surfaces (O'Connor, 2025; O'Connor et al., 2025), as well as plantarly expanded joint pads adapted for terrestrial locomotion (O'Connor et al., 2025).

**FIGURE 28:**
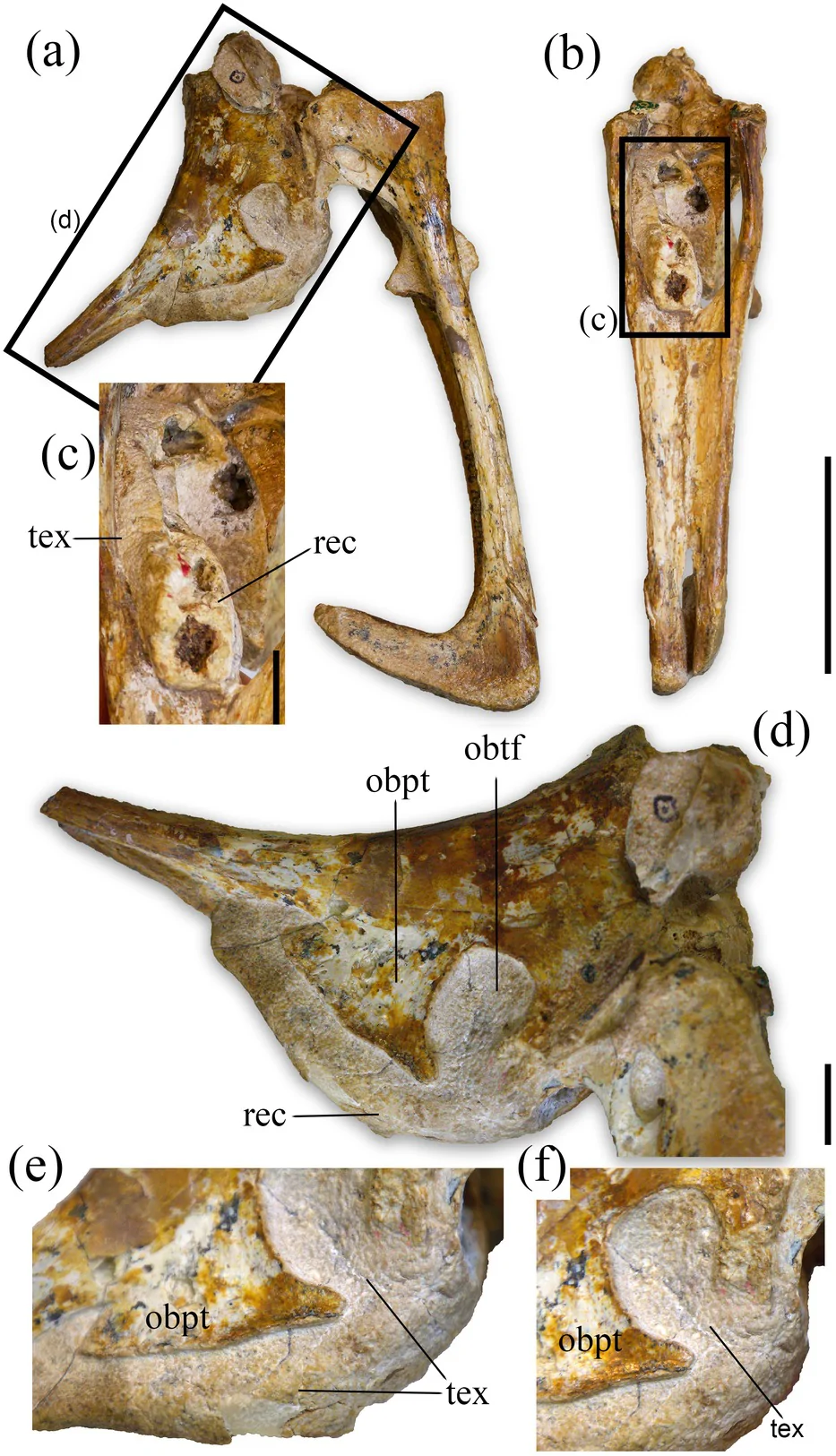
*Mirischia asymmetrica* (SMNK 2349 PAL). Soft tissue associated with the pelvis in (a) right lateral and (b) cranial views; with a close‐up on (c) a cross section of the rectum situated between the proximal branches of the left and right pubes in cranial view; (d) the rectum loop ventral to the right ischium in right lateral view; and (e) the globular surface texture of the rectum ventral to the obturator flange, and (f) within the obturator notch of the ischium in lateroventral views. obtf, obturator foramen; obtp, obturator plate; rec, rectum; tex, surface texture of the rectum. Scale bars equal 5 cm (a, b) and 1 cm (c, d); e and f without scale.

Soft tissue in *M. asymmetrica* is associated with the pelvic girdle (Figure 28) and its nature is more difficult to interpret. Martill et al. (2000, p. 898) described “a conical structure […] in the matrix behind the pubis, between the ventral surface of the sacrum and the distal end of the pubis”. A thin section made by these authors revealed that the internal texture of this structure was spongy, with “small, irregular, calcite‐lined vacuities”, whereas the outer layer was smooth and dense. Martill et al. (2000) identified this structure tapering towards the pubis and extending caudodorsally as a lithified portion of the intestine. They provided a detailed description of the route of the presumed intestinal tract: it enters cranioventrally inside the opening made by the pubic apron and the sacrum in a straight line, turns to the right to pass medially to the right pubic shaft, then turns caudodorsally to extend medial to the caudal margin of the pubic shaft and the ventral margin of the proximal portion of the pubis. It then loops ventromedially to reach the ventral margins of the ischia, where it apparently continues horizontally between the ischia. Based on its location, internal structure, and the route it follows, the interpretation of this structure as an intestinal tract is completely pertinent. As remarked by Martill et al. (2000), the route they identified indeed very closely matches that followed by the intestine of the coelurosaur *Scipionyx samniticus*, the only non‐avian theropod for which the intestinal tube is (exceptionally) preserved. Based on a comprehensive description of this organ given by Dal Sasso and Maganuco (2011), the portion of the intestinal tract ventral to the ilium and ischium can further be identified as the rectum. Likewise, the stony protuberance attached to the ventral portion of the ischium, identified by Martill et al. (2000) as a loop taken by the intestine, logically corresponds to the wide loop made by the caudalmost portion of the rectum before terminating as a straight tract (Dal Sasso & Maganuco, 2011, figure 150).

We, however, observe some differences in the position and external surface of the rectum of *M. asymmetrica* and *Sc. samniticus*. In the latter, the wide loop made by the distalmost portion of the rectum is preserved caudal to the ischium, whereas the supposed intestinal loop in *M. asymmetrica* occurs ventral to the ischium. Such a difference can, however, be explained by the “anomalous caudodorsal displacement of the rectum” noticed by Dal Sasso and Maganuco (2011, p. 151) for *Sc. samniticus*. We additionally note that the rectum of *M. asymmetrica* ventral to the ischium is not clearly delimited, but this might be due to the large dimension of this organ. The width of the rectum at the level of the ischium is indeed particularly large in *Sc. samniticus*, occupying approximately the proximal half of the bone, whereas the loop formed by the rectum at the level of the ischium extends over two‐thirds of the proximodistal length of this pelvic bone. Finally, the external surface texture of the intestinal loop on the lateral side of the right pelvic girdle of *M. asymmetrica* is globular and reminiscent of epidermal scales (Figure 28), whereas the best‐preserved surface of the rectum of *Sc. samniticus* is smooth and does not show a similar bumpy appearance. In *M. asymmetrica*, the globular texture visible on the concretion attached to the ventral surface of the ischium is made of irregularly shaped rounded bumps, changing gradually in size along its craniocaudal length (Figure 28). The surface is made of minute (0.45–0.95) mainly subcircular bumps ventral to the obturator flange and cranial to the ischial shaft (Figure 28). It conversely shows larger (0.8–1.9 mm) irregularly shaped rounded bumps within the obturator notch of the right ischium (Figure 28). Based on the regular distribution of the bumps and the gradual change in size along the concretion, the globular texture was initially thought to have resulted from the imprints of the overlying epidermal scales from the skin covering this portion of the pelvis. The bumpy texture is, however, visible on the ventralmost portion of the loop made by the rectum as well as its portion extending in a straight line between the pubic apron and the sacrum, rejecting the hypothesis that they represent the imprint of epidermal scales. Because *Sc. samniticus* corresponds to a hatchling individual whereas *M. asymmetrica* died as a much older and larger individual, although interpreted by Martill et al. (2000) as still immature, this suggests that the external surface texture of the rectum varied between theropods or possibly during growth.

Consequently, based on the route, position, and dimension of the rectum of *Sc. samniticus*, we concur with Martill et al. (2000) that the structure associated with the pelvic girdle of *M. asymmetrica* most probably corresponds to the fossilized intestinal tube, here identified as the rectum. We further postulate that the globular texture visible on different portions of the rectum genuinely represents the surface texture of this exceptionally preserved lithified portion of the intestine.

### 3.2 Phylogenetic results

**FIGURE 29:**
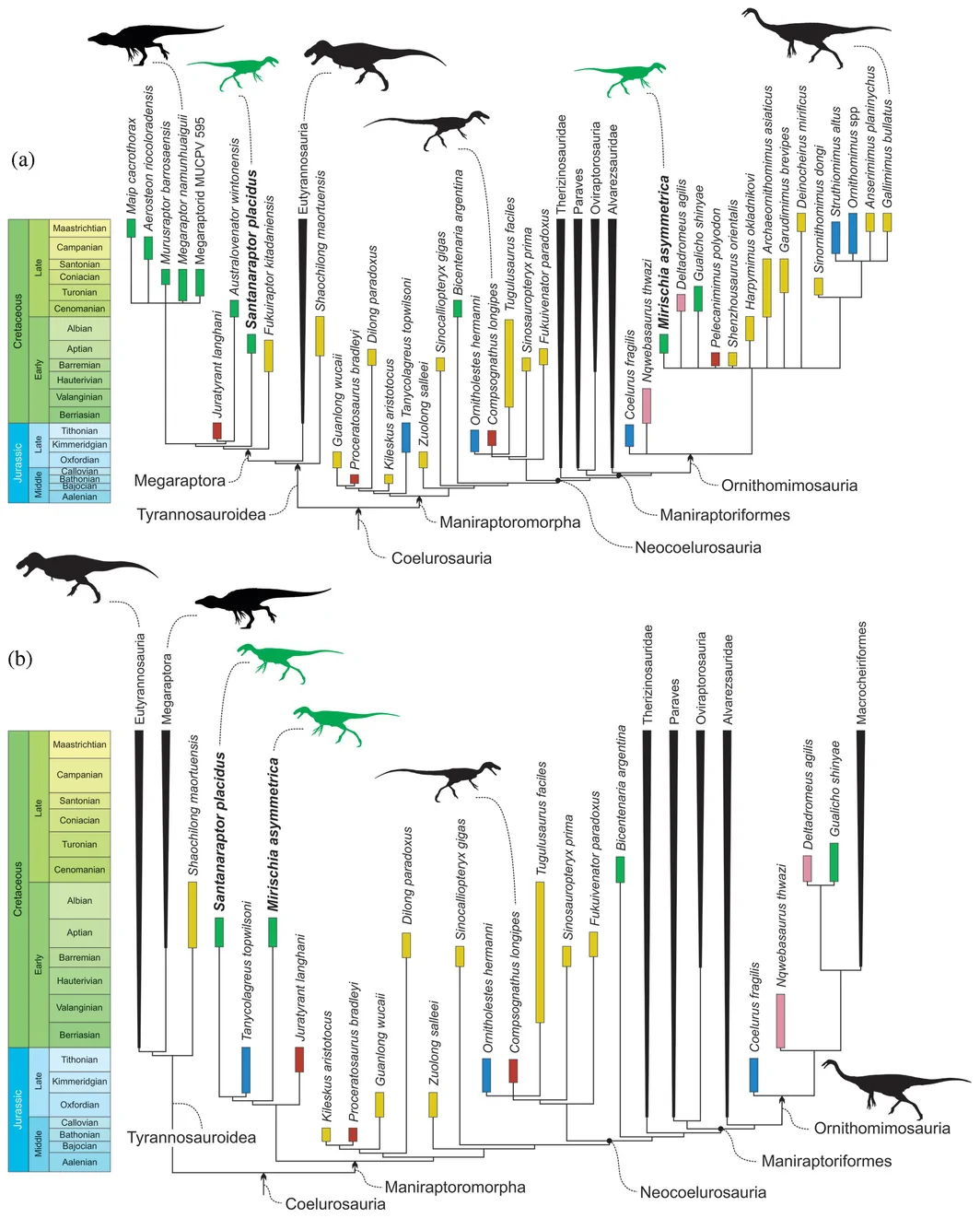
Phylogenetic hypotheses showing the position of *Santanaraptor placidus* and *Mirischia asymmetrica* (green outlines) as (a) early branching Megaraptora and *Ornithomimosauria*, respectively; and (b) early branching *Maniraptoromorpha*. Arrows indicate branch‐based clades and circles indicate node‐based clades, based on Table 1. Colored bars indicate the temporal range and geographical area. Yellow, Asia; green, South America; blue, North America; red, Europe; pink, Africa. Abridged version of the consensus of MPT found via a “New Technology Searches” in TNT.

Both equal and implied weight analyses resulted in broadly similar trees, with the main coelurosaur clades remaining largely consistent in their inclusivity and relations to one another. Main differences between the analyses include the number of trees found and the resulting position of some taxa at the base of *Coelurosauria* (including *S. placidus* and *M. asymmetrica*). The analysis using equal weights resulted in 100.000 trees (overflow) with a score of 8.403, whereas the analysis using the implied weight of *k* = 16 produced only nine trees with a score of 286.2031. Both analyses placed *S. placidus* and *M. asymmetrica* within *Coelurosauria*, but in differing positions. Under equal weights, *S. placidus* is placed as an early branching *Megaraptora*, with that clade nested within *Tyrannosauroidea*, whereas *M. asymmetrica* is placed in a large polytomic clade of *Ornithomimosauria* (Figure 29a). With implied weight, both were found at the earliest‐branching clade of *Maniraptoromorpha*, together with *Tanycolagreus topwilsoni* and *Juratyrant langhani* (Figure 29b). The unstable positions of *S. placidus* and *M. asymmetrica* likely result from their high amount of missing data (85.1% and 86.3% respectively) and the influence of homoplasy in the early radiation of *Coelurosauria*. Among the other interesting results of our analyses is the position of *Coelurus fragilis* and the enigmatic Gondwanan theropods *Deltadromeus agilis* and *Gu. shinyae*, within the *Ornithomimosauria*. Although an in‐depth examination of ornithomimosaur phylogeny is beyond the scope of this study and we do not expect to definitively settle this issue, the position of these taxa does deserve further discussion.

## 4 DISCUSSION

### 4.1 Alpha‐taxonomy of *S. placidus* and *M. asymmetrica*

Are *S. placidus* and *M. asymmetrica* unique taxa? Is the latter a junior synonym of the former? The latter question is relatively easy to evaluate and refute. Starting with their provenances, Kellner (1999) stated that the concretion preserving the holotype of *S. placidus* probably came from around the town of Santana do Cariri, Ceará, whereas Martill et al. (2000) suggested that the nodule preserving the holotype of *M. asymmetrica* came from the region of Araripina, Pernambuco, but that other source areas cannot be ruled out. Indeed, as almost all Romualdo Formation fossils that came to scientific knowledge were excavated by private fossil collectors of the Araripe Plateau, their ultimate provenances remain elusive. Hence, this is not useful to set them apart, but the two holotypes do bear several anatomical differences.

Among the skeletal parts that overlap between *S. placidus* and *M. asymmetrica*, that is, ischium, femur, and tibia, the former bone arguably bears more differences. Indeed, the iliac peduncle of *S. placidus* has a rugose dorsal protuberance for muscle attachment and is somewhat cranially bent, supporting a more verticalized iliac articular facet, whereas that of *M. asymmetrica* has a smoothly concave dorsal surface, supporting a more dorsally facing iliac articulation. The pubic peduncle of *S. placidus* is proximodistally narrower and tab‐shaped, whereas that of *M. asymmetrica* is hatchet‐shaped, leading to a more extensive pubic articulation. Its ventral extension transitions towards the obturator plate, which forms the cranial margin of the obturator foramen, fully enclosing that aperture. Instead, the obturator plate of *S. placidus* has a completely different configuration, forming a subtriangular projection that is equivalent only to the portion of the plate that, in *M. asymmetrica*, is positioned distal to the foramen. Indeed, that aperture is equivalent, in *S. placidus*, to a broad, cranioventrally open obturator notch. Such differences in the obturator plate were already used by Naish et al. (2004) to reject the synonymy between the two taxa. Another difference is that the distal margin of the plate merges smoothly onto the shaft in *S. placidus*, but more abruptly in *M. asymmetrica*. The femora of the two taxa are very similar, with only marginal differences, for example, that of *M. asymmetrica* is slightly more robust, has a less inturned head, a more distally positioned (as is also the case of the fourth trochanter) and proximodistally oriented lesser trochanter, and a distal end that bears a less marked “medial epicondylar flange” and a slightly convex (rather than concave) medial margin. As for the tibia, the cnemial crest seems to be more expanded in *M. asymmetrica*, but major differences relative to *S. placidus* are concentrated in the caudal portion of the proximal articulation, that is, the fibular condyle is distally displaced, has a more rounded contour, and is much less voluminous relative to the medial condyle. As such, we consider that there is compelling evidence for the non‐synonymy between the two Romualdo taxa. Of note, Naish et al. (2004) also differentiated *M. asymmetrica* from *S. placidus* based on the absence of a large groove in the femur, which likely refers to the capitate ligament sulcus of the femoral head. Yet, although partially hidden by sedimentary matrix and the acetabulum, the proximal portion of that sulcus is exposed in the proximal view of both *M. asymmetrica* femora (Figure 12), supporting the fact that it is as well developed as that of *S. placidus*.

**FIGURE 30:**
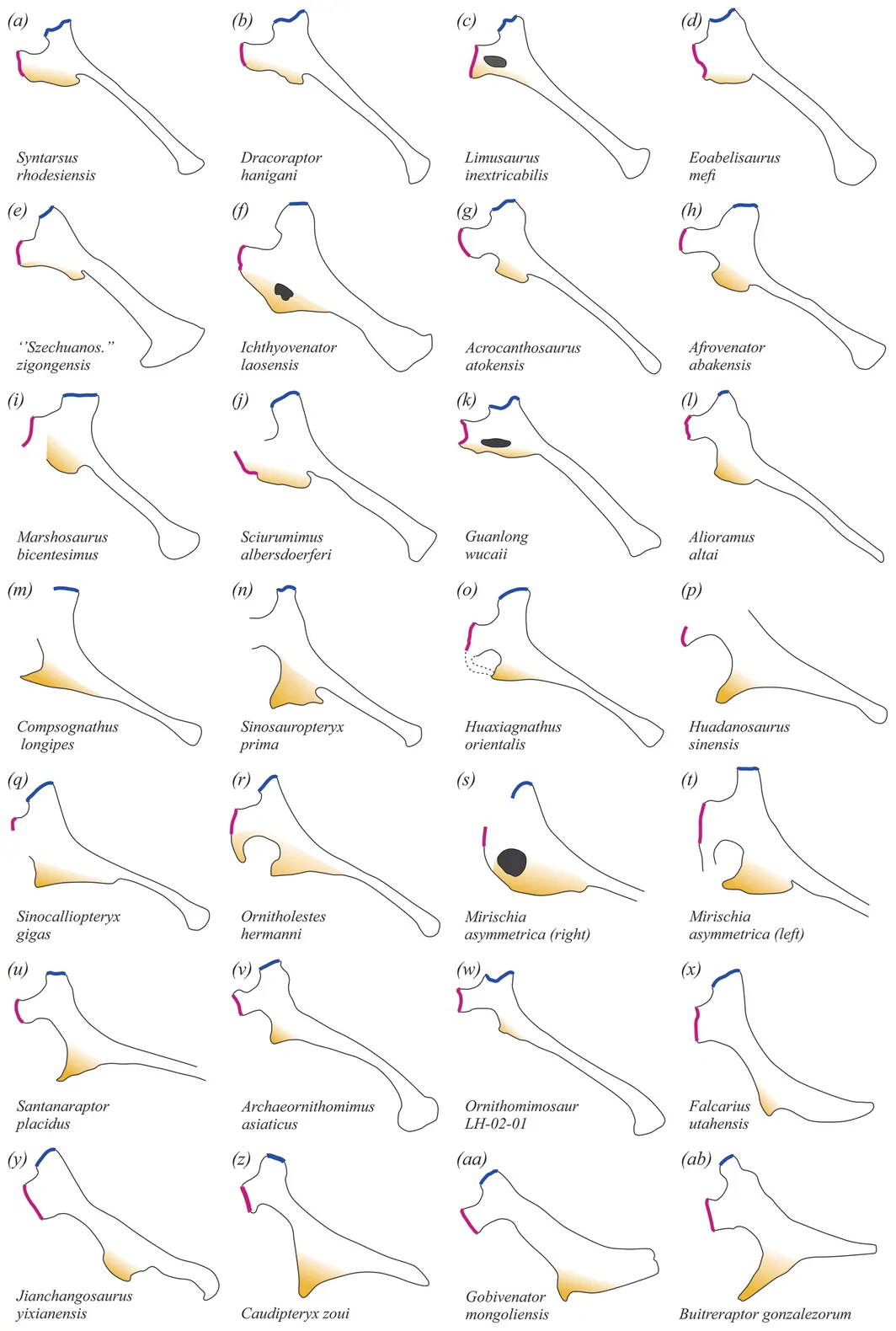
Ischia of assorted theropods in lateral view (not at the same scale and reversed from right to left if needed). (a) from Raath (1977); (b) from Martill et al. (2016); (c) from Xu et al. (2009); (d) MPEF‐PV 3990; (e) from Gao (1993); (f) from Allain et al. (2012); (g) NCSM‐14345; (h) UC OBA‐1; (i) from Madsen (1976); (j) from Rauhut et al. (2012); (k) from Xu et al. (2006); (l) from Brusatte et al. (2012); (m) B.S.P. A.S. I 563; (n) from Currie and Chen (2001); (o) from Hwang et al. (2004); (p) from Qiu et al. (2025); (q) from Xing et al. (2012); (r) AMNH FARB 619; (s) SMNK 2349 PAL; (t) SMNK 2349 PAL (personal archive); (u) MN 4802‐V; (v) from Smith and Galton (1990); (w) from Yao et al. (2022); (x) from Rhodes et al. (2021); (y) from Pu et al. (2013); (z) IVPP‐V 12344; (aa) from Tsuihiji et al. (2014); (ab) from Gianechini et al. (2018); Blue line = iliac articulation; red line = pubic articulation; yellow shadowing = obturator plate.

The important differences displayed by the ischial obturator plates of *M. asymmetrica* from *S. placidus* deserve some discussion. Among non‐averostran neotheropods (Figure 30), the plate is plesiomorphically placed proximally, continuous with the pubic peduncle (Griffin, 2018; Langer et al., 2017; Marsh & Rowe, 2020; Martill et al., 2016; Martínez & Apaldetti, 2017; Rauhut, 2003; Spiekman et al., 2021), and sometimes perforated by a foramen (Carrano et al., 2005), a condition typically retained by ceratosaurs (Coria et al., 2006; de Souza et al., 2021; Gilmore, 1920; Pol & Rauhut, 2012; Rauhut & Carrano, 2016; Xu et al., 2009). Conversely, the obturator process of maniraptorans is much more distally placed along the ischium (Rauhut, 2003), as seen in therizinosaurs (Liao et al., 2021; Zanno, 2010; Zanno et al., 2009), oviraptorosaurs (Barsbold et al., 2000; Funston et al., 2020; Lü et al., 2013), troodontids (Rhodes et al., 2021; Russell & Dong, 1993), and dromaeosaurs (Norell & Makovicky, 1997; Novas et al., 2021; Ostrom, 1969; Rhodes et al., 2021). In these taxa, the process is never perforated by a large opening and instead forms the distal margin of a broad obturator notch, which is arguably equivalent to the obturator foramen. The obturator process of maniraptorans would be, therefore, not equivalent to the whole obturator plate of early theropods, but only to its distal part. In fact, *M. asymmetrica* provides evidence for this homology, as its left ischium has a “notch” equivalent to the foramen on its right side and a “process” similar to that of maniraptorans and some other coelurosaurs, including *S. placidus*. We interpret the left obturator plate of *M. asymmetrica* as incomplete and note that its “hook‐shaped” proximal and “spur‐like” distal parts are reminiscent of the condition seen in some other non‐maniraptoran coelurosaurs such as *Ornitholestes hermanni* and *Huaxiagnathus orientalis* (Hwang et al., 2004; Carpenter, 2005), possibly representing a stereotyped breakage pattern of the bone. Indeed, non‐maniraptoran tetanurans display some variation in the position of the ischial obturator plate/process, with some exhibiting a plate plesiomorphically closer to the pubic peduncle, whereas others have a distally displaced process (although normally not as much as that of maniraptorans). Among coelurosaurs, *S. placidus* clearly fits onto the latter pattern, which also includes most ornithomimosaurs (Kobayashi & Lü, 2003; Macdonald & Currie, 2019; Makovicky et al., 2004; McFeeters et al., 2018; Smith & Galton, 1990; Yao et al., 2022) and tyrannosaurids (Brochu, 2003; Brusatte et al., 2010; Sereno et al., 2009), as well as taxa typically classified among Compsognathidae (Currie & Chen, 2001; Ostrom, 1978; Qiu et al., 2025; Xing et al., 2012). Some non‐coelurosaur tetanurans also have a distally displaced process (Madsen Jr, 1976; Currie & Zhao, 1993; Sereno et al., 1994; Brusatte et al., 2008; Malafaia et al., 2020; Canale et al., 2015, 2022), although these are usually not “spur‐like” as in the above‐mentioned coelurosaurs. On the contrary, some tetanurans (Allain et al., 2012; Charig & Milner, 1997; Gao, 1993; Rauhut et al., 2012) retain the plesiomorphic condition of a proximally placed plate continuous with the pubic peduncle, at times perforated by a foramen. This condition approaches that inferred for *M. asymmetrica*, suggesting that it is indeed plesiomorphic relative to that of *S. placidus*. Finally, the presence of the plesiomorphic condition in members of major coelurosaur clades (Ji et al., 2003; Xu et al., 2006) that otherwise typically share the apomorphic condition, suggests a considerable degree of homoplasy in the shape and position of the ischial obturator plate/process in the early radiation of *Coelurosauria*.

As for the taxonomic uniqueness of *S. placidus* and *M. asymmetrica* relative to other taxa, they are the only non‐spinosaur theropods named from the upper carbonate concretion levels of the Romualdo Formation, and we consider that such provenance would be, based on topotypical principles, enough to dismiss the possibility that either of them is synonymous with any other theropod named from elsewhere in the world (both geographically and chronologico‐stratigraphically). In any case, their rescoring into the MTWG taxon‐character matrix revealed that both have a unique combination of features in relation to other theropods of the rather comprehensive sample of that dataset (see Data S1). Hence, for now, we see no evidence for synonymy of either *S. placidus* or *M. asymmetrica* with other taxa.

### 4.2 Taxonomic affinities of *S. placidus* and *M. asymmetrica*

Under equal weighting, our phylogenetic analysis places *S. placidus* and *M. asymmetrica* among distantly related coelurosaur clades, respectively *Megaraptora* and *Ornithomimosauria* (Figure 29a). Only three characters pull *S. placidus* into *Megaraptora*: (1) neural spines on distal caudal vertebrae forming low ridges (char. 615); (2) femur with caudolateral bulge for m. *iliofemoralis externus* attachment (char. 849); (3) phalanx IV‐2 with neck longer than distal ginglymus (char. 955). Among the megaraptors included in our dataset, the first condition is only shared with *Fukuiraptor kitadaniensis* and the latter two only with *Australovenator wintonensis*. We note that these characters are widespread among *Maniraptoriformes*, as exemplified by *Albertosaurus sarcophagus*, *Saurornithoides mongoliensis*, *Mei long*, and *Sinornithosaurus millenii*. Hence, although *S. placidus* was placed within *Tyrannosauroidea* by Delcourt and Grillo (2018), and even though our results (see also Porfiri et al., 2018; Rolando et al., 2019, 2022) place *Megaraptora* as the sister‐clade of *Tyrannosauroidea*, we do not find any compelling evidence for the nesting of *S. placidus* within *Megaraptora*.

As for the nesting of *M. asymmetrica* within *Ornithomimosauria* (Figure 29a), this is likewise supported by a reduced sample of homoplastic characters spread among theropods in general (e.g., *Alioramus altai*, *Masiakasaurus knopfleri*, *Buitreraptor gonzalezorum*), namely: (1) elongated last trunk vertebrae (char. 550); (2) trunk postzygapophyses abutting above the neural canal, with the opposing hyposphenes meeting medially to form a lamina (char. 566); (3) pubic boot projecting mostly caudally, with little or no cranial process (char. 811). Among the ornithomimosaurs included in this analysis, the last condition is only shared with *Deinocheirus mirificus*, with which *M. asymmetrica* also shares an enclosed obturator foramen (Lee et al., 2014), but the pubic boot is clearly less cranially expanded in the Brazilian taxon. We also note that *M. asymmetrica* lacks a crest in the caudomedial surface of the femur for the attachment for m. *adductor femoris 1* (Carrano & Hutchinson, 2002; char. 860), which is a condition observed in *D. agilis* and core ornithomimosaurs such as *Str. altus* and *Gal. bullatus*. Indeed, our analysis nested not only *D. agilis*, but also *Gu. shinyae* among ornithomimosaurs, as similarly obtained by Kellermann and Rauhut (2022).

**FIGURE 31:**
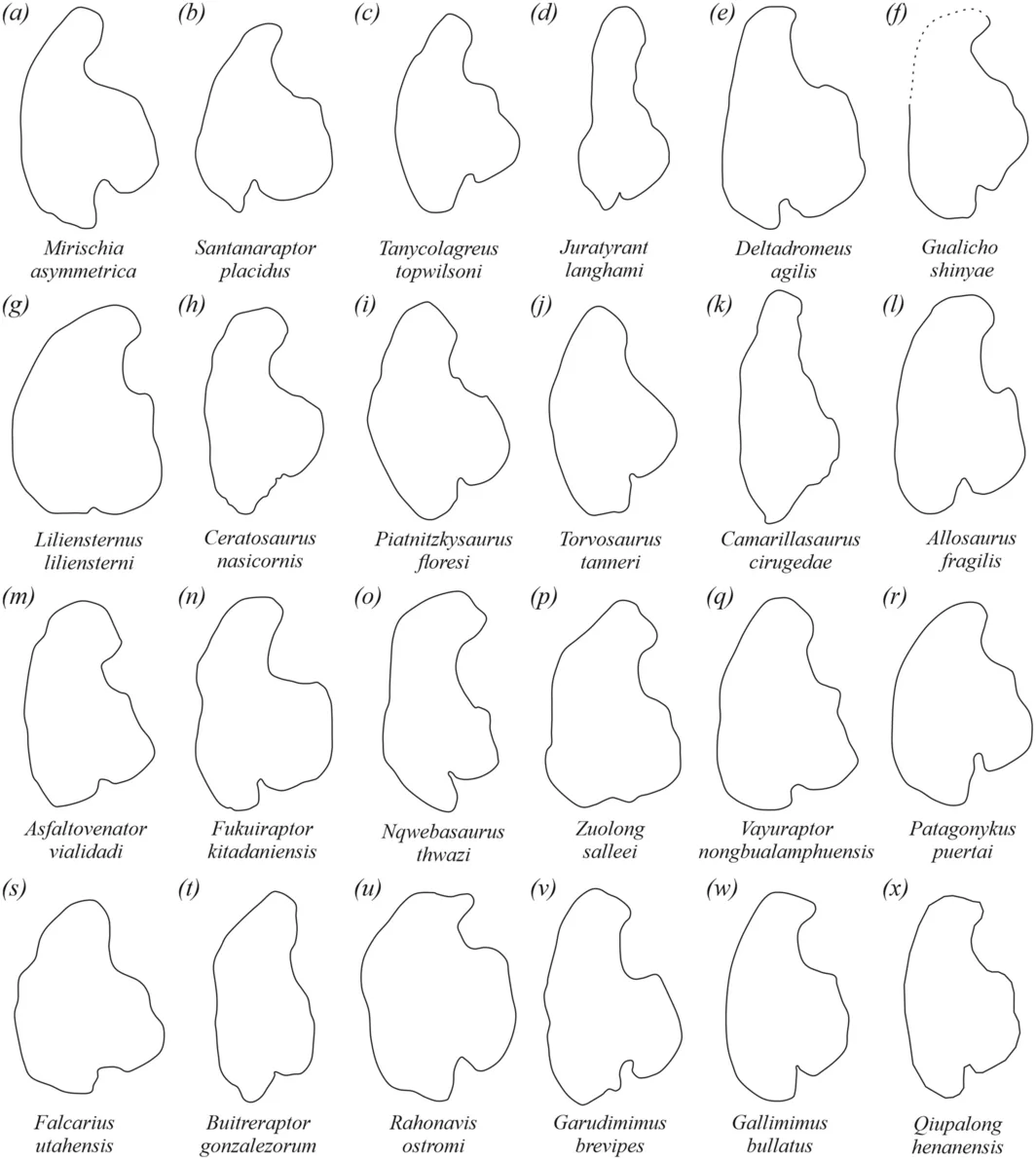
Tibiae of various non‐avian theropods in proximal view (not to scale and reversed from left to right if needed). (a) SMNK 2349; (b) MN 4802‐V; (c) from Carpenter et al. (2005); (d) from Benson (2008); (e) SGM‐Din 2; (f) from Apesteguía et al. (2016); (g) HMN MB.R. 2175; (h) from Samathi et al. (2021); (i) from Pradelli et al. (2025); (j) BYU 2002; (k) from Samathi et al. (2021); (l) UMNH VP 7928; (m) MPEF PV 3440; (n) FPDM‐V9712220; (o) AM 6040; (p) from Choiniere et al. (2010); (q) from Samathi et al. (2019); (r) from Novas (1997); (s) from Zanno (2010); (t) MPCA 245; (u) UA 8656; (v) from Kobayashi and Barsbold (2005); (w) from Osmόlska et al. (1972); (x) from Xu et al. (2011).

Our implied weighted (IW) *K = 16* analysis produced significantly different results (Figure 29b). Most importantly, we found *M. asymmetrica* and *S. placidus* nested into a clade of early diverging maniraptoromorphs, along with *Tanycolagreus topwilsoni* from the Morrison Formation (Carpenter et al., 2005) and *Juratyrant langhami* from the Kimmeridge Clay (Brusatte and Benson, 2013). *Juratyrant langhami* is the first‐branching member of that clade, whereas *S. placidus* and *T. topwilsoni* are sister‐taxa. The clade is supported by five synapomorphies: craniocaudally long and narrow pubic peduncle of the ilium (char. 783); lateromedially narrow flange below the ischial peduncle of the pubis (char. 796); enclosed pubic obturator foramen (char. 797); small bulge in the proximocaudal portion of the ischium (char. 821); arcuate and caudally angular medial condyle of the tibia (char. 883). This last character deserves some attention. In proximal view, the caudal margin of the medial condyle of the tibia is typically rounded among theropods (Figure 31). Its lateral side in *S. placidus* and *M. asymmetrica* is, however, marked by a subtriangular process that expands caudally and slightly laterally, forming a sharp caudolateral corner for the condyle. This process is very distinctive in *S. placidus*, but larger and more continuous to the medial margin of the condyle in *M. asymmetrica*. As for the other members of the clade, the medial condyle of *J. langhami* bears a caudally expanding process, as seen in few other theropods (Benson, 2008; Samathi et al., 2021), whereas that of *T. topwilsoni* has a somewhat angular caudomedial corner, as more commonly seen in the group (e.g., Azuma & Currie, 2000; Forster et al., 2020; Hocknull et al., 2009; Novas, 1997; Zanno, 2010). Similar constructions are also seen in some member of the ornithomimosaur lineage. In *N. thwazi*, the process is akin to that of *M. asymmetrica*, but slightly larger and with less defined boundaries. As such, this could be interpreted simply as a caudolateral extension of the condyle (see Brusatte et al., 2008; Novas et al., 2008; Zanno et al., 2009), but with a sharp caudolateral corner, as seen in the Romualdo forms. In *D. agilis*, the process is almost identical to that of *M. asymmetrica*, although the lateral condyle of its tibia is enlarged as that of *S. placidus*. The condition in *Gu. shinyae* is more disparate. The caudolateral corner of its medial condyle is clearly angular, but the process itself is not as prominent as in the other taxa. In unequivocal ornithomimosaurs (Allain et al., 2014; Buffetaut et al., 2009; McFeeters et al., 2016; Xu et al., 2011), the medial condyle typically exhibits a rounded, sometimes sub‐quadrangular outline, as commonly seen in other theropods. Yet, those of *Gal. bullatus* (Osmόlska et al., 1972) and *Sinornithomimus dongi* (Kobayashi & Lü, 2003), as well as that of the holotype of *Coelosaurus antiquus* (Sullivan, 1997), expands caudomedially to form an acute corner, similar to that of *N. thwazi*. In addition, the medial condyle of *Garudimimus brevipes* bears a small lateral tuber (Kobayashi & Barsbold, 2005) resembling the process discussed here.

The investigation of the early radiation of coelurosaurs is in a state of flux, with the proposal of different arrangements for the relation of its major groups and individual taxa of uncertain affinities (e.g., Brusatte et al., 2014; Cau, 2024; Novas et al., 2013). This is exemplified by the two species dealt with here, which were already considered to represent compsognathids (Brusatte et al., 2014; Foth et al., 2025; Naish et al., 2004; Qiu et al., 2025), tyrannosauroids (Delcourt & Grillo, 2018; Naish & Cau, 2022), and even noasaurid ceratosaurs (Brownstein, 2021). Our suggestion that, along with two Laurasian theropods, *M. asymmetrica* and *S. placidus* instead form the earliest branch of *Maniraptoromorpha* has implications for our understanding of the radiation of coelurosaurs as a whole, and other major groups in which the Brazilian taxa were previously included. Indeed, the exclusion of *M. asymmetrica* from Compsognathidae pushes the last record of the group by approximately 10 million years, from the end of the Aptian (Melo et al., 2020) to the early part of that stage (Zhong et al., 2021). More importantly, it restricts their known distribution to Eurasia (Ding et al., 2020), so that the current fossil record of Compsognathidae is restricted to Laurasia. Similarly, excluding *S. placidus* from *Tyrannosauroidea* weakens the record of the group in Gondwana, which is now restricted to two isolated bones from the Aptian–Albian Eumeralla Formation of Australia, including a pubis (NMV P186046) and the type femur of *Timimus hermani* (Benson et al., 2012; Benson, Barrett, et al., 2010a; Benson, Carrano, & Brusatte, 2010b; Delcourt & Grillo, 2018), plus the records of *Megaraptora*, most probably nested within that group (Novas et al., 2013; Lamanna et al., 2020; Naish & Cau, 2022; Kotevski et al., 2024).

**FIGURE 32:**
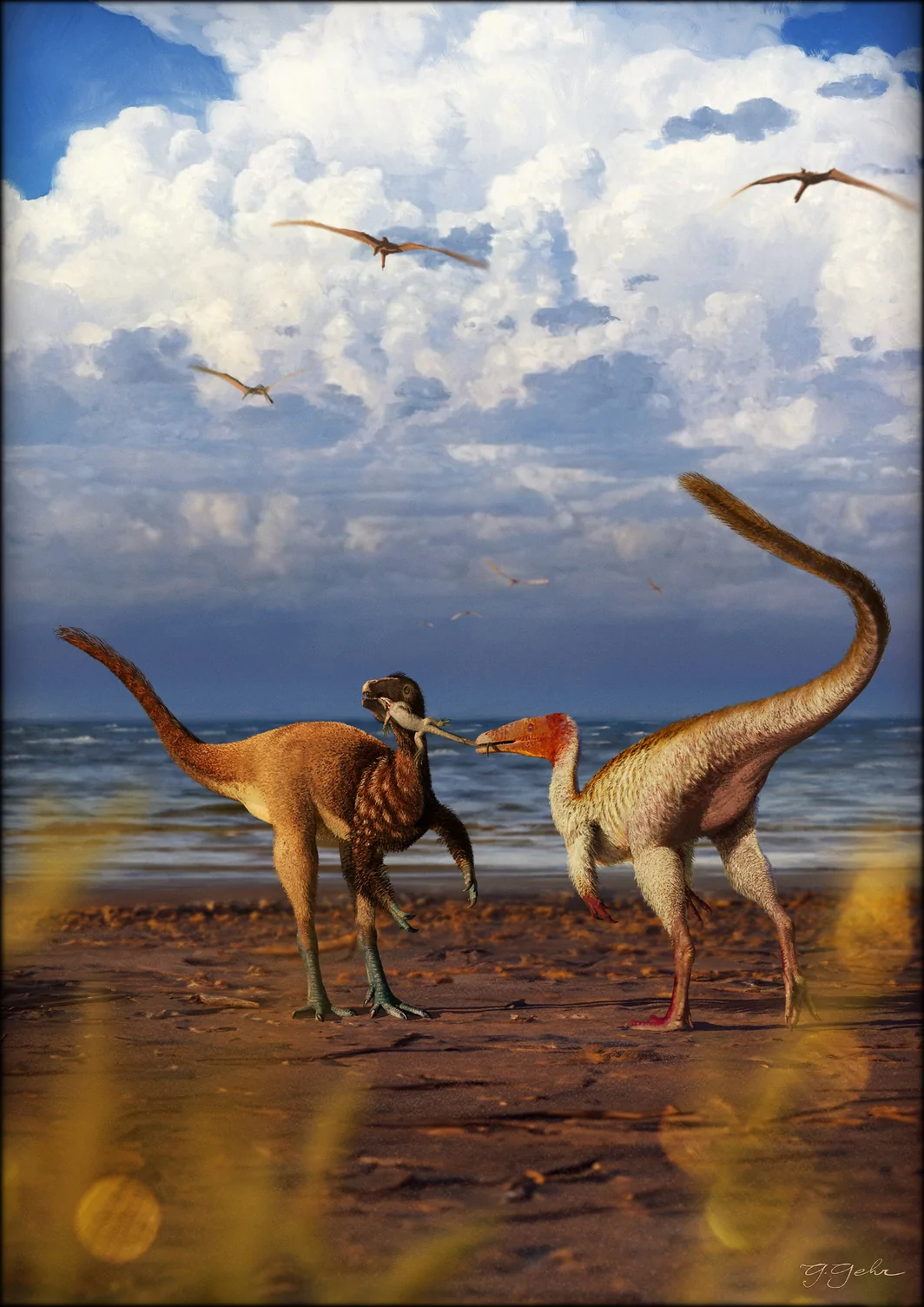
Hypothetical reconstruction of *Mirischia asymmetrica* (left) and *Santanaraptor placidus* (right) disputing a lizard in what is now northeastern Brazil ~112 million years ago (Early Cretaceous, Aptian). Illustration by Guilherme Gehr.

Brownstein (2021) recently suggested the nesting of *S. placidus* within Noasauridae, based on its non‐arctometatarsalian condition, where the shafts of metatarsals II and IV are narrower than that of metatarsal III. Even though the shaft of metatarsal II is indeed narrower than that of metatarsal III in *S. placidus*, which is typical of theropods, it is not as narrow as that of noasaurids (e.g., Brissón Egli et al., 2016; Carrano et al., 2002; Godoy et al., 2016; Rauhut & Carrano, 2016; Xu et al., 2009). Another feature mentioned by Brownstein (2021) is related to the dorsal surface of the distal end of metatarsal III, but this is not well preserved in *S. placidus*. Likewise, Qiu et al. (2025) nested *M. asymmetrica* within Sinosauropterygidae, but with no further comments on the morphological basis for such assignment (Figure 32).

The clade containing *J. langhami*, *M. asymmetrica*, *T. topwilsoni*, and *S. placidus* appears to trace back at least to the Late Jurassic of Laurasia, prior to the opening of the Apulian route, which allowed faunal exchange during the Early Cretaceous (Ding et al., 2020). In fact, several coelurosaur groups occur in both Laurasia and Gondwana at the time, including *Megaraptora*, as well as branches of *Ornithomimosauria*, Alvarezsauridae, and Dromaeosauridae (Ding et al., 2020). Our phylogenetic proposal better matches the available biogeographical data than those nesting the Romualdo coelurosaurs to the mostly Laurasian Compsognathidae and *Tyrannosauroidea*. Yet, further research, including more extensive field work, is needed to untangle the early radiation of coelurosaurs, including the affinity of the Romualdo forms.

### 4.3 A Gondwanan ornithomimosaur radiation and the origin of the clade

Despite its historical relevance, the affinities of *Coelu. fragilis* have been admittedly hard to define on the cladistic paradigm (Cau et al., 2015; Hendrickx et al., 2015; Rauhut, 2003; Senter, 2007; Smith et al., 2007; Zanno, 2010). We do not expect to settle this issue here, but briefly present the synapomorphies it shares with *Ornithomimosauria* in our analyses: centra of caudal trunk vertebrae much longer than high (char. 550); neural spines of mid‐caudal vertebrae subrectangular and sheet‐like (char. 610); neural spines of distal caudal vertebrae forming a low ridge (char. 615); internal tuberosity of the humerus triangular in cranial view (char. 663); a deep medial fossa on the proximal portion of the fibula (char 907). In addition, the shape of the pubic boot of *Coelu. fragilis* is reminiscent of that seen in some ornithomimosaurs (Ji et al., 2003). The recovery of *Coelu. fragilis*, as the earliest member of *Ornithomimosauria* would suggest a North American, Late Jurassic origin for the clade.

The presence of Ornithomimosaurs in Gondwana has been suggested on several occasions, with *Afromimus tenerensis*, *D. agilis*, *Elaphrosaurus bambergi*, *N. thwazi*, and *T. hermani* all, at one point, considered members of the clade (e.g., Galton, 1982; Rauhut, 2003; Rich & Vickers‐Rich, 1994; Sereno, 2017). However, the recognition of Noasauridae, a Gondwanan clade of ceratosaurs with some features common to most ornithomimosaurs, such as overall gracile limb proportions, elongated cervical vertebrae (e.g., Nopsca 1928) and edentulousness (de Souza et al., 2021; Pierossi et al., 2025; Wang et al., 2017), has led to most of these taxa being reclassified over the decades. Among those, only *N. thwazi* remains somewhat consistently recovered within *Ornithomimosauria* in recent analyses (Choiniere et al., 2012; Isasmendi et al., 2024; Lee et al., 2014; Serrano‐Brañas et al., 2020; Sues & Averianov, 2016). Although *T. hermani* remained within *Coelurosauria*, either with tyrannosauroid affinities (Benson et al., 2012; Delcourt & Grillo, 2018) or as a potential Dromaeosauridae (Agnolin et al., 2010), *E. bambergi*, *Af. tenerensis*, and *D. agilis* are now usually nested within ceratosaurs (e.g., Carrano & Sampson, 2008; Cerroni et al., 2019; Hendrickx et al., 2024; Rauhut & Carrano, 2016). *Gualicho shinyae* was first recovered, in close phylogenetic proximity to megaraptors, either as a carcharodontosaur, or close to coelurosaurs, depending on the position of *Megaraptora* (Apesteguía et al., 2016). Using a previous iteration of the MTWG dataset, both *D. agilis* and *Gu. shinyae* were recovered as early‐branching ornithomimosaurs in an unpublished Master's Thesis (Kellermann, 2021), a result replicated in our analyses here. A detailed discussion of the position of these two taxa is once again beyond the scope of this article, but in brief they share the following ornithomimosaur synapomorphies (the first of which is only recorded in *Gu. shinyae*): centra of caudal trunk vertebrae much longer than high (char. 550); neural spines of mid‐caudal vertebrae subrectangular and sheet‐like (char. 610); neural spines of distal caudal vertebrae forming a low ridge (char. 615); internal tuberosity of the humerus triangular in cranial view (char. 663); a deep medial fossa on the proximal end of the fibula (char 907). They also share the following synapomorphies with all ornithomimosaurs other than *Coelu. fragilis* and *N. thwazi* (the last of which is only recorded in *Gu. shinyae*): humerus straight in lateral view (char. 659); distal humeral condyles mainly distally developed (char 676); deltopectoral crest about one quarter the length of the humerus or less (char 668); pubic boot projecting both cranially and caudally (char 811).

## 5 CONCLUSIONS

The comprehensive reassessment of *S. placidus* (MN 4802‐V) and *M. asymmetric*a (SMNK 2349) presented here offers new insights into the anatomy, taxonomy, and phylogenetic affinities of these two coelurosaur theropods from the Early Cretaceous (Aptian) Romualdo Formation of Brazil. Despite the minor overlapping of skeletal elements, both specimens exhibit consistent anatomical differences that support their recognition as distinct species. These differences are particularly evident in the configuration of the ischial obturator plate and the structure of the proximal tibial condyles.

Some of our phylogenetic results place *S. placidus* and *M. asymmetrica* together with *J. langhami* and *T. topwilsoni*, from the Late Jurassic of Laurasia, in a clade of early branching maniraptoromorphs. This is the first time that these Brazilian taxa are found closely related within a lesser‐inclusive clade, corroborating previous assumptions of close biogeographic relations between Laurasian and Gondwanan landmasses by the Early Cretaceous. Further biogeographic patterns are, however, harder to define, especially having in mind the still patchy record of Jurassic theropods in areas such as Africa, Australia, India, Antarctica, and South America.

## AUTHOR CONTRIBUTIONS

**Rafael Delcourt:** Conceptualization; investigation; funding acquisition; writing – original draft; methodology; validation; visualization; writing – review and editing; formal analysis; project administration; data curation; supervision; resources. **Orlando Nelson Grillo:** Supervision; data curation; writing – original draft; writing – review and editing; investigation; validation; visualization; methodology; formal analysis. **Christophe Hendrickx:** Investigation; funding acquisition; writing – original draft; writing – review and editing; data curation; supervision; methodology; validation; visualization; formal analysis. **Maximilian Kellermann:** Investigation; writing – original draft; methodology; validation; visualization; writing – review and editing; formal analysis; data curation; supervision. **Max Cardoso Langer:** Conceptualization; investigation; funding acquisition; writing – original draft; methodology; validation; visualization; writing – review and editing; formal analysis; project administration; data curation; supervision; resources.

## Supporting information


**DATA S1.** Supporting Information.

**DATA S2.** Supporting Information.

## References

[ar70085-bib-0001] Agnolin, F. L. , Ezcurra, M. D. , Pais, D. F. , & Salisbury, S. W. (2010). A reappraisal of the cretaceous non‐avian dinosaur faunas from Australia and New Zealand: Evidence for their Gondwanan affinities. Journal of Systematic Palaeontology, 8(2), 257–300.

[ar70085-bib-0003] Allain, R. , Vullo, R. , Le Loeuff, J. , & Tournepiche, J. F. (2014). European ornithomimosaurs (Dinosauria, Theropoda): An undetected record. Geologica Acta, 12(2), 127–135.

[ar70085-bib-0004] Allain, R. , Xaisanavong, T. , Richir, P. , & Khentavong, B. (2012). The first definitive Asian spinosaurid (Dinosauria: Theropoda) from the early cretaceous of Laos. Naturwissenschaften, 99, 369–377. 22528021 10.1007/s00114-012-0911-7

[ar70085-bib-0005] Apesteguía, S. , Smith, N. D. , Juárez Valieri, R. , & Makovicky, P. J. (2016). An unusual new theropod with a didactyl manus from the upper cretaceous of Patagonia, Argentina. PLoS One, 11, 1–41. 10.1371/journal.pone.0157793 PMC4943716 27410683

[ar70085-bib-0006] Arai, M. , & Assine, M. L. (2020). Chronostratigraphic constraints and paleoenvironmental interpretation of the Romualdo formation (Santana group, Araripe Basin, northeastern Brazil) based on palynology. Cretaceous Research, 116, 104610.

[ar70085-bib-0007] Aranciaga Rolando, A. M. , Egli, F. B. , Sales, M. A. F. , Martinelli, A. G. , Canale, J. I. , & Ezcurra, M. D. (2018). A supposed Gondwanan oviraptorosaur from the Albian of Brazil represents the oldest south American megaraptoran. Cretaceous Research, 84, 107–119.

[ar70085-bib-0008] Aureliano, T. , Ghilardi, A. M. , Buck, P. V. , Fabbri, M. , Samathi, A. , Delcourt, R. , Fernandes, M. A. , & Sander, M. (2018). Semi‐aquatic adaptations in a spinosaur from the lower cretaceous of Brazil. Cretaceous Research, 90, 283–295.

[ar70085-bib-0010] Azuma, Y. , & Currie, P. J. (2000). A new carnosaur (Dinosauria: Theropoda) from the lower cretaceous of Japan. Canadian Journal of Earth Sciences, 37, 1735–1753.

[ar70085-bib-0012] Barsbold, R. (1976). K evolyutsii i sistematike pozdnemezozoyskikh khishchnykh dinozavrov (the evolution and systematics of late Mesozoic carnivorous dinosaurs) (Russian). The Joint Soviet‐Mongolian Paleontological Expedition, Transactions, 3, 68–75.

[ar70085-bib-0013] Barsbold, R. , Currie, P. , Myhrvold, N. , Osmolska, H. , Tsogtbaatar, K. , & Watabe, M. (2000). A pygostyle from a non‐avian theropod. Nature, 403, 155–156. 10646588 10.1038/35003103

[ar70085-bib-0014] Bates, K. T. , Maidment, S. C. R. , Allen, V. , & Barrett, P. M. (2012). Computational modelling of locomotor muscle moment arms in the basal dinosaur *Lesothosaurus diagnosticus*: Assessing convergence between birds and basal ornithischians. Journal of Anatomy, 220, 212–232. 22211275 10.1111/j.1469-7580.2011.01469.x PMC3381616

[ar70085-bib-0015] Benson, R. B. J. (2008). New information on *Stokesosaurus*, a tyrannosauroid (Dinosauria: Theropoda) from North America and the United Kingdom. Journal of Vertebrate Paleontology, 28, 732–750.

[ar70085-bib-0016] Benson, R. B. J. , Barrett, P. M. , Rich, T. H. , & Vickers‐Rich, P. (2010a). A southern tyrant reptile. Science, 327, 1613. 20339066 10.1126/science.1187456

[ar70085-bib-0017] Benson, R. B. J. , Carrano, M. T. , & Brusatte, S. L. (2010b). A new clade of archaic large‐bodied predatory dinosaurs (Theropoda: Allosauroidea) that survived to the latest Mesozoic. Naturwissenschaften, 97, 71–78. 19826771 10.1007/s00114-009-0614-x

[ar70085-bib-0018] Benson, R. B. J. , Rich, T. H. , Vickers‐Rich, P. , & Hall, M. (2012). Theropod fauna from southern Australia indicates high polar diversity and climate‐driven dinosaur provinciality. PLoS One, 7, e37122. 22615916 10.1371/journal.pone.0037122 PMC3353904

[ar70085-bib-0193] Bonaparte, J. F. (1991). Los vertebrados fósiles de la Formación Río Colorado de Neuquén y cercanias, Cretácico superior, Argentina. Revista del Museo Argentino de Ciencias Naturales “Bernardino Rivadavia”. Paleontología, 4(3), 17–123.

[ar70085-bib-0019] Brissón Egli, F. , AgnolÍn, F. L. , & Novas, F. E. (2016). A new specimen of *Velocisaurus unicus* (Theropoda, Abelisauroidea) from the Paso Córdoba locality (Santonian), Río Negro, Argentina. Journal of Vertebrate Paleontology, 36, 4634:e1119156.

[ar70085-bib-0020] Brochu, C. A. (2003). Osteology of *tyrannosaurus rex*: Insights from a nearly complete skeleton and high‐resolution computed tomographic analysis of the skull. Journal of Vertebrate Paleontology, 22, 1–138.

[ar70085-bib-0021] Brownstein, C. D. (2021). Dinosaurs from the Santonian: Campanian Atlantic coastline substantiate phylogenetic signatures of vicariance in cretaceous North America. Royal Society Open Science, 8, 210127. 34457333 10.1098/rsos.210127 PMC8385347

[ar70085-bib-0022] Brusatte, S. , Benson, R. , & Hutt, S. (2008). The osteology of *Neovenator salerii* (Dinosauria: Theropoda) from the Wealden group (Barremian) of the Isle of Wight. Monographs of the Palaeontographical Society, 162(631), 1–75.

[ar70085-bib-0188] Brusatte, S. L. , & Benson, R. B. (2013). The systematics of late Jurassic tyrannosauroid theropods from Europe and North America. Acta Palaeontologica Polonica, 58(1), 47–54.

[ar70085-bib-0024] Brusatte, S. L. , Carr, T. D. , & Norell, M. a. (2012). The osteology of *Alioramus*, a gracile and long‐snouted tyrannosaurid (Dinosauria: Theropoda) from the late cretaceous of Mongolia. Bulletin of the American Museum of Natural History, 366, 1–197.

[ar70085-bib-0025] Brusatte, S. L. , Lloyd, G. T. , Wang, S. C. , & Norell, M. A. (2014). Gradual assembly of avian body plan culminated in rapid rates of evolution across the dinosaur‐bird transition. Current Biology, 24, 2386–2392. 25264248 10.1016/j.cub.2014.08.034

[ar70085-bib-0026] Brusatte, S. L. , Norell, M. A. , Carr, T. D. , Erickson, G. M. , Hutchinson, J. R. , Balanoff, A. M. , Bever, G. S. , Choiniere, J. N. , Makovicky, P. J. , & Xu, X. (2010). Tyrannosaur paleobiology: New research on ancient exemplar organisms. Science, 329, 1481–1485. 20847260 10.1126/science.1193304

[ar70085-bib-0027] Buffetaut, E. , Suteethorn, V. , & Tong, H. (2009). An early “ostrich dinosaur” (Theropoda: Ornithomimosauria) Fromthe early cretaceous Sao Khua formation of NE Thailand. In E. Buffetaut , G. Cuny , J. Leloeuff , & V. Suteethorn (Eds.), Late Palaeozoic and Mesozoic ecosystems in SE Asia (pp. 229–243). the Geological Society, Special Publications.

[ar70085-bib-0028] Canale, J. I. , Apesteguía, S. , Gallina, P. A. , Mitchell, J. , Smith, N. D. , Cullen, T. M. , Shinya, A. , Haluza, A. , Gianechini, F. A. , & Makovicky, P. J. (2022). New giant carnivorous dinosaur reveals convergent evolutionary trends in theropod arm reduction. Current Biology, 32, 3195–3202.e5. 35803271 10.1016/j.cub.2022.05.057

[ar70085-bib-0029] Canale, J. I. , Novas, F. E. , & Pol, D. (2015). Osteology and phylogenetic relationships of *Tyrannotitan chubutensis* Novas, de Valais, Vickers‐Rich and Rich, 2005 (Theropoda: Carcharodontosauridae) from the lower cretaceous of Patagonia, Argentina. Historical Biology, 27, 1–32.

[ar70085-bib-0179] Cantino, P. D. , & De Queiroz, K. (Eds.). (2020). PhyloCode: A phylogenetic code of biological nomenclature. CRC Press.

[ar70085-bib-0184] Carpenter, K. (Ed.). (2005). The carnivorous dinosaurs. Indiana University Press.

[ar70085-bib-0185] Carpenter, K. , Miles, C. , Ostrom, J. H. , & Cloward, K. (2005). Redescription of the small maniraptoran theropods Ornitholestes and Coelurus from the upper Jurassic Morrison formation of Wyoming. In Carnivorous dinosaurs (p. 49).

[ar70085-bib-0030] Carrano, M. T. , & Hutchinson, J. R. (2002). Pelvic and hindlimb musculature of *Tyrannosaurus rex* (Dinosauria: Theropoda). Journal of Morphology, 253, 207–228. 12125061 10.1002/jmor.10018

[ar70085-bib-0031] Carrano, M. T. , Hutchinson, J. R. , & Sampson, S. D. (2005). New information on *Segisaurus halli*, a small theropod dinosaur from the early Jurassic of Arizona. Journal of Vertebrate Paleontology, 25, 835–849.

[ar70085-bib-0032] Carrano, M. T. , & Sampson, S. D. (2008). The phylogeny of Ceratosauria (Dinosauria: Theropoda). Journal of Systematic Palaeontology, 6, 183–236.

[ar70085-bib-0033] Carrano, M. T. , Sampson, S. D. , & Forster, C. a. (2002). The osteology of *Masiakasaurus knopfleri*, a small abelisauroid (Dinosauria: Theropoda) from the late cretaceous of Madagascar. Journal of Vertebrate Paleontology, 22, 510–534.

[ar70085-bib-0034] Cau, A. (2018). The assembly of the avian body plan: A 160‐million‐year long process. Bollettino Della Societa Paleontologica Italiana, 57(1), 1–25.

[ar70085-bib-0035] Cau, A. (2024). A unified framework for predatory dinosaur macroevolution. Bollettino Della Società Paleontologica Italiana, 63, 1–19.

[ar70085-bib-0036] Cau, A. , Brougham, T. , & Naish, D. (2015). The phylogenetic affinities of the bizarre late cretaceous Romanian theropod *Balaur bondoc* (Dinosauria, Maniraptora): Dromaeosaurid or flightless bird? PeerJ, 3, e1032. 26157616 10.7717/peerj.1032 PMC4476167

[ar70085-bib-0037] Cerroni, M. A. , Agnolin, F. L. , Brissón Egli, F. , & Novas, F. E. (2019). The phylogenetic position of Afromimus tenerensis Sereno, 2017 and its paleobiogeographical implications. Journal of African Earth Sciences, 159, 103572.

[ar70085-bib-0038] Charig, A. J. , & Milner, A. C. (1997). *Baryonyx walkeri*, a fish‐eating dinosaur from the Wealden of Surrey. Bulletin‐Natural History Museum Geology Series, 53, 11–70.

[ar70085-bib-0039] Choiniere, J. N. , Clark, J. M. , Forster, C. A. , Xu, X. , George, T. , & Sciences, B. (2010). A basal coelurosaur (Dinosauria: Theropoda) from the late Jurassic (Oxfordian) of the Shishugou formation in Wucaiwan, People's Republic of China, 30, 1773–1796.

[ar70085-bib-0040] Choiniere, J. N. , Forster, C. A. , & Klerk, W. J. (2012). New information on *Nqwebasaurus thwazi*, a coelurosaurian theropod from the early cretaceous Kirkwood formation in South Africa. Journal of African Earth Sciences, 71–72, 1–17.

[ar70085-bib-0041] Clark, G. A., Jr. (1993). Termini situm et directionem partium corporis indicantes. In J. J. Baumel (Ed.), Handbook of avian anatomy: Nomina anatomica avium. Nuttall Ornithological Club.

[ar70085-bib-0042] Coria, R. a. , Currie, P. J. , & Carabajal, A. P. (2006). A new abelisauroid theropod from northwestern Patagonia. Canadian Journal of Earth Sciences, 43, 1283–1289.

[ar70085-bib-0043] Cuesta, E. , Ortega, F. , & Sanz, J. L. (2018). Appendicular osteology of *Concavenator corcovatus* (Theropoda: Carcharodontosauridae) from the lower cretaceous of Spain. Journal of Vertebrate Paleontology, 38, 1–24.

[ar70085-bib-0045] Currie, P. J. , & Chen, P. (2001). Anatomy of *Sinosauropteryx prima* from Liaoning, northeastern China. Canadian Journal of Earth Sciences, 38, 1705–1727.

[ar70085-bib-0046] Currie, P. J. , & Zhao, X.‐J. (1993). A new carnosaur (Dinosauria, Theropoda) from the Jurassic of Xinjiang, People's Republic of China. Canadian Journal of Earth Sciences, 30, 2037–2081.

[ar70085-bib-0047] Custódio, M. A. , Quaglio, F. , Warren, L. V. , Simões, M. G. , Fürsich, F. T. , Perinotto, J. A. J. , & Assine, M. L. (2017). The transgressive‐regressive cycle of the Romualdo formation (Araripe Basin): Sedimentary archive of the early cretaceous marine ingression in the interior of Northeast Brazil. Sedimentary Geology, 359, 1–15.

[ar70085-bib-0048] Dal Sasso, C. , & Maganuco, S. (2011). *Scipionyx samniticus* (Theropoda: Compsognathidae) from the lower cretaceous of Italy. Osteology, ontogenetic assessment, phylogeny, soft tissue anatomy, taphonomy and palaeobiology. Memorie Della Società Italiana Di Scienze Naturali e Del Museo Civico Di Storia Naturale Di Milano, 27, 1–281.

[ar70085-bib-0049] de Araújo Gomes, B. , Cadeira Prado, L. A. , & Franca Barreto, A. M. (2023). New invertebrate sites and marine ingressions in the Romualdo formation, Aptian‐Albian, Araripe sedimentary basin, NE Brazil. Journal of South American Earth Sciences, 123, 104249.

[ar70085-bib-0050] de Lima, F. J. , Saraiva, A. Á. F. , & Sayão, J. M. (2012). Revisão da paleoflora das formações Missão Velha, Crato e Romualdo, Bacia do Araripe, Nordeste do Brasil. Estudos Geológicos, 22, 99–115.

[ar70085-bib-0180] De Queiroz, K. , Cantino, P. , & Gauthier, J. (2020). Phylonyms: A companion to the PhyloCode. CRC Press.

[ar70085-bib-0051] de Souza, G. A. , Soares, M. B. , Weinschütz, L. C. , Wilner, E. , Lopes, R. T. , de Araújo, O. M. O. , & Kellner, A. W. A. (2021). The first edentulous ceratosaur from South America. Scientific Reports, 11, 22281. 34795306 10.1038/s41598-021-01312-4 PMC8602317

[ar70085-bib-0052] Delcourt, R. , & Grillo, O. N. (2018). Tyrannosauroids from the southern hemisphere: Implications for biogeography, evolution, and taxonomy. Palaeogeography, Palaeoclimatology, Palaeoecology, 511, 379–387.

[ar70085-bib-0053] Ding, A. , Pittman, M. , Upchurch, P. , O'Connor, J. , Field, D. J. , & Xu, X. (2020). Chapter 4: The biogeography of coelurosaurian theropods and its impact on their evolutionary history. In Bulletin of the American Museum of Natural History (Vol. 440, pp. 117–157). Bulletin of the AMNH.

[ar70085-bib-0181] Ezcurra, M. D. (2024). Exploring the effects of weighting against homoplasy in genealogies of palaeontological phylogenetic matrices. Cladistics, 40(3), 242–281. 38728134 10.1111/cla.12581

[ar70085-bib-0054] Forster, C. A. , O'Connor, P. M. , Chiappe, L. M. , & Turner, A. H. (2020). The osteology of the late cretaceous paravian *Rahonavis ostromi* from Madagascar. Palaeontologia Electronica, 23(2), a29.

[ar70085-bib-0055] Foth, C. , van de Kamp, T. , Tischlinger, H. , Kantelis, T. , Carney, R. M. , Zuber, M. , Hamann, E. , Wallaard, J. J. W. , Lenz, N. , Rauhut, O. W. M. , & Frey, E. (2025). A new *archaeopteryx* from the lower Tithonian Mörnsheim formation at Mühlheim (late Jurassic). Fossil Record, 28, 17–43.

[ar70085-bib-0056] Funston, G. F. , Chinzorig, T. , Tsogtbaatar, K. , Kobayashi, Y. , Sullivan, C. , & Currie, P. J. (2020). A new two‐fingered dinosaur sheds light on the radiation of Oviraptorosauria: Two‐fingered oviraptorid. Royal Society Open Science, 7(10), 201184. 33204472 10.1098/rsos.201184 PMC7657903

[ar70085-bib-0057] Galton, P. M. (1982). Elaphrosaurus, an ornithomimid dinosaur from the upper jurassic of north America and Africa. Paläontologische Zeitschrift, 56(3–4), 265–275.

[ar70085-bib-0058] Gao, Y. (1993). A new species of *Szechuanosaurus* from the middle Jurassic of Dashanpu, Zigong, Sichuan. Vertebrata Palasiatica, 31, 308–314. [In Chinese].

[ar70085-bib-0059] Gianechini, F. A. , Makovicky, P. J. , Apesteguía, S. , & Cerda, I. (2018). Postcranial skeletal anatomy of the holotype and referred specimens of *Buitreraptor gonzalezorum* Makovicky, Apesteguía and Agnolín 2005 (Theropoda, Dromaeosauridae), from the late cretaceous of Patagonia. PeerJ, 6, e4558. 29607264 10.7717/peerj.4558 PMC5875404

[ar70085-bib-0060] Gilmore, C. W. (1920). Osteology of the carnivorous Dinosauria in the United States National Museum, with special reference to the genera *Antrodemus* (*Allosaurus*) and *Ceratosaurus* . Bulletin of the United States National Museum, 110, 1–159.

[ar70085-bib-0061] Godoy, P. L. , Bronzati, M. , Eltink, E. , Marsola, J. C. D. A. , Cidade, G. M. , Langer, M. C. , & Montefeltro, F. C. (2016). Postcranial anatomy of *Pissarrachampsa sera* (Crocodyliformes, Baurusuchidae) from the late cretaceous of Brazil: Insights on lifestyle and phylogenetic significance. PeerJ, 2016, e2075. 10.7717/peerj.2075 PMC4888301 27257551

[ar70085-bib-0062] Göhlich, U. B. , & Chiappe, L. M. (2006). A new carnivorous dinosaur from the late Jurassic Solnhofen archipelago. Nature, 440, 329–332. 16541071 10.1038/nature04579

[ar70085-bib-0063] Goloboff, P. (1999). Analyzing large data sets in reasonable times: Solutions for composite optima. Cladistics, 15, 415–428. 34902941 10.1111/j.1096-0031.1999.tb00278.x

[ar70085-bib-0064] Goloboff, P. A. , & Morales, M. E. (2023). TNT version 1.6, with a graphical interface for MacOS and Linux, including new routines in parallel. Cladistics, 39, 144–153. 36682054 10.1111/cla.12524

[ar70085-bib-0065] Griffin, C. T. (2018). Developmental patterns and variation among early theropods. Journal of Anatomy, 232, 604–640. 29363129 10.1111/joa.12775 PMC5835796

[ar70085-bib-0068] Hendrickx, C. , Bell, P. R. , Pittman, M. , Milner, A. R. C. , Cuesta, E. , O'Connor, J. , Loewen, M. , Currie, P. J. , Mateus, O. , Kaye, T. G. , & Delcourt, R. (2022). Morphology and distribution of scales, dermal ossifications, and other non‐feather integumentary structures in non‐avialan theropod dinosaurs. Biological Reviews, 97, 960–1004. 34991180 10.1111/brv.12829

[ar70085-bib-0069] Hendrickx, C. , Cerroni, M. A. , Agnolín, F. L. , Catalano, S. , Ribeiro, C. F. , & Delcourt, R. (2024). Osteology, relationship, and feeding ecology of the theropod dinosaur *Noasaurus leali* Bonaparte and Powell, 1980, from the late cretaceous of North‐Western Argentina. Zoological Journal of the Linnean Society, 202, zlae150.

[ar70085-bib-0070] Hendrickx, C. , Hartman, S. A. , & Mateus, O. (2015). An overview of non‐avian theropod discoveries and classification. PalArch's Journal of Vertebrate Palaeontology, 12(1), 1–73.

[ar70085-bib-0071] Hendrickx, C. , Mateus, O. , Araújo, R. , & Choiniere, J. (2019). The distribution of dental features in non‐avian theropod dinosaurs: Taxonomic potential, degree of homoplasy, and major evolutionary trends. Palaeontologia Electronica, 22, 1–110.

[ar70085-bib-0072] Hocknull, S. A. , White, M. A. , Tischler, T. R. , Cook, A. G. , Calleja, N. D. , Sloan, T. , & Elliott, D. A. (2009). New mid‐cretaceous (latest Albian) dinosaurs from Winton, Queensland, Australia. PLoS One, 4, e6190. 19584929 10.1371/journal.pone.0006190 PMC2703565

[ar70085-bib-0074] Holtz, T. R., Jr. (2004). Tyrannosauroidea. In D. B. Weishampel , P. Dodson , & H. Osmólska (Eds.), The Dinosauria (Vol. 2, 2nd ed., pp. 111–136). University of California Press.

[ar70085-bib-0075] Hutchinson, J. R. (2001). The evolution of femoral osteology and soft tissues on the line to extant birds (Neornithes). Zoological Journal of the Linnean Society, 131, 169–197.

[ar70085-bib-0076] Hutchinson, J. R. (2021). The evolutionary biomechanics of locomotor function in giant land animals. Journal of Experimental Biology, 224, 1–15. 10.1242/jeb.217463 PMC8214834 34100541

[ar70085-bib-0077] Hwang, S. H. , Norell, M. a. , Qiang, J. , & Keqin, G. (2004). A large compsognathid from the early cretaceous Yixian formation of China. Journal of Systematic Palaeontology, 2, 13–30.

[ar70085-bib-0078] Isasmendi, E. , Cuesta, E. , Díaz‐Martínez, I. , Company, J. , Sáez‐Benito, P. , Viera, L. I. , Torices, A. , & Pereda‐Suberbiola, X. (2024). Increasing the theropod record of Europe: a new basal spinosaurid from the Enciso Group of the Cameros Basin (La Rioja, Spain). Evolutionary implications and palaeobiodiversity. Zoological Journal of the Linnean Society, 202(3), zlad193.

[ar70085-bib-0079] Ji, Q. , Norell, M. a. , Makovicky, P. J. , Gao, K.‐Q. , Ji, S. , & Yuan, C. (2003). An early ostrich dinosaur and implications for ornithomimosaur phylogeny. American Museum Novitates, 3420, 1.

[ar70085-bib-0080] Kellermann, M. (2021). In O. W. M. Rauhut (Ed.), New data on dinosaur diversity in the “middle” cretaceous of North Africa. Ludwig‐Maximilians‐Universität München.

[ar70085-bib-0081] Kellermann, M. , Cuesta, E. , & Rauhut, O. W. M. (2025). Re‐evaluation of the Bahariya formation carcharodontosaurid (Dinosauria: Theropoda) and its implications for allosauroid phylogeny. PLoS One, 20, e0311096. 39808629 10.1371/journal.pone.0311096 PMC11731741

[ar70085-bib-0082] Kellermann, M. , & Rauhut, O. W. M. (2022). The “ghost” theropods of the Bahriya oasis and their bearing on cretaceous African theropod faunas. In M. Belvedere , B. Mecozzi , O. Amore , & R. Sardella (Eds.), Abstract book of the XIX annual conference of the European Association of Vertebrate Palaeontologists, Benevento/Pietraroja, Italy, 27th June‐2nd July 2022. PalaeoVertebrata (Vol. 1, p. 224). 10.18563/pv.eavp2022

[ar70085-bib-0083] Kellner, A. W. A. (1996). Fossilized theropod soft tissue. Nature, 379, 32.

[ar70085-bib-0084] Kellner, A. W. A. (1999). Short note on a new dinosaur (Theropoda, Coelurosauria) from the Santana formation (Romualdo member, Albian), northeastern Brazil. Boletim Do Museu Nacional Geologia, 49, 1–8.

[ar70085-bib-0085] Kellner, A. W. A. (2001). New information on the theropod dinosaurs from the Santana formation (Aptian‐Albian), Araripe Basin, northeastern Brazil. Journal of Vertebrate Paleontology, 21(Suppl 3), 67A.

[ar70085-bib-0086] Kellner, A. W. A. (2013). A new unusual tapejarid (Pterosauria, Pterodactyloidea) from the early cretaceous Romualdo formation, Araripe Basin, Brazil. Earth and Environmental Science Transactions of the Royal Society of Edinburgh, 103, 409–421.

[ar70085-bib-0087] Kellner, A. W. A. , & Campos, D. A. (1996). First early cretaceous theropod dinosaur from Brazil with comments on Spinosauridae. Neues Jahrbuch für Geologie und Paläontologie, 199, 151–166.

[ar70085-bib-0192] Kirkland, J. I. , & Wolfe, D. G. (2001). First definitive therizinosaurid (Dinosauria; Theropoda) from North America. Journal of Vertebrate Paleontology, 21(3), 410–414.

[ar70085-bib-0088] Kobayashi, Y. , & Barsbold, R. (2005). Reexamination of a primitive ornithomimosaur, *Garudimimus brevipes* Barsbold, 1981 (Dinosauria: Theropoda), from the late cretaceous of Mongolia. Canadian Journal of Earth Sciences, 42, 1501–1521.

[ar70085-bib-0089] Kobayashi, Y. , & Lü, J. U. N. C. (2003). A new ornithomimid dinosaur with gregarious habits from the late cretaceous of China. Acta Palaeontologica Polonica, 48, 235–259.

[ar70085-bib-0190] Kotevski, J. , Duncan, R. J. , Pentland, A. H. , Rule, J. P. , Vickers‐Rich, P. , Rich, T. H. , Fitzgeralda, E. M. G. , Evans, A. R. , & Poropat, S. F. (2024). A megaraptorid (Dinosauria: Theropoda) frontal from the upper strzelecki group (lower cretaceous) of Victoria, Australia. Cretaceous Research, 154, 105769.

[ar70085-bib-0090] Lacerda, M. B. S. , Bittencourt, J. S. , & Hutchinson, J. R. (2023). Macroevolutionary patterns in the pelvis, stylopodium and zeugopodium of megalosauroid theropod dinosaurs and their importance for locomotor function. Royal Society Open Science, 10, 230481. 37593714 10.1098/rsos.230481 PMC10427828

[ar70085-bib-0189] Lamanna, M. C. , Casal, G. A. , Martínez, R. D. , & Ibiricu, L. M. (2020). Megaraptorid (Theropoda: Tetanurae) partial skeletons from the upper cretaceous Bajo Barreal formation of central Patagonia, Argentina: Implications for the evolution of large body size in Gondwanan megaraptorans. Annals of Carnegie Museum, 86(3), 255–294.

[ar70085-bib-0091] Langer, M. A. X. C. (2003). The pelvic and hind limb anatomy of the stem‐sauropodomorph *Saturnalia tupiniquim* (late Triassic, Brazil). Paleo Bios, 23, 1–30.

[ar70085-bib-0092] Langer, M. C. , Ezcurra, M. D. , Rauhut, O. W. M. , Benton, M. J. , Knoll, F. , McPhee, B. W. , Novas, F. E. , Pol, D. , & Brusatte, S. L. (2017). Untangling the dinosaur family tree. Nature, 551, E1–E3. 29094688 10.1038/nature24011

[ar70085-bib-0093] Lee, Y.‐N. , Barsbold, R. , Currie, P. J. , Kobayashi, Y. , Lee, H.‐J. , Godefroit, P. , Escuillié, F. , & Chinzorig, T. (2014). Resolving the long‐standing enigmas of a giant ornithomimosaur *Deinocheirus mirificus* . Nature, 515, 257–260. 25337880 10.1038/nature13874

[ar70085-bib-0094] Liao, C. C. , Zanno, L. E. , Wang, S. , & Xu, X. (2021). Postcranial osteology of *Beipiaosaurus inexpectus* (Theropoda: Therizinosauria). PLoS One, 16, e0257913. 34591927 10.1371/journal.pone.0257913 PMC8483305

[ar70085-bib-0095] Lü, J. , Yi, L. , Zhong, H. , & Wei, X. (2013). A new oviraptorosaur (Dinosauria: Oviraptorosauria) from the late cretaceous of southern China and its paleoecological implications. PLoS One, 8, e80557. 24312233 10.1371/journal.pone.0080557 PMC3842309

[ar70085-bib-0096] Macdonald, I. , & Currie, P. J. (2019). Description of a partial *Dromiceiomimus* (Dinosauria: Theropoda) skeleton with comments on the validity of the genus. Canadian Journal of Earth Sciences, 56, 129–157.

[ar70085-bib-0097] Madsen, J. H. (1976). A second new theropod dinosaur from the late Jurassic of east Central Utah. Utah Geology, 3(1), 51–60. 10.34191/UG-3-1_51

[ar70085-bib-0098] Madsen, J. H., Jr. (1976). Allosaurus fragilis: a revised osteology. Utah Geological and Mining Survey Bulletin, 109, 1–163.

[ar70085-bib-0099] Maisey, J. G. (1991). Santana fossils: An illustrated atlas. TFH Publications Incorporated.

[ar70085-bib-0100] Makovicky, P. J. , Kobayashi, Y. , & Currie, P. J. (2004). Ornithomimosauria. In D. B. Weishampel , P. Dodson , & H. Osmólska (Eds.), The Dinosauria (2nd ed., pp. 137–150). University of California Press.

[ar70085-bib-0102] Malafaia, E. , Mocho, P. , Escaso, F. , & Ortega, F. (2020). A new carcharodontosaurian theropod from the Lusitanian Basin: Evidence of allosauroid sympatry in the European late Jurassic. Journal of Vertebrate Paleontology, 40(1), e1768106.

[ar70085-bib-0103] Marsh, A. D. , & Rowe, T. B. (2020). A comprehensive anatomical and phylogenetic evaluation of *Dilophosaurus wetherilli* (Dinosauria, Theropoda) with descriptions of new specimens from the Kayenta formation of northern Arizona. Journal of Paleontology, 94, 1–103.

[ar70085-bib-0104] Martill, D. M. , Frey, E. , Sues, H.‐D. , & Cruickshank, A. R. I. (2000). Skeletal remains of a small theropod dinosaur with associated soft structures from the lower cretaceous Santana formation of northeastern Brazil. Canadian Journal of Earth Sciences, 37, 891–900.

[ar70085-bib-0105] Martill, D. M. , Vidovic, S. U. , Howells, C. , & Nudds, J. R. (2016). The oldest Jurassic dinosaur: a basal neotheropod from the Hettangian of Great Britain. PLoS One, 11, e0145713. 26789843 10.1371/journal.pone.0145713 PMC4720452

[ar70085-bib-0106] Martínez, R. N. , & Apaldetti, C. (2017). A late Norian‐Rhaetian coelophysid neotheropod (Dinosauria, Saurischia) from the quebrada del barro formation, northwestern Argentina. Ameghiniana, 54, 488.

[ar70085-bib-0107] McFeeters, B. , Ryan, M. J. , & Cullen, T. M. (2018). Positional variation in pedal unguals of north American ornithomimids (Dinosauria, Theropoda): a response to Brownstein (2017). Vertebrate Anatomy Morphology Palaeontology, 6, 60–67.

[ar70085-bib-0108] McFeeters, B. , Ryan, M. J. , Schröder‐Adams, C. , & Cullen, T. M. (2016). A new ornithomimid theropod from the Dinosaur Park formation of Alberta, Canada. Journal of Vertebrate Paleontology, 36, e1221415.

[ar70085-bib-0109] McGowan, C. (1979). The hind limb musculature of the brown kiwi, *Apteryx australis* mantelli. Journal of Morphology, 160, 33–73. 458856 10.1002/jmor.1051600105

[ar70085-bib-0111] Melo, R. M. , Guzmán, J. , Almeida‐Lima, D. , Piovesan, E. K. , de Neumann, V. H. , & Sousa, A. (2020). New marine data and age accuracy of the Romualdo formation, Araripe Basin, Brazil. Scientific Reports, 10, 15779. 32978467 10.1038/s41598-020-72789-8 PMC7519656

[ar70085-bib-0178] Mendes, P. V. , Silva, H. P. , Bastos, M. , Bittar, V. , Reis, S. , & Rodrigues‐Carvalho, C. (2022). Osteological collections of the National Museum in Brazil: Challenges and new perspectives for a historical collection. Forensic Science, 2(2), 287–301.

[ar70085-bib-0112] Naish, D. , & Cau, A. (2022). The osteology and affinities of *Eotyrannus lengi*, a tyrannosauroid theropod from the Wealden supergroup of southern England. PeerJ, 10, e12727. 35821895 10.7717/peerj.12727 PMC9271276

[ar70085-bib-0113] Naish, D. , Martill, D. M. , & Frey, E. (2004). Ecology, systematics and biogeographical relationships of dinosaurs, including a new theropod from the Santana formation (?Albian, early cretaceous) of Brazil. Historical Biology, 16, 57–70.

[ar70085-bib-0163] Nomina Anatomica Veterinaria . (2005). International committee on veterinary gross anatomical nomenclature (Fifth ed.). Hannover.

[ar70085-bib-0114] Norell, M. A. , & Makovicky, P. J. (1997). Important features of the dromaeosaur skeleton: Information from a new specimen. American Museum Novitates, 3215, 1–28.

[ar70085-bib-0115] Novas, F. , Ezcurra, M. , & Lecuona, a. (2008). *Orkoraptor burkei* nov. gen. Et sp., a large theropod from the Maastrichtian Pari Aike formation, southern Patagonia, Argentina. Cretaceous Research, 29, 468–480.

[ar70085-bib-0116] Novas, F. E. (1997). Anatomy of *Patagonykus puertai* (Theropoda, Avialae, Alvarezsauridae), from the late cretaceous of Patagonia. Journal of Vertebrate Paleontology, 17(1), 137–166.

[ar70085-bib-0117] Novas, F. E. , Agnolín, F. L. , Ezcurra, M. D. , Porfiri, J. , & Canale, J. I. (2013). Evolution of the carnivorous dinosaurs during the cretaceous: The evidence from Patagonia. Cretaceous Research, 45, 174–215.

[ar70085-bib-0118] Novas, F. E. , Agnolín, F. L. , Motta, M. J. , & Brissón Egli, F. (2021). Osteology of *Unenlagia comahuensis* (Theropoda, Paraves, Unenlagiidae) from the late cretaceous of Patagonia. Anatomical Record, 304, 2741–2788. 10.1002/ar.24641 33894102

[ar70085-bib-0119] Novas, F. E. , Ezcurra, M. D. , Agnolin, F. L. , Pol, D. , & Ortíz, R. (2012). New Patagonian cretaceous theropod sheds light about the early radiation of Coelurosauria. Revista del Museo Argentino de Ciencias Naturales, 14, 57–81.

[ar70085-bib-0120] O'Connor, J. K. (2025). Insights into the early evolution of modern avian physiology from fossilized soft tissues from the Mesozoic. Philosophical Transactions of the Royal Society B: Biological Sciences, 380(1920), 20230426. 10.1098/rstb.2023.0426 PMC11864835 40010392

[ar70085-bib-0121] O'Connor, J. K. , Clark, A. , Kuo, P.‐C. , Kiat, Y. , Fabbri, M. , Shinya, A. , Van Beek, C. , Lu, J. , Wang, M. , & Hu, H. (2025). Chicago *archaeopteryx* informs on the early evolution of the avian bauplan. Nature, 641(8065), 1201–1207. 40369075 10.1038/s41586-025-08912-4

[ar70085-bib-0122] Osborn, H. F. (1906). Upper cretaceous carnivorous dinosaur (second communication). Bulletin of the American Museum of Natural History, 22, 281–296.

[ar70085-bib-0123] Osmόlska, H. , Roniewicz, E. , & Barsbold, R. (1972). A new dinosaur, *Gallimimus bullatus* n. gen., n. sp. (Ornithomimidae) from the upper cretaceous of Mongolia. Acta Palaeontologica Polonica, 27, 103–195.

[ar70085-bib-0124] Ostrom, J. H. (1969). Osteology of Deinonychus antirrhopus, an unusual theropod from the lower cretaceous of Montana. Peabody Museum of Natural History, Yale University, 1–165 pp.

[ar70085-bib-0125] Ostrom, J. H. (1978). The osteology of *Compsognathus longipes* Wagner. Zitteliana, 4, 73–118.

[ar70085-bib-0126] Pei, R. , Pittman, M. , Goloboff, P. A. , Dececchi, T. A. , Habib, M. B. , Kaye, T. G. , Larsson, H. C. E. , Norell, M. A. , Brusatte, S. L. , & Xu, X. (2020). Potential for powered flight neared by most close avialan relatives, but few crossed its thresholds. Current Biology, 30(20), 4033–4046. 32763170 10.1016/j.cub.2020.06.105

[ar70085-bib-0127] Peyer, K. (2006). A reconsideration of *Compsognathus* from the upper Tithonian of Canjuers, southeastern France. Journal of Vertebrate Paleontology, 26, 879–896.

[ar70085-bib-0128] Pierossi, F. F. , Delcourt, R. , Casali, D. d. M. , Leme, J. A. , de Oliveira Martins, N. , Manzig, P. , & Langer, M. C. (2025). Convergent evolution among non‐carnivorous, desert‐dwelling theropods as revealed by the dentary of the noasaurid *Berthasaura leopoldinae* (cretaceous of Brazil). Palaeontology, 68(4), e70014. 10.1111/pala.70014

[ar70085-bib-0129] Pol, D. , & Goloboff, P. A. (2020). Chapter 3: The impact of unstable taxa in coelurosaurian phylogeny and resampling support measures for parsimony analyses. In Bulletin of the American Museum of Natural History(Vol. 440), 97–116. Bulletin of the AMNH.

[ar70085-bib-0130] Pol, D. , & Rauhut, O. W. M. (2012). A middle Jurassic abelisaurid from Patagonia and the early diversification of theropod dinosaurs. Proceedings of the Royal Society B: Biological Sciences, 279, 3170–3175. 10.1098/rspb.2012.0660 PMC3385738 22628475

[ar70085-bib-0131] Porfiri, J. D. , Novas, F. E. , Calvo, J. O. , Agnolín, F. L. , Ezcurra, M. D. , & Cerda, I. A. (2014). Juvenile specimen of *Megaraptor* (Dinosauria, Theropoda) sheds light about tyrannosauroid radiation. Cretaceous Research, 51, 35–55.

[ar70085-bib-0186] Porfiri, J. D. , Valieri, R. D. J. , Santos, D. D. , & Lamanna, M. C. (2018). A new megaraptoran theropod dinosaur from the upper cretaceous Bajo de la Carpa formation of northwestern Patagonia. Cretaceous Research, 89, 302–319.

[ar70085-bib-0191] Pradelli, L. A. , Pol, D. , & Ezcurra, M. D. (2025). The appendicular osteology of the early Jurassic theropod Piatnitzkysaurus floresi and its implications on the morphological disparity of non‐coelurosaurian tetanurans. Zoological Journal of the Linnean Society, 203(1), zlae176.

[ar70085-bib-0177] Price, L. I. (1959). Sobre um Crocodilídeo Notossuquio do Cretáceo Brasileiro (Vol. 188, pp. 1–55). Boletim do Departamento Nacional de Produção Mineral/Divisão de Geologia e Mineralogia.

[ar70085-bib-0132] Pu, H. , Kobayashi, Y. , Lü, J. , Xu, L. , Wu, Y. , Chang, H. , Zhang, J. , & Jia, S. (2013). An unusual basal therizinosaur dinosaur with an ornithischian dental arrangement from northeastern China. PLoS One, 8, e63423. 23734177 10.1371/journal.pone.0063423 PMC3667168

[ar70085-bib-0133] Qiu, R. , Wang, X. , Jiang, S. , Meng, J. , & Zhou, Z. (2025). Two new compsognathid‐like theropods show diversified predation strategies in theropod dinosaurs. National Science Review, 12, nwaf068. 40191255 10.1093/nsr/nwaf068 PMC11970238

[ar70085-bib-0134] Raath, M. A. (1977). The anatomy of the Triassic theropod *Syntarsus rhodesiensis* (Saurischia: Podokesauridae) and a consideration of its biology.

[ar70085-bib-0135] Rauhut, O. W. M. (2003). The interrelationships and evolution of basal theropod dinosaurs. Special Papers in Palaeontology, 69, 1–213.

[ar70085-bib-0136] Rauhut, O. W. M. , & Carrano, M. T. (2016). The theropod dinosaur *Elaphrosaurus bambergi* Janensch, 1920, from the late Jurassic of Tendaguru, Tanzania. Zoological Journal of the Linnean Society, 178, 546–610.

[ar70085-bib-0137] Rauhut, O. W. M. , Foth, C. , Tischlinger, H. , & Norell, M. a. (2012). Exceptionally preserved juvenile megalosauroid theropod dinosaur with filamentous integument from the late Jurassic of Germany. Proceedings of the National Academy of Sciences of the United States of America, 109, 11746–11751. 22753486 10.1073/pnas.1203238109 PMC3406838

[ar70085-bib-0138] Rauhut, O. W. M. , & Pol, D. (2021). New theropod remains from the late Jurassic Cañadón Calcáreo formation of Chubut, Argentina. Journal of South American Earth Sciences, 111, 103434.

[ar70085-bib-0139] Rhodes, M. M. , Henderson, D. M. , & Currie, P. J. (2021). Maniraptoran pelvic musculature highlights evolutionary patterns in theropod locomotion on the line to birds. PeerJ, 9, e10855. 33717681 10.7717/peerj.10855 PMC7937347

[ar70085-bib-0140] Rich, T. H. , & Vickers‐Rich, P. (1994). Neoceratopsians and ornithomimosaurs: Dinosaurs of Gondwana origin. National Geographic Research and Exploration, 10(1), 129–131.

[ar70085-bib-0187] Rolando, A. M. A. , Novas, F. E. , & Agnolín, F. L. (2019). A reanalysis of Murusraptor barrosaensis Coria & Currie (2016) affords new evidence about the phylogenetical relationships of Megaraptora. Cretaceous Research, 99, 104–127.

[ar70085-bib-0141] Russell, D. A. , & Dong, Z. M. (1993). A nearly complete skeleton of a new troodontid dinosaur from the early cretaceous of the Ordos Basin, Inner Mongolia, People's Republic of China. Canadian Journal of Earth Sciences, 30, 2163–2173.

[ar70085-bib-0142] Sales, M. A. F. , & Schultz, C. L. (2017). Spinosaur taxonomy and evolution of craniodental features: Evidence from Brazil. PLoS One, 12, 1–30. 10.1371/journal.pone.0187070 PMC5673194 29107966

[ar70085-bib-0143] Samathi, A. , Chanthasit, P. , & Sander, P. M. (2019). Two new basal coelurosaurian theropod dinosaurs from the lower cretaceous Sao Khua formation of Thailand. Acta Palaeontologica Polonica, 64, 239–260.

[ar70085-bib-0144] Samathi, A. , Sander, P. M. , & Chanthasit, P. (2021). A spinosaurid from Thailand (Sao Khua formation, early cretaceous) and a reassessment of *Camarillasaurus cirugedae* from the early cretaceous of Spain. Historical Biology, 33, 3480–3494.

[ar70085-bib-0145] Sayão, J. M. , Saraiva, A. Á. F. , Brum, A. S. , Bantim, R. A. M. , de Andrade, R. C. L. P. , Cheng, X. , de Lima, F. J. , de Paula Silva, H. , & Kellner, A. W. A. (2020). The first theropod dinosaur (Coelurosauria, Theropoda) from the base of the Romualdo formation (Albian), Araripe Basin, Northeast Brazil. Scientific Reports, 10, 1–15. 32651406 10.1038/s41598-020-67822-9 PMC7351750

[ar70085-bib-0146] Schade, M. , Rauhut, O. , Foth, C. , Moleman, O. , & Evers, S. (2023). A reappraisal of the cranial and mandibular osteology of the spinosaurid *Irritator challengeri* (Dinosauria: Theropoda). Palaeontologia Electronica, 26(2), a17.

[ar70085-bib-0147] Scheyer, T. M. , Oliveira, G. R. , Romano, P. S. R. , Bastiaans, D. , Falco, L. , Ferreira, G. S. , & Rabi, M. (2023). A forged ‘chimera’ including the second specimen of the protostegid sea turtle *Santanachelys gaffneyi* and shell parts of the pleurodire *Araripemys* from the lower cretaceous Santana group of Brazil. Swiss Journal of Palaeontology, 142, 6. 37163143 10.1186/s13358-023-00271-9 PMC10163108

[ar70085-bib-0148] Senter, P. C. (2007). A new look at the phylogeny of Coelurosauria (Dinosauria: Theropoda). Journal of Systematic Palaeontology, 5, 1–35.

[ar70085-bib-0149] Sereno, P. C. (1999). The evolution of dinosaurs. Science, 284, 2137–2147. 10381873 10.1126/science.284.5423.2137

[ar70085-bib-0150] Sereno, P. C. (2017). Early cretaceous ornithomimosaurs (Dinosauria: Coelurosauria) from Africa. Ameghiniana, 54(5), 576–616.

[ar70085-bib-0152] Sereno, P. C. , Tan, L. , Brusatte, S. L. , Kriegstein, H. J. , Zhao, X. , & Cloward, K. (2009). Tyrannosaurid skeletal design first evolved at small body size. Science (New York, N.Y.), 326, 418–422. 19762599 10.1126/science.1177428

[ar70085-bib-0153] Sereno, P. C. , Wilson, J. a. , Larsson, H. C. , Dutheil, D. B. , & Sues, H. D. (1994). Early cretaceous dinosaurs from the Sahara. Science (New York, N.Y.), 266, 267–271. 17771449 10.1126/science.266.5183.267

[ar70085-bib-0154] Serrano‐Brañas, C. I. , Espinosa‐Chávez, B. , Maccracken, S. A. , Gutiérrez‐Blando, C. , de León‐Dávila, C. , & Ventura, J. F. (2020). *Paraxenisaurus normalensis*, a large deinocheirid ornithomimosaur from the Cerro del Pueblo formation (upper cretaceous), Coahuila, Mexico. Journal of South American Earth Sciences, 101, 102610.

[ar70085-bib-0155] Smith, D. , & Galton, P. (1990). Osteology of *Archaeornithomimus asiaticus* (upper cretaceous, Iren Dabasu formation, People's Republic of China). Journal of Vertebrate Paleontology, 10, 255–265.

[ar70085-bib-0156] Smith, N. D. , Makovicky, P. J. , Hammer, W. R. , & Currie, P. J. (2007). Osteology of *Cryolophosaurus ellioti* (Dinosauria: Theropoda) from the early Jurassic of Antarctica and implications for early theropod evolution. Zoological Journal of the Linnean Society, 151, 377–421.

[ar70085-bib-0157] Spiekman, S. N. F. , Ezcurra, M. D. , Butler, R. J. , Fraser, N. C. , & Maidment, S. C. R. (2021). *Pendraig milnerae*, a new small‐sized coelophysoid theropod from the late Triassic of Wales. Royal Society Open Science, 8, 210915. 34754500 10.1098/rsos.210915 PMC8493203

[ar70085-bib-0158] Sues, H. D. , & Averianov, A. (2016). Ornithomimidae (Dinosauria: Theropoda) from the Bissekty formation (upper cretaceous: Turonian) of Uzbekistan. Cretaceous Research, 57, 90–110.

[ar70085-bib-0159] Sues, H.‐D. , Frey, E. , Martill, D. M. , & Scott, D. M. (2002). *Irritator challengeri*, a Spinosaurid (Dinosauria: Theropoda) from the lower cretaceous of Brazil. Journal of Vertebrate Paleontology, 22, 535–547.

[ar70085-bib-0160] Sullivan, R. M. (1997). A juvenile *Ornithomimus antiquus* (Dinosauria: Theropoda: Ornithomimosauria), from the upper cretaceous Kirtland formation (De‐na‐zin member), San Juan Basin, New Mexico. 48th Field conference, Mesozoic geology and paleontology of the four corners region. pp. 249–254.

[ar70085-bib-0162] Tsuihiji, T. , Barsbold, R. , Watabe, M. , Tsogtbaatar, K. , Chinzorig, T. , Fujiyama, Y. , & Suzuki, S. (2014). An exquisitely preserved troodontid theropod with new information on the palatal structure from the upper cretaceous of Mongolia. Naturwissenschaften, 101, 131–142. 24441791 10.1007/s00114-014-1143-9

[ar70085-bib-0164] von Huene, F. (1914). Das natürliche System der Saurischia. Centralblatt Für Mineralogie, Geologie Und Paläontologie Jahrgang Abteilung (B), 1914, 154–158.

[ar70085-bib-0183] Walker, A. D. (1977). Evolution of the pelvis in birds and dinosaurs. In S. M. Andrews , R. S. Miles , & A. D. Walker (Eds.), Problems in vertebrate evolution. Linnean society symposia, n. 4 (pp. 319–358). Academic Press.

[ar70085-bib-0165] Wang, S. , Stiegler, J. , Amiot, R. , Wang, X. , Du, G.‐H. , Clark, J. M. , & Xu, X. (2017). Extreme ontogenetic changes in a Ceratosaurian theropod. Current Biology, 27, 144–148. 28017609 10.1016/j.cub.2016.10.043

[ar70085-bib-0182] Wilkinson, M. (1994). Common cladistic information and its consensus representation: Reduced Adams and reduced cladistic consensus trees and profiles. Systematic Biology, 43(3), 343–368.

[ar70085-bib-0166] Wilson, J. A. (1999). A nomenclature for vertebral laminae in sauropods and other saurischian dinosaurs. Journal of Vertebrate Paleontology, 19, 639–653.

[ar70085-bib-0167] Wilson, J. A. (2012). New vertebral laminae and patterns of serial variation in vertebral laminae of sauropod dinosaurs. Contributions from the Museum of Paleontology New Vertebral Laminae and Patterns of Serial Variation in Vertebral Laminae of Sauropod Dinosaurs, 32, 91–110.

[ar70085-bib-0168] Wilson, J. A. , D'Emic, M. D. , Ikejiri, T. , Moacdieh, E. M. , & Whitlock, J. A. (2011). A nomenclature for vertebral fossae in sauropods and other saurischian dinosaurs. PLoS One, 6, e17114. 21386963 10.1371/journal.pone.0017114 PMC3046170

[ar70085-bib-0169] Xing, L. , Bell, P. R. , Persons, W. S. , Ji, S. , Miyashita, T. , Burns, M. E. , Ji, Q. , & Currie, P. J. (2012). Abdominal contents from two large early cretaceous compsognathids (Dinosauria: Theropoda) demonstrate feeding on confuciusornithids and dromaeosaurids. PLoS One, 7, e44012. 22952855 10.1371/journal.pone.0044012 PMC3430630

[ar70085-bib-0170] Xu, L. , Kobayashi, Y. , Lü, J. , Lee, Y.‐N. , Liu, Y. , Tanaka, K. , Zhang, X. , Jia, S. , & Zhang, J. (2011). A new ornithomimid dinosaur with north American affinities from the late cretaceous Qiupa formation in Henan Province of China. Cretaceous Research, 32, 213–222.

[ar70085-bib-0171] Xu, X. , Clark, J. M. , Forster, C. A. , Norell, M. A. , Erickson, G. M. , Eberth, D. A. , Jia, C. , & Zhao, Q. (2006). A basal tyrannosauroid dinosaur from the late Jurassic of China. Nature, 439, 715–718. 16467836 10.1038/nature04511

[ar70085-bib-0172] Xu, X. , Clark, J. M. , Mo, J. , Choiniere, J. , a Forster, C. , Erickson, G. M. , Hone, D. W. E. , Sullivan, C. , Eberth, D. A. , Nesbitt, S. , Zhao, Q. , Hernandez, R. , Jia, C. , Han, F. , & Guo, Y. (2009). A Jurassic ceratosaur from China helps clarify avian digital homologies. Nature, 459, 940–944. 19536256 10.1038/nature08124

[ar70085-bib-0173] Yao, X. , Sullivan, C. , Tan, Q. , & Xu, X. (2022). New ornithomimosaurian (Dinosauria: Theropoda) pelvis from the upper cretaceous Erlian formation of Nei Mongol, North China. Cretaceous Research, 137, 105234.

[ar70085-bib-0174] Zanno, L. E. (2010). A taxonomic and phylogenetic re‐evaluation of Therizinosauria (Dinosauria: Maniraptora). Journal of Systematic Palaeontology, 8, 503–543.

[ar70085-bib-0175] Zanno, L. E. , Gillette, D. D. , Albright, L. B. , & Titus, A. L. (2009). A new North American therizinosaurid and the role of herbivory in “predatory” dinosaur evolution. Proceedings of the Royal Society B: Biological Sciences, 276, 3505–3511. 10.1098/rspb.2009.1029 PMC2817200 19605396

[ar70085-bib-0176] Zhong, Y. , Wang, Y. , Jia, B. , Wang, M. , Hu, L. , & Pan, Y. (2021). A potential terrestrial Albian–Cenomanian boundary in the Yanji Basin, Northeast China. Palaeogeography, Palaeoclimatology, Palaeoecology, 562, 110088.

