# Supplementary material for: The coelurosaur theropods of the Romualdo formation, early Cretaceous (Aptian) of Brazil: Santanaraptor placidus meets Mirischia asymmetrica
Source: Anat Rec (Hoboken). 2025 Nov 18;309(10):2752–89. doi: 10.1002/ar.70085 (PMC13549251; doi:10.1002/ar.70085)
Supplement: Supplementary file 1 [file AR-309-2752-s001.doc]

Supplementary Materials for

**The coelurosaur theropods of the Romualdo Formation, Early Cretaceous (Aptian) of Brazil: *Santanaraptor placidus* meets *Mirischia asymmetrica***

**Rafael Delcourt^1*^, Orlando Nelson Grillo^2^, Christophe Hendrickx^3,4^, Maximilian Kellermann^5,6^, Max Cardoso Langer^1^**

^1^ Laboratório de Paleontologia de Ribeirão Preto, FFCLRP, Universidade de São Paulo, Ribeirão Preto, Brazil

^2^ Laboratório de Processamento de Imagem Digital, Departamento de Geologia e Paleontologia, Museu Nacional, Universidade Federal do Rio de Janeiro, Rio de Janeiro, Brazil

^3^ Dinosauria Lab, Fundación Miguel Lillo, Miguel Lillo 251, San Miguel de Tucumán 4000, Tucumán, Argentina

^4^ Unidad Ejecutora Lillo, CONICET-Fundación Miguel Lillo, Miguel Lillo 251, San Miguel de Tucumán 4000, Tucumán, Argentina

^5^ Staatliche Naturwissenschaftliche Sammlungen Bayerns–Bayerische Staatssammlung für Paläontologie und Geologie, Munich, Germany

^6^ Department für Geo- und Umweltwissenschaften, Sektion Paläontologie und Geobiologie, Ludwig-Maximilians-Universität, Munich, Germany

* rafael.delcourt@gmail.com

1– List of characters modified from Rauhut & Pol (2021) - “Mesozoic Tetrapod Work Group” (MTWG)

2 – Complete topology recovered form MTWG data matrix using equal weight characters

3 – Complete topology recovered form MTWG data matrix using implied weight characters k =16

4 – List of synapomorphies

5 – Figure S1. *Mirischia asymmetrica* (SMNK 2349) - Vertebral series.

6 – Figure S2. *Mirischia asymmetrica* (SMNK 2349) - Details of the pelvic girdle.

1- List of characters modified from Rauhut & Pol (2021)

**Bold characters mark ontogenetic variable characters**

# Cranium

## Skull

1. **Skull, relative length in comparison to femoral length in subadult or adult individuals: (0) 75% or less; (1) between 75% and 110%; (2) 110 % or more ORDERED (modified from Rauhut & Pol 2021: C4)**
2. **Skull, relative length of preorbital region, compared to postorbital region of the skull in sub-adults or adults: (0) short, less than 170% of postorbital length; (1) moderately long, between 170% and 310%; (2) long, more than 310 % ORDERED (modified from Rauhut & Pol 2021: C196)**
3. Skull, angle between ventral margin of the preorbital part of the skull (as measured in straight line from the end of the tooth row [or the antorbital part of the maxilla] to the anteroventral tip of the premaxilla) and the ventral margin of the posterior part of the skull (as measured from the aforementioned point to the posteroventral margin of the quadratojugal): (0) less than -5° (postalveolar part is upturned); (1) between -5° and 20°; (2) between 20° and 35°; (3) more than 35° ORDERED (new)
4. External nares, relative length compared to skull length (the latter measured from the anterior tip of the premaxilla to the posterior end of the squamosal): (0) less than 14%; (1) 14% - 23%; (2) more than 23% (modified from Rauhut & Pol 2021: C85)
5. External nares, posterior margin, position relative to anterior border of antorbital fossa: (0) anterior to it, or completely anterior to the maxilla in cases where the antorbital fossa is absent; (1) reaching or overlapping the anterior border of the antorbital fossa (Rauhut & Pol 2021: C15)
6. Antorbital fenestra, composition of dorsal border in lateral view: (0) formed by lacrimal and maxilla; (1) formed by nasal and lacrimal (modified from Rauhut & Pol 2021: C 47)
7. Skull anteroposterior width of postorbital bar (measured at about mid-height of the orbit, excluding the anterior expansion into the orbit of the jugal process of the postorbital in taxa such as abelisaurids) compared to minimum width of preorbital bar: (0) subequal; (1) expanded, greater than twice width (modified from Rauhut & Pol 2021: C98)
8. **Orbit, shape in subadult to adult individuals: (0) sub-circular in lateral or dorsolateral view, or wider than high; (1) dorsoventrally long (slightly modified from Rauhut & Pol 2021: C157)**
9. **Orbit, ventral margin: (0) concave or straight; (1) tapering, V-shaped (new)**
10. **Infratemporal fenestra, relative size (measured as the ratio of the maximum length of this fenestra in its ventral half against the maximal length of the orbit) [Only applicable in adult or subadult individuals]: (0) less than 0.25; (1) between 0.25 and 1; (2) between 1 and 1.4; (3) greater than 1.4 ORDERED (modified from Rauhut & Pol 2021: C194)**
11. Infratemporal fenestra shape: (0) wider ventrally than dorsally; (1) of subequal width dorsally and ventrally; (2) wider dorsally than ventrally ORDERED (modified from Rauhut & Pol 2021: C167)
12. Infratemporal fenestra, posterior margin, shape: (0) convex, forming a small to moderate posterior constriction of the infratemporal fenestra; (1) straight or only very slightly convex; (2) with marked expansion of the squamosal and quadratojugal into the infratemporal fenestra, formed by the almost entirely anteriorly directed quadratojugal process of the squamosal and the expanded dorsal end of the quadratojugal (modified from Rauhut & Pol 2021: C167)
13. Infratemporal fenestra, subdivided, or almost subdivided by contact between the jugal/postorbital bar and the quadrato-jugal/squamosal bar: (0) absent; (1) present (new)
14. **Skull, location of mandibular joint: (0) significantly posterior to quadrate head; (1) approximately straight below quadrate head; (2) significantly anterior to quadrate head ORDERED (Rauhut & Pol 2021: C178)**

### Premaxilla

15. **Premaxillae, fusion: (0) separate; (1) fused (Rauhut & Pol 2021: C6)**
16. Premaxilla, anterior margin, angle between anterior margin (measured at base of nasal process) and alveolar margin of premaxilla: (0) more than 90 degrees (nasal process slightly overhangs anterior margin of premaxilla); (1) equal or more than 70 degrees; (2) less than 70 degrees; (3) equal or less than 40 degrees ORDERED (Rauhut & Pol 2021: C10)
17. Premaxilla, main body, height/length ratio ventral to external naris (length measured along ventral border of bone): (0) < 0.5 (0); (1) 0.5-0.99; (2) 1-1.49, (3) 1.5 or higher ORDERED (Rauhut & Pol 2021: C 5)
18. Premaxillary articulated elements, shape in dorsal view: (0) angular, V-shaped; (1) rounded, U-shaped (Rauhut & Pol 2021: C16)
19. Premaxillae, premaxilla-nasal suture, shape in dorsal view: (0) v-shaped; (1) w-shaped (Rauhut & Pol 2021: C7)
20. Premaxilla, external nares, location of anterior margin relative to premaxillary body: (0) over anterior half of ventral margin of premaxillary body; (1) over the posterior half of the ventral margin of the premaxillary body; (2) close to (behind last premaxillary tooth) or posterior to the ventral margin of the premaxillary body ORDERED (Rauhut & Pol 2021: C9)
21. **Premaxilla, narial fossa: (0) absent, anteroventral margin of the external nares forms a sharp rim; (1) present, excavation of the anteroventral margin of the nares between premaxillary body and nasal process (modified from Rauhut & Pol 2021: C 23)**
22. Premaxilla, narial fossa, lateral step extending ventrally from anterior end of narial fossa: (0) absent; (1) present (modified from Rauhut & Pol 2021: C 18)
23. Premaxilla, large neurovascular foramen at the base of the nasal process: (0) absent; (1) present (modified from Rauhut & Pol 2021: C 26)
24. **Premaxilla, nasal process, posterior extent relative to posterior tip of subnarial process: (0) considerably more anterior; (1) subequal; (2) posterior ORDERED (modified from Rauhut et al. 2016; modified from Wang et al., 2017: C26; modified from Rauhut & Pol 2021: C27)**
25. Premaxilla, nasal process: (0) does not contact frontal; (1) contacts frontal (modified from Turner et al., 2012)
26. Premaxilla, contribution to midline crest on nasals: (0) absent; (1) present (Rauhut & Pol 2021: C 28)
27. Premaxilla, ventral margin, shape: (0) straight; (1) strongly concave, anterior end downturned (new)
28. Premaxilla, buccal edge, shape [inapplicable for taxa without a rhamphotheca in the premaxilla]: (0) straight; (1) crenulate (Rauhut & Pol 2021: C14)
29. Premaxilla, enlarged foramen above second premaxillary tooth: (0) absent; (1) present (Rauhut & Pol 2021: C 19)
30. Premaxilla, ventral margin, alignment compared to ventral margin of maxilla (posterior part of the maxilla in taxa that show an upturned anterior maxillary ventral margin): (0) not downturned in its entirety; (1) downturned; (2) upturned (modified from Rauhut & Pol 2021: C 21)
31. Premaxilla, ventral margin, transition of alveolar margin into maxillary articular facet: (0) marked, angle more than 45°; (1) gradual transition, angle less than 45° (modified from Rauhut & Pol 2021: ch. 20)
32. Premaxilla, suture with the maxilla, lateral view: (0) planar; (1) with small notch in the posterior margin of the premaxillary body for reception of the tapering anterior end of the maxilla (modified from Rauhut & Pol 2021: C8)
33. Premaxilla, subnarial foramen: (0) present; (1) absent (modified from Choiniere et al. 2010; modified from Wang et al., 2017: C24; modified from Rauhut & Pol 2021: C25)
34. Premaxilla, posterodorsal process (maxillary process, subnarial process): (0) wide, platelike; (1) thin, rodlike; (2) short, triangular tubercle (Rauhut & Pol 2021: C 22)
35. Premaxilla, maxillary process, size: (0) small, maxilla participates broadly in external naris; (1) moderate, contacts nasal, maxilla does not participate in external nares; (2) large, partially separates maxilla from nasal posterior to external nares; (3) extensive, contacts lacrimal, completely separates maxilla from nasal ORDERED (Rauhut & Pol 2021: C12)
36. Premaxilla, suture with the maxilla, posteromedial palatal process: (0) absent; (1) present (modified from Rauhut & Pol 2021: C11)
37. Premaxilla-maxillary contact, diastema [inapplicable for taxa that don’t have maxillary teeth]: (0) absent, no space between the premaxillary and maxillary tooth rows at the premaxillary/maxillary suture; (1) present, substantial diastema of at least one crowns mesiodistal length between maxillary and premaxillary teeth on the posterior end of the premaxilla (Rauhut & Pol 2021: C 29)

### Maxilla

38. Maxillae, orientation of the maxillae towards each other, dorsal view: (0) acutely angled; (1) subparallel (Rauhut & Pol 2021: C 42)
39. **Maxilla, alveolar (or rhamphothecal) margin, shape in lateral view: (0) ventrally flat; (1) ventrally convex over its entire length; (2) sinuous, anteriorly convex, posteriorly concave (modified from Rauhut & Pol 2021: C 63)**
40. Maxilla, ascending process and jugal ramus, posterior extent: (0) jugal ramus extends considerably further posteriorly than ascending process; (1) subequal in length or ascending process longer (new)
41. **Maxilla, main body, extensive grooved sculpturing of external surface in mature individuals: (0) absent; (1) well-developed, covering most of lateral surface (modified from Rauhut & Pol , 2021: C72)**
42. Maxilla, nutrient foramina on lateral surface, arrangement: (0) single row or no distinct pattern; (1) a second, more dorsally placed row is present anteriorly and converges with the ventral row posteriorly; (2) a second, dorsally placed row is present and extends posteriorly more or less parallel to the ventral row (modified from Rauhut & Pol 2021: C70; see Rauhut & Pol 2019)
43. Maxilla-premaxillary contact, constriction between articulated premaxillae and maxillae in dorsal view: (0) absent; (1) present (Rauhut & Pol 2021: C 56)
44. Maxilla-premaxillary contact, subnarial gap at the alveolar margin: (0) absent; (1) present (Rauhut & Pol 2021: C 57)
45. Maxilla, anterior ramus: (0) absent, anterior margin of maxillary body and ascending process continuously convexly sloping posterodorsally; (1) present, ascending process offset from anterior rim of maxillary body by a markedly concave area (modified from Rauhut & Pol 2021: C 43)
46. Maxilla, anterior ramus, relative length(ratio measured from the anterior rim of the antorbital fossa to anterior end of the maxillary body divided by the height of the maxillary body at mid-length of the latter measurement, as a proxy for the length of the anterior ramus): (0) very short (length/height ratio less than 1); (1) moderately long (1-1.35); (2) long (1.35-1.7); (3) very long (more than 2) ORDERED (modified from Rauhut & Pol 2021: C 44; not applicable for taxa that lack an anterior ramus [coded 0 for character 43])
47. Maxilla, anterior ramus, subnarial fossa in dorsolateral position: (0) absent; (1) present (modified from Rauhut & Pol 2021: C 66)
48. **Maxilla, ascending process, inclination of the anterior margin, as measured at the half height of the ascending process towards the ventral maxillary margin (or the line connecting the anterior end of the first to the posterior end of the last alveolus of the maxilla in taxa with convex or sinuous ventral margins): (0) steeply angled, angle > 50°; (1) moderately inclined posteriorly, angle between 50° and 20°; (2) very steeply inclined posteriorly, angle < 20° ORDERED (modified from Rauhut & Pol 2021: C 69)**
49. Maxilla, ascending process, shape of anterodorsal margin in lateral view: (0) straight or gently curved; (1) with pronounced kink at about mid-length, with a more anteriorly facing margin ventral and a more dorsally facing margin dorsal to the kink. (modified from Rauhut & Pol 2021: C 67 according to Rauhut & Pol, 2019)
50. Maxilla, ascending process, exposure of lateral lamina: (0) large, broad exposure, forming the anterior and anterodorsal rim of the antorbital fossa; (1) small triangular exposure at (the anterior rim of the antorbital fossa only (modified from Rauhut & Pol 2021: C 49)
51. Maxilla, ascending process, anteroventral end of facet for nasal articulation: (0) flat or shallow groove on the anterolateral surface; (1) socket, bordered anteroventrally by a projection of the maxilla and visible in lateral view (modified from Rauhut & Pol 2021: C 65)
52. Maxilla, ascending process, pneumatic fossa (excavatio pneumatica) on the lateral surface: (0) absent; (1) present (Rauhut & Pol 2021: C78)
53. Maxilla, ascending process, posterior end: (0) forked to receive an anterior process of the lacrimal; (1) tapering and overlying the anterior process of the lacrimal laterally (new)
54. Maxilla, antorbital fossa, anteroposterior length in front of the internal antorbital fenestra relative to length of the external antorbital fenestra: (0) 40% or less; (1) more than 40% (Rauhut & Pol 2021: C 50)
55. Maxilla, antorbital fossa, anterior margin, shape: (0) rounded or pointed; (1) square (modified from Rauhut & Pol 2021: C 46)
56. Maxilla, antorbital fossa, anterior part, oblique ridge in the ventral part: (0) absent; (1) present (Rauhut et al. 2020)
57. Maxilla, antorbital fossa, dorsal extent: (0) expands dorsally over entire height of ascending process; (1) bordered dorsally by rim on ascending process of maxilla (modified from Rauhut & Pol 2021: C 48)
58. Maxilla, antorbital fossa, texture of lateral side of medial wall: (0) smooth; (1) with notable pits (new)
59. Maxilla, promaxillary foramen (Also called fenestra promaxillaris, tertiary fenestra of the maxilla, or promaxillary fenestra): (0) absent; (1) present, developed as a shallow fossa; (2) present as an opening leading anteriorly into promaxillary antrum ORDERED (modified from Rauhut & Pol 2021: C36)
60. Maxilla, promaxillary foramen: (0) covered in lateral view by a lamina covering the anteroventral corner of the antorbital fossa; (1) exposed in lateral view (modified from Rauhut & Pol 2021: C71)
61. Maxilla, promaxillary antrum, development: (0) small bulla extending into the ascending process; (1) extends into the base of the ascending process as an elongate canal; (2) extends into the base of the ascending process as a mediolaterally inflated pocket (modified from Rauhut & Pol 2021: C36)
62. Maxilla, antorbital fossa, anterior part of medial wall: (0) lacks depressions or foramina; (1) with a large depression without well-defined margins; (2) with a sharply rimmed maxillary fenestra (Rauhut & Pol 2021: C 30)
63. Maxilla, maxillary fenestra, development: (0) opens medially into a small maxillary antrum with a robust medial wall; (1) opens medially into a large maxillary antrum without or an extremely thin medial wall (modified from Rauhut & Pol 2021: C 31)
64. Maxilla, maxillary fenestra (or depression for taxa without a sharply rimmed fenestra), anteroposterior position: (0) situated at anterior border of antorbital fossa; (1) situated posterior to anterior border of antorbital fossa (Rauhut & Pol 2021: C 33)
65. Maxilla, antorbital fossa, maxillary fenestra recessed within a shallow, posteriorly, or posterodorsally open fossa: (0) absent, the maxillary fenestra is not perforated within a subsidiary recess; (1) present (Rauhut & Pol 2021: C 32)
66. Maxilla, antorbital fenestra, maxillary fenestra and promaxillary foramen, alignment: (0) ± aligned, ventral margins of all openings more or less on the same level; (1) maxillary fenestra displaced dorsally in respect to promaxillary foramen and antorbital fenestra, ventral margin of this fenestra distinctly dorsal to ventral margin of the other openings; (2) maxillary fenestra displaced dorsally, ventral margin entirely dorsal to promaxillary foramen ORDERED (modified from Rauhut & Pol 2021: C 34)
67. Maxilla, antorbital fossa, medial wall of the postantral strut extended posteriorly and visible behind the antorbital fossa in lateral view: (0) absent; (1) present (new)
68. Maxilla, medial side, pneumatic region posteroventral to maxillary fenestra: (0) absent; (1) present (Rauhut & Pol 2021: C 52; Wang et al. 2017: C49)
69. Maxilla, interfenestral bar, foramen on caudal edge: (0) absent; (1) present, pierces ventral portion of bar (Rauhut & Pol 2021: C 35)
70. Maxilla, antorbital fossa, ventral extent (depth) at the half length of the antorbital fenestra (measured from the rim of the antorbital fenestra to the highest point of the ridge marking its border): (0) absent or small, less than 1/3 the height of the maxillary body at this level; (1) moderate, between 1/3 and half the height; (2) dorsoventrally deep, more than half the height ORDERED (Rauhut & Pol 2021: C 51)
71. Maxilla, antorbital fossa, development of ventral border: (0) distinct ridge in at least the jugal ramus of the maxilla; (1) only dorsally defined step (Rauhut & Pol 2021: C 53)
72. Maxilla, antorbital fossa, development of horizontal ridge at the ventral border [inapplicable in taxa that do not have a ridge]: (0) both dorsally and ventrally clearly defined, rising abruptly from the lateral maxillary surface; (1) only ventrally defined and dorsally rising gradually from the surface of the maxilla, or only developed as a change in inclination of the lateral surface (Rauhut & Pol 2021: C 54)
73. Maxilla, antorbital fossa, extent of horizontal ridge at the ventral border [inapplicable in taxa that do not have a ridge]: (0) extending from the anteroventral corner of the antorbital fossa posteriorly; (1) only expressed strongly posteriorly on the jugal ramus, the anteroventral border is developed as a step (Rauhut & Pol 2021: C 55)
74. Maxilla, anteromedial process, morphology: (0) ridged or fluted prong; (1) blunt, simple ridge or tubercle; (2) absent (modified from Rauhut & Pol 2021: C75)
75. Maxilla, anteromedial process, anterior extent: (0) extends anteriorly beyond the maxillary body; (1) does not extend beyond the maxillary body anteriorly (modified from Rauhut & Pol 2021: C75)
76. Maxilla, anteromedial process, position of the base of the process: (0) ventral, immediately dorsal to paradental plates; (1) dorsal, immediately ventral to dorsal surface of maxillary anterior ramus; (2) occupies the entire space between the dorsal margin of the interdental plates and the dorsal rim of the anterior maxillary body (modified from Rauhut & Pol 2021: C76)
77. Maxilla, palatal shelf extending posteriorly from the anteromedial (palatal) process of the maxilla: (0) absent; (1) present, developed as a medial swelling (lingual bar); (2) present, developed as a sharply defined shelf ORDERED (modified from Rauhut & Pol 2021: C 37)
78. Maxilla, palatal shelf, extent: (0) ends or becomes inconspicuous anterior or at the anterior end of the antorbital fenestra; (1) extends posteriorly below the antorbital fenestra (Rauhut & Pol 2021: C 38)
79. Maxilla, palatal shelf: (0) flat; (1) with midline ventral "tooth-like" projection (Rauhut & Pol 2021: C 39)
80. Maxilla, anteriormost alveolus, orientation: (0) vertical; (1) angled anteriorly (Rauhut & Pol 2021: C74)
81. **Maxilla and dentary, paradental (interdental) plates: (0) unfused; (1) fused (modified from Rauhut & Pol 2021: C 58)**
82. Maxilla, paradental (interdental) plates, medial surface: (0) smooth or finely pitted; (1) dorsoventrally striated (Rauhut & Pol 2021: C 59)
83. Maxilla, paradental lamina, inclination of anterior part: (0) inclined anterodorsally; (1) horizontal; (2) inclined anteroventrally ORDERED (modified from Rauhut & Pol 2021: C 61)
84. Maxilla and dentary, paradental (interdental) plates, visibility in medial view: (0) widely exposed; (1) obscured by an expanded paradental lamina, triangular apices only may be visible (Carrano et al. 2012, as modified by Rauhut and Pol 2019)
85. Maxilla, paradental (interdental) plates, shape of ventral margin [inapplicable for taxa with unfused paradental plates]: (0) serrated, lower between the alveoli than at the alveoli; (1) largely straight (modified from Hendrickx & Mateus 2014)
86. Maxilla, paradental (interdental) plates, ventral extent: (0) to the same ventral level as lateral maxillary wall; (1) dorsal to ventral level of maxillary wall (Rauhut & Pol 2021: C 60)
87. Maxilla, paradental (interdental) plates, depth of largest plate relative to its anteroposterior width: (0) low, < 1; (1) moderate, between 1 and 1.6; (2) tall > 1.6 (modify from Rauhut & Pol 2021: C 62)
88. Maxilla, jugal ramus, ventrolateral margin posterior to ascending process: (0) flat or convex as it grades onto tooth row; (1) developed as a sharp, ventrolaterally-projecting ridge (Rauhut & Pol 2021: C 40)
89. Maxilla, jugal ramus, prominent subhorizontal neurovascular groove extending posteriorly from a foramen: (0) absent; (1) present (modified from Rauhut & Pol 2021: C73)
90. Maxilla, jugal ramus, posterior end: single (0); forked (1) (new)
91. Maxilla, jugal ramus, morphology of posterior end: (0) straight; (1) with markedly downturned, toothless posterior end (new character)
92. Maxillary-jugal contact, anteroposterior length relative to total maxilla length: (0) less than 25%; (1) between 25% and 40%; (2) more than 40% ORDERED (modified from Rauhut & Pol 2021: C 64)
93. Maxilla, jugal ramus, morphology of jugal contact: (0) dorsally facing groove in the anterior part, laterally facing shelf posteriorly; (1) shelf only, groove absent (modified from Rauhut & Pol 2021: C91)

### Lacrimal

94. Lacrimal, anterior process, length compared to dorsoventral height of ventral process (excluding horns, crests, or expansions): (0) much shorter than ventral process or absent; (1) subequal or longer than ventral process (modified from Rauhut & Pol 2021: C117)
95. Lacrimal, anterior and ventral process, relative angle between processes (as measured along the midlines of the processes): (0) > 60°; (1) < 60° (modified from Carrano et al. 2012: C49)
96. **Lacrimal, ventral process, orientation: (0) strongly sloping anteroventrally; (1) subvertical; (2) sloping posteroventrally ORDERED (Rauhut & Pol 2021: C125)**
97. **Lacrimal, supraantorbital crest on the lacrimal ("Lacrimal horn"): (0) absent; (1) small dorsal rugosity; (2) low, broad, curved brow; (3) large triangular horn ORDERED (Rauhut & Pol 2021: C119)**
98. **Lacrimal, dorsal extent of the lacrimal above the ventral process (excluding lacrimal horns or projections): (0) significantly less than height of the orbit, dorsal rim of orbit considerably above lacrimal; (1) as high as the orbit, at about the same level of the dorsal margin of the orbit (new)**
99. Lacrimal, posterodorsal process: (0) absent; (1) present, much shorter than anterior ramus; (2) present, subequal in length to anterior ramus ORDERED (Rauhut & Pol 2021: C128)
100. Lacrimal, posterodorsal process, direction: (0) projects posteriorly; (1) projects posterodorsally or completely dorsally (Rauhut & Pol 2021: C129)
101. Lacrimal, lateral bulge of the lacrimal/prefrontal at the anterodorsal border of the orbit, best visible in dorsal view: (0) absent; (1) present (modified from Rauhut & Pol 2021: C130)
102. Lacrimal, lateral bulge of the lacrimal/prefrontal at the anterodorsal margin of the orbit: (0) confluent with lateral surface of ventral process; (1) forms an overhanging shelf above ventral process. Not applicable for taxa that do not have the lateral bulge
103. **Lacrimal, lateral lamina, development: (0) continuous between the anterior and ventral process; (1) deeply invaginated in the lacrimal angle, lamina ventral to the angle extends further anteriorly than in the lacrimal angle (modified from Rauhut & Pol 2021: C116)**
104. Lacrimal, lateral shelf on the dorsal end of the lacrimal where the anterior and ventral processes of the lacrimal meet: (0) absent; (1) present (Rauhut & Pol 2021: C120)
105. Lacrimal, large pneumatic recess at the angle of the lacrimal on the lateral side ("lacrimal fenestra"): (0) absent; (1) present (modified from Rauhut & Pol 2021: C121)
106. Lacrimal, development of lacrimal recess: (0) largely exposed laterally, opening of the overhanging lamina is above the posterior half of the ventral process; (1) developed as an anterolaterally opening pocket with the anterior rim of the lateral lamina being placed at about the anterior margin of the ventral process; (2) completely covered laterally by a lamina and opening anteriorly or anteromedially (modified from Rauhut & Pol 2021: C123)
107. Lacrimal, medial/anterior lacrimal recess at the dorsal end of the ventral process: (0) absent; (1) present (Rauhut & Pol 2021: C118)
108. Lacrimal, anterior process, development: (0) well developed, dorsal margin confluent with the dorsal margin of the posterodorsal angle of the bone; (1) reduced, offset from the dorsal margin of the lacrimal angle (new)
109. Lacrimal, anterior process, dorsoventral thickness at the half-length of the process (length measured from the posterior margin of the antorbital fenestra to the anterior tip): (0) very slender, much less than the minimal anteroposterior thickness of ventral process; (1) subequal to minimal anteroposterior thickness of ventral process; (2) greater than minimal anteroposterior thickness of ventral process ORDERED (modified from Rauhut & Pol 2021: C126)
110. Lacrimal, anterior process, sharply defined depressions or foramina anterior to the lacrimal recess: (0) absent; (1) one depression or foramen present at the base of the anterior process; (2) multiple depressions or foramina present ORDERED (modified from Rauhut & Pol 2021: C122)
111. Lacrimal, anterior process, development of antorbital fossa: (0) fossa present, bordered dorsally by a marked rim over the entire length of the process; (1) fossa present, only bordered posteriorly by a dorsal rim; (2) fossa present, without dorsal rim on anterior process; (3) fossa absent, lateral surface of anterior process confluent with lateral surface of posterodorsal angle of the lacrimal (modified from Rauhut & Pol 2021: C113)
112. Lacrimal, ventral process, antorbital fossa on the ventral end: (0) present; (1) absent (new)
113. **Lacrimal, antorbital fossa on anterior and ventral processes [inapplicable for taxa that do not have an antorbital fossa in either one or both lacrimal processes]: (0) continuous; (1) separated by an overhang of the lacrimal lamina over the antorbital fenestra (modified from Rauhut & Pol 2021: C116)**
114. Lacrimal, ventral process, dorsoventral groove on anterior surface of the process continuous ventrally with ventral part of the lacrimal antorbital fossa [inapplicable in taxa that do not have a ventral part of the antorbital fossa on the ventral process of the lacrimal]: (0) absent; (1) present (new)
115. Lacrimal, ventral process, contact to maxilla: (0) present; (1) absent (modified from Rauhut & Pol 2021: C114)
116. Lacrimal, ventral process, texture of lateral surface: (0) largely smooth; (1) notably rugose (modified from Rauhut & Pol 2021: C115)
117. Lacrimal, ventral process, shape: (0) broadly triangular, ventral articular end at least 1.5 times as wide anteroposteriorly as the minimal width of the ventral process; (1) strut-like, roughly same width anteroposteriorly throughout ventral process or only very slightly expanded ventrally (modified from Rauhut & Pol 2021: C131)
118. Lacrimal, ventral process, location of minimal anteroposterior width: (0) placed at about mid-height of the jugal process; (1) placed in the upper third of the jugal ramus, close to the lacrimal angle (new)
119. Lacrimal, ventral process, shape of orbital margin in lateral view: (0) concave along its entire length (excluding possible suborbital spurs); (1) straight to notably convex over at least most of its length (new)
120. Lacrimal, ventral process, suborbital spur on posterior edge of process: (0) absent; (1) present (Rauhut & Pol 2021: C127)

### Prefrontal

121. Prefrontal: (0) present; (1) absent (modified from Rauhut & Pol, 2021: C133)
122. **Prefrontal, size: (0) large, widely exposed on the anterodorsal margin of the orbit in lateral view and on the skull roof; (1) moderate, only small exposure on the orbital margin; (2) small, completely displaced from the orbital margin ORDERED (modified from Rauhut & Pol 2021: C134)**

### Jugal

123. Jugal, maxillary contact, inclination: (0) low, less than 45° from the horizontal (measured at the posterior end of the maxillary tooth row); (1) steep, more than 45° from the horizontal (modified from Rauhut & Pol 2021: C100)
124. Jugal, preorbital dorsal expansion: (0) absent; (1) present (modified from Rauhut & Pol 2021: C93)
125. Jugal, anterior extent: (0) excluded from the rim of the internal antorbital fenestra; (1) expressed on the rim of the internal antorbital fenestra (modified from Rauhut & Pol 2021: C94)
126. **Jugal, antorbital fossa: (0) absent or only a slight depression; (1) large, crescentic depression on the anterior end of the jugal forming posteroventral corner of antorbital fossa (modified from Rauhut & Pol 2021: C95)**
127. **Jugal, invasive jugal recess: (0) absent; (1) present (modified from Rauhut & Pol 2021: C95)**
128. **Jugal, height below the orbit in relation to length of jugal anterior to the posterior rim of the postorbital process at the mid-height of this process: (0) less than 0.1; (1) between 0.1 and 0.2; (2) between 0.2 and 0.35; (3) more than 0.35 ORDERED (modified from Rauhut & Pol 2021: C96)**
129. Jugal, main body, longitudinal ridge on the lateral surface: (0) absent; (1) present (Rauhut & Pol 2021: C104)
130. Jugal, ventral margin, localized ventral expansion below the postorbital process (‘cornual process‘): (0) absent; (1) present (modified from Rauhut & Pol 2021: C99)
131. Jugal, foramen on medial surface ventral to postorbital bar (Often called medial jugal foramen): (0) present; (1) absent (Rauhut & Pol 2021: C92)
132. Jugal, postorbital process: (0) present; (1) absent.
133. Jugal, postorbital process, relative height: (0) reaches at least half height of the orbit; (1) ends considerably below the half height of the orbit (modified from Rauhut & Pol 2021: C 97)
134. Jugal, postorbital process, lateral side of anterior margin: (0) level with the posterior part of the lateral surface of the postorbital process; (1) notably thickened laterally (modified from Rauhut & Pol 2021: C 101)
135. Jugal, postorbital process, pronounced rugosity on the posterior margin: (0) absent; (1) present (new)
136. Jugal, posterior termination: (0) at about the half length of the lower temporal fenestra; (1) between the half length and the posterior extent of the lower temporal fenestra; (2) posterior to the lower temporal fenestra (modified from Rauhut & Pol 2021: C102)
137. Jugal, posterior end, morphology: (0) forked for the reception of an anterior process of the quadratojugal; (1) with a single, tapering process ORDERED (new)
138. Jugal, posterior extent of forked processes [inapplicable for taxa without a forked posterior end]: (0) ventral process extends further posteriorly than dorsal process; (1) both processes extend more or less equally posteriorly (new)

### Postorbital

139. Postorbital: (0) present; (1) absent. (new)
140. Postorbital, shape in lateral view: (0) T-shaped, with straight anteriorly directed anterior process; (1) anterior process anterodorsally directed and dorsal border of temporal bar is dorsally concave, postorbital Y-shaped in lateral view; (2) anterior end of frontal process curves ventrally (Rauhut & Pol 2021: C150)
141. **Postorbital, anteroposterior length relative to dorsoventral height: (0) markedly shorter than high; (1) equally long or longer than high (Rauhut & Pol 2021: C145)**
142. **Postorbital, supraorbital brow: (0) absent, anterior process slender and tapering; (1) present, at least anterior process dorsoventrally expanded and at least slightly rugose (modified from Rauhut & Pol 2021: C151)**
143. Postorbital, postorbital brow, surface texture: (0) mildly rugose, with small pits and ridges; (1) pronounced grooves and ridges; (2) granulate (new)
144. Postorbital, supraorbital brow, extent: (0) extending from anterior process to the anterodorsal margin of ventral process; (1) restricted to the anterior process; (2) continuing posteriorly to at least the base of the posterior process or further, overhanging the lateral surface of the ventral process (modified from Rauhut & Pol 2021: C151)
145. Postorbital, supraorbital brow in dorsal view: (0) does not bulge laterally over the orbit; (1) bulges notably laterally over the orbit (modified from Rauhut & Pol 2021: C151)
146. Postorbital, ventral process, shape of posterior margin: (0) sigmoidal, concave dorsally and convex ventrally; (1) straight to slightly convex between ventral end and squamosal articulation (modified from Rauhut & Pol 2021: C146)
147. Postorbital, ventral process, marked step or ridge extending along lateral side, separating a more rugose anterior surface from a smooth posterior surface: 0 absent; 1 present (new)
148. **Postorbital, ventral process, anterior flange intruding into the orbit: (0) absent; (1) present (modified from Rauhut & Pol 2021: C154)**
149. Postorbital, ventral process, flange intruding into the orbit, shape of anterior margin: (0) rounded; (1) pointed anteriorly
150. Postorbital, ventral process, flange intruding into the orbit, shape of ventral margin: (0) straight and ventrally continuous with distal end of jugal process; (1) straight but offset from ventral process by a small notch; (2) notably concave, creating an offset from distal end of ventral process (modified from Rauhut & Pol 2021: C155)
151. Postorbital, ventral process, flange intruding into the orbit, morphology of lateral surface: (0) confluent with remainder of lateral surface; (1) marked step present (modified from Rauhut & Pol 2021: C147)
152. Postorbital, ventral process, median ridge curving anteriorly creating a tubercle-like suborbital process: (0) absent; (1) present (new)
153. Postorbital, ventral process, cross-section at the jugal articulation; (0) triangular; (1) U- shaped with a posteriorly opening groove (slightly modified Rauhut & Pol 2021: C153)
154. Postorbital, dorsal margin above ventral process in lateral view: (0) convex; (1) straight to slightly concave; (2) with pointed, triangular cornual process that is anteriorly offset from a possible step at the beginning of the squamosal articulation (new)
155. Postorbital, dorsal surface, extent of supratemporal fossa: (0) restricted to the dorsal surface above the ventral process, non-depressed area anterior to fossa is as broad or broader than depressed area; (1) supratemporal fossa extends far onto the anterior process of the postorbital, non-depressed surface much smaller than depressed surface (modified from Rauhut & Pol 2021: C149)
156. Postorbital, posterior process, length: (0) less than half of the length of the anterior process; (1) more than half, but less than the length of the anterior process; (2) longer than the anterior process ORDERED (new)
157. Postorbital, posterior process, medial side: (0) straight or concave, dorsal margin forms a sharp rim; (1) convex, medial margin curves into dorsal margin (supratemporal fossa extends onto process) (modified from Rauhut & Pol 2021: C 149)
158. Postorbital, posterior process, mediolateral width: (0) significantly higher than wide, only the distal tip might be widened mediolaterally; (1) strongly broadened, wider than high already at about mid-length (modified from Rauhut & Pol 2021: C148)

### Squamosal

159. Squamosal, anterior and posterior processes, relative length: (0) anterior process subequal to posterior process or shorter; (1) anterior process considerably longer than posterior process (modified from Schade et al. 2023)
160. Squamosal, posterior extent of postorbital facet: (0) ends anterior to or at the anterior border of the ventral process; (1) extends posteriorly to at least the half-width of the ventral process (Schade et al. 2023)
161. Squamosal, prominent and sharply defined ridge extending posteriorly from squamosal-postorbital articulation on lateral side: (0) absent; (1) present (Schade et al. 2023)
162. Squamosal, prominent and sharply defined ridge extending posteriorly from squamosal-postorbital articulation on lateral side, posterior extent [inapplicable for taxa without the ridge]: (0) towards posterior end of quadrate articulation; (1) towards dorsal margin of posterior process, well separated from quadrate cotyle; (2) extends onto the ventral process (new)
163. **Squamosal, dorsal surface, supratemporal fossa: (0) absent, lateral and posterior margin of supratemporal fenestra sharp; (1) present, supratemporal fenestra continues onto dorsal surface of squamosal as a fossa (Rauhut & Pol 2021: C172)**
164. Squamosal contribution to nuchal crest in posterior view: (0) continuous with the parietal nuchal crest; (1) with deep and narrow, slit-like incision between the squamosal and parietal; (2) with broad, U-shaped incision between squamosal and parietal, squamosal nuchal crest much lower than parietal nuchal crest ORDERED (new)
165. Squamosal, quadratojugal process, ventral extent: (0) makes up 50% or less of the height of the infratemporal fenestra; (1) makes up more than 50% of the height of the infratemporal fenestra (modified from Rauhut & Pol 2021: C174)
166. Squamosal, quadratojugal process, shape: (0) tapering; (1) broad, and usually somewhat expanded (Rauhut & Pol 2021: C168)
167. **Squamosal-quadratojugal contact: (0) present; (1) absent (Rauhut & Pol 2021: C166)**
168. Squamosal-quadratojugal contact, morphology: (0) at tip only; (1) broad (Carrano et al. 2012)
169. Development of quadrate cotyle between the ventral and posterior processes of the squamosal in lateral view: (0) forming a laterally open, dorsally concave socket, quadrate head exposed in lateral view; (1) quadrate head partially hidden in lateral view by a flange expanding anteroventrally from the posterior process; (2) quadrate head hidden in lateral view by a bony lamina connecting the ventral and posterior processes; (3) with a horizontal shelf extending posteriorly from the ventral process, partially hiding the quadrate head. (modified from Rauhut & Pol 2021: C169 and C170)

### Quadratojugal

170. **Quadratojugal: (0) present as separate ossification in adults; (1) fused with the jugal bone (1) (modified from Turner et al. 2012).**
171. **Quadratojugal, jugal process, position of anterior end of process relative to infratemporal fenestra: (0) ventral to infratemporal fenestra or at the anterior rim of this opening; (1) anterior to infratemporal fenestra (modified from Rauhut & Pol 2021: C110)**
172. Quadratojugal, anterior process, thickened rugose area: (0) absent; (1) present (modified from Rauhut & Pol 2021: C112)
173. Quadratojugal, lateral surface, marked depression on the angle between the dorsal and anterior processes: (0) absent; (1) present (modified from Rauhut & Pol 2021: C111)
174. Quadratojugal, posterior process visible in lateral view: (0) absent; (1) present (modified from Rauhut & Pol 2021: C108)
175. Quadratojugal, posterior process, shape: (0) dorsoventrally short process confluent with ventral margin and gradually flexing into posterior margin; (1) tab-like, offset from ventral and posterior margin (modified from Rauhut & Pol 2021: C108)
176. Quadratojugal, posterior process, orientation: (0) posteriorly; (1) strongly posteroventrally (modified from Rauhut & Pol 2021: C 108)
177. Quadratojugal-quadrate contact: (0) restricted to the lateral side of the quadrate; (1) quadratojugal wraps around the posteroventral edge of the quadrate (modified from Rauhut 2003)

### Quadrate

178. Quadrate, quadratojugal flange, development: (0) lateral ridge gradually extending from the head ventrally and becoming more pronounced towards the level of the quadrate foramen; (1) expanded lateral lamina that is offset from the shaft (modified from Rauhut & Pol 2021: C176)
179. Quadrate-quadratojugal contact, dorsal portion: (0) quadratojugal wraps around quadrate posteriorly, articular facet on quadrate faces posterolaterally; (1) articulation of quadratojugal faces strictly laterally; (2) quadrate flange wraps around posterior edge of quadratojugal, articular facet faces anterolaterally (modified from Hendrickx et al. 2015)
180. Quadrate, quadrate foramen: (0) absent; (1) present (Rauhut & Pol 2021: C182)
181. **Quadrate, quadrate foramen, shape: (0) semioval notch, widely open laterally; (1) with a ventrally flexed lateral spur at least the dorsal margin; (2) completely enclosed in the quadrate (modified from Rauhut & Pol 2021: C183)**
182. Quadrate, dorsal end: (0) with a single head that fits into a slot on the ventral side of the squamosal; (1) double-headed, medial head contacts the braincase (Rauhut & Pol 2021: C181)
183. Quadrate, pneumatic foramen on the medial base of the pterygoid wing: (0) absent; (1) present (modified from Brusatte et al. 2014, C567)
184. Quadrate, pneumatic foramen on the medial base of the pterygoid wing, shape [inapplicable for taxa without the foramen]: (0) completely open medially; (1) covered at least partially by a medial lamina of the pterygoid wing, recess opens mainly anteroventrally (modified from Brusatte et al. 2014, C567 & Rauhut & Pol 2021: C177, C179)
185. Quadrate, posterior pneumatic foramen: (0) absent; (1) small pneumatic foramen on the posterior side of the quadrate, medial or posteromedial to the quadrate foramen; (2) large pneumatic opening present on the posterior side of the quadrate, above the quadrate condyles and ventral to the level of the quadrate foramen (modified from Rauhut & Pol 2021: C177, C179)

### Nasal

186. **Nasals, fusion in mature individuals: (0) unfused; (1) partially or completely fused (slightly modified from Rauhut & Pol 2021: C79)**
187. Nasals, shape: (0) expanding posteriorly; (1) of subequal width throughout their length (Rauhut & Pol 2021:83)
188. **Nasals, posterior margin of nares, development: (0) gently convex; (1) depressed into a distinct supranarial fossa; (2) laterally splayed hood (modified from Rauhut & Pol 2021: C86)**
189. Nasals, supranarial fossa, development: (0) shallow fossa with indistinct margins; (1) well-developed fossa with especially sharp posterodorsal margin (modified from Rauhut & Pol 2021: C86)
190. **Nasals, exterior surface, texture: (0) smooth; (1) rugose with finely pitted surface; (2) with distinct bumps and grooves (modified from Rauhut & Pol 2021: C80)**
191. Nasals, midline crest or horn: (0)absent; (1) present (Rauhut & Pol 2021: C81)
192. Nasals, longitudinal row of enlarged neurovascular foramina on dorsal surface: (0) absent; (1) present (modified from Rauhut & Pol 2021: C88)
193. **Nasals, pronounced lateral rims sometimes bearing lateral cranial crests: (0) absent; (1) present as lateral rims; (2) present as dorsoventrally tall crests ORDERED (Rauhut & Pol 2021: C84)**
194. Nasals, prominent lateral shelf overhanging antorbital fenestra: (0) absent; (1) present (modified from Rauhut & Pol 2021: C89)
195. **Nasals, antorbital fossa, dorsal expansion onto the ventrolateral margin: 0 absent; 1 present (modified from Rauhut & Pol 2021: C48)**
196. **Nasals, pneumatization: (0) absent, (1) present (modified from Rauhut & Pol 2021: 82)**
197. Nasals, posterolateral process: (0) absent, anterior ramus of the lacrimal not enveloped by nasal process; (1) present, posterolateral process of nasal envelops part of the anterior ramus of the lacrimal (Rauhut & Pol 2021: C90)
198. Nasal-frontal contact, location relative to mid-length of orbit: (0) entirely anterior; (1) reaches mid-length at the median part of the skull; (2) reaches mid-length laterally but not medially (modified from Rauhut & Pol 2021: C87)

### Frontal

199. Frontal-nasal suture, configuration along skull midline: (0) frontal inserts between the nasals; (1) nasals insert between the frontals; (2) frontal-nasal suture transversely orientated (modified from Rauhut & Pol 2021: C136)
200. Frontal-nasal suture, anterolateral process of frontal clasping nasal laterally: (0) absent; (1) present (modified from Rauhut & Pol 2021: C136)
201. **Frontal, fusion of interfrontal suture: (0) paired frontals, suture open; (1) fused in presumed adults (Rauhut & Pol 2021: C144)**
202. **Frontal, dorsal surface: (0) anteroposteriorly straight; (1) anteroposteriorly vaulted (modified from Rauhut & Pol 2021: C195)**
203. Frontal, median cornual process: (0) absent; (1) present (Rauhut & Pol 2021: C143)
204. Frontal, lateral edge: (0) smooth in region of lacrimal/prefrontal suture; (1) edge notched (slightly modified from Rauhut & Pol 2021: C140)
205. Frontal, dorsal surface across the frontal-prefrontal suture in lateral view: (0) straight; (1) expanded dorsally, dorsal surface convex along the suture (new)
206. **Frontal, exposure of orbital rim: (0) widely exposed on the dorsal margin; (1) reduced, only exposed in a deep incision of the dorsal orbital margin between lacrimal/prefrontal and postorbital; (2) frontal excluded from orbital rim by a lacrimal/prefrontal - postorbital contact ORDERED (modified from Rauhut & Pol 2021: C152)**
207. Frontal, supraorbital rim along laterodorsal margin of the orbit: (0) absent; (1) present (Senter 2007)
208. Frontal, postorbital process in dorsal view: (0) smooth transition from orbital margin; (1) sharply demarcated from orbital margin (Rauhut & Pol 2021: C139)
209. Frontal, supratemporal fossa, anterior extent: (0) extends anteriorly onto the frontal, forming a broad, dorsally facing depressed shelf anterior to the supratemporal fenestra; (1) anterior extension reduced, facet on frontal small and steeply posterodorsally inclined or absent (modified from Rauhut & Pol 2021: C137)
210. Frontal, supratemporal fossa, anteromedial emargination: (0) straight or continuously curved; (1) strongly sinusoidal on the frontal and reaching onto postorbital process; (2) sinusoidal, with a lateral projection at the frontoparietal suture (modified from Rauhut & Pol 2021: C138)
211. Frontal, supratemporal fossa, raised ridges along the anteromedial and medial margins: (0) present; (1) absent (modified from Rauhut & Pol 2021: C158)
212. Frontal, supratemporal fossa, shelf overhanging anteromedial corner of the fossa: (0) absent; (1) present (Rauhut & Pol 2021: C162)
213. Frontal-parietal suture, morphology within supratemporal fossa: (0) continuous between frontal and parietal; (1) marked as a raised ridge at which the slope changes (new)
214. Frontal-parietal contact, fusion: (0) unfused; (1) frontal at least partially or completely fused to parietal (slightly modified from Rauhut & Pol 2021: C142)

### Parietal

215. **Parietals, fusion: (0) separate; (1) at least partially fused in subadult to adult individuals (modified from Rauhut & Pol 2021: C159)**
216. Parietals, sagittal crest: (0) absent, supratemporal fenestrae separated by horizontal plate formed by parietals; (1) short crest present posteriorly, supratemporal fenestrae anteriorly separated by anteriorly widening triangular plate; (2) thin sagittal crest present along sagittal suture, supratemporal fenestrae nearly confluent
217. Parietals, sagittal crest, form [inapplicable in taxa that do not have a sagittal crest]: (0) low midline ridge; (1) well-developed midline bony sheet (slightly modified from Rauhut & Pol 2021: C161)
218. Parietals & Frontals, sagittal crest, extent [inapplicable in taxa that do not have a sagittal crest]: (0) restricted to the parietals; (1) extends anteriorly onto the frontals [this character is inapplicable in taxa that do not have a sagittal crest] (new)
219. **Parietal-supraoccipital, nuchal crest, projection in skull midline: (0) approximately level with skull roof; (1) projects notably dorsal from skull roof (modified from Rauhut & Pol 2021: C163)**
220. Parietal-supraoccipital, nuchal crest in posterior view: (0) confluent in the midline; (1) dorsally indented at the midline (new)
221. Parietal, nuchal crest, height at midline: (0) approximately level with the dorsal end of the supraoccipital; (1) notably exceeds the dorsal end of the supraoccipital and visible in occipital view above supraoccipital (new)
222. Parietal, posterior surface, sagittal crest in continuation of the supraoccipital sagittal crest[inapplicable in taxa where the parietal nuchal crest does not exceed the height of the supraoccipotal]: (0) absent; (1) present (new)
223. Parietal, posteriorly projecting tongue overlapping supraoccipital in dorsal view: (0) absent, parietal does not significantly overlap supraoccipital in dorsal view; (1) present, parietal nearly completely or completely obscures the supraoccipital in dorsal view (Rauhut & Pol 2021: C164)

## Braincase

### Foramen Magnum

224. Foramen magnum, paired raised lips for attachment of the proatlas along the dorsolateral rim: (0) inconspicuous; (1) marked (Schade et al. 2023)
225. Foramen magnum, shape: (0) subcircular, slightly wider than tall; (1) oval, taller than wide (Rauhut & Pol 2021: C217)
226. **Foramen magnum, size: (0) smaller than or subequal to occipital condyle; (1) larger than occipital condyle (Rauhut & Pol 2021: C218)**
227. Foramen magnum, composition of dorsal margin: (0) formed by contacting exoccpitials; (1) narrow median process of supraoccipital separates exoccipitals and forms small part of the dorsal margin; (2) ventral margin of supraoccipital forms most of the dorsal margin ORDERED (modified from Rauhut & Pol 2021: C220)
228. Foramen magnum, elevated tubercle at the dorsal margin: (0) absent; (1) present (new)

### Occipital Condyle

229. Occipital condyle, orientation of long axis: (0) posteriorly; (1) posteroventrally (modified from Rauhut & Pol 2021:C197)
230. Occipital condyle, size of dorsal groove as compared to width of the foramen magnum: (0) wide, wider than 50% of the width of the foramen magnum; (1) narrow, less than 50% of the width of the foramen magnum (modified from Rauhut & Pol 2021: C227)
231. Occipital condyle, lateral margins in dorsal or ventral view: (0) anterolaterally expanding; (1) parallel or with slight constriction (modified from Rauhut & Pol 2021: C223)
232. Occipital condyle, shape of posterior articular surface: (0) flattened; (1) subspherical (modified from Rauhut & Pol 2021: C223)
233. Occipital condyle, ventral margin in lateral view: (0) straight or slightly anteroventrally expanding; (1) rim of the articular surface forms notable ventral lip (modified from Rauhut & Pol 2021: C223)
234. Occipital condyle, ventral margin, concavity or sulcus indenting into articular surface: (0) absent; (1) present (new)

### Supraoccipital

235. **Supraoccipital, sagittal crest: (0) present and pronounced; (1) absent or reduced to a low, rounded eminence (Rauhut & Pol 2021: C212)**
236. Supraoccipital, sagittal crest, development [inapplicable in taxa that have a reduced sagittal crest]: (0) sharply defined, narrow ridge; (1) broad, transversely rounded ridge (new)
237. Supraoccipital, sagittal crest, shape of dorsal end: (0) single; (1) bifurcated (new)
238. Supraoccipital, sagittal crest, maximal width of dorsal expansion: (0) less than or subequal to width of foramen magnum; (1) notably greater than width of foramen magnum (Schade et al. 2023)
239. Posterior exit of mid-cerebral vein: 0 on the posterior surface of the supraoccipital, without any associated marked depressions; 1 placed within a notable, funnel-shaped depression; 2 associated with a curved groove leading towards the posttemporal fenestra (Schade et al. 2023)

### Otoccipital

240. Caudal (posterior) tympanic recess: 0 absent; 1 present (Rauhut & Pol 2021: C241)
241. Otoccipital, placement of columellar recess: (0) on the anterior side of the paroccipital process, separated from the occiput by the crista tuberalis; (1) within the crista tuberalis, interrupting this crest and visible as a notch in anterior or posterior view (new)
242. Otoccipital, horizontal ridge extending from the dorsal edge of the paroccipital process medially above the foramen magnum: (0) absent; (1) present, developed as a rounded swelling; (2) present, developed as a sharp crest; ORDERED (new)
243. Otoccipital (	exoccipital+opisthotic), marked depression on posterior surface dorsolateral to the foramen magnum: (0) absent; (1) present (slightly modified from Rauhut & Pol 2021: C221)
244. Paracondylar recess lateral to the occipital condyle: (0) absent, exit of posteriorly exiting cranial nerves flush with surface of exoccipital; (1) present, exits of cranial nerves located together in a bowl-like depression (modified from Rauhut & Pol 2021: C210)
245. Paracondylar recess, ventral border [inapplicable for taxa that do not have a paracondylar recess]: (0) flush with the posterior surface of the exoccipital/basioccipital below the occipital condyle (or the subcondylar recess, if present); (1) delimited from the posterior surface of the exoccipital/basioccipital surface below the condyle or subcondylar recess by a step or ridge (modified from Rauhut & Pol 2021: 234)
246. CN X and XI, courses: (0) exit the braincase laterally, anterior to the crista tuberalis; (1) exit the braincase posteriorly onto the occiput through a foramen that pierces the exoccipital from the recessus scala tympani (modified from Rauhut & Pol 2021: 211)

### Paroccipital processes

247. Paroccipital processes, shape: (0) long and slender, with dorsal and ventral edges nearly parallel; (1) short, deep with convex dorsal margin (slightly modified from Rauhut & Pol 2021: C213)
248. Paroccipital processes, direction: (0) laterally or slightly ventrolaterally; (1) project strongly ventrolaterally, distal ends placed entirely below the level of the foramen magnum (modified from Rauhut & Pol 2021: C214)
249. Paroccipital processes, angle between the processes in dorsal or ventral view:(0) < 100° (processes directed strongly posterolaterally); (1) 110° - 140° (processes directed more laterally); (2) > 140°(processes directed almost entirely laterally) ORDERED.
250. Paroccipital processes, dorsal edge: (0) with straight dorsal edge; (1) distal end twists anteriorly, distal ends of the processes oriented transversely rather than vertically (Rauhut & Pol 2021: C215)
251. **Paroccipital processes, ventral rim at the base of the processes: (0) above or level with the dorsal border of the occipital condyle; (1) situated at mid-height of occipital condyle or lower (Rauhut & Pol 2021: C216)**

### Basioccipital

252. Basioccipital, width ventral to the occipital condyle: (0) greater than width of occipital condyle; (1) subequal to or less than the width of the occipital condyle (modified from Rauhut & Pol 2021: 232)
253. Basioccipital, sharp dorsoventrally oriented ridge situated ventral to occipital condyle: (0) absent; (1) present (slightly modified from Rauhut & Pol 2021: C229)
254. Basioccipital, infracondylar fossa below the occipital condyle: (0) absent; (1) present (Rauhut & Pol 2021: C224)
255. Basioccipital, infracondylar fossa, depth [inapplicable for taxa that do not have a infracondylar fossa]: (0) shallow, without clearly defined margins; (1) deep, bordered laterally by well-defined ridges (new)
256. Basioccipital, infracondylar fossa, width: (0) narrow and groove-like; (1) broad depression approximately two-thirds the width of the occipital condyle at its widest point (Rauhut & Pol 2021: C225)
257. Basioccipital, infracondylar fossa, borders of fossa: (0) subparallel; (1) diverging ventrally; (2) oval to subcircular, bordered ventrally (new)
258. Basioccipital-otoccipital suture, subcondylar recesses below the paracondylar recess: (0) absent; (1) present (modified from Rauhut & Pol 2021: C233)
259. Basioccipital-otoccipital suture, subcondylar recesses below the paracondylar recess, morphology [inapplicable for taxa without a subcondylar recess]: (0) simple depression; (1) with pneumatopores leading into the neck of the occipital condyle medially; (2) with pneumatic recesses expanding dorsally or anterodorsally into the cranial base; (3) with pneumatic recesses invading the basioccipital medially below the condyle (modified from Rauhut & Pol 2021: C228, C 233)
260. Basal tubera: (0) present; (1) absent or weakly developed (Rauhut & Pol 2021: C230)
261. Basal tubera, composition: (0) equally formed by basioccipital and basisphenoid and not subdivided; (1) subdivided by a lateral longitudinal groove into a medial part entirely formed by the basioccipital, and a lateral part, entirely formed by the basisphenoid (Rauhut & Pol 2021: C231)
262. Basal tubera, spacing (measured from the inflection of the ventrally convex margin of the tubera to the ventrally straight or concave margin of the notch separating the tubera): (0) widely separated, width of notch between tubera at least two thirds the width of the occipital condyle; (1) narrow, notch less than two thirds the width of the occipital condyle (modified from Rauhut & Pol 2021: 232)
263. Basal tubera, shape of notch between the tubera: (0) U-shaped, with rounded or horizontal median margin; (1) V-shaped, gradually expanding ventrally
264. Basal tubera, depth of notch between the tubera: (0) deep; (1) very shallow

### Basisphenoid

265. Basisphenoid, basisphenoid bulla (inflated basisphenoid with convex ventral surface): (0) absent; (1) present (Rauhut & Pol 2021: C203)
266. Basisphenoid, width between basal tubera and basipterygoid processes: 0 approximately as wide as long, or wider; 1 at least 1.5 times longer than wide (modified from Rauhut & Pol 2021: C207)
267. Basisphenoid, basisphenoid recess: (0) absent or only developed as shallow depression without sharp margins: (1) deep and with well-developed margins (modified from Rauhut & Pol 2021: C204)
268. Basisphenoid, basisphenoid recess, depth: (0) longer anteroposteriorly than deep dorsoventrally; (1) deeper dorsoventrally than long anteroposteriorly (modified from Rauhut 2003)
269. Basisphenoid, basisphenoid recess, shape of opening: elongate oval (0), teardrop-shaped, narrowing posteriorly (1), subcircular (2), anteroposteriorly compressed, slit-like (3), trapezoidal, widening posteriorly (4) (modified fromRauhut & Pol 2019)
270. Basisphenoid, basisphenoid recess, subdivision: (0) single undivided opening; (1) partially divided into a left and right compartment by a thin lamina internally; (2) divided into a double recess posteriorly and a single recess anteriorly; (3) internally subdivided into paired anterior recesses and a large, unpaired posterior recess by two oblique laminae; (4) subdivided into single anterior and single posterior recesses by a transverse lamina or wall of bone (modified from Rauhut & Pol 2021: 206)
271. Anterior tympanic recess in the braincase: 0 shallow, without clearly defined margins on the basisphenoid; 1 deep, with clearly defined margins on the basisphenoid (modified from Rauhut & Pol 2021: C237)
272. Basisphenoid, location of basipterygoid processes relative to basal tubera: (0) anterior or slightly anteroventral, basisphenoid recess (if present) opens ventrally; (1) anteroventrally, basisphenoid recess opens posteroventrally; (2) almost directly ventral, basisphenoid recess opens more posteriorly than ventrally ORDERED (modified from Rauhut & Pol 2021: 208)
273. Basisphenoid, basipterygoid processes, development: (0) well-developed, extending as distinct processes from the base of the basisphenoid; (1) absent (modified from Rauhut & Pol 2021: C198)
274. Basisphenoid, basipterygoid processes, relative anteroposterior length at base compared to proximodistal height: (0) longer anteroposteriorly than high proximodistally; (1) about as high proximodistally as anteroposteriorly long or slightly higher; (2) considerably higher proximodistally than anteroposteriorly long ORDERED (modified from Rauhut & Pol 2021: C199)
275. Basisphenoid, basipterygoid processes, arrangement in posterior or anterior view: (0) narrow, processes mainly ventrally projecting; (1) more laterally than ventrally projecting processes (modified from Rauhut & Pol 2021: C200)
276. Basisphenoid, basipterygoid processes, arrangement in lateral view: (0) processes project anteroventrally; (1) processes project strictly ventrally (modified from Rauhut & Pol 2021: C200)
277. Basisphenoid, basipterygoid processes, articular surface for pterygoid, direction: (0) strictly anteriorly or mainly anteriorly directed; (1) anteroventrally or ventrally directed
278. Basisphenoid, basipterygoid processes: (0) solid; (1) hollow (Rauhut & Pol 2021: C201)
279. Basipterygoid recesses, development on dorsolateral surfaces of basipterygoid processes: (0) absent; (1) present (Rauhut & Pol 2021: C202)

### Parasphenoid

280. Cultriform process, orientation and shape: (0) rod like, directed anteriorly; (1) triangular in lateral view and anterodorsally directed (modified from Rauhut et al. 2010)
281. Cultriform process, expanded parasphenoid bulla at the base of the process: (0) absent; (1) present (modified from Rauhut & Pol 2021: 209)
282. Cultriform process, subsellar recess, on the ventral side of base of the process: (0) absent; (1) present as a shallow depression; (2) deep, narrow, flanked laterally by bony lamina extending from the basipterygoid processes towards the cultriform process ORDERED (new)
283. Cultriform process, longitudinal groove on the ventral side of the process: (0) absent, ventral surface convex transversely; (1) shallow groove without clearly defined margins; (2) well-developed groove with sharply defined margins (new)
284. Cultriform process, dorsal process anterior to the pituitary fossa: (0) absent; (1) present (Schade et al. 2023)
285. Abducens nerve (CN VI), location of exit: (0) placed within the pituitary fossa or the depression surrounding it; (1) placed lateral to the pituitary fossa and its surrounding depression (modified from Rauhut & Pol 2021: C222; see Rauhut & Pol 2019: C105)

### Prootic

286. Prootic, depression for pneumatic recess (=dorsal tympanic recess): (0) absent; (1) present as a dorsally open fossa on prootic/opisthotic; (2) present and invasive, often as a deep, posterolaterally directed concavity (modified from Rauhut & Pol 2021: C238, 239)
287. Prootic, superficial lamina forming a triangular posterior process below the columellar recess: (0) absent; (1) present (new; Bever et al. 2012)
288. Prootic-opisthotic, crista interfenestralis: (0) confluent with lateral surface of prootic and opisthotic at the ventral border of the fenestra ovalis; (1) distinctly depressed within middle ear opening (modified from Rauhut & Pol 2021: C240)
289. Subotic recess (pneumatic fossa ventral to fenestra ovalis): 0 absent; 1 present as a posterior subdivision of the anterior tympanic recess; 2 present as a distinct recess separate from the anterior tympanic recess (modified from Rauhut & Pol 2021: C244)
290. Cochlear recess of braincase: 0 separate from anterior tympanic recess; 1 confluent within a depression of the anterior tympanic recess (new)
291. Prootic, otosphenoidal crest, preotic pendant: (0) absent; (1) present as a small, posteroventrally facing crest; (2) present as a large, posteroventrally directed flange overhanging the anterior tympanic recess. ORDERED (modified from Rauhut & Pol 2021: C243)
292. Prootic, otosphenoidal crest between the lateral margin of the stapedial groove and the anterodorsal margin of the anterior tympanic recess: (0) continuous; (1) discontinuous, no crest present on the central part of the prootic (new)
293. Prootic, exit of facialis (VII) nerve, relative position: (0) on lateral side of prootic, separated from the trigeminal foramen; (1) placed in a joint depression (prootic fossa) with the trigeminal foramen on the lateral side of the prootic; (2) placed in a large lateral depression on the lateral side of the braincase, together with the anterior tympanic recess, subotic recess and cochlear recess (new; see Norell et al. 2009; Bever et al. 2013)
294. Prootic, exit of facialis nerve: (0) single foramen; (1) two separate foramina (new)
295. Trigeminal foramen (CN V), placement: (0) between prootic and laterosphenoid; (1) completely within prootic (new)
296. Trigeminal foramen (CN V), morphology: (0) single; (1) partly split; (2) fully split ORDERED (Rauhut & Pol 2021: C219)
297. Exit of mid-cerebral vein: 0 included in trigeminal foramen; 1 vein exits braincase through a separate foramen anterodorsal to the trigeminal foramen (Rauhut & Pol 2021: C235)

### Laterosphenoid

298. Depression (possibly pneumatic) on ventral surface of postorbital process of laterosphenoid: 0 absent; 1 present (Rauhut & Pol 2021: C245)
299. Contact between the frontal and laterosphenoid at the anterior end of the supratemporal fenestra, visible in dorsal view: 0 present; 1 absent, frontal excluded from laterosphenoid by a lateral process of the parietal (modified from Rauhut & Pol 2021: C246)

### Ethmoid ossifications

300. **Sphenethmoid, ossification: (0) unossified; (1) ossified (Rauhut & Pol 2021: C248)**
301. **Interorbital septum in adults (mesethmoid): (0) unossified; (1) ossified (modified from Rauhut & Pol 2021: C247)**

### Endocranium

302. Brain, proportions of forebrain: (0) small and narrow; (1) significantly enlarged and triangular in dorsal view (also as seen by the forebrain facet on the ventral side of the frontal) (Rauhut & Pol 2021: C236)
303. Brain, length of olfactory bulbs relative to length of brain: (0) elongate, olfactory bulbs at least two thirds of the length of the brain; (1) reduced, olfactory bulbs less than half the length of the brain (new)

## Palate

304. Suborbital fenestra: (0) present; (1) absent (modified from Rauhut & Pol 2021: C193)
305. Suborbital fenestra, size in relation to subtemporal fenestra [inapplicable for taxa that do not have a suborbital fenestra or lack the ectopterygoid]: (0) considerably smaller; (1) subequal in size or larger (modified from Rauhut & Pol 2021: C193)

### Pterygoid

306. Pterygoid-palatine contact: (0) continuous; (1) discontinuous in the mid-region, resulting in a pterygopalatine fenestra (Rauhut & Pol 2021: C190)
307. Pterygoid, ectopterygoid wing, development: (0) well developed, with posterior margin set at an angle of approximately 90° towards the long axis of the pterygoid; (1) reduced or absent (modified from Rauhut & Pol 2021: C191)
308. Pterygoid, ectopterygoid wing, ventromedially opening pneumatic recess confluent with the ectopterygoid recess: (0) absent; (1) present (modified from Eddy & Clarke 2011: C77)

### Ectopterygoid

309. Ectopterygoid: (0) present; (1) absent (new)
310. Ectopterygoid, relative position: (0) posterior to palatine; (1) lateral to palatine (Rauhut & Pol 2021: C187)
311. Ectopterygoid-palatine contact: (0) absent, palatine and ectopterygoid separated by pterygoid; (1) present (Rauhut & Pol 2021: C189)
312. Ectopterygoid: (0) slender, without ventral fossa; (1) expanded, with a distinct ventral groove medially; (2) expanded, with a groove from which the ectopterygoid body is invaded medially (ectopterygoid medial recess of Eddy & Clarke 2011) ORDERED (modified from Rauhut & Pol 2021: C185)
313. Medial opening of ectopterygoid medial recess: (0) developed as an undercut of the ventral groove into the ectopterygoid body, extending over most of the anteroposterior width of the ectopterygoid; (1) anteroposteriorly restricted, developed as a large foramen (modified from Rauhut & Pol 2021: C185) (not applicable to taxa in which the ectopterygoid body is not invaded)
314. Ectopterygoid, lateral ramus, anteroventral process: (0) absent; (1) present (Rauhut & Pol 2021: C188)

### Palatine

315. **Palatine, vomerine process, pneumatic recess on the base of the process on dorsolateral side: (0) absent; (1) present (modified from Rauhut & Pol 2021: C184)**
316. Palatine, confluence of jugal, pterygoid and vomerine process, deep, posterodorsally opening recess: (0) absent; (1) present (Schade et al. 2023)
317. Palatine, maxillary process, relative length (as measured from the anterior end of the junction with the vomerine process to the anterior tip): (0) less than palatine body between the internal choanae and suborbital fenestra, (1) longer than the palatine body; (2) strongly elongate, extending anterior to the vomerine process ORDERED (modified from Rauhut & Pol 2019)
318. Palatine, jugal process: (0) at least half the length of the palatine body; (1) reduced, less than half the length of the palatine body; (2) absent ORDERED (modified from Rauhut & Pol 2021: C192)
319. Palatine, pterygoid process, relative length: (0) longer than palatine body; (1) shorter than palatine body (new)
320. Palatine, vomerine process, posterior expansion: (0) absent, vomerine process ± hook-shaped in lateral view; (1) present (new)

## Mandible

321. Mandible, mandibular fenestra: (0) present; (1) absent (modified from Rauhut & Pol 2021: C249)
322. Mandible, mandibular fenestra, minimum height of surangular above the fenestra: (0) accounts for less than half of the height of the mandible at this point; (1) accounts for half the height or more of the height of the mandible (modified from Rauhut & Pol 2021: C249, C279)
323. Mandible, mandibular fenestra, length compared to total length of mandible: (0) < 10%; (1) 10-20%; (2) >20% ORDERED (modified from Rauhut & Pol 2021: C249)
324. Mandible, mandibular fenestra, composition of posterior margin in lateral view of the articulated mandible: (0) formed largely or entirely by the surangular, with an anteroventrally directed process of the surangular forming the posteroventral margin; (1) formed at least partially or entirely by the angular, anteroventrally directed process of surangular absent (new)
325. Mandible, internal mandibular fenestra, length relative to length of mandible: (0) less than 40%; (1) more than 40% (modified from Rauhut & Pol 2021: C252)

### Dentary

326. Dentary, shape in lateral view: (0) at least somewhat expanding posteriorly; (1) with subparallel margins up to the surangular articulation (modified from Rauhut & Pol 2021:C269, 270)
327. Dentary, mandibular symphysis, fusion: (0) absent; (1) present (Rauhut & Pol 2021: C257)
328. Dentary, mandibular symphysis, curvature in occlusal view: (0) straight, dentaries meet in a flat symphysis that is aligned with the long axis of the bone; (1) slightly medially recurved, articulated dentaries form a V-shape in dorsal view; (2) strongly medially curved, articulated dentaries form a U-shape in dorsal view ORDERED (modified from Rauhut & Pol 2021: C255, C256)
329. Dentary, anterior end, ventral deflection in lateral view: (0) absent, anterior end in line with main part of buccal edge; (1) present, anterior end deflected ventrally (Rauhut & Pol 2021: C258)
330. Dentary, mandibular symphysis, inclination of symphysis in relation to the dorsal margin of the anterior end of the dentary: (0) more than 40°; (1) less than 40° (modified from Rauhut & Pol 2021: C260)
331. Dentary, anterior end, shape: (0) rounded, gently flexing into the ventral margin ventrally; (1) squared, anterior margin straight and set at a marked angle towards the ventral margin; (2) with straight anterior margin and anterodorsally pointed (modified from Rauhut & Pol 2021: C253)
332. **Dentary, anterior end, dorsal expansion: (0) absent; (1) small expansion present, less than 1/3 of the minimal height of the dentary; (2) large expansion, more than 1/3 the minimal height of the dentary ORDERED (modified from Rauhut & Pol 2021: C253)**
333. Dentary, anterior end, dorsally expanded arcuate eminence: (0) absent; (1) present (modified from Rauhut & Pol 2021: C254)
334. **Dentary, anterior end, ventral expansion: (0) absent; (1) present (modified from Rauhut & Pol 2021: C253)**
335. Dentary, anterior end, ventral process in the area of the symphysis: (0) absent; (1) present (modified from Rauhut & Pol 2021: C253)
336. Dentary, lateral surface, nutrient foramina: (0) superficial; (1) placed in a longitudinal groove (modified from Rauhut & Pol 2021: C267)
337. Dentary, lateral surface, lateral foraminal groove, development: (0) most conspicuous in the medial part of the dentary, but shallowing posteriorly; (1) shallow anteriorly and deepening posteriorly (new)
338. Dentary, lateral surface, lateral foraminal groove, relative position posteriorly: (0) more or less parallel to alveolar border; (1) approaching alveolar border posteriorly (new)
339. Dentary, lateral surface, texture below the lateral row of foramina: (0) smooth; (1) sculptured by low ridges and grooves (modified from Rauhut & Pol 2021: C263)
340. Dentary, lateral surface, large triangular depression on the posterior part: (0) absent; (1) present (modified from Rauhut & Pol 2021: C268)
341. Dentary, medial surface, meckelian foramina, number: (0) one; (1) two (Rauhut & Pol 2021: C264)
342. Dentary, lateral surface: (0) flat; (1) with lateral ridge or shelf and inset tooth row (Rauhut & Pol 2021: C266)
343. Dentary, posterior end, marked dorsal expansion: (0) absent; (1) present (modified from Rauhut & Pol 2021: C269)
344. **Dentary, posterior end, ventral edge in lateral view: (0) straight or nearly straight; (1) descends strongly posteriorly, forming a marked ventral expansion (modified from Rauhut & Pol 2021: C271)**
345. Dentary, dorsal surangular process (sensu Sampson & Witmer 2007), posterior extent: (0) placed far anterior to anterior margin of mandibular fenestra; (1) placed over anterior part of mandibular fenestra; (2) reaches far posteriorly, posterior to the end of the ventral surangular process ORDERED (modified from Rauhut & Pol 2021: C262)
346. Dentary, ventral surangular process (sensu Sampson & Witmer 2007): (0) absent; (1) present, but small, restricted to the anterodorsal border of the mandibular fenestra; (2) large, forming half or more of the dorsal margin of the mandibular fenestra ORDERED (modified from Rauhut & Pol 2021: C261)
347. Dentary, ventral (angular) process, posterior extent: (0) approximately at the same level as posterior end of ventral surangular process; (1) reaching far posterior to posterior end of ventral surangular process (modified from Rauhut & Pol 2021: C262)

### Splenial

348. Splenial, exposure on the lateral side of the mandible: (0) absent or minimal; (1) exposed as a broad triangle between dentary and angular (Rauhut & Pol 2021: C287)
349. Splenial, anterior end, morphology: (0) single tapering process; (1) forked or stepped, with a long ventral process; (2) forked, with two subequal anterior processes; (3) forked, with a longer dorsal than ventral process (new)
350. Splenial, myliohyoid foramen: (0) absent; (1) present (modified from Rauhut & Pol 2021: C288)
351. Splenial, myliohyoid foramen, size [measured as greatest height of foramen compared to height of splenial at this level]: (0) small (less than 15 %); (1) intermediate (between 15 % and 25 %); (2) large ( 25% or more) ORDERED (modified from Rauhut & Pol 20231: C288, see Schade et al. 2023)
352. Splenial, myliohyoid foramen, form: (0) completely enclosed in the splenial; (1) opened anteroventrally (Rauhut & Pol 2021: C289)
353. Splenial, foramen in the dorsal part of the anterior half, dorsal to the myliohyoid foramen: (0) absent; (1) present (new)
354. Splenial, posterior edge, contour: (0) straight; (1) curved; (2) notched (modified from Rauhut & Pol 2021: C290)

### Supradentary

### Surangular

355. Surangular, pronounced coronoid eminence: (0) absent; (1) present (Rauhut & Pol 2021: C272)
356. Surrangular, spinous process intruding into the mandibular fenestra: (0) absent; (1) present (modified from Rauhut & Pol 2021: C250)
357. **Surangular, anterior surangular foramen: (0) absent; (1) present (modified from Rauhut & Pol 2021: C276)**
358. Surangular, lateral ridge on the dorsal part of the surangular anteroventral to the mandibular articulation: (0) absent; (1) present (modified from Rauhut & Pol 2021: C278)
359. **Surangular, depressed lateral shelf for the attachment of the M. adductor mandibulae externus superficialis, bound medially by a dorsally facing ridge: (0) absent, dorsal surface of the surangular mediolaterally convex anterior to the glenoid; (1) present (Schade et al. 2023)**
360. Surangular, shelf for the attachment of the M. adductor mandibulae externus superficialis [inapplicable for taxa that do not have a lateral shelf]: (0) faces mainly dorsally; (1) faces notably laterodorsally (Schade et al. 2023)
361. **Surangular, posterior surangular foramen (foramen in lateral surface rostral to mandibular articulation): (0) absent; (1) present (Rauhut & Pol 2021: C274)**
362. Surangular, posterior surangular foramen, number: (0) one; (1) two (Rauhut & Pol 2021: C275)
363. Surangular, posterior surangular foramen, size: (0) small foramen; (1) large fenestra, approximately 30% depth of posterior surangular (modified from Rauhut & Pol 2021: C277)

### Angular

364. Angular, posterior extent: (0) exposed almost to end of mandible in lateral view, reaches beyond the posterior margin of the glenoid; (1) excluded from posterior end, angular suture turns ventrally and meets ventral border of mandible below glenoid; (2) posterior end of angular completely anterior to glenoid in lateral view ORDERED (modified from Rauhut & Pol 2021: C284)

### Coronoid

365. Coronoid, ossified as a separate element even in late ontogeny: (0) present; (1) absent (modified from Rauhut & Pol 2021: C285)
366. Coronoid, shape: (0) triangular; (1) splint-like (modified from Rauhut & Pol 2021: C286)
367. Coronoid, supradentary ossification covering the dentary interdental plates medially: (0) absent, (1) present (new)

### Prearticular

368. Prearticular, shape of anterior margin facing the splenial: (0) moderately inclined and straight or convex; (1) steeply inclined and notably concave (new)
369. Prearticular, indentation or incision in the anteroventral margin: (0) absent; (1) present (Schade et al. 2023)
370. Prearticular, indentation or incision in the anteroventral margin, shape: (0) shallow indentation; (1) deep notch (new)

### Antarticular

371. Mandible, ossified antarticular between articular, surangular and prearticular: (0) absent; (1) present (Rauhut & Pol 2021: C294)

### Glenoid

372. Mandibular glenoid, morphology: (0) formed by notably concave cotyles for the condyles of the quadrate; (1) convex in lateral view and with anteroposteriorly oriented longitudinal ridge (new)
373. Mandibular glenoid, anteroposterior length of articular fossa in relation to length of quadrate condyles: (0) subequal; (1) about twice or more as long, allowing anteroposterior movement of mandible (modified from Rauhut & Pol 2021: C291)
374. Mandibular glenoid, anterior rim in lateral view: (0) confluent with the dorsal margin of the surangular; (1) raised above the dorsal margin of the surangular (modified from Rauhut & Pol 2021: C293)
375. Mandibular glenoid, lateral expansion of the articular facet, overhanging the lateral side of the posterior mandible: (0) absent; (1) present as a small lateral ridge; (2) present as a large, semicircular flange (new)
376. Mandibular glenoid, posterior margin of the lateral side: (0) slightly raised, approximately as high as anterior margin; (1) strongly raised, forming a dorsally projecting process that is more than twice the height of the anterior margin (new)

### Articular

377. Articular, medially to medioventrally directed pending process posterior to the glenoid: (0) present; (1) absent (modified from Rauhut & Pol 2021: C292)
378. Articular, medially widening and medially opening groove posterior to the glenoid: (0) present; (1) absent (new)
379. Articular, chorda tympani foramen, position in dorsal view: (0) at the medial edge of the base of the retroarticular process; (1) more centrally placed, towards the mid-width of the articular (Rauhut & Pol 2021)
380. Articular, retroarticular process, medial process at the posterior end: (0) absent; (1) present (modified from Rauhut & Pol 2021: C283)
381. Articular, vertical columnar process on the medial side of the retroarticular process posterior to the chorda tympani foramen: (0) absent; (1) present (modified from Rauhut & Pol 2021: C283)

### Retroarticular process

382. Mandible, retroarticular process, anteroposterior length in relation to length of mandibular glenoid: (0) as long as or longer; (1) shorter (Schade et al. 2023)
383. Mandible, retroarticular process, orientation in respect to the long axis of the mandible: (0) ventrally; (1) mainly straight; (2) dorsally ORDERED (Rauhut & Pol 2021: C282)
384. Mandible, retroarticular process, dorsal margin of the lateral side: (0) approximately on the same dorsoventral level as the glenoid; (1) strongly offset ventrally from the glenoid (modified from Schade et al. 2023)
385. Mandible, retroarticular process, alignment with the lateral side of the mandible: (0) aligned; (1) offset medially (new)
386. Mandible, retroarticular process, width in relation to width of mandibular glenoid: (0) two thirds or less; (1) subequal (modified from Rauhut & Pol 2021: C280)
387. Mandible, retroarticular process, posterior end in dorsal view: (0) broadly rounded; (1) triangular, tapering posteriorly (new)
388. Mandible, retroarticular process, surface for attachment of M. depressor mandibulae: (0) transversally flat or convex; (1) transversely notably concave, forming a groove (modified from Rauhut & Pol 2021: C280)
389. **Mandible, retroarticular process, orientation of surface for attachment of M. depressor mandibulae: (0) facing dorsally; (1) facing posterodorsally (Rauhut & Pol 2021: C281)**

## Dentition

390. Dentition, upper jaw, size of the largest premaxillary tooth compared to largest maxillary tooth: (0) premaxillary tooth markedly larger; (1) subequal in size; (2) at least one third smaller than largest maxillary tooth ORDERED (new)
391. Dentition, premaxillary and anterior dentary teeth, large teeth in the front, followed by a pairwise arrangement of considerably smaller teeth: (0) absent, teeth more or less equally spaced; (1) present (modified from Carrano et al. 2012: C151)
392. Dentition, maxillary tooth row, posterior extent: (0) extends posteriorly to approximately half the length of the orbit or further posteriorly; (1) ends at the anterior rim of the orbit; (2) completely antorbital, tooth row ends anterior to the posterior margin of the antorbital fenestra; (3) ends below the anterior end of the antorbital fenestra or more anteriorly ORDERED (modified from Rauhut & Pol 2021: C306)
393. Dentition, relative length of longest maxillary tooth crown: (0) less than minimal height of dentary; (1) equal to or longer than minimal dentary height (modified from Rauhut & Pol 2021: C309)
394. Dentition, serrations, relative size: (0) numerous small denticles; (1) few large denticles (modified from Rauhut & Pol 2021: C326)

### Premaxillary teeth

395. Premaxillary teeth: (0) present; (1) absent (Rauhut & Pol 2021: C296)
396. Premaxillary teeth, number: (0) three; (1) four; (2) five; (3) six or more ORDERED (modified from Rauhut & Pol 2021: C297)
397. Premaxillary teeth, arrangement: (0) present, but rostral tip edentulous; (1) fully toothed; (2) restricted to rostral portion (modified from Rauhut & Pol 2021: C303)
398. Premaxillary teeth, direction (in relation to the alveolar rim, so as to consider taxa with non horizontal alveolar rim): (0) decumbent or ventrally projecting; (1) at least anterior teeth procumbent (Rauhut & Pol 2021: C300)
399. Premaxillary teeth, size of first premaxillary tooth in comparison to second tooth: (0) notably smaller (at least 25% of mesiodistal alveolar length); (1) subequal; (2) notably larger (at least 25% of mesiodistal alveolar length) ORDERED (modified from Rauhut & Pol 2021: C298)
400. Premaxillary teeth, size of second premaxillary tooth in comparison to third tooth: (0) notably smaller (at least 25% of mesiodistal alveolar length); (1) subequal; (2) notably larger (at least 25% of mesiodistal alveolar length) ORDERED (modified from Rauhut & Pol 2021: C298, C299)
401. Premaxillary teeth, size of third premaxillary tooth in comparison to fourth tooth: (0) notably smaller (at least 25% of mesiodistal alveolar length); (1) subequal; (2) notably larger (at least 25% of mesiodistal alveolar length) ORDERED (new)
402. **Premaxillary teeth, carinae: (0) present on both mesial and distal side of the tooth; (1) absent on mesial side and present on distal side; (2) absent on both mesial and distal side (Hendrickx & Mateus, 2014)**
403. Premaxillary teeth, carinae, arrangement: (0) nearly symmetrical, on opposite sides; (1) more asymmetrical, mesial carina displaced mesially; (2) both carinae displaced mesially, resulting in a D-shaped cross-section with flat lingual surface (modified from Rauhut & Pol 2021: C302, C304)
404. Premaxillary teeth, mesial carina, shape: (0) oriented mesiodistally; (1) flexed mesially, overhanging the mesial side, giving the tooth a spade-shaped appearance (new)
405. **Premaxillary teeth, serrations (=denticles): (0) present on mesial and distal carinae; (1) absent on mesial and present on distal carina in at least some teeth; (2) absent (modified from Rauhut & Pol 2021: C301)**

### Mesial teeth

406. Mesial teeth, mesial carina, shape of denticles: (0) rounded and symmetrically convex, or slightly asymmetrical; (1) strongly asymmetrical, hooked apically (modified from Rauhut & Pol 2021: C 325; see Hendrickx et al. 2019 C 58)
407. Mesial teeth, distal carina, shape of denticles: (0) rounded and symmetrically convex, or slightly asymmetrical; (1) strongly asymmetrical, hooked apically (modified from Rauhut & Pol 2021: C 325; see Hendrickx et al. 2019: C 59)
408. Mesial tooth crowns, vertical striations: (0) generally absent; (1) present in at least some crowns and/or at least one side of the crown (modified from Rauhut & Pol 2021: C330)

### Maxillary teeth

409. Maxillary teeth: (0) present; (1) absent (Rauhut & Pol 2021: C305)
410. **Maxillary teeth, number: (0) 1-9; (1) 10-14; (2) 15-19: (3) 20 or more ORDERED (Rauhut & Pol 2021: C307)**
411. Maxillary teeth, tooth implantation: (0) separate alveoli; (1) set in an open groove (Rauhut & Pol 2021: C316)
412. Maxillary teeth, spacing: (0) adjacent, closely spaced; (1) more widely spaced (at least one third of an alveolar width apart) in the mid-section of the maxilla; (2) widely spaced in the mid-section and posteriorly; (3) all maxillary teeth widely spaced (modified from Schade et al. 2023)
413. Maxillary dentition, mesial teeth, direction (in relation to the alveolar rim, so as to consider taxa with non horizontal alveolar rim): (0) ventrally or posteriorly; (1) at least some procumbent (modified from Rauhut & Pol 2021: C308)
414. Maxillary dentition, distal teeth, direction (in relation to the alveolar rim, so as to consider taxa with non horizontal alveolar rim): (0) ventrally or posteriorly; (1) at least some procumbent (modified from Rauhut & Pol 2021: C308)
415. Maxillary teeth, mesial portion of the tooth row, expansion of the crown above the base: (0) absent; (1) present in at least some teeth (modified from Rauhut & Pol 2021: C327)
416. Maxillary teeth, distal portion of the tooth row, expansion of the crown above the base: (0) absent; (1) present in at least some teeth (modified from Rauhut & Pol 2021: C327)
417. Maxillary teeth, mesial portion of the tooth row, outline of distal carina in labial or lingual view: (0) concave; (1) straight; (2) convex (Hendrickx et al. 2020)
418. Maxillary teeth, distal portion of the tooth row, outline of distal carina in labial or lingual view: (0) concave; (1) straight; (2) convex; (3) sigmoidal, convex basally and concave apically (Hendrickx et al. 2020)
419. **Maxillary teeth, mesial carina: (0) present; (1) absent (modified from Rauhut & Pol 2021: C310)**

### Lateral teeth

420. Lateral maxillary and dentary teeth, cross-section at the base: (0) ovoid; (1) 8-shaped (modified from Hendrickx & Mateus 2014)
421. Lateral maxillary and dentary teeth, mesial portion of the tooth row, labiolingual compression of the crown (CBR=CBW/CBL): (0) strongly compressed, CBR<0.5; (1) moderately compressed, CBR between 0.5 and 0.75; (2) subcircular, CBR > 0.75 ORDERED (modified from Rauhut & Pol 2021: C313)
422. Lateral maxillary and dentary teeth, distal portion of the tooth row, labiolingual compression of the crown (CBR=CBW/CBL): (0) strongly compressed, CBR<0.5; (1) moderately compressed, CBR between 0.5 and 0.75; (2) subcircular, CBR > 0.75 ORDERED (modified from Rauhut & Pol 2021: C313)
423. Lateral maxillary and dentary teeth, mesial portion of the tooth row, position of the tooth apex in relation to tooth base: (0) above the mesial ½; (1) centrally positioned; (2) above the distal ½; (3) distally positioned ORDERED (modified from Rauhut & Pol 2021: C314)
424. Lateral maxillary and dentary teeth, distal portion of the tooth row, position of the tooth apex in relation to tooth base: (0) above the mesial ½; (1) centrally positioned; (2) above the distal ½; (3) distally positioned ORDERED (modified from Rauhut & Pol 2021: C314)
425. Maxillary and dentary teeth, mesial carina, basal extent: (0) to base of crown; (1) terminates basally above the basal third of the crown (Rauhut & Pol 2021: 311)
426. **Maxillary and dentary teeth, mesial denticles: (0) present; (1) some without; (2) absent ORDERED (modified from Rauhut & Pol 2021: C315)**
427. Lateral teeth, mesial carina, shape of denticles: (0) rounded and symmetrically convex, or slightly asymmetrical; (1) strongly asymmetrical, hooked apically (modified from Rauhut & Pol 2021: C 325; see Hendrickx et al. 2019: C 90)
428. Maxillary and dentary teeth, distal denticles: (0) present; (1) some without; (2) absent ORDERED (modified from Rauhut & Pol 2021: C315)
429. Lateral teeth, distal carina, shape of denticles: (0) rounded and symmetrically convex, or slightly asymmetrical; (1) strongly asymmetrical, hooked apically (modified from Rauhut & Pol 2021: C 325; see Hendrickx et al. 2019: C 91)
430. Lateral tooth crowns, pronounced enamel wrinkles adjacent to the carinae [Cannot be coded for taxa for which only one or few teeth are known]: (0) generally absent; (1) present in at least some teeth in at least some carinae (modified from Rauhut & Pol 2021: C328)
431. Lateral tooth crowns, vertical striations: (0) generally absent; (1) present in at least some crowns and/or at least one side of the crown (modified from Rauhut & POl 2021: C330)
432. Maxillary and dentary teeth, cross-section of roots: (0) labiolingually compressed; (1) subcircular (Rauhut & Pol 2021: C315)

### Palatal teeth

433. Palatal teeth on the palatal process of the pterygoid: (0) present; (1) absent (Rauhut & Pol 2021: C295)

### Dentary teeth

434. Dentary, teeth: (0) present; (1) absent (Rauhut & Pol 2021: C318)
435. Number of dentary teeth: (0) less than 8; (1) 9 to 20; (2) 20 to 29; (3) 30 or more ORDERED (modified from Rauhut & Pol 2021: C320)
436. Dentary dentition, tooth implantation: (0) separate alveoli; (1) set in an open groove (Rauhut & Pol 2021: C323)
437. Dentary, tooth row, anterior extent [inapplicable in taxa that lack dentary teeth]: (0) up to symphysis; (1) ends more posteriorly, anterior end edentulous (modified from Rauhut & Pol 2021: C319)
438. Dentary, tooth row, posterior extent: (0) extends more posteriorly than the mid-length of the dorsal margin of the dentary; (1) ends anterior to the mid-length of the dorsal margin of the dentary (modified from Rauhut & Pol 2021: C319)
439. Dentary dentition, shape across the tooth row: (0) more or less homodont, teeth of equal height, only decreasing in size somewhat posteriorly; (1) gradually increasing from the anterior end towards the mesial mid-section and then decreasing again; (2) with one or more non-gradual transitions in tooth shape (modified from Rauhut & Pol 2021: C321, 322)
440. Dentary dentition, mesial teeth, direction (in relation to the alveolar rim, so as to consider taxa with non horizontal alveolar rim): (0) dorsally or posteriorly inclined; (1) at least some procumbent (anteriorly inclined) (modified from Rauhut & Pol 2021: C324)
441. Dentary dentition, distal teeth, direction (in relation to the alveolar rim, so as to consider taxa with non horizontal alveolar rim): (0) dorsally or posteriorly inclined; (1) at least some procumbent (modified from Rauhut & Pol 2021: C324)
442. **Dentary teeth, mesialmost or first and second tooth or alveolus, relative size (mesiodistal length): (0) subequal to subsequent teeth or slightly smaller; (1) significantly smaller than subsequent teeth (half the size or less); (2) larger than subsequent teeth (modified from Rauhut & Pol 2021: C321; Hendrickx et al. 2019)**
443. **Lateral dentary teeth, mesial carina: (0) present; (1) absent (modified from Rauhut & Pol 2021: C310)**

# Axial skeleton

## Cervical Series

### General

444. Cervical vertebrae, number, at least 9 present: (0) no; (1) yes (modified from Rauhut & Pol 2021: C344)
445. Cervical vertebrae, number, at least 10 present: (0) no; (1) yes (modified from Rauhut & Pol 2021: C344)
446. Cervical vertebrae, number, at least 11 present: (0) no; (1) yes (modified from Rauhut & Pol 2021: C344)
447. Cervical vertebrae, number, at least 12 present: (0) no; (1) yes (modified from Rauhut & Pol 2021: C344)
448. Cervical vertebrae, number, at least 13 present: (0) no; (1) yes (modified from Rauhut & Pol 2021: C344)
449. Cervical vertebrae, number, at least 14 present: (0) no; (1) yes (modified from Rauhut & Pol 2021: C344)

### Proatlas - Atlas - Axis

450. Proatlas, shape: (0) simple tongue shaped; (1) plate like, wide anteriorly, narrowing posteriorly; (2) triradiate (new)
451. Atlas, neural arch, depression on the dorsolateral side of the arch, above the pedicle (0) absent; (1) present (modified from Rauhut & Pol 2021: C331)
452. Atlas, neural arch, development of atlantal epipophyses: (0) spike-like, approximately, as long as articular surface of atlantal postzygapophysis; (1) strongly elongate and rod-like in lateral view; (2) expanded and triangular in lateral view (modified from Rauhut & Pol 2021: C332)
453. Axis, orientation of intercentrum ventral surface: (0) horizontal or slightly anteroventral; (1) tilted anterodorsally (Rauhut & Pol 2021: C339)
454. Axis, pleurocentrum, relative elongation (measured as posterior height in relation to pleurocentrum length): (0) elongate < 75%; (1) short 75% - 120%; (2) very short >120%
455. Axis, pleurocentrum, constriction (measured as minimal transversal width, compared to posterior width in ventral view): (0) < 0.5; (1) > 0.5 (modified from Rauhut & Pol 2021: C341)
456. Axis, pleurocentrum, ventral surface: (0) keeled; (1) flat or simply rounded; (2) grooved (new)
457. Axis, pleurocentrum, ventral keel, shape [inapplicable for taxa without a ventral keel]: (0) weak ridge or step like; (1) pronounced ridge or lamina
458. **Axis, pleurocoel: (0) absent; (1) present (Rauhut & Pol 2021: C338)**
459. Axis, number of pleurocoels: (0) one; (1) two, openings clearly separated from one another (new)
460. Axis, pleurocoel, position [inapplicable for taxa that have two pleurocoels]: (0) at about mid-lenght of centrum; (1) closer to anterior end of the centrum (new)
461. Axis, diapophyses, development: (0) absent; (1) small spike or nubbin; (2) elongate, pendant and overhanging the lateral side of the neural arch and/or centrum ORDERED (modified from Rauhut & Pol 2021: C333)
462. Axis, diapophysis, dorsoventral position on vertebra [inapplicable for taxa that do not have an axial diapophysis]: (0) base of diapophysis at the level of the dorsal rim of the centrum; (1) base of diapophysis located above the dorsal rim of the centrum on the neural arch (new)
463. Axis, diapophysis, anteroposterior position on vertebra [inapplicable for taxa that do not have an axial diapophysis]: (0) just anterior to mid-length of the vertebral centrum (excluding the dens); (1) closer to the anterior end of the centrum (new)
464. Axis, neural arch, prezygodiapophyseal lamina [inapplicable for taxa that do not have an axial diapophysis]: (0)absent; (1) present (new)
465. Axis, neural arch, lateral lamina extending anteriorly from the lateral side of the postzygapophysis (postzygodiapophyseal lamina in taxa in which this connects to the diapophysis): (0) absent; (1) present (new)
466. Axis, neural arch, lateral lamina extending anteriorly from the lateral side of the postzygapophysis, orientation [inapplicable for taxa that do not have the lamina]: (0) extending straight anteriorly or anteroventrally, remaining above the diapophysis in taxa that have a diapophysis; (1) flexing ventrally towards the diapophysis anteriorly (new)
467. Axis, neural arch, pneumatic recess posterodorsal to the prezygapophysis (lateral axial pneumatic chamber): (0) absent; (1) present (new)
468. **Axis, neural spine, shape of dorsal margin: (0) continuously; (1) subdivided by a marked anterior concavity separating low anterior portion from strongly rising posterior portion (modified from Rauhut & Pol 2021: C335)**
469. Axis, neural spine, shape of continuous dorsal margin [inapplicable for taxa with a subdivided neural arch]: (0) more or less straight; (1) strongly convex (modified from Rauhut & Pol: C 335)
470. **Axis, neural spine, height of continuous dorsal margin [inapplicable for taxa with a subdivided neural arch]: (0) increases in height posteriorly; (1) more or less equal throughout the length of the neural spine (modified from Rauhut & Pol: C 335)**
471. Axis, neural spine, shape of dorsal margin posterior to anterior concavity [inapplicable for taxa with continuous neural arch]: (0) straight; (1) markedly convex (modified from Rauhut & Pol: C 335)
472. Axis, neural spine, dorsal end, bifurcation by a posteriorly invaginating notch in dorsal view: (0) absent; (1) present (modified from Rauhut & Pol 2021: C343)
473. Axis, neural spine, dorsal end, shape in posterior view: (0) convexly rounded; (1) straight to indented medially (new)
474. Axis, neural spine, posterior margin, dorsal extent of postspinal fossa: (0) to the tip of the spine; (1) restricted to the base of the spine (modified from Rauhut 2003)
475. **Axis, neural spine, posterior margin, development of attachment for interspinous ligament: (0) absent or low rugose area; (1) dorsoventrally extending rugose ridge on posterior side of dorsal part of spine; (2) rugose rounded tubercle at the base of the neural spine (modified from Rauhut & Pol, 2021: C342)**
476. Axis, spinopostzygapophyseal laminae in posterior view: (0) gradually diverging ventrolaterally from tip of spine, with a straight lateral margin; (1) strongly flaring laterally directly below the tip of the spine, with convex lateral margin; (2) extending strictly ventrally in their dorsal part, only diverging towards their base, margins of the axial spine subparallel (modified from Rauhut & Pol 2021: C335)
477. Axis, spinopostzygapophyseal lamina, ventrolateral extent: (0) more or less straight up to the tip of epipophyses/postzygapophyses; (1) invaginated in posterior view, does not reach the tip of the epipophyses/postzygapphyses (modified from Rauhut & Pol 2021: C340)
478. Axis, epipophyses on postzygapophyses: (0) absent; (1) present (Rauhut & Pol 2021: C336)
479. Axis, epipophyses, shape: (0) ridge or spike like; (1) rounded; (2) rectangular (mediolaterally expanded) (modified from Rauhut & Pol 2021: C337)
480. Axis, epipophyses, posterior extent, compared to posterior extent of postzygapophysis: (0) ends anterior to it; (1) extends approximately to the same level; (2) slightly overhangs the postzygapophysis; (3) significantly overhangs the postzygapophysis to more than half the length of the postzygapophyseal articular facet ORDERED (modified from Rauhut & Pol 2021: C337)
481. Axis, epipophyses, dorsoventral height in respect to the posterior height of the neural arch (as measured from the centrum to the roof of the neural canal): (0) low, less than half; (1) high, half the height of the neural arch or more (modified from Rauhut & Pol 2021: C337)

### Postaxial vertebrae

482. **Postaxial cervical centra, anterior articulation surfaces: (0) concave; (1) platycoelous to very slightly convex; (2) notably convex; (3) heterocoelus (modified from Rauhut & Pol 2021: C358)**
483. Postaxial cervical centra, extend of anterior convexity [inapplicable for taxa without opisthocoelous cervical vertebrae]: (0) moderate, anterior articular surface notably convex, but not semicircular, and anterior ball accounts for less than half of the length of the effective centrum (excluding anterior convexity); (1) extreme, anterior articular surface semicircular and accounts for more than half of the length of the effective centrum (strongly modified from Rauhut & Pol 2021: C369)
484. Postaxial cervical centra, posterior articular surface: (0) slightly concave; (1) deeply concave, cup-shaped; (2) heterocoelous (modified from Rauhut & Pol 2021: C358)
485. Postaxial cervical centra, anterior pneumatic recess, posterodorsal to the parapophysis: (0) absent; (1) present (modified from Rauhut & Pol 2021: C346)
486. Postaxial cervical centra, anterior pneumatic recess, development [inapplicable for taxa without anterior pneumatic recess]: (0) deep depression; (1) foramen leading into the interior of the centrum (modified from Rauhut & Pol 2021: C349)
487. Postaxial cervical centra, anterior pneumatic foramen [inapplicable for taxa without the anterior foramen]: (0) single opening; (1) double opening or interiorly subdivided in at least some cervical vertebrae (modified from Rauhut & Pol 2021: C. 347)
488. Postaxial cervical centra, anterior pneumatic foramen, position [inapplicable for taxa without the anterior foramen]: (0) dorsal to parapophysis; (1) posterior to parapophysis in anterior cervicals; (2) posterior to parapophysis in all cervicals ORDERED (new)
489. Postaxial cervical centra, posterior pneumatic recess, in posterior half of the centrum: (0) absent; (1) present (modified from Rauhut & Pol 2021: C347)
490. Postaxial cervical centra, posterior pneumatic recess, development: (0) fossa; (1) fossa with pneumatic foramen (modified from Rauhut & Pol 2021: C376)
491. Postaxial cervical centra, pneumatization [inapplicable for taxa without pneumatization and not scorable for taxa with a largely incomplete cervical series]: (0) consistent; (1) highly variable in the number size and arrangement of foramina within the series and/or a single vertebra (new)
492. Presacral vertebrae, centra, interior pneumatic spaces [inapplicable for vertebrae that lack pneumatic foramina in the centra]: (0) camerate; (1) camellate (modified from Rauhut & Pol 2021: C 349)
493. Cervical centrum of third cervical vertebra, length compared to length of middle cervicals [mid cervical length/C3 length]: (0) less than 1.3; (1) more than 1.3 (new)
494. **Neural spine of third cervical vertebra: (0) similar to spines of more posterior cervicals; (1) anteroposteriorly slender and strongly backswept (Rauhut & Pol 2019)**

### Anterior cervical vertebrae

495. Anterior cervical vertebrae, anterior articular facet, relative width: (0) approximately as high as wide or higher (width/height ratio 1.1 or lower); (1) significantly wider than high (ratio more than 1.15) ORDERED (modified from Wang et al., 2017: C356)
496. Anterior cervical vertebrae, anterior articular facet, emargination of neural canal: (0) absent; (1) present, articular surface extends higher than floor of neural canal lateral to it (modified from Wang et al., 2017: C356)
497. Anterior and mid cervical centra, lateral side, ridge extending from diapophysis to posteroventral end of the centrum: (0) absent; (1) present (modified from Rauhut & Pol 2021 :C352)
498. Anterior cervical vertebrae, anterior articular facet, shape of ventral margin of anterior (not considering the parapophyses): (0) notably convex; (1) more or less straight; (2) notably concave (modified from Rauhut & Pol 355)
499. Ventral surface of anterior cervicals: 0 keeled; 1 smooth; 2 ventral depression (Wang et al., 2017: C353)
500. Anterior cervical centra relative length: 0 level with or shorter than posterior extent of neural arch; 1 centra extending beyond posterior limit of neural arch (Wang et al., 2017: C357)
501. Anterior to middle cervical vertebrae, pneumatic foramina in prezygapophyseal centrodiapophyseal fossa: (0) absent [only codeable for taxa where a somewhat complete cervical series is known]; (1) present in at least some anterior to middle cervicals (modified from (modified from Rauhut & Pol 2021: 380)
502. Anterior to middle cervical vertebrae, pneumatic foramina in centrodiapophyseal fossa: (0) absent [only codeable for taxa where a somewhat complete cervical series is known]; (1) present in at least some anterior to middle cervicals (modified from Rauhut & Pol 2021: 380)
503. Anterior to middle cervical vertebrae, pneumatic foramina in postzygapophyseal centrodiapophyseal fossa: (0) absent [only codeable for taxa where a somewhat complete cervical series is known]; (1) present in at least some anterior to middle cervicals (modified from Rauhut & Pol 2021: 380)
504. Prezygapophyses in anterior postaxial cervicals: 0 straight; 1 anteroposteriorly convex, flexed ventrally anteriorly (Wang et al., 2017: C367)
505. Prezygapophyses in anterior cervicals: 0 transverse distance between prezygapophyses less than width of neural canal; 1 prezygapophyses situated lateral to the neural canal (Wang et al., 2017: C366)
506. Prezygapophyseal-epipophyseal lamina on dorsal surface of neural arch: 0 absent or poorly developed; 1 low lamina anterior to middle cervicals; 2 extending anteriorly from epipophysis as a mediolaterally thin ridge that separates dorsal surface of diapophysis from rest of dorsal neural arch (Wang et al., 2017: C362)
507. Anterior to middle cervical vertebrae (C3-C6), neural spines, height (measured from the lowest point of the spinopostzygapophyseal lamina to the top of the neural spine) compared to centrum height in posterior view: (0) < 30%; (1) 30 - 80%; (2) > 80% ORDERED (modified from Rauhut & Pol 2021: C 365)
508. Anterior to middle cervical vertebrae, neural spines, elongation anteroposterior length (measured from the dorsal end of the spinopostzygapophyseal lamina anteriorly towards the prespinal fossa), compared to posterior height (measured as in the previous character): (0) highly elongated, ratio > 300%; (1) moderately elongated (2) very short, much less than 50% ORDERED (new)
509. Anterior to middle cervical vertebrae, neural spines, relative mediolateral width compared to anteroposterior length: (0) much less, spines more or less sheet-like; (1) subequal or more, spines robust (new)
510. **Mid-cervical vertebrae, neural spine, position over centrum (as judged by the relative distances of the anterior margin of the spine in relation to the anterior margin of the centrum and the posterior margin of the spine in relation to the posterior margin of the centrum, respectively): (0) more over the posterior half of the centrum; (1) more or less centrally; (2) more over the anterior half of the centrum (modified from Rauhut & Pol 2021: C370) ORDERED**
511. Postzygapophyses of cervical vertebrae 2-4: 0 well-separated, or connected only at the base; 1 medially connected along their entire length by a intrazygapophyseal lamina that is dorsally concave for attachment of the interspinous ligaments (Wang et al., 2017: C363)
512. Epipophyses in anterior cervical vertebrae: 0 absent or poorly developed; 1 well-developed (Wang et al., 2017: C360)
513. **Form of well-developed cervical epipophyses: 0 proximal to postzygapophyseal facets; 1 strongly overhanging postzygapophyseal facets (Wang et al., 2017: C361)**

### Middle cervical vertebrae

514. Middle cervical centra, length [measured in a straight line along the dorsal margin of the centrum and excluding rounded anterior end in opisthocoelous vertebrae] against posterior centrum height: (0) less than one; (1) between one and two times; (2) between two and three times; (3) more than three times as long as high ORDERED (modified from Rauhut & Pol 2021: C352)
515. Ventral surface of middle cervicals: 0 keeled, sometimes bearing a shallow groove along the keel; 1 smooth, convex or flat; 2 craniocaudally elongate ventral depression (Wang et al., 2017: C378)
516. Cervical vertebrae, middle, shape of anterior pleurocoel: 0 round; 1 anteroposteriorly elongate (Wang et al., 2017: C377)
517. Mid-cervical vertebrae, pleurocoel, position in relation to parapophysis: (0) separate; (1) excavates dorsal surface of parapophysis (modified from Benson 2010: C112)
518. Accessory fossa on posterodorsal surface of transverse processes in middle to posterior cervical vertebrae: 0 absent; 1 present as a fossa; 2 fossa is invasively pneumatized ORDERED (Wang et al., 2017: C374)
519. **Height of cervical epipophyses: 0 at or below height of neural spine without hypertrophy; 1 above height of neural spine, hypertrophied, and sometimes dorsolaterally directed in mid-cervicals (Wang et al., 2017: C375)**
520. Anterior prongs on postaxial cervical epipophyses in C5-C9: 0 absent; 1 present (Wang et al., 2017: C373)

### Posterior cervical & anterior dorsal vertebrae

521. Posterior cervical centrum (8-10 in theropods) length: 0 roughly twice as long as the width of the anterior centrum face or more; 1 less than 1.5 times width of the anterior centrum face (Wang et al., 2017: C379)
522. Centra of cervicodorsal vertebrae with convex anterior articular surface: 0 perimeter of anterior articular surface not rimmed by a flattened peripheral band; 1 flat, forming a distinct rim (Wang et al., 2017: C368)
523. Posterior cervical and/or anteriormost dorsal vertebrae, anterior tubercle on anterior articular surface (best visible in ventral view) [inapplicable for taxa without anteriorly convex centrum]: (0) absent; (1) present (new)
524. Posteriormost cervical and/or anteriormost dorsal vertebrae, pneumatic recesses (pleurocoels) in the centrum [inapplicable to taxa that lack cervical pleurocoels altogether]: (0) present; (1) absent (new)
525. Carotid process on posterior cervical centra: 0 absent; 1 present (Wang et al., 2017: C359)
526. Bifurcation of the neural spine in posteriormost cervical vertebrae: 0 absent; 1 present (Wang et al., 2017: C381)
527. **Posterior cervical vertebrae, neural spines, height (measured from the lowest point of the spinopostzygapophyseal lamina to the top of the neural spine) compared to centrum height in posterior view: (0) < 30%; (1) 30 - 100%; (2) > 100% ORDERED (modified from Rauhut & Pol 2021: C 365)**
528. Posterior cervical vertebrae, neural spines, anteroposterior length (measured from the dorsal end of the spinopostzygapophyseal lamina anteriorly towards the prespinal fossa), compared to posterior height (measured as in the previous character): (0) highly elongated, ratio > 250%; (1) moderately elongated; (2) very short, much less than 50% ORDERED (new)
529. Posteriormost cervical and anterior dorsal vertebrae, neural spines, relative mediolateral width compared to anteroposterior length: (0) much less, spines more or less sheet-like; (1) subequal or more, spines robust; (2) considerably more, spine wider mediolaterally than long anteroposteriorly ORDERED (new)
530. Postaxial cervical and anterior dorsal vertebrae, fossae on dorsal surface of neural arch lateral to the spinoprezygapophyseal lamina posterior to prezygapophysis: (0) absent; (1) present (modified from Rauhut & Pol 2021: C371)
531. Hyposphene-hypantrum articulation in dorsal vertebrae and/or posterior cervicals: 0 absent; 1 present (Wang et al., 2017: C395)

### Cervical ribs

532. **Cervical ribs with anteroposteriorly elongate craniolateral processes: 0 absent; 1 present (Wang et al., 2017: C440)**
533. **Shaft of cervical ribs: 0 broad and shorter than vertebra; 1 slender and longer than vertebra to which they articulate; 2 anteroposteriorly longer than the articulating vertebra and the posteriorly adjacent vertebra combined ORDERED (Wang et al., 2017: C442)**
534. Mid-cervical ribs bifurcate distally into rib and styliform process: 0 absent; 1 present (Wang et al., 2017: C441)
535. **Posterior cervical ribs and centra: 0 separate; 1 fused (Wang et al., 2017: C443)**

## Dorsal Series

### General (No section specified in the original characters!)

536. Shape of dorsal centra in anterior view: 0 subcircular or oval; 1 significantly wider than high in the anterior dorsals; 2 significantly wider than high throughout the dorsal column (Wang et al., 2017: C387)
537. **Pneumatic foramina in the cortical surfaces of the centra in dorsal vertebrae: 0 absent; 1 present in anterior dorsals (pectorals); 2 present in anterior dorsals + mid to posterior dorsals (past 4th dorsal) ORDERED (Wang et al., 2017: C384)**
538. Dorsal vertebrae, morphology of neural spines: 0 transversely compressed sheets; 1 transversely broad anteriorly and posteriorly, central regions of lateral surface embayed by deep vertical troughs (Wang et al., 2017: C382)
539. **Notarium of dorsal vertebrae (fusion of two or more vertebrae near the shoulder joint): 0 absent; 1 present (Wang et al., 2017: C409)**

### Anterior dorsal vertebrae

540. Parapophyseal facets of anterior dorsal vertebrae: 0 moderate in size (less than half height of centrum); 1 hypertrophied (greater than two thirds centrum height) (Wang et al., 2017: C394)
541. **Hypapophyses in anterior dorsals: 0 absent or poorly developed; 1 pronounced (Wang et al., 2017: C383)**
542. Anterior dorsal vertebrae, centrum, morphology of ventral surface: (0) mediolaterally rounded, or with weakly developed keel; (1) with pronounced ventral keel; (2) with anteriorly placed depression defined by lateroventral ridges (modified from Rauhut & Pol 2021: C385)
543. **Dorsal vertebrae, anterior, size of pneumatic foramen in centrum: 0 small, < 50% anteroposterior length of centrum; 1 enlarged, >50% anteroposterior length of centrum (Wang et al., 2017: C408)**
544. Anterior dorsal vertebrae, height of neural arch (as measured from the floor of the neural canal to the roof of the transverse process): 0 less than height of centrum; 1 hypaxially inflated, height subequal to or greater than centrum height (modified from Rauhut & Pol 2021: C390)
545. Anterior dorsal vertebrae, anterior and posterior infrazygapophyseal fossae: 0 single; 1 with one or more accessory centrodiapophyseal laminae dividing fossa into multiple chambers (Wang et al., 2017: C392)
546. Transverse processes of anterior dorsal vertebrae: 0 subhorizontal to dorsally inclined; 1 pendant (Wang et al., 2017: C393)
547. Transverse processes of anterior dorsal vertebrae: 0 proximodistally long and anteroposteriorly thin; 1 proximodistally short, anteroposteriorly wide (Wang et al., 2017: C406)

### Middle and posterior dorsal vertebrae

548. Mid to posterior dorsal vertebrae, centra, articular surfaces: (0) amphiplatyan; (1) middle dorsals opisthocoelous, posterior dorsals amphyplatyan; (2) opisthocoelus up to the sacrum ORDERED (modified from Rauhut & Pol 2021: C384)
549. Mid-dorsal vertebrae, centrum, morphology of ventral surface: (0) vertebrae transversally rounded; (1) transversely flattened with midline sulcus; (2) keel present at least as a midline ridge (new)
550. **Posterior dorsal vertebrae: 0 very short, centra much shorter anteroposteriorly than dorsoventrally tall; 1 relatively short, centra approximately as tall as long, or only slightly longer; 2 much longer than high ORDERED (Wang et al., 2017: C388)**
551. Posterior dorsal vertebrae, centrum, morphology of ventral surface: (0) transversely convex; (1) transversely flattened, sometimes with very shallow midline sulcus; (2) low midline ridge present in at least the anterior section (modified from Rauhut & Pol 2021: C399)
552. Posterior dorsal prezygapophyses: 0 steeply inclined mediodorsally (angle from the horizontal > 30°); 1 subhorizontal (angle < 30°, > 0°); 2 slightly inclined laterodorsally ORDERED (modified from Farke & Sertich, 2013)
553. **Paradiapophyseal lamina in mid-dorsal vertebrae: 0 absent or weak; 1 pronounced (Wang et al., 2017: C407)**
554. Parapophyses of posterior trunk vertebrae: 0 flush with neural arch or only slightly projecting; 1 project far laterally, sometimes visible lateral to diapophyses in dorsal view (Wang et al., 2017: C404)
555. Parapophyses in posteriormost dorsals: 0 on same level as transverse process; 1 distinctly below transverse process (Wang et al., 2017: C405)
556. **Neural spines of posterior dorsals: 0 broadly rectangular and approximately as dorsoventrally high as anteroposteriorly long; 1 high rectangular, significantly dorsoventrally higher than anteroposteriorly long; 2 very tall, more than 3 times as tall as wide ORDERED (Wang et al., 2017: C402)**
557. Mid to posterior dorsal vertebrae, neural spines, basal anteroposterior expansion: (0) absent; (1) present (new)
558. Posterior dorsal and sacral vertebrae, neural spines, shape in lateral view: (0) rectangular or square; (1) anteroposteriorly expanded distally, fan-shaped; (2) expand gradually dorsally, funnel-shaped (modified from Rauhut & Pol: C400)
559. Posterior dorsal vertebrae, orientation of neural spines: 0 vertically or posteriorly; 1 anteriorly (Wang et al., 2017: C390)
560. Posterior dorsal vertebrae, neural spine, basal webbing between neural spine and transverse process: (0) absent; (1) present (modified from Rauhut & Pol 2021: C389)
561. Neural spines of dorsal vertebrae in dorsal view: 0 not expanded distally; 1 expanded laterally in dorsal view to form "spine table" (Wang et al., 2017: C400)
562. Hook-like extension on anterior end of dorsal neural spines in lateral view: 0 absent; 1 present (with associated depression immediately caudal to the projection for interspinous ligament attachment) (Wang et al., 2017: C403)
563. **Scars for interspinous ligaments: 0 terminate at apex of neural spine in dorsal vertebrae; 1 terminate below apex of neural spine (Wang et al., 2017: C401)**
564. **Mid to posterior dorsal vertebrae, postzygapophyses, dorsoventral position: (0) level with the posterior base of the transverse process; (1) offset ventrally from the level of the posterior base of the transverse process (new)**
565. **Step-like ridge lateral to hyposphene running posterodorsally from the dorsal border of the neural canal to the posterior edge of the postzgyapophyses of dorsal vertebrae (visible in lateral view): 0 absent; 1 present (Wang et al., 2017: C396)**
566. Postzygapophyses of dorsal vertebrae: 0 abutting one another above neural canal, opposite hyposphenes meet to form lamina; 1 zygapophyses placed lateral to neural canal and separated by groove for interspinous ligaments, hyposphenes separated (Wang et al., 2017: C398)
567. Mid to posterior dorsal vertebrae, postzygapophyses, shape of articular surface in posterior view: (0) straight to slightly mediolaterally concave; (1) markedly concave with ventrolaterally flexed lateral margins (modified from Rauhut & Pol 2021: C397)
568. Mid to posterior dorsal vertebrae, lateral rims of the postzygapophyses and postzygodiapophyseal lamina: (0) confluent or only distinguished by a slight concavity; (1) separated by a deep, U-shaped incision that is visible in dorsal (in taxa with level postzygapophyses and transverse processes) or posterior view (in taxa that have a ventrally offset postzygapophysis) (new)

### Ribs, gastralia and ossified tendons

569. **Ossified sternal ribs: 0 absent; 1 present (Wang et al., 2017: C447)**
570. Cross section of proximal ends of dorsal ribs: 0 subcircular or subquadrangular (intercostal ridge may be present anteriorly or posteriorly); 1 T-shaped, anterior and posterior intercostal ridges both present and strongly projecting (Wang et al., 2017: C444)
571. Dorsal ribs, pneumatic opening in the angle of the capitulum and tuberculum, visible in anterior view: (0) absent; (1) present (modified from Novas et al., 2013)
572. **Ossified uncinate processes: 0 absent; 1 present and free; 2 present and fused ORDERED (Wang et al., 2017: C446)**
573. Lateral gastral segment: 0 shorter than medial one in each arch; 1 distal segment longer than proximal segment (Wang et al., 2017: C448)
574. **Thin, rod-like epaxial ossified tendons at the level of the dorsal, sacral, or caudal vertebrae: 0 absent; 1 present (Wang et al., 2017: C445)**

## Sacral Series

575. Number of vertebrae incorporated into sacrum: 0 two; 1 three; 2 four or five; 3 six; 4 seven or eight; 5 nine or more ORDERED (Wang et al., 2017: C411)
576. Foramina in cortical surfaces of centra of sacral vertebrae: 0 absent; 1 present on only some sacrals; 2 present on all sacrals ORDERED (Wang et al., 2017: C412)
577. **Transverse dimensions of mid-sacral centra relative to other sacral centra: 0 subequal; 1 mediolaterally narrower (Wang et al., 2017: C414)**
578. Sacral vertebrae, orientation of ventral margin of middle centra: 0 approximately horizontal; 1 strongly arched (Wang et al., 2017: C410)
579. Ventral surface of posterior sacral centra: 0 convex; 1 flattened ventrally, sometimes with shallow sulcus; 2 centrum strongly constricted transversely, ventral surface keeled (Wang et al., 2017: C413)
580. Last sacral centrum: 0 with flat posterior articulation surface; 1 convex articulation surface (Wang et al., 2017: C416)
581. **Sacral vertebrae: 0 with unfused zygapophyses; 1 with fused zygapophyses forming a sinuous ridge in dorsal view (Wang et al., 2017: C415)**
582. **Sacral vertebrae, pneumaticity of neural arches: 0 weak or absent; 1 paired fossae ventral to diapophyses (Wang et al., 2017: C419)**
583. **Fenestrae between neural spines of sacral vertebrae: 0 absent; 1 present (Wang et al., 2017: C417)**
584. Posteriormost sacral and anterior caudal vertebrae, accessory lamina in postzygocentrodiapophyseal fossa extending from roof of neural canal to the transverse process: (0) absent; (1) present (new)
585. **Sacral ribs: 0 slender and well-separated; 1 forming a more or less continuous sheet in ventral or dorsal view; 2 very massive and strongly expanded (Wang et al., 2017: C418)**

## Caudal Series

### Caudal vertebrae

586. Number of caudal vertebrae: 0 8 or less; 1 9-19; 2 20-25; 3 26-40; 4 41-60; 5 > 60 ORDERED (Wang et al., 2017: C420)
587. Caudal centra: 0 amphiplatyan; 1 procoelous (Wang et al., 2017: C423)
588. Caudal vertebrae: 0 with distinct transition point, from shorter centra with long transverse processes proximally to longer centra with small or no transverse processes distally; 1 homogeneous in shape, with no transition point (Wang et al., 2017: C427)
589. Position of transition point: 0 distal to the tenth caudal vertebra; 1 between the 7th and 10th caudal vertebrae; 2 proximal to the 7th caudal vertebra ORDERED (Wang et al., 2017: C428)
590. Neural spines of caudal vertebrae: 0 simple, undivided; 1 separated into anterior and posterior alae throughout much of caudal sequence (Wang et al., 2017: C432)
591. **Degree of fusion of distal caudal vertebrae: 0 fusion absent; 1 few vertebrae partially ankylosed (intervening elements are well-discernable); 2 vertebrae completely fused into a pygostyle (Wang et al., 2017: C421)**
592. Shape of anterior caudal centra: 0 oval; 1 subrectangular and box-like; 2 laterally compressed with a ventral keel (Wang et al., 2017: C424)
593. **Ventral surface of anterior caudals: 0 convex; 1 with a distinct keel sometimes bearing a narrow, shallow groove on its midline; 2 grooved without a keel (Wang et al., 2017: C425)**
594. Anterior to mid-caudal vertebrae, centra, depressions or pneumatic foramina: (0) absent; (1) large, pronounced pleurocentral depressions on the dorsal part of the lateral side; (2) pneumatic foramina present (modified from Wang et al., 2017: C422 according to Rauhut et al. 2016)
595. Location of transverse processes of proximal caudals: 0 centrally positioned on centrum; 1 anteriorly displaced (Wang et al., 2017: C429)
596. Transverse processes of anterior caudals: 0 subhorizontal or slightly upturned; 1 steeply angled (> ~35-40 degrees) (Wang et al., 2017: C437)
597. Distal expansion of transverse processes in proximal to middle caudals: 0 absent; 1 craniocaudal expansion present (Wang et al., 2017: C439)
598. Ventrodistal bony ridge on the distal end of the transverse processes in anterior caudal vertebrae: 0 absent; 1 present (Wang et al., 2017: C438)
599. Anterior caudal vertebrae, anterior centrodiapophyseal lamina: (0) absent; (1) present as low ridge; (2) present as a lamina ORDERED (modified from Rauhut & Pol : C432)
600. Anterior caudal vertebrae, posterior centrodiapophyseal lamina: (0) absent; (1) present as low ridge; (2) present as a lamina ORDERED (modified from Rauhut & Pol : C432)
601. **Anterior caudal vertebrae, pneumatic foramen in prezygapophyseal centrodiapophyseal fossa: (0) absent; (1) present (modified from Rauhut & Pol : C432)**
602. **Anterior caudal vertebrae, pneumatic foramen in centrodiapophyseal fossa: (0) absent; (1) present (modified from Rauhut & Pol : C432)**
603. **Anterior caudal vertebrae, pneumatic foramen in postzygapophyseal centrodiapophyseal fossa: (0) absent; (1) present (modified from Rauhut & Pol : C432)**
604. Anterior to mid caudal vertebrae, neural spine, depression on lateral side of base of neural spine, defined anteromedially by the spinoprezygapophyseal lamina: (0) absent; (1) present (modified from Rauhut & Pol 2021: C389)
605. Spinodiapophyseal ridge or lamina in anterior and mid-caudal vertebra: 0 absent; 1 present (new character)
606. Hyposphene-hypantrum articulations in proximal to mid-caudal vertebrae: 0 absent; 1 present (Wang et al., 2017: C436)
607. Mid-caudal vertebrae, lateral ridge extending anteriorly from the dorsal surface of the base of the transverse process: (0) absent; (1) present (Rauhut 2011)
608. Mid-caudal vertebrae, lateral ridge extending posteriorly from the base of the transverse process towards the posterodorsal end of the vertebral centrum: (0) absent; (1) present (new)
609. Mid-caudal vertebrae, anterior entrance of neural canal: (0) simple, broad, anteriorly directed rim; (1) laterally flared, funnel-like (Rauhut 2011)
610. Neural spines of mid-caudals: 0 elongate plate-like and posteriorly inclined; 1 rod-like (approximately as broad transversely as long anteroposteriorly) and posteriorly inclined; 2 anteroposteriorly short and vertical; 3 subrectangular and sheet-like; 4 inconspicuous low midline ridge or absent (modified from Wang et al., 2017: C433)
611. Anterior margin of neural spines of anterior mid-caudal vertebrae: 0 straight; 1 with distinct kink, dorsal part of anterior margin more strongly inclined posteriorly than ventral part (Wang et al., 2017: C435)
612. Postzygapophyses in posterior middle and anterior distal caudal vertebrae: 0 placed immediately below the base of the neural spine; 1 project posteriorly from the base of the neural spine (new character)
613. Relative length of distal caudal centra: 0 much longer than centrum height; 1 subequal in length to centrum height (Wang et al., 2017: C426)
614. **Prezygapophyses of distal caudal vertebrae: 0 between 1/3 and whole centrum length; 1 with extremely long extensions of the prezygapophyses (up to 10 vertebral segments in some taxa); 2 very short anteroposteriorly, as in Archaeopteryx lithographica (Wang et al., 2017: C434)**
615. Neural spines on distal caudals: 0 form a low ridge; 1 spine absent; 2 midline sulcus in center of the neural arch ORDERED (Wang et al., 2017: C431)

### Chevrons

616. Cranial process at base of chevrons: 0 absent; 1 present (Wang et al., 2017: C449)
617. Proximal surface of chevrons: 0 distinct transverse ridge dividing surface into anterior and posterior facets; 1 no ridge, low mounds may be present laterally (Wang et al., 2017: C450)
618. Proximal end of chevrons of proximal caudals: 0 short anteroposteriorly, shaft cylindrical; 1 proximal end long anteroposteriorly, flattened and plate-like (Wang et al., 2017: C451)
619. Mid-caudal chevrons: 0 rod-like or only slightly expanded ventrally; 1 L-shaped (Wang et al., 2017: C452)
620. Distal chevrons: 0 rod-like or L-shaped; 1 skid-like (Wang et al., 2017: C453)
621. Distal caudal chevrons: 0 simple; 1 anteriorly bifurcate; 2 bifurcate at both ends ORDERED (Wang et al., 2017: C454)

# Appendicular skeleton

## Pectoral girdle

### Sternum

622. **Ossified sternal plates: 0 separate in adults; 1 fused (Wang et al., 2017: C457)**
623. Anterior edge of sternum: 0 grooved for reception of coracoids; 1 without grooves (Wang et al., 2017: C464)
624. Articular facet of coracoid on sternum: 0 anterolateral or more lateral than anterior; 1 almost anterior (Wang et al., 2017: C463)
625. Sternal midline, caudal end: 0 blunt W-shape; 1 V-shape; 2 elongate straight projection (xiphoid process); 3 flat; 4 rounded (Wang et al., 2017: C455)
626. **Ventral midline keel on sternum: 0 absent; 1 present (Wang et al., 2017: C458)**
627. Sternum: 0 without distinct lateral xiphoid process posterior to costal margin; 1 with lateral xiphoid process (Wang et al., 2017: C459)

### Clavicles/Furcula

628. Clavicles: 0 present and unfused; 1 fused into a furcula (Wang et al., 2017: C460)
629. Furcula shape: 0 v-shaped; 1 u-shaped, with bowed epicleidea (Wang et al., 2017: C461)
630. Furcula interclavicular angle: 0 obtuse, >90 degrees; 1 between 90 and 70 degrees; 2 less than 70 ORDERED (Wang et al., 2017: C456)
631. **Hypocleidium on furcula: 0 absent; 1 present (Wang et al., 2017: C462)**

### Scapulacoracoid

632. **Scapula and coracoid: 0 separate; 1 fused to form scapulocoracoid (Wang et al., 2017: C485)**
633. Scapula and coracoid orientation: 0 continuous arc in posterior and anterior views; 1 coracoid inflected medially, scapulocoracoid L-shaped in anterior view (Wang et al., 2017: C486)
634. Scapula articulation: 0 articulated at the shoulder (proximal) end of the coracoid; 1 well below it (Wang et al., 2017: C480)
635. Glenoid fossa: 0 faces posteriorly or posterolaterally; 1 faces laterally (Wang et al., 2017: C484)
636. **Scapula length: 0 longer than humerus; 1 shorter than humerus (Wang et al., 2017: C487)**
637. **Scapula shape: 0 extremely broad (ratio length/minimal height of shaft <5); 1 short and broad (ratio length/minimal height of shaft 5<x<9); 2 slender and long (ratio >10) ORDERED (Wang et al., 2017: C472)**
638. Distal end of scapula: 0 expanded; 1 not expanded (Wang et al., 2017: C483)
639. Anterodorsal scapular flange: 0 absent; 1 present (Wang et al., 2017: C478)
640. **Scapula, midshaft expansion of blade: 0 absent; 1 present (Wang et al., 2017: C479)**
641. Scapula, medial surface, ridge along the long axis of the scapular shaft towards the coracoid articulation: (0) absent, no fossa present on either side; (1) defined mainly by a fossa on the dorsal side; (2) defined by longitudinal fossae dorsally and ventrally
642. **Acromion margin of scapula: 0 continuous with blade; 1 anterior edge enlarged and projects anteriorly at approximately a right angle (Wang et al., 2017: C481)**
643. **Scapula, size of acromion process: 0 moderate, distance from tip of acromion to scapular shaft equal or less than width of scapular shaft; 1 marked, distance from tip of acromion to scapular shaft more than width of scapular shaft (Wang et al., 2017: C477)**
644. Scapulocoracoid junction anterior surface: 0 indented or notched between the scapular acromial process and the coracoid suture; 1 smoothly curved and uninterrupted across the contact between the scapula and coracoid (Wang et al., 2017: C473)
645. Scapula and coracoid junction, preacromial groove: 0 absent; 1 moderate groove present; 2 very deep groove present ORDERED (Wang et al., 2017: C474)
646. Flange on supraglenoid buttress on scapula: 0 absent; 1 present (Wang et al., 2017: C482)
647. **Coracoid in lateral view: 0 subcircular, with low ventral blade and no or small posterior process; 1 shallow ventral blade with long posterior process; 2 subquadrangular with extensive ventral blade; 3 strut-like, very tall ventral blade with little or no posterior process ORDERED** (Wang et al., 2017: C466)
648. Procoracoid process on coracoid: 0 absent; 1 present (Wang et al., 2017: C465)
649. Anterior surface of coracoid ventral to glenoid fossa: 0 unexpanded; 1 expanded, forms triangular subglenoid fossa bounded laterally by coracoid tuber (Wang et al., 2017: C468)
650. Ratio of glenoid height to pre-glenoid length of the coracoid: 0 >0.25; 1 less than or equal to 0.25 (Wang et al., 2017: C476)
651. **Posterior edge of coracoid: 0 not or shallowly indented below glenoid; 1 posterior edge of coracoid deeply notched just ventral to glenoid, glenoid lip everted (Wang et al., 2017: C467)**
652. Coracoid foramen: 0 present; 1 absent (Wang et al., 2017: C471)
653. **Coracoid tubercle: 0 absent; 1 present (Wang et al., 2017: C469)**
654. Coracoid tubercle form: 0 anteroposteriorly short, mound-like; 1 anteroposteriorly long, ridge-like (Wang et al., 2017: C470)
655. **Coracoid, glenoid, lateral lip: (0) absent or indistinct; (1) marked as a distinct, laterally directed ridge, also visible in posteroventral view (modified from Rauhut & Pol 2021: C477**)

## Forelimbs

### Humerus

656. **Ratio femur/humerus: 0 more than 2.5; 1 between 1.2 and 2.2; 2 1 or less ORDERED (Wang et al., 2017: C497)**
657. **Humeral robusticity: 0 Total length/midshaft width in anterior view <10; 1 Total length/midshaft width in anterior view = 10 or more (Wang et al., 2017: C488)**
658. **Humerus, shape in anterior view: (0) straight; (1) bowed laterally, lateral side slightly convex and medial side notably concave (modified from Carrano et al. 2012: C236)**
659. Humerus in lateral view: 0 sigmoidal; 1 straight (Wang et al., 2017: C501)
660. Humerus, relative orientation of proximal & distal condyles in anteroposterior view: (0) subparallel; (1) distal condyles notably canted medially (modified from Rauhut & Pol 2021: C494)
661. Outline of proximal articular facet of humerus: 0 broadly oval (more than twice as broad transversely than anteroposteriorly); 1 distinctly convex, often globular (less than twice as broad anteroposteriorly than transversely) (Wang et al., 2017: C498)
662. **Internal tuberosity of humerus: 0 small and confluent with humeral head; 1 offset from humeral head by distinct notch, often projects proximally above humeral head; 2 hypertrophied but not distinct from humeral head (as in *Baryonyx walkeri*) (Wang et al., 2017: C499)**
663. Shape of internal tuberosity on humerus in anterior view: 0 triangular, often rounded; 1 rectangular (Wang et al., 2017: C500)
664. Humerus, proximal end, pronounced longitudinal groove on the posteromedial side: (0) absent; (1) present (new)
665. Humerus, greater tubercle: (0) indistinct and confluent with humeral head; (1) forms a pronounced proximolateral edge and slightly separated from humeral head by a slightly concave surface; (2) forms a pronounced proximolateral edge and strongly separated from humeral head by a marked concavity (see Cuesta et al. 2018)
666. Humerus, greater tubercle, placement: (0) lateral to humeral head near proximal end; (1) offset distally from humeral head towards the deltopectoral crest (Carrano & Sampson 2008)
667. Deltopectoral crest: 0 large and distinct, proximal end of humerus quadrangular in anterior view; 1 less pronounced, forming an arc rather than being quadrangular; 2 very weakly developed, proximal end of humerus with convex edges; 3 extremely long anteroposteriorly (Wang et al., 2017: C494)
668. **Deltopectoral crest length: 0 Approximately one quarter humeral length or less; 1 approximately one third humeral length; 2 greater than one half humeral length ORDERED (Wang et al., 2017: C493)**
669. Humerus, deltopectoral crest, projection: 0 anteriorly or ventrally directed; 1 laterally deflected (Wang et al., 2017: C489)
670. Deltopectoral crest orientation: 0 longitudinal; 1 oblique distolaterally and distal end of crest oriented laterally rather than anteriorly from the humeral shaft (Wang et al., 2017: C495)
671. Lateral surface of distal end of deltopectoral crest: 0 smooth; 1 with distinct muscle scar near lateral edge along distal end of crest for insertion of biceps muscle (Wang et al., 2017: C496)
672. **Humerus, shaft, lateral tubercle distal to deltopectoral crest: (0) absent; (1) present (new)**
673. **Transverse width of distal humerus: 0 greater than 2.7 times shaft width; 1 between 2 and 2.5 times humeral shaft width; 2 less than twice shaft width ORDERED (Wang et al., 2017: C502)**
674. Well-developed olecranon fossa on the caudal face of the distal end of the humerus: 0 absent; 1 present (Wang et al., 2017: C490)
675. Distal humeral condyles: 0 rounded; 1 flat (Wang et al., 2017: C491)
676. Distal humeral condyles: 0 primarily developed on distal end of humerus, but may have some articular surface extending to anterior edge; 1 limited to anterior surface, condylar surfaces not present on distal end (Wang et al., 2017: C505)
677. **Ectepicondyle of humerus (lateral epicondyle): 0 absent; 1 present (modified from Rauhut & Pol 2021: C504)**
678. **Development of ectepicondyle of humerus: 0 moderately developed, proximodistally elongate, often rounded flange, confluent with distal articular surface; 1 rounded anterolateral tubercle, offset from distal articular surface; 2 strongly expanded laterally, with articular surface distally that is offset from main humeral articular surface by a small incision (modified from Rauhut & Pol 2021: C504) inapplicable in taxa that lack an ectepicondyle that is distinct from the radial articular condyle**
679. Entepicondyle of humerus (medial epicondyle): 0 absent; 1 present (modified from Rauhut & Pol 2021: C505)
680. **Development of entepicondyle: 0 anteromedial tubercle, offset from distal articular end; 1 rounded medial projection, confluent distally with the articular surface; 2 expanded, subtriangular, mediodistally pointed, confluent with or only incipiently offset from articular surface; 3 hypertrophied medial flange, offset from distal articular surface (modified from Rauhut & Pol 2021: C505)**

### Ulna

681. Ulnar shaft: 0 straight; 1 bowed (Wang et al., 2017: C506)
682. Proximal surface of ulna: 0 single continuous articular facet; 1 divided into two distinct fossae (Wang et al., 2017: C510)
683. Proximal end of the ulna in proximal view: 0 without extensive coronoid process and radial process on radial side of proximal end; 1 coronoid and radial processes large, at right angles to each other in proximal view (Wang et al., 2017: C511)
684. **Olecranon process of ulna: 0 absent or weakly developed; 1 well-developed; 2 hypertrophied ORDERED (Wang et al., 2017: C507)**
685. Shape of olecranon process: 0 transversely broad; 1 mediolaterally thin, blade-like (Wang et al., 2017: C508)
686. Crest extending along posterior surface of ulnar shaft from olecranon process: 0 absent; 1 present (Wang et al., 2017: C509)
687. Distal articular surface of ulna: 0 flat; 1 convex, semilunate surface (Wang et al., 2017: C512)
688. Distal condyle articular surface of ulna: 0 unexpanded or spatulate, articular surface limited to distal end; 1 bulbous, trochlear articular surface extends onto dorsal surface of ulna (Wang et al., 2017: C513)

### Radius

689. **Radius length ORDERED: 0 less than half the length of humerus; 1 more than half the length of humerus; 2 longer than the humerus (Wang et al., 2017: C514)**
690. Radial shaft: 0 straight; 1 bowed laterally (Wang et al., 2017: C515)
691. Radius and ulna: 0 well-separated; 1 with distinct adherence or syndesmosis distally (Wang et al., 2017: C516)

## Manus

692. **Manual length (measured as the average length of digit positions I-III): 0 accounts for less than 0.3 of the total length of humerus plus radius; 1 more than 0.3 but less than 0.4 of the total length of humerus plus radius; 2 more than 0.4 of the total length of humerus plus radius ORDERED (Wang et al., 2017: C522)**
693. Number of manual digits with one or more phalanges: 0 five; 1 four; 2 three; 3 two ORDERED (Wang et al., 2017: C545)
694. Longest digit of the manus, including metacarpals: 0 third position; 1 second and third roughly equal; 2 second position; 3 first position (present in some Alvarezsauroidea) (Wang et al., 2017: C524)
695. Unguals on all manual digits: 0 generally similar in size; 1 digit I bearing large ungual and unguals of other digits distinctly smaller (Wang et al., 2017: C558)

### Carpals

696. **Ossified carpals: 0 absent; 1 present (Wang et al., 2017: C517)**
697. Lateral proximal carpal (ulnare?): 0 quadrangular; 1 triangular in proximal view (Wang et al., 2017: C518)
698. Trochlea on the proximal surface of distal carpal 1: 0 absent; 1 present (Wang et al., 2017: C519)
699. Distal carpals: 0 two distal carpals in contact with metacarpals, one covering the base of metacarpal I (and perhaps contacting metacarpal II), the other covering the base of metacarpal II; 1 single distal carpal capping metacarpal I and II (Wang et al., 2017: C520)
700. **Semilunate carpal and proximal ends of metacarpals in adults: 0 unfused; 1 semilunate fused to the alular (I) metacarpal; 2 semilunate fused to the major (II) and minor (III) metacarpals; 3 fusion of semilunate and all metacarpals (Wang et al., 2017: C521)**

### Metacarpals

701. Number of metacarpals: 0 five; 1 four; 2 three ORDERED (Wang et al., 2017: C546)
702. **Longest metacarpal/Longest metatarsal: 0 greater than 0.5; 1 less than 0.5 (Wang et al., 2017: C523)**
703. **Distal end of metacarpals: 0 unfused; 1 partially or completely fused to each other (Wang et al., 2017: C525)**
704. **Extensor pits on the dorsal surface of the distal end of metacarpals: 0 absent or poorly developed; 1 deep, well-developed (Wang et al., 2017: C544)**
705. Length of first position metacarpal: 0 approximately half the length of second position metacarpal; 1 subequal in length to 2nd position (Wang et al., 2017: C531)
706. Shape of first position metacarpal: 0 significantly longer than broad; 1 very stout, approximately as long as broad (Wang et al., 2017: C532)
707. **Metacarpal I, shaft, flexion: (0) straight or slightly flexed; (1) with pronounced medial inflection distal to the end of the articular facet for mc II (New)**
708. Contact between first and second position metacarpals: 0 metacarpals contact each other at their bases only; 1 metacarpal II closely appressed to metacarpal III, at least the proximal half of metacarpal II flattened (Wang et al., 2017: C533)
709. Medial tab on proximal end of first position metacarpal: 0 absent or poorly developed; 1 well-developed, extending far medially (Wang et al., 2017: C534)
710. Tab on dorsomedial (ulnar) aspect of medial most metacarpal with phalanges, overlapping adjacent metacarpal: 0 absent; 1 present (Wang et al., 2017: C526)
711. Rectangular buttress on ventrolateral surface of proximal end of first position metacarpal: 0 absent; 1 present (Wang et al., 2017: C529)
712. Ventrolateral tab on the proximolateral surface of first position metacarpal: 0 absent; 1 present (Wang et al., 2017: C530)
713. Distal articular end of first position metacarpal: 0 ginglymoid; 1 convex and smooth (Wang et al., 2017: C536)
714. Asymmetry of distal condyles of metacarpal on second manual digit counting medial to lateral: 0 absent; 1 present (Wang et al., 2017: C527)
715. Distal end of first position metacarpal: 0 condyles more or less symmetrical; 1 condyles strongly asymmetrical, the medial condyle being positioned more proximally than the lateral (Wang et al., 2017: C535)
716. **Prominent tendon/ligament attachment on the dorsal surface of the second position metacarpal immediately proximal to the distal condyles/extensor ligament pits: 0 absent; 1 present (Wang et al., 2017: C528)**
717. Medial side of second position metacarpal: 0 expanded proximally; 1 not expanded (Wang et al., 2017: C537)
718. Distal articular end of second position metacarpal: 0 ginglymoid; 1 convex and smooth (Wang et al., 2017: C538)
719. Shaft of third position metacarpal: 0 subequal in width to second position metacarpal; 1 considerably slender than second position metacarpal (less than 70% of the width of second position metacarpal); 2 splint-like (less than 40% of the width of metacarpal III) ORDERED (Wang et al., 2017: C540)
720. Proximal articular end of third position metacarpal: 0 expanded and similar in width to metacarpal II and III; 1 not expanded, very slender when compared to metacarpal II and III (Wang et al., 2017: C541)
721. Proximal outline of third position metacarpal: 0 subrectangular; 1 triangular, apex dorsal (Wang et al., 2017: C542)
722. Shaft of third position metacarpal: 0 straight; 1 bowed laterally (Wang et al., 2017: C543)
723. **Distal condyles/articular surface of third position metacarpal: 0 symmetrical; 1 asymmetrical (Wang et al., 2017: C539)**

### Phalanges

724. Paired flexor processes on proximal ventral surfaces of proximalmost phalanges: 0 absent; 1 present (Wang et al., 2017: C547)
725. Collateral Ligament Pits on non-ungual phalanges of the manus: 0 well developed; 1 reduced or absent (Wang et al., 2017: C548)
726. Distal articular surface of proximal manual phalanges: 0 symmetrical or weakly asymmetrical; 1 strongly asymmetrical, lateral condyles project further distally than medial condyles (Wang et al., 2017: C549)
727. Ratio length phalanx I-1/metacarpal I: 0 1 or less; 1 between 1 and 1.5; 2 more than 1.5 ORDERED (Wang et al., 2017: C552)
728. **Shaft diameter of phalanx I-1: 0 less than shaft diameter of radius; 1 greater than shaft diameter of radius (Wang et al., 2017: C551)**
729. Flexor surface of manual phalanx I-1: 0 convex or flat; 1 concave, 'axial furrow' along proximodistal axis (Wang et al., 2017: C550)
730. Manual phalanx II-1, proximal side of ventral surface: (0) flat; (1) with broad, shallow depression, defined by slightly raised medial and lateral edges; (2) with longitudinal sulcus defined by medial and lateral flanges (new)
731. Manual phalanx II-1, expanded medioventral process on proximal end: (0) absent, proximal articular facet medioventrally rounded; (1) present, medioventral edge of articular surface forms a sharp angle (new)
732. Manual phalanx II-1, distal articular end: (0) more or less symmetrical, both condyles extend subequally distally; (1) markedly asymmetrical, lateral condyle projects much further distally, medial condyle mediolaterally flat (new)
733. Second phalanx of the second finger: 0 shorter than first phalanx; 1 longer than first phalanx (Wang et al., 2017: C553)
734. Phalangeal count on the third digit counting medially to laterally: 0 Four; 1 Three; 2 Two; 3 Zero ORDERED (Wang et al., 2017: C556)
735. Penultimate phalanx of the third finger: 0 as long as, or shorter than, more proximal phalanges; 1 longer than each of the more proximal phalanges; 2 longer than both proximal phalanges taken togetherORDERED (Wang et al., 2017: C554)
736. Transverse ridge immediately dorsal to the articulating surface of unguals: 0 absent; 1 present (Wang et al., 2017: C561)
737. Flexor tubercle placement in manual unguals: 0 proximal; 1 distal (Wang et al., 2017: C562)
738. **Flexor tubercle size ORDERED: 0 large (> 1/3 articular facet height); 1 small (< 1/3 articular facet height); 2 absent or very weakly developed (Wang et al., 2017: C565)**
739. Manual unguals, ventral surface: (0) rounded mediolaterally; (1) flattened; (2) narrow, sharp-edged (new character)
740. **Manual ungual of first digit, length (as measured in a straight line from the proximodorsalmost point towards the distalmost point) compared to the maximum proximodistal length of the humerus: (0) < 0.3; (1) 0.3 – 0.4; (2) > 0.4 ORDERED (New)**
741. Proximal articular surface of manual ungual I-2: 0 dorsoventrally much taller than mediolaterally wide; 1 mediolaterally as broad as tall (Wang et al., 2017: C557)
742. Manual ungual of first digit, dorsal margin (excluding proximodorsal lip if present): (0) curves strictly ventrally from the proximal articulation facet (proximodorsal edge is highest point when the articular surface is oriented vertically); (1) curves anterodorsally from the proximal articular facet (highest point of ungual placed more distally when oriented with proximal articulation vertically) (new)
743. Lateral grooves of manual ungual I-2 in ventral view: 0 unenclosed; 1 proximal end of grooves partially enclosed by lateral notches; 2 proximal end of grooves passes through foramina on ventral surface of ungual ORDERED (Wang et al., 2017: C566)
744. Ungual phalanx on the digit in the second position on the manus: 0 present; 1 absent (Wang et al., 2017: C559)
745. Manual ungual of second digit, dorsal margin: (0) curves strictly ventrally from the proximal articulation facet; (1) curves anterodorsally from the proximal articular facet (new)
746. Ungual phalanx on the digit in the third position of the manus: 0 present; 1 absent (Wang et al., 2017: C560)
747. Manual ungual of third digit, dorsal margin: (0) curves strictly ventrally from the proximal articulation facet; (1) curves anterodorsally from the proximal articular facet (new)
748. **Curvature of ventral surface manual ungual I: 0 strongly curved; 1 weakly curved; 2 straight ORDERED (Wang et al., 2017: C563)**
749. **Curvature of ventral surface of manual unguals on the second and third manual digits counting medially to laterally: 0 strongly curved; 1 weakly curved; 2 straight ORDERED (Wang et al., 2017: C564)**

## Pelvic girdle

750. **Pelvis, fusion of ilium and pubis at the peduncle: (0) absent; (1) present**
751. **Pelvis, fusion of ilium and ischium at the peduncle: (0) absent; (1) present**
752. **Pelvis, fusion of ilium and ischium at the distal end of the ischium: (0) absent; (1) present**
753. **Pelvis, fusion of pubis and ischium at the peduncles: (0) absent; (1) present**
754. Pelvis, pubic orientation: (0) propubic; (1) vertical; (2) moderately posteriorly oriented; (3) opisthopubic, pubis parallel to ischium ORDERED (modified from Rauhut & Pol 2021: C614)

### Ilium

755. Ilium, anteriormost point: (0) formed by the pubic peduncle (brachyiliacic condition); (1) formed by the preacetabular process (dolichoiliacic condition) (modified from Rauhut & Pol et al., 2021: C576)
756. Ilium, ratio of anteroposterior length/height above acetabulum (measurement is taken as maximum anteroposterior length vs height from acetabular rim to maximum vertical height of the ilium): 0 x<2.5; 1 2.5<x<4; 2 4<x<5; 3 x>5 (Wang et al., 2017: C567)
757. Iliac pubic peduncle length relative to iliac ischial peduncle: 0 significantly longer than ischial peduncle, ischial peduncle tapering ventrally and without clearly defined articular facet; 1 subequal in length to ischial peduncle, ischial peduncle does not taper distally (this does not include the peg in taxa that have peg and socket articulations of the ischiadic peduncle); 2 anteroposteriorly shorter than the ischial peduncle ORDERED (Wang et al., 2017: C594)
758. **Ilium pneumaticity: 0 little or none; 1 large external pneumatic foramina and internal pneumatic spaces** (Wang et al., 2017: C574)
759. **Ilium, preacetabular process, height compared to postacetabular height [both measured at the bases of the respective blades, excluding cuppedicus fossa, anterior hook or medial brevis shelf]: (0) postacetabular part significantly higher; (1) at least as high as postacetabular blade (modified from Rauhut & Pol 2021: C584)**
760. Preacetabular part of ilium: 0 significantly shorter than postacetabular part; 1 subequal in length to postacetabular part; 2 significantly longer than postacetabular process ORDERED (Wang et al., 2017: C578)
761. Preacetabular portion of ilium: 0 parasagittal; 1 moderately laterally flaring (Wang et al., 2017: C585)
762. Ilium, preacetabular process, anterior margin: (0) shallowly convex or straight; (1) anterior end strongly convex; (2) with a notable concavity in at least the dorsal part of the anterior margin; (3) pointed (modified from Rauhut & Pol 2021: C580 and 581)
763. Ilium, preacetabular process, shape of anteroventral corner [inapplicable for brachyiliac ilia]: (0) broadly rounded; (1) sharply angled (modified from Rauhut & Pol 2021: 579)
764. **Ilium, preacetabular process, significant anteroventral expansion (also called “preacetabular hook”): (0) absent; (1) present (modified from Rauhut & Pol 2021: C 578).**
765. Ilium, preacetabular process, position of the anteroventral corner [inapplicable for taxa without an anteroventral expansion]: (0) at the level of the dorsal margin of the acetabulum or higher; (1) between the dorsal margin of the acetabulum and the articular surface of the pubic peduncle; (2) reaching the ventral end of the pubic peduncle or going beyond it (new) ORDERED.
766. Ilium, preacetabular process, separation of the anteroventral expansion from the pubic peduncle [inapplicable for taxa without an anteroventral expansion]: (0) well separated from pubic peduncle; (1) poorly separated, ventral edge very near pubic peduncle and parallel to its anterior edge (slightly modified from Rauhut & Pol 2021: 574).
767. Ilium, dorsal margin: 0 subhorizontal or gently inclined relative to axis of pubic and ischial contact; 1 rises steeply as it extends anteriorly, at least 30 degree angle from the axis of the pubic and ischial contact (Wang et al., 2017: C575)
768. Dorsal margin of ilium: 0 straight, slightly convex, or slightly concave; 1 distinctly convex as in Ceratosaurus, Eoabelisaurus, and some gravisaurian sauropodomorphs (Wang et al., 2017: C568)
769. **Dorsal margin of iliac blade: 0 indistinct from rest of blade or rugose scar; 1 hypertrophied rim (Wang et al., 2017: C570)**
770. **Ilium, blades above sacrum: (0) vertical, well-separated above sacrum; (1) strongly inclined mediodorsally, almost contacting each other or sacral neural spines at midline; (2) contacting each other and fused to one another (modified from Rauhut & Pol 2021: C593) ORDERED.**
771. Tuber along dorsal edge of ilium, dorsal or slightly posterior to acetabulum: 0 absent; 1 present (Wang et al., 2017: C603)
772. Dorsal margin of postacetabular ala in lateral view: 0 convex or straight; 1 concave, brevis shelf extends posterior to lateral ilium making it appear concave in lateral view (Wang et al., 2017: C604)
773. Ilium, postacetabular ala, shape in lateral view: (0) square; (1) pointed; (2) with dorsally positioned posterior lobe; (3) notched (modified from Wang et al., 2017: C589)
774. Postacetabular blades of ilia in dorsal view: 0 parallel; 1 diverge posteriorly (Wang et al., 2017: C602)
775. Posterior end of postacetabular ala in dorsal view: 0 rounded or squared in dorsal view; 1 lobate, with brevis shelf extending posteriorly beyond posterior end of the postacetabular (Wang et al., 2017: C605)
776. **Vertical ridge on iliac blade above acetabulum: 0 absent; 1 low ridge with associated foramina; 2 well-developedORDERED (Wang et al., 2017: C591)**
777. Vertical ridges on lateral surface of ilium: 0 single; 1 double (Wang et al., 2017: C592)
778. Cuppedicus fossa: 0 absent; 1 present (Wang et al., 2017: C582)
779. **Form of cuppedicus fossa: 0 deep, ventrally concave; 1 fossa shallow or flat, with no lateral overhang (Wang et al., 2017: C583)**
780. Cuppedicus fossa position: 0 ridge bounding fossa terminates rostral to acetabulum or curves ventrally onto anterior end of pubic peduncle; 1 rim extends far posteriorly and is confluent or almost confluent with acetabular rim (Wang et al., 2017: C584)
781. Anterior margin of pubic peduncle: 0 straight or convex; 1 concave (Wang et al., 2017: C596)
782. Articulation facet of pubic peduncle of ilium: 0 facing more ventrally than anteriorly or completely anteroventrally, and without a pronounced kink. No portion facing directly anteriorly; 1 with pronounced kink and anterior part facing almost entirely anteriorly (Wang et al., 2017: C595)
783. Shape of pubic peduncle of ilium: 0 transversely broad and roughly triangular in outline; 1 anteroposteriorly long and narrow (Wang et al., 2017: C593)
784. Supraacetabular crest: 0 absent; 1 present (Wang et al., 2017: C597)
785. Form of supraacetabular crest: 0 Forms hood over femoral head; 1 reduced, not forming hood (Wang et al., 2017: C598)
786. Supraacetabular crest extends nearly to ventral edge of pubic peduncle, and large area of pubic peduncle visible posterior to the crest in lateral view: 0 absent; 1 present (Wang et al., 2017: C599)
787. Morphology between supraacetabular crest and brevis shelf on lateral surface: 0 gap; 1 continuous ridge (Wang et al., 2017: C600, Carrano & Sampson 2008: C118 Fig. 11)
788. **Antitrochanter posterior to acetabulum and supraacetabular crest: 0 absent or poorly developed; 1 prominent (Wang et al., 2017: C601)**
789. Ilium and ischium articulation: 0 flat or slightly concavo-convex; 1 with process projecting into socket in ischium (Wang et al., 2017: C606)
790. Ilium, distinct fossa present for the attachment of the caudifemoralis brevis muscle: 0 absent; 1 present as an embankment on the lateral side of the posterior portion of the ilium; 2 present as a deep fossa on the ventral surface of postacetabular part or ilium ORDERED (Wang et al., 2017: C569)
791. Brevis fossa shape: 0 shelf-like, narrow with subparallel margins; 1 deeply concave, expanded posteriorly with lateral overhang (Wang et al., 2017: C586)
792. Brevis fossa lateral view: 0 Poorly developed adjacent to ischial peduncle, without lateral overhang and medial brevis fossa is visible: 1 well-developed fossa along full length of postacetabular blade, lateral overhang edge extends along full length of fossa, medial edge of brevis fossa covered in lateral view (Wang et al., 2017: C587)
793. Medial brevis shelf: 0 strongly developed, projects medially; 1 low ridge on medial surface of postacetabular (Wang et al., 2017: C588)

### Pubis

794. **Articulated pubes in anterior view: 0 with straight lateral margins, narrowing gradually distally; 1 with convex proximal and concave distal lateral margins, resulting in a marked inflection below the proximal part (new)**
795. Pubis, articulation with ilium: 0 planoconcave; 1 peg and socket (Wang et al., 2017: C607)
796. Pubis, dorsoventrally extensive, lateromedially narrow flange below the (lateromedially thickened portion of the) ischial peduncle: (0) absent; (1) present (modified from Rauhut & Pol 2021: C611)
797. Pubis, obturator foramen: (0) completely enclosed; (1) open ventrally, developed as a notch; (2) completely open, foramen absent. ORDERED (modified from Rauhut & Pol 2021 C624 & 625)
798. Pubis, obturator foramen, shape in lateral view [inapplicable for taxa with a ventrally open, or absent obturator foramen]: (0) round or slightly elongated; (1) significantly elongated, long axis approximately 2 times longer than short axis or more (modified from Rauhut & Pol 2021: C626)
799. Pubis, obturator foramen, lateral swelling on the dorsal part of the ischial peduncle, overhanging the obturator foramen [inapplicable for taxa with a ventrally open, or absent obturator foramen]: (0) present; (1) strongly reduced or absent (new)
800. Pubis, pubic fenestra below obturator foramen [inapplicable for taxa without a ventrally extensive lateromedially narrow flange]: (0) absent; (1) present (modified from Rauhut & Pol 2021: C627).
801. Pubis, pubic fenestra, configuration: (0) completely enclosed; (1) open ventrally; (2) open posteriorly and confluent dorsally with the obturator foramen; (3) posteriorly open and offset from the obturator foramen (new).
802. **Pubis, origin of M.ambiens: 0 absent, rugosity, or depression; 1 present as a tubercle raised from proximolateral pubic shaft (Wang et al., 2017: C610**)
803. Flange-like morphology of pubic tubercle: 0 absent; 1 present (Wang et al., 2017: C608)
804. Pubis, shaft, shape in lateral view: (0) anteriorly convex; (1) straight; (2) anteriorly slightly concave and posteriorly ± straight; (3) notably anteriorly concave and posteriorly convex ORDERED (modified from Rauhut & Pol 2021: C627)
805. Lateral face of pubic shafts: 0 smooth; 1 with prominent lateral tubercle about halfway down the (Wang et al., 2017: C626)
806. Pubic apron: 0 present; 1 absent (Wang et al., 2017: C617)
807. Form of pubic apron: 0 extends medially from middle of cylindrical pubic shaft; 1 shelf extends medially from anterior edge of anteroposteriorly flattened shaft (Wang et al., 2017: C618)
808. Pubic apron, length: 0 about half of pubic shaft length or more; 1 less than 1/3 of shaft length (Wang et al., 2017: C619)
809. Pubis, pubic apron, ventral extent: (0) completely closed, or with only a minor medial notch with converging edges; (1) with a marked ventral notch with parallel edges (as in Coelophysis); (2) deep notch that forms a median opening due to distal contact between pubic boots ORDERED (modified from Rauhut & Pol 2021: C622)
810. **Pubis, expanded pubic boot: (0) absent; (1) present (modified from Rauhut & Pol 2021: C614)**
811. Pubic boot projects: 0 anteriorly and posteriorly; 1 posteriorly, but little or no anterior process; 2 only expanded anteriorly (Wang et al., 2017: C613)
812. **Ratio length of pubic boot to length of pubic shaft: 0 less than 0.3; 1 more than 0.3 (Wang et al., 2017: C614)**
813. Pubic boot outline, distal view: 0 triangular; 1 narrow, with subparallel margins (Wang et al., 2017: C615)
814. Lateral fossa on pubic boot: 0 absent; 1 present (Wang et al., 2017: C616)
815. Pubis, pubic boot, large medial opening distally within pubic boot, forming a posterodorsally opening pubic cup: (0) absent; (1) present (modified from Rauhut & Pol 2021: C622)

### Ischium

816. Length of ischium: 0 more than two-thirds pubis length; 1 two thirds or less of pubic length (Wang et al., 2017: C628)
817. Ischium, posterior deflection distal to the obturator process (measured as the angle between the main axis of the ischium proximal to the obturator process towards the iliac peduncle and the main axis of the ischial shaft distal to the obturator process): (0) >120°; (1) 120° - 150°; (2) 150° - 175° (3) >175°ORDERED
818. Ischium, proximal end, anterior view: (0) straight; (1) laterally convex; (2) laterally concave (modified from Rauhut and Pol 2021: 637)
819. Ischium, proximoposterior part, groove on lateral side that extends from proximal end of ischial shaft to almost iliac articulation: (0) absent; (1) present (modified from Zanno & Makovicky 2013)
820. Ischium, proximoposterior part, posterior process: (0) absent; (1) developed as a small, rounded flange; (2) developed as a pronounced posterodorsal process; (3) developed as a large posterodorsal process, offset from iliac peduncle by a notch ORDERED (modified from Rauhut & Pol 2021: C644, 645)
821. Ischium, proximoposterior part, semicircular scar: (0) absent; (1) developed as a small posterior bulge; 2 developed as a large triangular flange ORDERED (modified from Rauhut & Pol 2021: C646)
822. Ischium, pubic peduncle, relative length excluding obturator flange (see image): (0) as long as or shorter than high; (1) considerably longer than high (new)
823. Obturator process of ischium: 0 absent; 1 present (Wang et al., 2017: C629)
824. Obturator process on ischium: 0 confluent with pubic peduncle; 1 offset from pubic peduncle by a distinct notch (Wang et al., 2017: C637)
825. Morphology of offset triangular obturator process of ischium: 0 proximodistally wide base	along ischiac shaft, rostral process short; 1 proximodistally narrow base, rostral process long (Wang et al., 2017: C638)
826. Position of obturator process: 0 proximal in position; 1 located near middle of ischiadic shaft; 2 located at distal end of ischium ORDERED (Wang et al., 2017: C630)
827. Ventral notch at distal edge of ischial obturator process: 0 absent, grades smoothly into ischial shafts; 1 present (Wang et al., 2017: C636)
828. Ischium, subsidiary process below the obturator process: (0) absent; (1) present (Coria & Currie 2006)
829. Ischium, contact with pubis distal to pubic peduncle: (0) absent; (1) present (modified from Rauhut & Pol 2021: C638)
830. Ischial shaft: 0 Rodlike; 1 anteroposteriorly wide and plate like (Wang et al., 2017: C631)
831. Ischium, lateral view, shaft distal to the obturator process: (0) straight; (1) at least slightly flexed anteriorly over most of its length; (2) flexed anteriorly only towards the distal end (modified from Rauhut & Pol 2021: C636)
832. Lateral blade of ischium: 0 flat or laterally convex; 1 laterally concave; 2 with longitudinal ridge subdividing lateral surface into anterior (including obturator process) and posterior parts (Wang et al., 2017: C632)
833. Ischium, morphology of symphysis: 0 unexpanded; 1 expanded as apron (Wang et al., 2017: C627)
834. Tubercle on anterior edge of ischium: 0 absent; 1 present (Wang et al., 2017: C642)
835. Ischium, conjoined shafts, posterior midline crest: (0) absent; (1) present (modified from Rauhut & Pol 2021: 645)
836. **Distal end of ischium: 0 strongly expanded, forming ischial "boot" (2x or more the width of ischial shaft); 1 slightly expanded; 2 tapering or unexpanded ORDERED (Wang et al., 2017: C639)**
837. Distal ends of ischia: 0 form symphysis; 1 approach one another but do not form symphysis; 2 widely separated ORDERED (Wang et al., 2017: C640)

## Hindlimbs

838. **Femur, maximum length in relation to maximal length of tibia in subadult to adult individuals: (0) greater than or equal; (1) shorter than tibia (modified from Rauhut & Pol 2021: C649)**

### Femur

839. Femur, femoral head and greater trochanter in anterior or posterior view: (0) confluent with greater trochanter; (1) separated by a distinct cleft (Rauhut & Pol 2021: C653)
840. **Femur, femoral head, orientation in the dorsoventral plane: (0) ventromedial; (1) horizontal; (2) dorsomedial ORDERED (Rauhut & Pol 2021: C654)**
841. Femur, femoral head, orientation in the horizontal plane: (0) anteromedially; (1) strictly medially (Rauhut & Pol 2021: C653)
842. Femur, femoral head, shape in proximal view: (0) medially rounded; (1) medially pointed (new)
843. Femur, femoral head, anteromedial tuber: (0) inconspicuous, rounded; (1) developed as an anteromedially pointed projection (new)
844. Femur, groove on proximal surface of head oriented oblique to long axis of head (‘articular groove’): (0) present; (1) absent (Benson 2010; Carrano et al. 2012)
845. Femur, femoral head, fovea ligamentum capitis on the proximomedial side: (0) inconspicuous; (1) developed as a posteroproximal incision, confluent with the oblique ligament groove; (2) developed as a proxeromedial depression (modified from Rauhut & Pol 2021: C650; see Egawa et al. 2022)
846. **Femur, femoral head, oblique ligament groove on the posterior surface: (0) absent or very shallow; (1) deep, bound medially by a well-developed posterior lip (Rauhut & Pol 2021: 651)**
847. Femur, femoral head, mediodistal margin: (0) curves smoothly into shaft; (1) separated from shaft by marked incision, formed by a ventral lip of the articular surface (new)
848. Femur, proximal end, greater trochanter: (0) anteroposteriorly narrow and narrowing from medial to lateral; (1) anteroposteriorly expanded, forming a trochanteric crest (Rauhut & Pol 2021: C655)
849. **Femur, proximal end, attachment area for the M. iliofemoralis externus on the posterolateral side: (0) indistinct; (1) developed as a posterolateral bulge (=posterior trochanter; posterolateral trochanter); (2) developed as a trochanteric shelf in at least some individuals (modified from Rauhut & Pol 2021: C656)**
850. **Femur, proximal end, dorsolateral trochanter: (0) indistinct; (1) ridge-like (modified from Griffin 2018)**
851. Femur, lesser trochanter (=anterior trochanter, M. *iliofemoralis cranialis* insertion), cleft separating proximal end of lesser trochanter from femoral shaft or greater trochanter: (0) absent; (1) present (modified from Rauhut & Pol 2021: C658)
852. Femur, lesser trochanter, position of cleft separating proximal end of lesser trochanter from femoral shaft or greater trochanter: (0) distal to femoral head; (1) at level of femoral head, but below the mid-height of the former; (2) more proximally, at proximal half of femoral head ORDERED (modified from Rauhut & Pol 2021: C657)
853. Femur, lesser trochanter, shape [inapplicable for taxa in which the lesser trochanter is fused to the greater trochanter]: (0) short and ridge-like; (1) tab-like, squared (state 1 in Rauhut 2003: C198, Fig. 42A; “spike-like” in Carrano & Sampson 2008: C132); (2) alariform; (3) Cylindrical in cross-section (sometimes “finger-like”, state 1 of Norell et al. 2001: C183) (modified from Rauhut & Pol 2021: C658; Wang et al. 2071: C656; Choiniere et al. 2014: C487; Langer & Benton 2006: C82; Rauhut 2003: C198; Norell et al. 2001: C183; Gauthier 1986: C49)
854. Femur, lesser trochanter, placement of proximal end: (0) at distal end of femoral head; (1) more proximally placed, but distal to greater trochanter; (2) as proximal or more proximal than greater trochanter ORDERED (Rauhut & Pol 2021: C659)
855. **Femur, lesser trochanter, anterior accessory trochanteric crest on distal end: (0) absent; (1) present as a low anterior flange, confluent with the anterior border of the lesser trochanter; (2) strongly projecting process that is clearly offset from the anterior margin of the lesser trochanter, at least proximally ORDERED (modified from Rauhut & Pol 2021: C660)**
856. **Femur, fourth trochanter, development: (0) well-developed as a large, semioval crest; (1) reduced to a low, elongate ridge; (2) absent ORDERED (modified from Rauhut & Pol 2021: C662)**
857. Femur, fourth trochanter, shape in lateral or medial view: (0) proximodistally symmetrical; (1) asymmetrical, distal end forms higher angle with diaphysis (Rauhut & Pol 2021: 663)
858. Femur, fourth trochanter, placement of distal end: (0) at about the mid-length of the femur; (1) considerably more proximally, closer to the proximal third (new)
859. Femur, fourth trochanter, groove on the medial side: (0) absent; (1) developed as shallow depression adjacent to the trochanter; (2) developed as a narrow groove on the medial side of the trochanter (new)
860. **Femur, adductor ridge on posterior side of distal half: (0) absent or inconspicuous; (1) developed as a low, rounded crest in its proximal part (new)**
861. **Femur, distal condyles, distal extent of lateral condyle if compared to medial condyle: (0) approximately the same level as the medial condyle; (1) distinctly farther than medial condyle and distal surface of medial condyle is flattened (Rauhut & Pol 2021: C674; Wang et al. 2017: C671; Choinere et al. 2014: C498; Benson 2010: C196; Brusatte & Sereno 2008: C84 )**
862. Femur, distal end, marked depression on the medial side of the anterior surface distally: (0) absent; (1) present (modified from Rauhut & Pol 2021: C665)
863. Femur, medial epicondyle on the anteromedial side of the distal shaft ORDERED: (0) absent, anterior side of femur curves gently into medial side; (1) medial surface offset from the anterior surface by marked edge; (2) developed as a medial flange that overhangs the medial side (modified from Rauhut & Pol 2021: C667)
864. Femur, flange formed by medial epicondyle on anteromedial side of distal shaft, extent [inapplicable for taxa that do not have a flange]: (0) extends to distal end of femur, medial side straight; (1) ends proximal to distal end, medial side proximodistally convex (new)
865. Femur, flange formed by medial epicondyle on anteromedial side of distal shaft, development [not applicable to taxa that do not have a flange]: (0) flange proximally that gradually widens in medial view distally to form a triangular medial platform towards its distal end; (1) remains flange-like throughout its length (new)
866. Femur, flange formed by medial epicondyle on anteromedial side of distal shaft, orientation [not applicable to taxa that do not have a flange]: (0) strictly medially; (1) anteromedially (new)
867. Femur, medial distal condyle, orientation in distal view: (0) anteroposteriorly; (1) anterolateral to posteromedial (modified from Rauhut & Pol 2021: C666)
868. Femur, distal end, anteriorly projecting tubercle at the anterolateral side of the medial condyle: (0) absent; (1) present (new)
869. **Femur, distal end, extensor groove broadly incising into the anterior surface in distal view: (0) absent or poorly developed; (1) well developed (modified from Rauhut & Pol 2021: C664)**
870. Femur, distal articular surface, morphology in distal view: (0) central depression; (1) continuous anteroposterior groove separating the lateral and medial condyles (Carrano et al. 2012: C316)
871. Femur, distal end, prominent tubercle within the flexor groove: (0) absent; (1) present (new)
872. Femur, popliteal fossa: (0) open distally; (1) closed by contact between distal condyles (Rauhut & Pol 2021: C668)
873. Femur, distal end, transverse ridge connecting distal condyles in popliteal fossa: (0) absent; (1) present (Rauhut & Pol 2021: C669)
874. Femur, morphology tibiofibularis crest in posterior view: (0) broad, only slightly longer proximodistally than wide transversely; (1) narrow, at least twice as long proximodistally as wide transversely (modified from Rauhut & Pol 2021: C670)
875. Femur, orientation of tibiofibularis crest in posterior view: (0) longitudinal, proximodistally; (1) oblique (modified from Rauhut & Pol 2021: C670)

### Tibia

876. Anteroposterior length of proximal end of tibia in proximal view: 0 exceeds mediolateral width; 1 less than mediolateral width (Wang et al., 2017: C672)
877. Tibia, lateral condyle, anterior offset (measured as the angle between two lines connecting the anteriormost point of the cnemial crest to the posteriormost point of the medial condyle and the latter to the posteriormost point of the lateral condyle, respectively): (0) condyles point equally posteriorly, angle > 75°; (1) moderate offset, angle 50- 75°; (2) condyle strongly offset, angle < 50° ORDERED (new)
878. **Cnemial crest proximal projection: 0 approximately at the same level as posterior condyles; 1 projects strongly proximal to posterior condyles (Wang et al., 2017: C673)**
879. **Anteroposterior length of cnemial crest: 0 prominent but not expanded; 1 strongly anteroposteriorly expanded; 2 anteroposterior expansion proximodistally expanded at its distal end, sometimes expanding ventrally. (Wang et al., 2017: C674)**
880. Accessory ridge on lateral surface cnemial crest: 0 absent; 1 (Wang et al., 2017: C675)
881. Medial cnemial crest: 0 absent; 1 (Wang et al., 2017: C676)
882. Fibular condyle on proximal end of tibia: 0 confluent with cnemial crest anteriorly in proximal view; 1 strongly offset from cnemial crest (Wang et al., 2017: C677)
883. Tibia, proximal articulation, medial condyle, caudal margin in proximal view: (0) broadly rounded or subrectangular; (1) arcuate and posteriorly angular (modified from Rauhut et al. 2021: C679).
884. Tibia, proximal articulation, posterior cleft between lateral and medial condyle: (0) absent; (1) present (Rauhut et al. 2021: C680)
885. Tibia, proximal articulation, lateral condyle, lateromedial width relative to medial condyle [inapplicable for taxa with poorly defined posterior condyles]: (0) significantly smaller; (1) subequal; (2) significantly larger (new).
886. Fibular crest (ridge on lateral side of tibia for connection with fibula): 0 absent; 1 present (Wang et al., 2017: C680)
887. Form of fibular crest ORDERED: 0 extending from proximal articular surface distally; 1 incomplete ossification, lateral process from the fibular condyle proximally separated from the rest of the crest placed more distally; 2 completely separated from proximal articular surface (Wang et al., 2017: C681)
888. Fibular crest length: 0 short, less than one fifth tibial length; 1 long, between one quarter and one third tibial length (Wang et al., 2017: C686)
889. Shape of fibular crest: 0 quadrangular; 1 low and rounded (Wang et al., 2017: C684)
890. Tibia, morphology of fibular crest: 0 narrow; 1 bulbous (Wang et al., 2017: C683)
891. Dorsal portion of fibular crest of the tibia: 0 subvertical; 1 bifurcate fibular crest; 2 proximal end curves strongly anteriorly onto the lateral surface of the cnemial crest (Wang et al., 2017: C682)
892. Fibular crest distal extension: 0 proximally positioned; 1 extends to midshaft of tibia (Wang et al., 2017: C685)
893. Bracing for fibula on lateral midshaft of the tibia below the fibular crest: 0 absent; 1 present (Wang et al., 2017: C687)
894. Lateral malleolus of tibia in anterior view: 0 distal extension level with medial malleolus; 1 lateral malleolus projects far distal to medial malleolus (Wang et al., 2017: C688)
895. Bracing for ascending process of astragalus on anterior side of distal tibia: 0 distinct 'step' running obliquely from mediodistal to lateroproximal; 1 anterior side of tibia flat where it articulates with the ascending process; 2 central longitudinal ridge bounded medially and laterally by fossae (Wang et al., 2017: C689)
896. **Diagonal tuberosity on the anterior surface of the distal end of tibia: 0 absent; 1 present (Wang et al., 2017: C690)**
897. **Tibia, distal end, rugose patch on the posterior side: (0) absent; (1) present (modified Rauhut & Pol 2021: C648)**
898. Tibia, distal articular surface, outline: (0) subquadrangular, only slightly wider transversely than anteroposteriorly; (1) subquadrangular with a small lateral process; (2) triangular, strongly expanded mediolaterally; (3) subrectangular, more than three times wider than deep (Rauhut, 2003)
899. Tibia, distal articular surface, groove extending from the posteromedial side anterolaterally, for the reception of a peg on the posteromedial side of the astragalus (can also be coded based on the presence of the peg on the astragalus): (0) absent; (1) present (new)

### Fibula

900. Fibula: 0 reaches proximal tarsals; 1 short, tapering distally, and not in contact with proximal tarsals (Wang et al., 2017: C691)
901. Proximal fibular margin: 0 caudal portion extends proximally beyond level of anterior portion with a distinct posterodorsal projection; 1 subhorizontal or shallowly inclined in either direction; 2 cranial portion extends proximally beyond level of posterior ORDERED (Wang et al., 2017: C693)
902. Fibula, proximal end, anteroposteriorly expansion in lateral view: 0 more posteriorly than anteriorly expanded; 1 anterior and posterior expansion roughly equal in size; 2 anterior expansion more pronounced than posterior expansion (new)
903. Fibular proximal dimensions (of mediolaterally compressed fibula) in proximal view: 0 anterior portion subequal to posterior portion in mediolateral width; 1 anterior portion mediolaterally wider than posterior portion (Wang et al., 2017: C694)
904. **Lateral surface of proximal fibula: 0 shallow longitudinal trough situated posteriorly; 1 trough absent or weak groove present, surface convex (Wang et al., 2017: C692)**
905. Ridge on medial side of proximal end of fibula, that runs anterodistally from the posteroproximal end: 0 weak or absent; 1 robust ridge; 2 ridge strongly developed, creates an additional well defined and deep fossa dorsal to the fibular fossa ORDERED (Wang et al., 2017: C698)
906. Medial surface of proximal end of fibula: 0 concave along long axis; 1 flat (Wang et al., 2017: C699)
907. Deep oval fossa on medial surface of fibula near proximal end: 0 absent; 1 present (Wang et al., 2017: C700)
908. Extent of fibular fossa: 0 present on medial and posterior aspects of diaphysis; 1 only medially (Wang et al., 2017: C701)
909. **Insertion of m.iliofibularis on fibular shaft: 0 not especially marked or rugose area only; 1 present as a well-developed anterolateral tubercle or flange (Wang et al., 2017: C695)**
910. Form of m.iliofibularis insertion: 0 tubercle; 1 anterolaterally curving flange (Wang et al., 2017: C696)
911. Position of insertion of m.iliofibularis on fibular shaft: 0 proximal; 1 midshaft (Wang et al., 2017: C697)

### Astragalus & Calcaneum

912. Astragalus and calcaneum: 0 condyles indistinct or poorly separated; 1 distinct condyles separated by prominent vertical tendinal groove on anterior surface (Wang et al., 2017: C702)
913. **Astragalus and calcaneum: 0 not fused to each other or to the tibia; 1 fused to each other and sometimes to the tibia in late ontogeny (Wang et al., 2017: C703)**
914. Fibular facet on astragalus: 0 large and facing partially proximally; 1 reduced and facing laterally or absent (Wang et al., 2017: C704)
915. Ascending process of the astragalus: 0 absent; 1 lower than the posterior dorsoventral height of the astragalar body; 2 higher than the posterior dorsoventral height of the astragalar body; 3 more than twice the posterior dorsoventral height of the astragalar body ORDERED (Wang et al., 2017: C705)
916. Shape of ascending process of the astragalus: 0 broad, covering most of anterior surface of distal end of tibia; 1 narrow, covering only lateral half of anterior surface of tibia; 2 narrow, base of ascending process inset laterally and medially from the edges of the (Wang et al., 2017: C706)
917. Notch on medial edge of ascending process of the astragalus: 0 absent; 1 present (Wang et al., 2017: C707)
918. Fossa on anterior surface of mesial base of ascending process of astragalus, sometimes bearing accessory fenestrations: 0 absent; 1 present (Wang et al., 2017: C708)
919. Ascending process of astragalus and astragalar body: 0 confluent or only slightly offset from astragalar body; 1 offset from astragalar body by a pronounced groove (Wang et al., 2017: C709)
920. Horizontal groove across anterior surface of astragalar condyles: 0 absent; 1 (Wang et al., 2017: C711)
921. Shallow fossa on the medial surface of the astragalar body: 0 absent; 1 present (Wang et al., 2017: C712)
922. Astragalar condyles: 0 almost entirely below tibia and face distally; 1 significantly expanded proximally on anterior side of tibia and face anterodistally (Wang et al., 2017: C710)
923. Calcaneum: 0 without facet for tibia; 1 well-developed facet for tibia present (Wang et al., 2017: C713)

## Pes

924. Completely reversed hallux (arch of ungual phalanx of digit I opposing the arch of the unguals of digits II-IV): 0 absent; 1 present (Wang et al., 2017: C736)
925. Pedal digit IV: 0 significantly shorter than III and subequal in length to II, foot is symmetrical; 1 significantly longer than II and only slightly shorter than or subequal to III, foot is asymmetrical (Wang et al., 2017: C737)

### Tarsals

926. **Distal tarsals: 0 separate, not fused to metatarsals or fused late in ontogeny; 1 form metatarsal cap with intercondylar prominence that fuses to metatarsal early in postnatal ontogeny (Wang et al., 2017: C714)**

### Metatarsals

927. **Metatarsals: 0 not co-ossified; 1 co-ossifed (tarsometatarsus) (Wang et al., 2017: C715)**
928. **Maximum length of metatarsals: 0 greater than 50% tibia length; 1 49-26% tibia length (Wang et al., 2017: C716)**
929. Metatarsal I: 0 present; 1 absent (Wang et al., 2017: C717)
930. Metatarsal I ORDERED: 0 proximal end of MTI similar to that of MT II-IV and reaches ankle joint; 1 proximal end of MTI narrower than II-IV and reaches ankle joint; 2 proximal end of MTI narrower than II-IV and fails to reach ankle joint (Wang et al., 2017: C718)
931. Metatarsal I position: 0 articulates in the middle of the medial surface of Mt II; 1 articulates with the distal quarter of Mt II (Wang et al., 2017: C719)
932. Diaphysis of Metatarsal II: 0 Of consistent mediolateral width and subequal or slightly narrower than MT III; 1 Of consistent mediolateral with and much narrower than MT III; 2 Diaphysis attenuates proximally relative to distal condyle and is much thinner than MT III ORDERED (Wang et al., 2017: C724)
933. Posteromedial margin metatarsal II diaphysis: 0 well-developed axial ridge absent or area rugose; 1 with axial ridge projecting caudally or medially (Wang et al., 2017: C723)
934. Metatarsal II proximal end of flexor surface: 0 flat or small tab present; 1 large quadrangular flange present (Wang et al., 2017: C720)
935. **Tuber along extensor surface of MtII: 0 absent; 1 present (Wang et al., 2017: C722)**
936. Distal end of metatarsal II: 0 smooth, not ginglymoid; 1 with developed ginglymus (Wang et al., 2017: C721)
937. Metatarsal III: 0 subequal in width to Mt II and IV proximally; 1 pinched between II and IV and not visible in anterior view proximally; 2 does not reach the proximal end of the ORDERED (Wang et al., 2017: C726)
938. Metatarsal III shape of proximal end: 0 rectangular, medial and lateral surfaces pinched; 1 hourglass-shaped, medial and or lateral surface(s) concave; 2 T-Shaped, posterior end like the top of the letter T, anterior end not expanded (Wang et al., 2017: C727)
939. Metatarsal III shape of shaft in cross section: 0 rectangular; 1 wedge-shaped, plantar surface pinched (Wang et al., 2017: C728)
940. Distal end of metatarsal III: 0 smooth, not ginglymoid; 1 with developed ginglymus (Wang et al., 2017: C725)
941. Length of MtIV: 0 subequal to Mt II; 1 markedly longer than Mt II (Wang et al., 2017: C730)
942. Shaft of MT IV: 0 round or thicker dorsoventrally than wide in cross section; 1 shaft of Mt IV mediolaterally widened and flat in cross section (Wang et al., 2017: C729)
943. **Posterolateral margin of MtIV diaphysis: 0 well-developed flange absent or area rugose; 1 with flange projecting caudally or laterally (Wang et al., 2017: C731)**
944. Metatarsal IV, width of distal condyle: 0 subequal; 1 or less than 50% width of distal condyle of metatarsal II (Wang et al., 2017: C732)
945. Distal end of metatarsal IV diverges strongly laterally at its distal end: 0 absent; 1 (Wang et al., 2017: C733)
946. Metatarsal V: 0 present; 1 absent (Wang et al., 2017: C735)
947. Metatarsal V: 0 with convex distal articular facet; 1 strongly reduced and lacking distal articular facet; 2 short, without articular surface, transversely flattened and bowed anteriorly distally ORDERED (Wang et al., 2017: C734)

### Phalanges

948. Pedal digit II, phalanx II-1, relative length in comparison to III-1: (0) significantly shorter; (1) approximately as long; (2) markedly longer (new)
949. Pedal digit II, phalanx II-1, relative length in comparison to IV-1: (0) longer; (1) shorter (new)
950. Pedal digit II, relative length of phalanx II-2 (excluding ventral heel): (0) about half or less the length of II-1; (1) considerably more than half the length of II-1 (2) around the same length of II-1 or longer ORDERED (new)
951. Pedal digit II, phalanx II-2, pronounced proximoventral heel: (0) absent, (1) present (modified from Rauhut & Pol, 2021: C741)
952. Pedal digit III, phalanx III-2, relative length: (0) with neck that is longer than distal gynglimus; (1) with neck that is as long as or shorter than distal gynglimus (new)
953. Pedal digit III, phalanx III-3, relative length: (0) with neck that is longer than distal gynglimus; (1) with neck that is as long as or shorter than distal gynglimus; (2) extremely shortened, neck absent ORDERED (new)
954. Pedal digit IV, phalanx IV-1, relative length: (0) with neck that is longer than distal gynglimus; (1) with neck that is as long as or shorter than distal gynglimus; (2) extremely shortened, neck absent (modified from Rauhut & Pol 2021: 742)
955. Pedal digit IV, phalanx IV-2, relative length: (0) with neck that is longer than distal gynglimus; (1) with neck that is as long as or shorter than distal gynglimus; (2) extremely shortened, neck absent ORDERED (modified from Rauhut & Pol 2021: 742)
956. Pedal digit IV, phalanx IV-3, relative length: (0) with neck that is longer than distal gynglimus; (1) with neck that is as long as or shorter than distal gynglimus; (2) extremely shortened, neck absent ORDERED (modified from Rauhut & Pol 2021: 742)
957. Pedal digit IV, phalanx IV-4, relative length: (0) with neck that is longer than distal gynglimus; (1) with neck that is as long as or shorter than distal gynglimus; (2) extremely shortened, neck absent ORDERED (modified from Rauhut & Pol 2021: 742)
958. Pedal unguals, morphology of lateral and medial grooves: (0) single; (1) fork proximally (modified from Rauhut & Pol, 2021: C744)
959. Pedal unguals, attachment of flexor tendon, development: (0) small protuberance or flat; (1) well-developed flexor tubercle; (2) marked proximal depression (modified from Rauhut & Pol 2021: C746)
960. Pedal unguals, digit I, relative size (measured from the dorsal end of the proximal articular facet in a straight line to the tip of the claw) compared to the smallest remaining pedal ungual: (0) significantly smaller, half the size or less; (1) smaller; (2) approximately as long or longer ORDERED (modified from Rauhut & Pol 2021: C747)
961. Pedal unguals, digit II, relative size (measured from the dorsal end of the proximal articular facet in a straight line to the tip of the claw): (0) shorter than or approximately as long as pedal ungual III; (1) longer than pedal ungual III; (2) more than 30% longer than pedal ungual III ORDERED (modified from Rauhut & Pol, 2021: C741)
962. Pedal unguals, digit II, claw grooves mediolateral symmetry: (0) symmetrical; (1) asymmetrical, on different levels medially and laterally (modified from Rauhut & Pol 2021: C745)
963. Pedal unguals, digit II, dorsal view: (0) straight; (1) slightly curved medially (new) EC
964. Pedal unguals, digit II, ventral side: (0) rounded, or flattened; (1) sharp-edged (modified from Rauhut & Pol, 2021: C741)
965. Pedal unguals II-IV, pronounced ventrolateral and ventromedial edges: (0) absent; (1) present (new)

# Integument

966. Integument, feather morphology: filamentous feathers only (0); contour feathers (1) (Rauhut & Pol 2021: C1)
967. Integument, vaned feathers on forelimb: (0) symmetric; (1) asymmetric (Rauhut & Pol 2021: C2)

2 – Complete topology recovered form MTWG data matrix using equal weight characters

3 – Complete topology recovered form MTWG data matrix using implied weight characters k =16

4 – List of synapomorphies

Synapomorphies for Consensus equal weight

Tree 10000 :

Silesaurus_opolensis :

No autapomorphies

Eoraptor_lunensis :

Char. 1: 1 --> 0

Char. 10: 0 --> 1

Char. 21: 0 --> 1

Char. 45: 1 --> 0

Char. 54: 0 --> 1

Char. 103: 0 --> 1

Char. 128: 1 --> 0

Char. 135: 1 --> 0

Char. 174: 0 --> 1

Char. 193: 0 --> 1

Char. 250: 0 --> 1

Char. 328: 0 --> 1

Char. 348: 0 --> 1

Char. 349: 1 --> 0

Char. 387: 0 --> 1

Char. 391: 1 --> 0

Char. 432: 1 --> 0

Char. 513: 2 --> 1

Char. 642: 1 --> 0

Char. 650: 1 --> 0

Char. 713: 1 --> 0

Char. 746: 0 --> 1

Char. 877: 0 --> 1

Char. 917: 0 --> 1

Bagualosaurus_agudoensis :

Char. 858: 1 --> 0

Buriolestes_schultzi :

Char. 36: 0 --> 1

Char. 43: 0 --> 1

Char. 52: 1 --> 0

Char. 112: 0 --> 1

Char. 116: 0 --> 1

Char. 179: 1 --> 0

Char. 398: 1 --> 0

Char. 406: 1 --> 0

Char. 412: 0 --> 1

Char. 414: 1 --> 0

Char. 416: 2 --> 0

Char. 417: 2 --> 01

Char. 598: 0 --> 1

Char. 599: 0 --> 1

Char. 668: 0 --> 1

Char. 673: 0 --> 1

Char. 852: 0 --> 1

Char. 900: 1 --> 2

Char. 905: 0 --> 1

Char. 908: 0 --> 1

Plateosaurus_trossingensis :

Char. 549: 2 --> 1

Char. 554: 0 --> 1

Char. 580: 0 --> 1

Char. 644: 2 --> 1

Char. 671: 0 --> 1

Char. 705: 0 --> 1

Char. 732: 1 --> 0

Char. 790: 0 --> 1

Char. 791: 0 --> 1

Char. 792: 1 --> 0

Char. 858: 1 --> 0

Char. 946: 1 --> 0

Char. 951: 0 --> 1

Pampadromaeus_barberenai :

Char. 91: 0 --> 1

Char. 95: 2 --> 1

Char. 127: 1 --> 0

Char. 305: 0 --> 1

Char. 367: 0 --> 1

Char. 436: 1 --> 0

Char. 549: 2 --> 1

Char. 606: 0 --> 1

Char. 635: 0 --> 1

Char. 842: 1 --> 0

Saturnalia_tupiniquim :

Char. 155: 1 --> 0

Char. 276: 1 --> 0

Char. 288: 0 --> 1

Char. 404: 0 --> 1

Char. 438: 0 --> 2

Char. 441: 0 --> 2

Char. 649: 0 --> 1

Char. 673: 0 --> 1

Char. 772: 0 --> 2

Char. 839: 1 --> 0

Char. 852: 0 --> 1

Guaibasaurus_candelariensis :

Char. 584: 2 --> 0

Char. 603: 0 --> 1

Char. 606: 0 --> 1

Char. 718: 0 --> 1

Char. 816: 1 --> 0

Char. 846: 1 --> 0

Char. 917: 0 --> 1

Eodromaeus_murphi :

Char. 38: 0 --> 1

Char. 45: 1 --> 0

Char. 47: 1 --> 0

Char. 72: 0 --> 1

Char. 76: 0 --> 1

Char. 114: 0 --> 1

Char. 228: 0 --> 1

Char. 288: 0 --> 1

Char. 391: 1 --> 2

Char. 409: 2 --> 1

Char. 432: 1 --> 0

Char. 475: 0 --> 2

Char. 649: 0 --> 1

Char. 664: 0 --> 1

Char. 692: 1 --> 0

Char. 803: 1 --> 0

Char. 862: 0 --> 1

Char. 909: 1 --> 0

Abelisaurus_comahuensis :

Char. 19: 0 --> 1

Char. 288: 0 --> 1

Acrocanthosaurus_atokensis :

Char. 22: 1 --> 0

Char. 49: 0 --> 1

Char. 62: 1 --> 0

Char. 65: 0 --> 1

Char. 185: 0 --> 1

Char. 194: 1 --> 0

Char. 452: 1 --> 0

Char. 487: 0 --> 2

Char. 543: 0 --> 1

Char. 608: 0 --> 1

Char. 618: 1 --> 0

Char. 801: 0 --> 1

Char. 803: 1 --> 3

Char. 839: 2 --> 1

Char. 841: 0 --> 1

Char. 879: 1 --> 0

Char. 884: 1 --> 2

Char. 955: 1 --> 2

Aerosteon_riocoloradensis :

Char. 155: 1 --> 0

Char. 157: 0 --> 1

Char. 540: 0 --> 1

Char. 549: 1 --> 0

Char. 558: 0 --> 1

Char. 566: 0 --> 1

Char. 631: 0 --> 1

Char. 637: 1 --> 0

Char. 782: 1 --> 0

Char. 803: 1 --> 2

Afromimus_tenerensis :

Char. 894: 0 --> 2

Char. 896: 0 --> 1

Char. 912: 1 --> 0

Char. 918: 0 --> 1

Afrovenator_abakensis :

Char. 40: 0 --> 1

Char. 54: 0 --> 1

Char. 59: 0 --> 1

Char. 93: 1 --> 0

Char. 94: 0 --> 1

Char. 96: 1 --> 2

Char. 110: 0 --> 1

Char. 113: 1 --> 0

Char. 115: 0 --> 1

Char. 153: 1 --> 2

Char. 457: 1 --> 0

Char. 467: 1 --> 0

Char. 532: 1 --> 2

Char. 711: 0 --> 1

Char. 803: 1 --> 2

Char. 809: 1 --> 0

Char. 846: 1 --> 0

Albertosaurus_sarcophagus :

Char. 127: 2 --> 3

Char. 191: 0 --> 1

Char. 271: 0 --> 1

Char. 368: 1 --> 0

Alioramus :

Char. 0: 1 --> 2

Char. 3: 1 --> 0

Char. 7: 1 --> 0

Char. 59: 0 --> 1

Char. 60: 0 --> 1

Char. 105: 0 --> 1

Char. 127: 2 --> 1

Char. 227: 0 --> 1

Char. 253: 1 --> 0

Char. 277: 0 --> 1

Char. 293: 0 --> 1

Char. 350: 1 --> 0

Char. 381: 1 --> 0

Char. 382: 1 --> 0

Char. 386: 0 --> 1

Char. 409: 1 --> 2

Char. 451: 2 --> 0

Char. 455: 1 --> 0

Char. 470: 1 --> 0

Char. 483: 1 --> 0

Char. 582: 0 --> 1

Char. 773: 0 --> 1

Char. 784: 0 --> 1

Char. 790: 0 --> 1

Alpkarakush_kyrgyzicus :

Char. 135: 2 --> 1

Char. 139: 0 --> 2

Char. 152: 0 --> 1

Char. 157: 0 --> 1

Char. 173: 1 --> 0

Char. 176: 1 --> 0

Char. 417: 1 --> 0

Char. 550: 0 --> 1

Char. 569: 0 --> 1

Char. 723: 0 --> 1

Char. 766: 0 --> 1

Char. 788: 0 --> 1

Char. 793: 1 --> 0

Char. 803: 1 --> 3

Char. 818: 1 --> 0

Char. 839: 1 --> 2

Char. 853: 1 --> 0

Char. 858: 1 --> 0

Char. 861: 1 --> 0

Char. 862: 1 --> 2

Char. 886: 2 --> 1

Char. 888: 0 --> 1

Char. 909: 0 --> 1

Char. 911: 0 --> 1

Char. 912: 0 --> 1

Char. 918: 0 --> 1

Allosaurus :

Char. 19: 0 --> 1

Char. 21: 1 --> 0

Char. 80: 0 --> 1

Char. 90: 0 --> 1

Char. 91: 1 --> 2

Char. 96: 2 --> 3

Char. 113: 1 --> 0

Char. 135: 2 --> 1

Char. 146: 0 --> 1

Char. 154: 0 --> 1

Char. 173: 1 --> 0

Char. 214: 1 --> 0

Char. 229: 1 --> 0

Char. 238: 0 --> 2

Char. 251: 0 --> 1

Char. 254: 1 --> 0

Char. 261: 0 --> 1

Char. 278: 0 --> 1

Char. 316: 1 --> 2

Char. 327: 0 --> 1

Char. 335: 1 --> 0

Char. 350: 0 --> 1

Char. 351: 0 --> 1

Char. 395: 1 --> 2

Char. 402: 0 --> 2

Char. 420: 1 --> 2

Char. 505: 1 --> 0

Char. 516: 1 --> 0

Char. 540: 0 --> 1

Char. 549: 1 --> 0

Char. 552: 1 --> 0

Char. 558: 0 --> 1

Char. 637: 1 --> 0

Char. 677: 0 --> 1

Char. 713: 1 --> 0

Char. 808: 2 --> 1

Char. 876: 1 --> 0

Alvarezsaurus_calvoi :

Char. 498: 1 --> 0

Char. 914: 3 --> 2

Char. 940: 0 --> 1

Char. 944: 0 --> 1

Alxasaurus_elesitaiensis :

Char. 436: 1 --> 0

Char. 613: 0 --> 2

Char. 735: 0 --> 1

Char. 748: 0 --> 1

Char. 853: 1 --> 2

Char. 960: 1 --> 0

Anchiornis_huxleyi :

Char. 13: 1 --> 2

Char. 16: 1 --> 0

Char. 17: 1 --> 0

Char. 19: 0 --> 1

Char. 30: 0 --> 1

Char. 32: 0 --> 1

Char. 37: 1 --> 0

Char. 49: 0 --> 1

Char. 57: 0 --> 1

Char. 69: 0 --> 2

Char. 71: 1 --> 0

Char. 80: 1 --> 0

Char. 91: 1 --> 0

Char. 98: 1 --> 2

Char. 100: 1 --> 0

Char. 111: 1 --> 0

Char. 135: 0 --> 1

Char. 145: 0 --> 1

Char. 164: 0 --> 1

Char. 166: 1 --> 0

Char. 184: 1 --> 0

Char. 186: 1 --> 0

Char. 198: 0 --> 2

Char. 201: 1 --> 0

Char. 216: 1 --> 0

Char. 228: 0 --> 1

Char. 250: 0 --> 1

Char. 254: 0 --> 1

Char. 262: 1 --> 0

Char. 266: 0 --> 1

Char. 320: 0 --> 1

Char. 357: 0 --> 1

Char. 362: 0 --> 1

Char. 397: 0 --> 1

Char. 409: 23 --> 1

Char. 412: 0 --> 1

Char. 413: 0 --> 1

Char. 440: 0 --> 1

Char. 526: 0 --> 1

Char. 629: 1 --> 0

Char. 701: 1 --> 0

Char. 741: 1 --> 0

Char. 761: 1 --> 2

Char. 803: 1 --> 0

Char. 816: 1 --> 0

Char. 850: 0 --> 1

Char. 853: 1 --> 2

Char. 949: 1 --> 2

Char. 950: 1 --> 0

Anserimimus_planinychus :

Char. 637: 1 --> 0

Char. 653: 1 --> 0

Char. 657: 1 --> 0

Char. 667: 0 --> 1

Char. 719: 1 --> 0

Char. 734: 2 --> 1

Char. 740: 0 --> 1

Char. 747: 1 --> 2

Char. 767: 1 --> 0

Char. 830: 2 --> 0

Char. 835: 1 --> 0

Char. 851: 1 --> 2

Char. 879: 1 --> 0

Char. 901: 0 --> 1

Char. 909: 1 --> 0

Char. 917: 0 --> 1

Anzu_wyliei :

Char. 21: 0 --> 1

Char. 23: 1 --> 2

Char. 44: 0 --> 1

Char. 48: 0 --> 1

Char. 58: 2 --> 0

Char. 127: 1 --> 2

Char. 132: 0 --> 1

Char. 225: 1 --> 0

Char. 231: 0 --> 1

Char. 247: 1 --> 0

Char. 253: 1 --> 0

Char. 274: 1 --> 0

Char. 328: 1 --> 0

Char. 382: 1 --> 0

Char. 496: 0 --> 1

Char. 529: 0 --> 1

Char. 532: 0 --> 1

Char. 539: 0 --> 1

Char. 543: 0 --> 1

Char. 587: 1 --> 0

Char. 598: 1 --> 2

Char. 610: 0 --> 1

Char. 660: 0 --> 1

Char. 672: 1 --> 2

Char. 694: 0 --> 1

Char. 819: 0 --> 1

Char. 853: 1 --> 2

Char. 854: 0 --> 1

Char. 906: 0 --> 1

Apsaravis_ukhaana :

Char. 342: 0 --> 1

Char. 343: 0 --> 1

Char. 571: 1 --> 0

Char. 587: 1 --> 0

Char. 659: 0 --> 1

Char. 676: 0 --> 1

Char. 773: 1 --> 0

Char. 819: 1 --> 0

Char. 829: 1 --> 0

Char. 848: 1 --> 0

Char. 860: 0 --> 1

Char. 954: 0 --> 1

Char. 955: 0 --> 1

Archaeopteryx :

Char. 13: 1 --> 2

Char. 15: 2 --> 3

Char. 16: 1 --> 0

Char. 17: 1 --> 0

Char. 18: 0 --> 1

Char. 19: 0 --> 1

Char. 20: 1 --> 0

Char. 30: 0 --> 1

Char. 32: 0 --> 1

Char. 37: 1 --> 0

Char. 45: 2 --> 3

Char. 49: 0 --> 1

Char. 56: 1 --> 0

Char. 70: 0 --> 1

Char. 76: 2 --> 1

Char. 80: 1 --> 0

Char. 100: 1 --> 0

Char. 104: 0 --> 1

Char. 108: 1 --> 0

Char. 120: 1 --> 0

Char. 127: 1 --> 0

Char. 132: 0 --> 1

Char. 140: 1 --> 0

Char. 159: 0 --> 1

Char. 160: 0 --> 1

Char. 164: 0 --> 1

Char. 170: 1 --> 0

Char. 196: 0 --> 1

Char. 214: 1 --> 0

Char. 215: 2 --> 0

Char. 228: 0 --> 1

Char. 230: 1 --> 0

Char. 246: 1 --> 0

Char. 247: 0 --> 1

Char. 253: 1 --> 0

Char. 259: 1 --> 0

Char. 266: 0 --> 1

Char. 273: 1 --> 0

Char. 274: 1 --> 0

Char. 278: 0 --> 1

Char. 283: 1 --> 0

Char. 287: 1 --> 0

Char. 288: 1 --> 0

Char. 290: 0 --> 1

Char. 310: 1 --> 0

Char. 316: 1 --> 2

Char. 320: 0 --> 1

Char. 325: 0 --> 1

Char. 366: 1 --> 0

Char. 396: 1 --> 0

Char. 402: 0 --> 1

Char. 409: 23 --> 0

Char. 412: 0 --> 1

Char. 413: 0 --> 1

Char. 414: 0 --> 1

Char. 416: 0 --> 2

Char. 422: 3 --> 2

Char. 423: 2 --> 1

Char. 431: 0 --> 1

Char. 439: 0 --> 1

Char. 440: 0 --> 1

Char. 444: 1 --> 0

Char. 459: 1 --> 0

Char. 481: 0 --> 2

Char. 487: 2 --> 0

Char. 491: 0 --> 1

Char. 526: 0 --> 1

Char. 532: 0 --> 1

Char. 585: 3 --> 2

Char. 620: 2 --> 1

Char. 655: 1 --> 2

Char. 675: 1 --> 0

Char. 676: 0 --> 1

Char. 701: 1 --> 0

Char. 717: 0 --> 1

Char. 735: 1 --> 0

Char. 741: 1 --> 0

Char. 753: 1 --> 2

Char. 787: 1 --> 0

Char. 793: 1 --> 0

Char. 796: 2 --> 1

Char. 807: 0 --> 1

Char. 819: 01 --> 2

Char. 820: 0 --> 2

Char. 830: 0 --> 3

Char. 836: 1 --> 2

Char. 852: 1 --> 3

Char. 853: 1 --> 2

Char. 899: 1 --> 0

Char. 913: 1 --> 0

Char. 925: 0 --> 1

Char. 939: 1 --> 0

Char. 942: 1 --> 0

Char. 949: 1 --> 2

Char. 950: 1 --> 0

Char. 959: 1 --> 2

Char. 963: 0 --> 1

Archaeornithomimus :

Char. 511: 0 --> 1

Char. 524: 0 --> 1

Char. 526: 0 --> 1

Char. 549: 2 --> 1

Char. 574: 3 --> 2

Char. 581: 0 --> 1

Char. 592: 0 --> 2

Char. 601: 0 --> 1

Char. 648: 0 --> 1

Char. 678: 0 --> 1

Char. 680: 0 --> 1

Char. 683: 1 --> 0

Char. 712: 0 --> 1

Char. 748: 1 --> 2

Char. 759: 1 --> 0

Char. 761: 0 --> 2

Char. 764: 1 --> 0

Char. 770: 0 --> 1

Char. 773: 1 --> 0

Char. 791: 1 --> 0

Char. 801: 0 --> 1

Char. 802: 0 --> 1

Char. 803: 1 --> 0

Char. 806: 1 --> 0

Char. 822: 1 --> 0

Char. 829: 0 --> 1

Char. 830: 1 --> 2

Char. 835: 1 --> 0

Char. 883: 1 --> 0

Char. 884: 1 --> 2

Char. 897: 2 --> 3

Char. 911: 0 --> 1

Char. 918: 1 --> 0

Char. 936: 0 --> 1

Char. 939: 0 --> 1

Char. 944: 0 --> 1

Asfaltovenator_vialidadi :

Char. 0: 1 --> 0

Char. 3: 1 --> 2

Char. 28: 0 --> 1

Char. 62: 1 --> 0

Char. 67: 0 --> 1

Char. 76: 1 --> 0

Char. 85: 0 --> 1

Char. 128: 0 --> 1

Char. 152: 0 --> 1

Char. 159: 0 --> 1

Char. 172: 0 --> 1

Char. 182: 0 --> 1

Char. 205: 1 --> 0

Char. 226: 1 --> 0

Char. 235: 0 --> 1

Char. 257: 1 --> 0

Char. 263: 0 --> 1

Char. 277: 0 --> 1

Char. 331: 0 --> 1

Char. 333: 0 --> 1

Char. 357: 1 --> 0

Char. 363: 0 --> 1

Char. 373: 1 --> 0

Char. 375: 0 --> 1

Char. 385: 1 --> 0

Char. 387: 1 --> 0

Char. 420: 1 --> 0

Char. 457: 1 --> 0

Char. 465: 1 --> 0

Char. 541: 1 --> 0

Char. 553: 0 --> 1

Char. 563: 1 --> 0

Char. 564: 0 --> 1

Char. 567: 0 --> 1

Char. 636: 2 --> 1

Char. 642: 1 --> 0

Char. 643: 0 --> 1

Char. 648: 0 --> 1

Char. 652: 1 --> 0

Char. 658: 0 --> 1

Char. 664: 0 --> 1

Char. 737: 0 --> 1

Char. 810: 0 --> 1

Char. 861: 1 --> 0

Char. 862: 1 --> 0

Char. 888: 0 --> 1

Char. 906: 1 --> 0

Aucasaurus_garridoi :

Char. 860: 0 --> 1

Char. 909: 0 --> 1

Australovenator_wintonensis :

No autapomorphies

Austroraptor_cabazai :

Char. 0: 1 --> 2

Char. 1: 1 --> 2

Char. 6: 0 --> 1

Char. 44: 1 --> 0

Char. 47: 1 --> 2

Char. 52: 0 --> 1

Char. 58: 2 --> 0

Char. 63: 1 --> 0

Char. 66: 0 --> 1

Char. 70: 0 --> 1

Char. 94: 0 --> 1

Char. 95: 1 --> 0

Char. 97: 0 --> 1

Char. 99: 1 --> 0

Char. 104: 0 --> 1

Char. 105: 1 --> 0

Char. 111: 1 --> 0

Char. 155: 1 --> 2

Char. 156: 0 --> 1

Char. 203: 0 --> 1

Char. 208: 1 --> 0

Char. 327: 1 --> 0

Char. 333: 0 --> 1

Char. 337: 0 --> 1

Char. 407: 0 --> 1

Char. 430: 0 --> 1

Char. 438: 0 --> 1

Char. 486: 0 --> 1

Char. 496: 0 --> 1

Char. 499: 1 --> 0

Char. 512: 0 --> 1

Char. 527: 1 --> 0

Char. 560: 0 --> 1

Char. 694: 0 --> 1

Char. 855: 2 --> 1

Char. 952: 0 --> 1

Char. 953: 0 --> 1

Char. 954: 0 --> 1

Char. 959: 1 --> 2

Char. 964: 0 --> 1

Avimimus :

Char. 10: 1 --> 2

Char. 15: 2 --> 1

Char. 33: 0 --> 2

Char. 179: 1 --> 0

Char. 228: 0 --> 1

Char. 232: 1 --> 0

Char. 234: 1 --> 0

Char. 243: 1 --> 0

Char. 250: 0 --> 1

Char. 255: 0 --> 1

Char. 272: 0 --> 1

Char. 298: 0 --> 1

Char. 300: 0 --> 1

Char. 330: 2 --> 0

Char. 332: 0 --> 1

Char. 344: 2 --> 1

Char. 346: 1 --> 0

Char. 496: 0 --> 1

Char. 498: 1 --> 0

Char. 513: 1 --> 2

Char. 536: 2 --> 0

Char. 539: 0 --> 1

Char. 545: 0 --> 1

Char. 565: 1 --> 0

Char. 574: 3 --> 4

Char. 592: 2 --> 0

Char. 593: 2 --> 0

Char. 637: 1 --> 0

Char. 685: 0 --> 1

Char. 699: 0 --> 1

Char. 749: 0 --> 1

Char. 750: 0 --> 1

Char. 752: 0 --> 1

Char. 762: 0 --> 1

Char. 778: 1 --> 0

Char. 783: 0 --> 1

Char. 803: 1 --> 0

Char. 836: 1 --> 0

Char. 839: 1 --> 2

Char. 852: 1 --> 3

Char. 855: 2 --> 1

Char. 883: 1 --> 0

Char. 900: 1 --> 2

Char. 902: 0 --> 1

Char. 906: 0 --> 1

Char. 925: 0 --> 1

Char. 926: 0 --> 1

Char. 936: 01 --> 2

Char. 949: 1 --> 0

Baryonyx_walkeri :

Char. 203: 0 --> 1

Char. 241: 1 --> 0

Char. 244: 0 --> 1

Char. 249: 0 --> 1

Char. 262: 0 --> 1

Char. 296: 1 --> 0

Char. 337: 1 --> 0

Char. 351: 1 --> 0

Char. 390: 1 --> 0

Char. 398: 0 --> 1

Char. 409: 12 --> 3

Char. 416: 1 --> 0

Char. 422: 1 --> 3

Char. 425: 2 --> 0

Char. 427: 2 --> 0

Char. 461: 0 --> 1

Char. 471: 0 --> 1

Char. 473: 1 --> 0

Char. 487: 0 --> 1

Char. 516: 1 --> 0

Char. 540: 0 --> 1

Char. 548: 0 --> 2

Char. 550: 01 --> 2

Char. 551: 0 --> 1

Char. 678: 0 --> 1

Char. 690: 0 --> 1

Char. 861: 0 --> 1

Beipiaosaurus_inexpectus :

Char. 47: 1 --> 2

Char. 56: 1 --> 0

Char. 330: 0 --> 1

Char. 637: 1 --> 0

Char. 664: 0 --> 12

Char. 716: 0 --> 1

Char. 853: 1 --> 2

Berthasaura_leopoldinae :

Char. 155: 1 --> 2

Char. 203: 0 --> 1

Char. 206: 0 --> 1

Char. 339: 0 --> 1

Char. 345: 1 --> 2

Char. 408: 0 --> 1

Char. 455: 0 --> 12

Char. 483: 1 --> 0

Char. 513: 2 --> 01

Char. 515: 0 --> 1

Char. 536: 1 --> 0

Char. 577: 1 --> 0

Char. 578: 0 --> 1

Char. 593: 0 --> 2

Char. 656: 1 --> 0

Char. 660: 1 --> 0

Char. 667: 1 --> 0

Char. 672: 2 --> 01

Char. 676: 0 --> 1

Char. 678: 0 --> 1

Char. 761: 0 --> 12

Char. 777: 0 --> 1

Char. 791: 0 --> 1

Char. 809: 1 --> 0

Char. 835: 0 --> 2

Char. 851: 0 --> 1

Char. 886: 0 --> 2

Char. 901: 0 --> 1

Bicentenaria_argentina :

Char. 28: 0 --> 1

Char. 178: 1 --> 0

Char. 276: 1 --> 0

Char. 536: 0 --> 1

Char. 539: 0 --> 1

Char. 564: 0 --> 1

Char. 580: 1 --> 0

Char. 614: 1 --> 0

Char. 676: 0 --> 1

Char. 840: 1 --> 0

Char. 842: 0 --> 1

Char. 848: 0 --> 12

Char. 849: 0 --> 1

Char. 883: 1 --> 0

Char. 911: 0 --> 1

Char. 915: 0 --> 1

Char. 917: 0 --> 1

Char. 919: 1 --> 0

Bistahieversor_sealeyi :

Char. 8: 0 --> 1

Char. 72: 1 --> 0

Char. 90: 0 --> 1

Char. 148: 1 --> 0

Char. 153: 0 --> 1

Char. 196: 0 --> 1

Char. 361: 0 --> 1

Char. 422: 3 --> 2

Buitreraptor_gonzalezorum :

Char. 0: 1 --> 2

Char. 1: 1 --> 2

Char. 47: 1 --> 2

Char. 66: 0 --> 1

Char. 145: 0 --> 1

Char. 155: 1 --> 2

Char. 156: 0 --> 1

Char. 177: 0 --> 1

Char. 214: 1 --> 0

Char. 216: 1 --> 0

Char. 327: 1 --> 0

Char. 357: 0 --> 1

Char. 363: 0 --> 2

Char. 407: 0 --> 1

Char. 419: 0 --> 1

Char. 430: 0 --> 1

Char. 434: 1 --> 2

Char. 486: 0 --> 1

Char. 496: 0 --> 1

Char. 498: 1 --> 2

Char. 506: 1 --> 0

Char. 527: 1 --> 0

Char. 549: 1 --> 2

Char. 553: 0 --> 1

Char. 557: 0 --> 1

Char. 641: 0 --> 1

Char. 655: 1 --> 2

Char. 657: 0 --> 1

Char. 676: 0 --> 1

Char. 729: 1 --> 0

Char. 738: 0 --> 1

Char. 760: 0 --> 1

Char. 771: 0 --> 1

Char. 774: 0 --> 1

Char. 783: 0 --> 1

Char. 803: 1 --> 0

Char. 824: 0 --> 1

Char. 825: 2 --> 1

Char. 831: 0 --> 2

Char. 839: 1 --> 0

Char. 855: 2 --> 1

Char. 902: 0 --> 1

Char. 911: 0 --> 1

Char. 912: 0 --> 1

Char. 935: 0 --> 1

Char. 936: 0 --> 1

Char. 948: 0 --> 1

Char. 960: 1 --> 2

Byronosaurus_jaffei :

Char. 3: 1 --> 0

Char. 15: 2 --> 1

Char. 23: 2 --> 1

Char. 30: 0 --> 1

Char. 38: 0 --> 2

Char. 44: 1 --> 0

Char. 47: 1 --> 2

Char. 62: 1 --> 0

Char. 66: 0 --> 1

Char. 76: 2 --> 1

Char. 84: 1 --> 0

Char. 100: 1 --> 0

Char. 103: 0 --> 1

Char. 254: 0 --> 1

Char. 256: 2 --> 0

Char. 273: 1 --> 0

Char. 280: 0 --> 1

Char. 287: 1 --> 0

Char. 291: 1 --> 0

Char. 327: 1 --> 0

Char. 334: 0 --> 1

Char. 402: 0 --> 2

Char. 414: 0 --> 1

Char. 416: 0 --> 12

Char. 417: 12 --> 0

Char. 418: 1 --> 0

Char. 422: 3 --> 2

Char. 423: 2 --> 3

Char. 434: 1 --> 3

Char. 435: 0 --> 1

Char. 441: 0 --> 2

Char. 503: 1 --> 0

Char. 513: 1 --> 2

Char. 614: 1 --> 2

Char. 852: 1 --> 2

Char. 873: 0 --> 1

Carcharodontosaurus :

No autapomorphies

Carnotaurus_sastrei :

Char. 1: 1 --> 0

Char. 8: 0 --> 1

Char. 10: 0 --> 1

Char. 13: 1 --> 0

Char. 23: 1 --> 0

Char. 47: 1 --> 0

Char. 90: 0 --> 1

Char. 134: 0 --> 1

Char. 153: 0 --> 2

Char. 155: 0 --> 1

Char. 158: 0 --> 1

Char. 175: 0 --> 1

Char. 185: 1 --> 0

Char. 229: 0 --> 1

Char. 247: 0 --> 1

Char. 350: 0 --> 1

Char. 399: 1 --> 0

Char. 409: 2 --> 1

Char. 519: 0 --> 1

Char. 541: 0 --> 2

Char. 595: 0 --> 1

Char. 761: 0 --> 1

Char. 768: 0 --> 1

Caudipteryx :

Char. 15: 2 --> 3

Char. 19: 0 --> 1

Char. 38: 0 --> 2

Char. 63: 1 --> 0

Char. 187: 0 --> 1

Char. 334: 0 --> 1

Char. 353: 0 --> 2

Char. 397: 0 --> 1

Char. 401: 0 --> 1

Char. 404: 2 --> 1

Char. 585: 3 --> 2

Char. 646: 12 --> 0

Char. 707: 1 --> 0

Char. 717: 0 --> 1

Char. 762: 0 --> 1

Char. 849: 0 --> 1

Char. 851: 1 --> 2

Char. 852: 1 --> 3

Ceratosaurus :

Char. 84: 1 --> 0

Char. 96: 1 --> 3

Char. 110: 2 --> 1

Char. 118: 1 --> 0

Char. 123: 1 --> 0

Char. 159: 0 --> 1

Char. 198: 1 --> 2

Char. 247: 0 --> 1

Char. 494: 1 --> 0

Char. 573: 0 --> 1

Char. 584: 0 --> 1

Char. 654: 0 --> 1

Char. 715: 0 --> 1

Char. 801: 1 --> 0

Char. 842: 0 --> 1

Char. 844: 0 --> 1

Char. 852: 2 --> 1

Char. 862: 1 --> 2

Char. 863: 1 --> 0

Char. 882: 0 --> 1

Char. 904: 1 --> 2

Char. 927: 0 --> 1

Char. 957: 1 --> 0

Citipati_osmolskae :

Char. 9: 1 --> 2

Char. 10: 1 --> 0

Char. 15: 2 --> 1

Char. 20: 1 --> 0

Char. 22: 1 --> 0

Char. 37: 1 --> 0

Char. 90: 0 --> 1

Char. 154: 0 --> 1

Char. 160: 0 --> 1

Char. 199: 0 --> 1

Char. 575: 1 --> 2

Char. 629: 0 --> 1

Coelophysis_bauri :

Char. 3: 0 --> 1

Char. 29: 1 --> 0

Char. 35: 1 --> 0

Char. 45: 2 --> 3

Char. 208: 0 --> 1

Char. 273: 1 --> 0

Char. 347: 0 --> 1

Char. 409: 2 --> 3

Char. 457: 0 --> 1

Char. 479: 2 --> 0

Char. 661: 1 --> 0

Char. 682: 0 --> 1

Char. 750: 1 --> 0

Megapnosaurus_rhodesiensis :

Char. 512: 1 --> 0

Char. 536: 1 --> 0

Char. 788: 0 --> 1

"Syntarsus"_kayentakatae :

Char. 79: 0 --> 1

Char. 112: 0 --> 1

Char. 124: 1 --> 0

Char. 139: 1 --> 0

Char. 140: 0 --> 1

Char. 173: 0 --> 1

Char. 198: 0 --> 1

Char. 199: 0 --> 1

Char. 257: 0 --> 1

Char. 389: 1 --> 2

Char. 412: 0 --> 1

Char. 492: 0 --> 1

Char. 513: 2 --> 1

Char. 577: 0 --> 1

Char. 853: 0 --> 1

Char. 872: 0 --> 1

Char. 926: 0 --> 1

Segisaurus_halli :

Char. 628: 0 --> 1

Char. 636: 1 --> 2

Char. 637: 0 --> 1

Char. 646: 0 --> 1

Char. 673: 0 --> 1

Char. 828: 0 --> 1

Char. 861: 0 --> 1

Char. 903: 1 --> 0

Coelurus_fragilis :

Char. 505: 0 --> 1

Char. 536: 0 --> 1

Char. 598: 1 --> 0

Char. 599: 1 --> 0

Char. 606: 0 --> 1

Char. 641: 1 --> 0

Char. 670: 0 --> 1

Char. 676: 0 --> 1

Char. 679: 1 --> 2

Char. 680: 0 --> 1

Char. 716: 1 --> 0

Char. 803: 1 --> 0

Char. 814: 0 --> 1

Char. 840: 1 --> 0

Char. 855: 1 --> 0

Char. 870: 0 --> 1

Char. 903: 1 --> 0

Char. 915: 0 --> 1

Compsognathus_longipes :

Char. 15: 1 --> 3

Char. 16: 1 --> 0

Char. 30: 0 --> 1

Char. 76: 1 --> 0

Char. 126: 1 --> 0

Char. 335: 0 --> 1

Char. 395: 1 --> 0

Char. 409: 1 --> 2

Char. 417: 12 --> 3

Char. 422: 3 --> 2

Char. 425: 2 --> 1

Char. 498: 1 --> 0

Char. 505: 0 --> 1

Char. 541: 1 --> 0

Char. 549: 1 --> 2

Char. 592: 2 --> 0

Char. 599: 1 --> 0

Char. 618: 1 --> 0

Char. 658: 0 --> 1

Char. 707: 1 --> 0

Char. 777: 1 --> 0

Char. 780: 1 --> 0

Char. 808: 2 --> 0

Char. 815: 0 --> 1

Char. 911: 0 --> 1

Char. 912: 0 --> 1

Concavenator_corcovatus :

Char. 109: 1 --> 2

Char. 127: 2 --> 1

Char. 149: 0 --> 2

Char. 173: 1 --> 0

Char. 192: 0 --> 1

Char. 532: 1 --> 2

Char. 561: 0 --> 1

Char. 562: 0 --> 1

Char. 609: 0 --> 2

Char. 640: 2 --> 1

Char. 680: 0 --> 1

Char. 689: 0 --> 1

Char. 691: 2 --> 1

Char. 695: 1 --> 0

Char. 714: 1 --> 0

Char. 734: 1 --> 2

Char. 759: 0 --> 2

Char. 764: 1 --> 0

Char. 784: 0 --> 1

Char. 837: 0 --> 1

Char. 946: 2 --> 1

Char. 956: 2 --> 1

Conchoraptor_gracilis :

Char. 127: 0 --> 1

Char. 224: 0 --> 1

Char. 261: 0 --> 1

Char. 457: 1 --> 0

Char. 562: 0 --> 1

Char. 744: 1 --> 0

Char. 763: 1 --> 0

Char. 764: 1 --> 2

Char. 787: 0 --> 1

Char. 803: 3 --> 2

Char. 951: 0 --> 1

Condorraptor_currumili :

Char. 487: 0 --> 1

Char. 539: 0 --> 1

Char. 546: 0 --> 1

Confuciusornis_sanctus :

Char. 0: 1 --> 2

Char. 9: 0 --> 1

Char. 19: 1 --> 2

Char. 124: 1 --> 0

Char. 322: 1 --> 2

Char. 334: 0 --> 1

Char. 355: 0 --> 1

Char. 553: 0 --> 1

Char. 631: 0 --> 1

Char. 676: 0 --> 1

Char. 739: 0 --> 1

Char. 759: 1 --> 2

Char. 918: 1 --> 0

Char. 927: 0 --> 1

Char. 934: 0 --> 1

Char. 949: 1 --> 2

Cryolophosaurus_ellioti :

Char. 96: 1 --> 3

Char. 105: 0 --> 1

Char. 155: 1 --> 2

Char. 165: 0 --> 1

Char. 194: 0 --> 1

Char. 205: 1 --> 0

Char. 219: 0 --> 1

Char. 223: 0 --> 1

Char. 226: 1 --> 0

Char. 262: 0 --> 1

Char. 270: 1 --> 0

Char. 290: 2 --> 0

Char. 487: 01 --> 2

Char. 550: 0 --> 2

Char. 565: 0 --> 1

Char. 615: 1 --> 0

Char. 661: 1 --> 2

Char. 664: 0 --> 1

Char. 678: 0 --> 1

Char. 852: 2 --> 1

Char. 873: 0 --> 1

Char. 877: 1 --> 0

Daspletosaurus :

Char. 72: 1 --> 0

Char. 76: 2 --> 1

Char. 133: 0 --> 1

Char. 154: 1 --> 0

Char. 191: 0 --> 1

Char. 196: 0 --> 1

Char. 221: 1 --> 0

Char. 353: 2 --> 1

Char. 363: 1 --> 2

Char. 463: 0 --> 1

Char. 837: 0 --> 1

Char. 858: 0 --> 1

Char. 953: 0 --> 1

Char. 955: 1 --> 2

Deinocheirus :

Char. 2: 1 --> 3

Char. 18: 0 --> 1

Char. 21: 0 --> 1

Char. 23: 1 --> 0

Char. 30: 0 --> 1

Char. 38: 0 --> 2

Char. 58: 12 --> 0

Char. 94: 0 --> 1

Char. 95: 1 --> 0

Char. 99: 1 --> 0

Char. 101: 0 --> 1

Char. 108: 12 --> 0

Char. 124: 1 --> 0

Char. 164: 0 --> 1

Char. 186: 0 --> 1

Char. 229: 1 --> 0

Char. 231: 0 --> 1

Char. 323: 1 --> 0

Char. 327: 1 --> 2

Char. 381: 0 --> 1

Char. 433: 0 --> 1

Char. 481: 0 --> 1

Char. 498: 1 --> 2

Char. 499: 0 --> 1

Char. 506: 0 --> 1

Char. 508: 0 --> 1

Char. 509: 0 --> 2

Char. 528: 0 --> 2

Char. 536: 0 --> 2

Char. 555: 0 --> 2

Char. 557: 0 --> 2

Char. 558: 0 --> 1

Char. 559: 0 --> 1

Char. 582: 0 --> 1

Char. 598: 1 --> 0

Char. 599: 1 --> 0

Char. 604: 0 --> 1

Char. 631: 0 --> 1

Char. 640: 0 --> 1

Char. 652: 1 --> 0

Char. 656: 1 --> 0

Char. 657: 1 --> 0

Char. 673: 1 --> 0

Char. 678: 0 --> 1

Char. 679: 1 --> 0

Char. 693: 2 --> 01

Char. 718: 1 --> 0

Char. 736: 1 --> 0

Char. 737: 1 --> 0

Char. 738: 1 --> 0

Char. 741: 0 --> 1

Char. 747: 1 --> 0

Char. 748: 1 --> 0

Char. 757: 0 --> 1

Char. 759: 1 --> 0

Char. 761: 0 --> 1

Char. 764: 1 --> 0

Char. 766: 0 --> 1

Char. 769: 1 --> 0

Char. 795: 0 --> 1

Char. 796: 1 --> 0

Char. 803: 1 --> 2

Char. 810: 0 --> 1

Char. 812: 1 --> 0

Char. 837: 1 --> 0

Char. 840: 1 --> 0

Char. 841: 0 --> 1

Char. 876: 1 --> 2

Char. 877: 0 --> 1

Char. 882: 1 --> 0

Char. 884: 1 --> 2

Char. 902: 1 --> 0

Char. 917: 0 --> 1

Char. 938: 1 --> 0

Char. 947: 1 --> 2

Char. 950: 0 --> 1

Char. 951: 0 --> 1

Char. 952: 01 --> 2

Char. 954: 1 --> 2

Char. 960: 0 --> 1

Deinonychus_antirrhopus :

Char. 0: 1 --> 2

Char. 22: 1 --> 0

Char. 37: 1 --> 0

Char. 56: 1 --> 0

Char. 65: 1 --> 2

Char. 69: 0 --> 1

Char. 99: 1 --> 0

Char. 120: 1 --> 0

Char. 127: 1 --> 2

Char. 155: 1 --> 2

Char. 186: 1 --> 0

Char. 198: 2 --> 1

Char. 225: 1 --> 0

Char. 357: 0 --> 1

Char. 409: 1 --> 2

Char. 419: 1 --> 0

Char. 479: 0 --> 1

Char. 510: 1 --> 0

Char. 540: 1 --> 0

Char. 563: 0 --> 1

Char. 708: 0 --> 1

Char. 779: 1 --> 0

Char. 788: 0 --> 1

Char. 810: 1 --> 0

Char. 913: 1 --> 0

Char. 946: 2 --> 1

Char. 954: 0 --> 1

Char. 955: 0 --> 1

Char. 956: 0 --> 1

Deltadromeus_agilis :

Char. 617: 0 --> 1

Char. 620: 0 --> 1

Char. 631: 0 --> 1

Char. 649: 0 --> 1

Char. 650: 1 --> 0

Char. 652: 1 --> 0

Char. 660: 0 --> 1

Char. 835: 1 --> 0

Char. 839: 1 --> 2

Char. 848: 0 --> 1

Char. 854: 1 --> 2

Char. 860: 0 --> 1

Char. 862: 0 --> 1

Char. 867: 0 --> 1

Char. 870: 0 --> 1

Char. 874: 0 --> 1

Char. 879: 1 --> 0

Char. 884: 1 --> 2

Char. 886: 2 --> 1

Char. 900: 1 --> 2

Char. 917: 0 --> 1

Char. 919: 0 --> 1

Char. 930: 1 --> 0

Char. 938: 1 --> 0

Char. 943: 0 --> 1

Char. 955: 2 --> 1

Dilong_paradoxus :

Char. 20: 1 --> 0

Char. 38: 0 --> 1

Char. 52: 0 --> 1

Char. 63: 1 --> 0

Char. 71: 1 --> 0

Char. 96: 0 --> 1

Char. 103: 0 --> 1

Char. 159: 0 --> 1

Char. 182: 0 --> 1

Char. 184: 0 --> 1

Char. 187: 0 --> 1

Char. 192: 0 --> 1

Char. 215: 0 --> 2

Char. 276: 1 --> 0

Char. 380: 0 --> 1

Char. 411: 0 --> 12

Char. 494: 1 --> 0

Char. 635: 0 --> 1

Char. 636: 2 --> 1

Char. 672: 1 --> 2

Char. 688: 1 --> 0

Char. 725: 1 --> 0

Char. 761: 2 --> 1

Char. 763: 1 --> 0

Char. 775: 1 --> 0

Char. 780: 1 --> 0

Char. 838: 0 --> 1

Char. 843: 1 --> 0

Char. 879: 1 --> 0

Sinosaurus_triassicus :

Char. 0: 1 --> 2

Char. 15: 2 --> 1

Char. 23: 1 --> 0

Char. 63: 1 --> 0

Char. 94: 0 --> 1

Char. 128: 0 --> 1

Char. 141: 0 --> 1

Char. 160: 1 --> 0

Char. 218: 0 --> 1

Char. 222: 0 --> 1

Char. 248: 1 --> 0

Char. 278: 0 --> 1

Char. 283: 0 --> 1

Char. 363: 0 --> 1

Char. 602: 0 --> 1

Char. 652: 1 --> 0

Char. 655: 1 --> 0

Char. 760: 0 --> 1

Char. 803: 1 --> 0

Char. 830: 0 --> 1

Char. 887: 0 --> 1

Char. 889: 0 --> 1

Char. 908: 1 --> 0

Char. 944: 0 --> 1

Char. 955: 1 --> 2

Dilophosaurus_wetherilli :

Char. 20: 1 --> 0

Char. 23: 1 --> 2

Char. 30: 0 --> 1

Char. 79: 0 --> 1

Char. 80: 0 --> 1

Char. 83: 0 --> 1

Char. 98: 0 --> 12

Char. 110: 0 --> 2

Char. 119: 0 --> 1

Char. 161: 0 --> 1

Char. 165: 0 --> 1

Char. 166: 0 --> 1

Char. 194: 0 --> 1

Char. 195: 0 --> 1

Char. 207: 0 --> 1

Char. 252: 0 --> 1

Char. 256: 0 --> 1

Char. 257: 0 --> 1

Char. 340: 0 --> 1

Char. 399: 1 --> 2

Char. 400: 1 --> 2

Char. 442: 0 --> 1

Char. 457: 0 --> 1

Char. 561: 0 --> 1

Char. 567: 0 --> 1

Char. 640: 02 --> 1

Char. 642: 1 --> 0

Char. 661: 1 --> 0

Char. 881: 1 --> 0

Char. 920: 0 --> 1

Dromaeosaurus_albertensis :

Char. 3: 0 --> 1

Char. 38: 1 --> 0

Char. 88: 0 --> 1

Char. 114: 0 --> 1

Char. 127: 1 --> 2

Char. 137: 0 --> 1

Char. 198: 2 --> 1

Char. 231: 0 --> 1

Char. 268: 4 --> 0

Char. 270: 1 --> 0

Char. 273: 1 --> 0

Char. 321: 0 --> 1

Char. 377: 1 --> 0

Char. 381: 0 --> 1

Char. 402: 0 --> 1

Char. 409: 1 --> 0

Char. 417: 0 --> 1

Char. 425: 1 --> 0

Char. 428: 1 --> 0

Dubreuillosaurus_valesdunensis :

Char. 54: 0 --> 1

Char. 67: 0 --> 1

Char. 112: 1 --> 0

Char. 203: 0 --> 1

Char. 252: 0 --> 1

Char. 295: 0 --> 1

Char. 606: 0 --> 1

Char. 608: 0 --> 1

Char. 637: 1 --> 0

Char. 839: 1 --> 0

Char. 844: 1 --> 0

Char. 868: 1 --> 0

Ekrixinatosaurus_novasi :

Char. 47: 1 --> 0

Char. 212: 0 --> 1

Char. 241: 1 --> 2

Char. 242: 1 --> 0

Char. 795: 1 --> 0

Char. 854: 1 --> 0

Char. 856: 0 --> 1

Char. 874: 1 --> 0

Char. 915: 2 --> 1

Elaphrosaurus_bambergi :

Char. 483: 1 --> 0

Char. 485: 1 --> 0

Char. 505: 1 --> 0

Char. 523: 0 --> 1

Char. 576: 0 --> 1

Char. 599: 1 --> 0

Char. 609: 2 --> 3

Char. 653: 0 --> 1

Char. 654: 0 --> 1

Char. 750: 0 --> 1

Char. 757: 0 --> 1

Char. 781: 0 --> 1

Char. 796: 0 --> 1

Char. 854: 0 --> 1

Char. 868: 0 --> 1

Char. 878: 1 --> 0

Char. 884: 1 --> 2

Char. 921: 1 --> 0

Char. 937: 0 --> 2

Char. 954: 1 --> 0

Eoabelisaurus_mefi :

Char. 93: 1 --> 0

Char. 96: 1 --> 0

Char. 126: 0 --> 1

Char. 153: 1 --> 0

Char. 179: 0 --> 1

Char. 212: 0 --> 1

Char. 213: 0 --> 1

Char. 238: 0 --> 1

Char. 242: 1 --> 0

Char. 252: 0 --> 1

Char. 276: 1 --> 0

Char. 284: 0 --> 1

Char. 514: 1 --> 0

Char. 592: 2 --> 0

Char. 595: 0 --> 1

Char. 640: 0 --> 1

Char. 683: 1 --> 2

Char. 695: 0 --> 1

Char. 768: 0 --> 1

Char. 799: 0 --> 1

Char. 809: 1 --> 0

Char. 843: 1 --> 0

Char. 851: 0 --> 1

Char. 868: 0 --> 1

Char. 895: 0 --> 1

Char. 896: 0 --> 1

Eotyrannus_lengi :

Char. 187: 0 --> 1

Char. 385: 1 --> 0

Char. 422: 3 --> 2

Char. 480: 1 --> 0

Char. 487: 0 --> 12

Char. 549: 1 --> 2

Char. 651: 0 --> 1

Char. 675: 0 --> 1

Char. 707: 1 --> 0

Char. 726: 2 --> 1

Char. 888: 0 --> 1

Erlikosaurus_andrewsi :

Char. 231: 0 --> 1

Char. 234: 1 --> 0

Char. 270: 1 --> 0

Char. 285: 1 --> 0

Char. 288: 2 --> 0

Char. 340: 0 --> 1

Char. 342: 1 --> 0

Char. 406: 1 --> 0

Char. 949: 1 --> 0

Eustreptospondylus_oxoniensis :

Char. 29: 0 --> 1

Char. 58: 2 --> 0

Char. 61: 2 --> 1

Char. 82: 1 --> 0

Char. 108: 1 --> 0

Char. 113: 1 --> 0

Char. 160: 1 --> 0

Char. 248: 1 --> 0

Char. 254: 1 --> 0

Char. 263: 0 --> 1

Char. 293: 0 --> 1

Char. 541: 1 --> 0

Char. 542: 0 --> 1

Falcarius_utahensis :

Char. 178: 1 --> 0

Char. 182: 0 --> 1

Char. 339: 0 --> 1

Char. 340: 0 --> 1

Char. 486: 0 --> 1

Char. 507: 1 --> 0

Char. 534: 0 --> 1

Char. 536: 0 --> 2

Char. 549: 1 --> 2

Char. 557: 0 --> 1

Char. 560: 0 --> 1

Char. 598: 1 --> 0

Char. 617: 0 --> 1

Char. 620: 0 --> 1

Char. 632: 0 --> 1

Char. 656: 0 --> 1

Char. 670: 0 --> 1

Char. 672: 1 --> 0

Char. 680: 0 --> 1

Char. 735: 0 --> 1

Char. 764: 1 --> 0

Char. 774: 0 --> 1

Char. 813: 0 --> 1

Char. 815: 0 --> 1

Char. 816: 1 --> 0

Char. 874: 0 --> 1

Char. 893: 0 --> 1

Char. 949: 1 --> 2

Char. 959: 1 --> 2

Fukuiraptor_kitadaniensis :

Char. 659: 0 --> 1

Char. 661: 0 --> 2

Char. 667: 1 --> 0

Char. 678: 1 --> 0

Char. 682: 1 --> 0

Char. 874: 0 --> 1

Char. 914: 3 --> 2

Fukuivenator_paradoxus :

Char. 51: 0 --> 1

Char. 63: 1 --> 0

Char. 68: 0 --> 1

Char. 70: 0 --> 1

Char. 93: 1 --> 0

Char. 104: 0 --> 1

Char. 108: 1 --> 0

Char. 110: 0 --> 1

Char. 177: 0 --> 1

Char. 209: 0 --> 1

Char. 238: 0 --> 1

Char. 271: 0 --> 1

Char. 420: 1 --> 2

Char. 427: 0 --> 2

Char. 445: 0 --> 1

Char. 461: 1 --> 0

Char. 464: 1 --> 0

Char. 478: 1 --> 2

Char. 486: 0 --> 1

Char. 488: 0 --> 1

Char. 496: 0 --> 1

Char. 506: 1 --> 2

Char. 531: 0 --> 1

Char. 543: 0 --> 1

Char. 549: 1 --> 2

Char. 567: 0 --> 1

Char. 577: 0 --> 1

Char. 578: 0 --> 2

Char. 588: 0 --> 1

Char. 590: 0 --> 1

Char. 599: 1 --> 2

Char. 606: 0 --> 1

Char. 610: 0 --> 1

Char. 635: 0 --> 1

Char. 636: 2 --> 1

Char. 657: 0 --> 1

Char. 670: 0 --> 1

Char. 671: 0 --> 1

Char. 680: 0 --> 1

Char. 710: 0 --> 1

Char. 734: 1 --> 2

Char. 806: 1 --> 0

Char. 848: 0 --> 2

Char. 893: 0 --> 1

Char. 917: 0 --> 1

Char. 958: 0 --> 1

Gallimimus_bullatus :

Char. 15: 2 --> 3

Char. 29: 0 --> 2

Char. 94: 0 --> 1

Char. 103: 0 --> 1

Char. 114: 0 --> 1

Char. 179: 1 --> 0

Char. 230: 0 --> 1

Char. 231: 0 --> 1

Char. 234: 0 --> 1

Char. 381: 0 --> 1

Char. 534: 0 --> 1

Char. 541: 1 --> 0

Char. 575: 0 --> 12

Char. 646: 1 --> 0

Char. 653: 1 --> 0

Char. 689: 0 --> 1

Char. 726: 1 --> 2

Char. 748: 2 --> 0

Char. 762: 1 --> 0

Char. 767: 1 --> 0

Char. 908: 1 --> 0

Gallus :

Char. 325: 0 --> 1

Char. 553: 0 --> 1

Char. 571: 1 --> 2

Char. 591: 1 --> 0

Char. 657: 0 --> 1

Char. 661: 0 --> 2

Char. 681: 1 --> 0

Char. 683: 0 --> 1

Char. 751: 0 --> 1

Char. 769: 1 --> 2

Char. 783: 0 --> 1

Char. 828: 0 --> 1

Char. 833: 0 --> 1

Char. 924: 0 --> 1

Char. 936: 0 --> 2

Char. 939: 1 --> 0

Char. 941: 0 --> 1

Char. 942: 0 --> 1

Garudimimus_brevipes :

Char. 20: 1 --> 0

Char. 23: 1 --> 0

Char. 63: 1 --> 0

Char. 65: 0 --> 2

Char. 158: 1 --> 0

Char. 159: 0 --> 1

Char. 255: 0 --> 1

Char. 256: 0 --> 2

Char. 285: 1 --> 2

Char. 322: 1 --> 0

Char. 333: 0 --> 1

Char. 334: 0 --> 1

Char. 360: 1 --> 0

Char. 433: 0 --> 1

Char. 457: 1 --> 0

Char. 469: 0 --> 1

Char. 544: 0 --> 1

Char. 598: 1 --> 0

Char. 760: 0 --> 1

Char. 806: 1 --> 0

Char. 854: 1 --> 2

Char. 860: 0 --> 1

Char. 868: 0 --> 1

Char. 877: 0 --> 1

Char. 908: 1 --> 0

Char. 937: 0 --> 2

Char. 940: 0 --> 1

Giganotosaurus_carolinii :

Char. 113: 1 --> 0

Char. 338: 0 --> 1

Char. 467: 1 --> 0

Char. 479: 3 --> 2

Char. 552: 1 --> 0

Char. 589: 1 --> 0

Char. 610: 1 --> 0

Char. 639: 0 --> 1

Char. 767: 0 --> 1

Char. 853: 1 --> 0

Char. 856: 0 --> 1

Gigantoraptor_erlianensis :

Char. 330: 2 --> 0

Char. 332: 0 --> 1

Char. 345: 2 --> 1

Char. 587: 1 --> 0

Char. 657: 0 --> 1

Char. 660: 0 --> 1

Char. 852: 1 --> 2

Char. 867: 0 --> 1

Char. 868: 0 --> 1

Gnathovorax_cabreirai :

Char. 22: 0 --> 1

Char. 96: 0 --> 1

Char. 108: 1 --> 0

Char. 136: 0 --> 1

Char. 158: 0 --> 1

Char. 241: 0 --> 2

Char. 275: 0 --> 1

Char. 309: 0 --> 1

Char. 376: 1 --> 0

Char. 395: 1 --> 0

Char. 407: 0 --> 1

Char. 425: 0 --> 1

Char. 430: 0 --> 1

Char. 586: 0 --> 1

Char. 605: 0 --> 1

Char. 789: 1 --> 0

Char. 877: 0 --> 1

Char. 895: 0 --> 1

Tawa_hallae :

Char. 2: 1 --> 0

Char. 3: 0 --> 1

Char. 10: 0 --> 2

Char. 11: 0 --> 2

Char. 23: 0 --> 1

Char. 29: 0 --> 1

Char. 30: 0 --> 1

Char. 36: 0 --> 1

Char. 43: 0 --> 1

Char. 80: 0 --> 1

Char. 116: 0 --> 1

Char. 128: 1 --> 0

Char. 170: 0 --> 1

Char. 231: 0 --> 1

Char. 232: 1 --> 0

Char. 290: 1 --> 2

Char. 342: 1 --> 0

Char. 360: 1 --> 0

Char. 409: 2 --> 1

Char. 505: 0 --> 1

Char. 636: 1 --> 2

Char. 683: 1 --> 0

Char. 700: 0 --> 1

Char. 703: 1 --> 0

Char. 713: 1 --> 0

Char. 803: 1 --> 0

Char. 848: 2 --> 0

Char. 868: 0 --> 1

Char. 920: 0 --> 1

Herrerasaurus_ischigualastensis :

Char. 17: 0 --> 1

Char. 117: 1 --> 0

Char. 247: 0 --> 1

Char. 343: 0 --> 1

Char. 416: 0 --> 1

Char. 441: 0 --> 1

Char. 797: 0 --> 1

Char. 828: 0 --> 1

Char. 876: 1 --> 0

Staurikosaurus_pricei :

Char. 381: 0 --> 1

Char. 385: 0 --> 1

Char. 819: 0 --> 1

Char. 883: 1 --> 0

Gobivenator :

Char. 58: 2 --> 0

Char. 72: 0 --> 1

Char. 94: 0 --> 1

Char. 95: 1 --> 0

Char. 98: 1 --> 2

Char. 208: 1 --> 0

Char. 214: 1 --> 0

Char. 285: 1 --> 0

Char. 949: 1 --> 0

Gorgosaurus_libratus :

Char. 3: 1 --> 0

Char. 13: 1 --> 0

Char. 53: 1 --> 0

Char. 105: 0 --> 1

Char. 128: 1 --> 0

Char. 197: 0 --> 1

Char. 234: 1 --> 0

Char. 381: 1 --> 0

Char. 400: 1 --> 2

Char. 629: 0 --> 1

Char. 764: 1 --> 2

Char. 782: 0 --> 1

Char. 954: 1 --> 0

Gualicho_shinyae :

Char. 631: 0 --> 1

Char. 649: 0 --> 1

Char. 650: 1 --> 0

Char. 652: 1 --> 0

Char. 655: 1 --> 0

Char. 666: 1 --> 2

Char. 673: 1 --> 0

Char. 686: 0 --> 1

Char. 692: 2 --> 3

Char. 694: 0 --> 1

Char. 703: 0 --> 1

Char. 714: 1 --> 0

Char. 718: 1 --> 2

Char. 719: 1 --> 0

Char. 723: 1 --> 0

Char. 738: 1 --> 0

Char. 745: 0 --> 1

Char. 747: 1 --> 0

Char. 806: 1 --> 0

Char. 808: 2 --> 0

Char. 839: 1 --> 2

Char. 851: 1 --> 0

Char. 867: 0 --> 1

Char. 870: 0 --> 1

Char. 873: 0 --> 1

Char. 879: 1 --> 0

Char. 882: 1 --> 0

Char. 888: 0 --> 1

Char. 900: 1 --> 0

Char. 903: 1 --> 0

Char. 937: 0 --> 1

Char. 938: 1 --> 0

Char. 953: 1 --> 0

Guanlong_wucaii :

Char. 1: 1 --> 2

Char. 278: 1 --> 0

Char. 400: 1 --> 2

Char. 425: 0 --> 1

Haplocheirus_sollers :

Char. 6: 0 --> 1

Char. 10: 1 --> 0

Char. 11: 1 --> 0

Char. 19: 0 --> 1

Char. 21: 0 --> 1

Char. 56: 1 --> 0

Char. 94: 0 --> 1

Char. 95: 1 --> 0

Char. 124: 1 --> 0

Char. 164: 0 --> 1

Char. 187: 0 --> 1

Char. 192: 0 --> 1

Char. 195: 0 --> 1

Char. 206: 1 --> 0

Char. 327: 1 --> 0

Char. 368: 0 --> 1

Char. 423: 12 --> 3

Char. 427: 2 --> 1

Char. 430: 0 --> 1

Char. 484: 1 --> 0

Char. 532: 0 --> 1

Char. 632: 0 --> 1

Char. 670: 0 --> 1

Char. 682: 0 --> 1

Char. 707: 1 --> 0

Char. 784: 1 --> 0

Char. 803: 1 --> 0

Char. 808: 2 --> 0

Char. 826: 0 --> 1

Char. 902: 1 --> 0

Harpymimus_okladnikovi :

Char. 199: 0 --> 1

Char. 201: 1 --> 0

Char. 210: 1 --> 0

Char. 334: 0 --> 1

Char. 382: 2 --> 1

Char. 486: 0 --> 1

Char. 493: 0 --> 1

Char. 503: 1 --> 0

Char. 546: 0 --> 1

Char. 555: 0 --> 1

Char. 562: 0 --> 1

Char. 592: 0 --> 2

Char. 648: 0 --> 1

Char. 657: 1 --> 0

Char. 664: 0 --> 1

Char. 667: 0 --> 1

Char. 690: 0 --> 1

Char. 721: 0 --> 1

Char. 726: 1 --> 2

Char. 741: 0 --> 1

Char. 746: 1 --> 0

Char. 758: 1 --> 0

Char. 762: 1 --> 0

Char. 788: 1 --> 0

Char. 901: 0 --> 1

Char. 913: 1 --> 0

Char. 914: 3 --> 2

Char. 947: 1 --> 2

Ichthyovenator_laosensis :

Char. 467: 1 --> 0

Char. 494: 1 --> 0

Char. 495: 0 --> 1

Char. 506: 1 --> 2

Char. 526: 1 --> 0

Char. 564: 1 --> 0

Char. 592: 2 --> 0

Char. 604: 0 --> 1

Char. 609: 0 --> 1

Char. 775: 1 --> 0

Char. 780: 0 --> 1

Char. 800: 2 --> 3

Char. 807: 0 --> 1

Char. 821: 1 --> 0

Char. 826: 1 --> 0

Incisivosaurus_gauthieri :

Char. 3: 1 --> 0

Char. 10: 1 --> 0

Char. 14: 1 --> 0

Char. 52: 1 --> 0

Char. 69: 0 --> 2

Char. 112: 0 --> 1

Char. 116: 1 --> 0

Char. 118: 1 --> 0

Char. 125: 0 --> 1

Char. 128: 0 --> 1

Char. 133: 0 --> 1

Char. 140: 1 --> 0

Char. 197: 0 --> 1

Char. 224: 0 --> 1

Char. 234: 1 --> 0

Char. 248: 2 --> 0

Char. 253: 1 --> 0

Char. 257: 0 --> 1

Char. 259: 0 --> 1

Char. 265: 0 --> 1

Char. 267: 1 --> 0

Char. 269: 0 --> 1

Char. 288: 1 --> 0

Char. 343: 1 --> 0

Char. 354: 1 --> 0

Char. 394: 1 --> 0

Char. 408: 1 --> 0

Char. 433: 1 --> 0

Irritator_challengeri :

Char. 80: 0 --> 1

Char. 97: 1 --> 0

Char. 108: 1 --> 0

Char. 113: 1 --> 0

Char. 180: 1 --> 0

Char. 187: 1 --> 0

Char. 247: 0 --> 1

Char. 254: 1 --> 0

Char. 290: 12 --> 0

Char. 420: 2 --> 1

Jianchangosaurus_yixianensis :

Char. 343: 1 --> 0

Char. 375: 0 --> 1

Char. 391: 2 --> 0

Char. 464: 1 --> 0

Char. 487: 1 --> 0

Char. 694: 0 --> 1

Char. 747: 0 --> 1

Char. 755: 1 --> 23

Char. 758: 1 --> 0

Char. 763: 1 --> 0

Char. 766: 1 --> 0

Char. 830: 0 --> 23

Char. 852: 2 --> 1

Char. 927: 1 --> 0

Juratyrant_langhami :

Char. 882: 0 --> 1

Char. 886: 1 --> 0

Khaan_mckennai :

Char. 95: 1 --> 2

Char. 155: 1 --> 2

Char. 164: 1 --> 0

Char. 246: 1 --> 0

Char. 694: 0 --> 1

Char. 783: 0 --> 1

Char. 955: 2 --> 1

Kileskus_aristotocus :

Char. 15: 1 --> 0

Char. 53: 0 --> 1

Char. 65: 1 --> 2

Char. 70: 0 --> 1

Char. 409: 1 --> 2

Char. 420: 1 --> 0

Char. 529: 0 --> 1

Kiyacursor_longipes :

Char. 481: 1 --> 0

Char. 486: 0 --> 1

Char. 511: 1 --> 0

Char. 642: 1 --> 0

Char. 652: 1 --> 0

Char. 655: 1 --> 0

Char. 659: 0 --> 1

Char. 839: 0 --> 1

Char. 840: 0 --> 1

Char. 845: 1 --> 0

Char. 875: 0 --> 1

Char. 877: 1 --> 0

Char. 886: 0 --> 2

Char. 912: 1 --> 0

Char. 926: 0 --> 1

Char. 928: 0 --> 1

Char. 941: 0 --> 1

Char. 957: 1 --> 0

Lajasvenator_ascheriae :

Char. 84: 0 --> 1

Char. 86: 2 --> 1

Char. 429: 1 --> 0

Char. 535: 0 --> 1

Char. 536: 2 --> 1

Char. 753: 0 --> 1

Leshansaurus_qianweiensis :

Char. 54: 0 --> 1

Char. 75: 0 --> 2

Char. 90: 0 --> 1

Char. 212: 0 --> 1

Char. 224: 0 --> 1

Char. 250: 1 --> 0

Char. 254: 1 --> 0

Char. 273: 0 --> 1

Char. 467: 1 --> 0

Char. 563: 0 --> 1

Char. 578: 0 --> 2

Char. 788: 0 --> 1

Char. 840: 1 --> 0

Liliensternus_liliensterni :

Char. 128: 0 --> 1

Char. 133: 0 --> 1

Char. 356: 0 --> 1

Char. 416: 0 --> 1

Char. 635: 0 --> 1

Char. 641: 0 --> 1

Char. 648: 0 --> 1

Char. 659: 0 --> 1

Char. 716: 0 --> 1

Char. 831: 0 --> 1

Notatesseraeraptor_frickensis :

Char. 75: 2 --> 1

Char. 238: 0 --> 1

Char. 252: 0 --> 1

Char. 357: 1 --> 0

Char. 366: 1 --> 0

Char. 578: 0 --> 1

Char. 683: 1 --> 0

Char. 727: 0 --> 1

Char. 777: 0 --> 1

Char. 803: 1 --> 0

Char. 832: 0 --> 1

Panguraptor_lufengensis :

Char. 10: 0 --> 1

Char. 11: 0 --> 1

Char. 44: 1 --> 0

Char. 322: 1 --> 0

Char. 487: 0 --> 1

Char. 554: 0 --> 1

Char. 655: 1 --> 0

Char. 693: 1 --> 2

Char. 848: 2 --> 1

Char. 900: 1 --> 2

Char. 908: 1 --> 0

Limusaurus_inextricabilis :

Char. 494: 1 --> 0

Char. 570: 0 --> 1

Char. 614: 0 --> 2

Char. 667: 1 --> 0

Char. 690: 0 --> 1

Char. 763: 1 --> 0

Char. 795: 0 --> 1

Char. 823: 0 --> 1

Char. 861: 1 --> 0

Char. 876: 1 --> 0

Char. 877: 1 --> 0

Char. 886: 0 --> 2

Char. 902: 1 --> 0

Linheraptor_exquisitus :

Char. 11: 1 --> 0

Char. 18: 0 --> 1

Char. 23: 1 --> 0

Char. 39: 0 --> 1

Char. 44: 0 --> 1

Char. 97: 1 --> 0

Char. 128: 0 --> 1

Char. 135: 1 --> 2

Char. 145: 1 --> 0

Char. 167: 0 --> 1

Char. 198: 2 --> 0

Char. 232: 1 --> 0

Char. 247: 0 --> 1

Char. 254: 0 --> 1

Char. 281: 2 --> 1

Char. 357: 0 --> 1

Char. 411: 1 --> 2

Char. 418: 0 --> 1

Char. 532: 0 --> 1

Char. 546: 0 --> 1

Char. 560: 1 --> 0

Char. 588: 1 --> 0

Char. 621: 0 --> 1

Char. 625: 0 --> 1

Char. 686: 0 --> 1

Char. 804: 0 --> 1

Char. 940: 1 --> 0

Lourinhanosaurus_antunesi :

Char. 799: 0 --> 1

Char. 854: 1 --> 2

Char. 856: 0 --> 1

Char. 874: 0 --> 1

Char. 877: 1 --> 0

Char. 881: 1 --> 0

Lucianovenator_bonoi :

Char. 504: 0 --> 1

Char. 781: 1 --> 0

Lusovenator_santosi :

Char. 801: 0 --> 1

Maip_macrothorax :

Char. 603: 0 --> 1

Char. 605: 1 --> 0

Majungasaurus_crenatissimus :

Char. 8: 0 --> 1

Char. 15: 1 --> 2

Char. 142: 0 --> 1

Char. 144: 1 --> 0

Char. 160: 1 --> 0

Char. 171: 0 --> 1

Char. 176: 0 --> 1

Char. 178: 1 --> 0

Char. 184: 0 --> 1

Char. 195: 0 --> 1

Char. 202: 0 --> 1

Char. 238: 0 --> 2

Char. 262: 0 --> 1

Char. 290: 2 --> 1

Char. 318: 0 --> 1

Char. 343: 0 --> 1

Char. 373: 1 --> 0

Char. 462: 0 --> 1

Char. 475: 2 --> 0

Char. 486: 0 --> 1

Char. 543: 0 --> 1

Char. 560: 0 --> 1

Char. 562: 1 --> 0

Char. 574: 3 --> 2

Char. 605: 1 --> 0

Char. 636: 1 --> 0

Char. 641: 0 --> 1

Char. 762: 1 --> 0

Char. 868: 0 --> 1

Char. 876: 1 --> 2

Char. 937: 2 --> 0

Char. 944: 0 --> 1

Char. 949: 1 --> 0

Char. 951: 0 --> 1

Mapusaurus_roseae :

Char. 474: 1 --> 0

Char. 478: 1 --> 0

Char. 827: 0 --> 1

Char. 830: 0 --> 1

Marshosaurus_bicentesimus :

Char. 54: 0 --> 1

Char. 481: 1 --> 2

Char. 544: 0 --> 1

Char. 558: 0 --> 1

Char. 781: 0 --> 1

Char. 803: 1 --> 0

Char. 810: 0 --> 1

Masiakasaurus_knopfleri :

Char. 41: 0 --> 1

Char. 70: 0 --> 1

Char. 84: 1 --> 0

Char. 112: 1 --> 0

Char. 115: 0 --> 1

Char. 139: 0 --> 1

Char. 141: 0 --> 1

Char. 327: 1 --> 0

Char. 430: 0 --> 1

Char. 457: 0 --> 1

Char. 505: 1 --> 2

Char. 512: 0 --> 1

Char. 513: 2 --> 1

Char. 517: 1 --> 0

Char. 570: 0 --> 1

Char. 609: 2 --> 3

Char. 657: 0 --> 1

Char. 667: 1 --> 0

Char. 763: 1 --> 0

Char. 795: 0 --> 1

Char. 801: 1 --> 0

Char. 819: 1 --> 0

Char. 848: 2 --> 1

Char. 854: 0 --> 1

Char. 858: 1 --> 0

Char. 868: 0 --> 1

Char. 888: 1 --> 0

Char. 909: 1 --> 0

Char. 943: 0 --> 1

Char. 944: 0 --> 1

Megalosaurus :

Char. 40: 0 --> 1

Char. 45: 2 --> 0

Char. 81: 0 --> 1

Char. 429: 0 --> 1

Char. 575: 0 --> 1

Char. 610: 0 --> 1

Char. 669: 0 --> 1

Char. 670: 0 --> 1

Char. 774: 0 --> 1

Char. 819: 0 --> 1

Char. 835: 1 --> 0

Char. 840: 1 --> 0

Char. 873: 1 --> 0

Char. 886: 2 --> 0

Char. 889: 0 --> 1

Megaraptor_namunhuaiquii :

Char. 592: 0 --> 2

Megaraptorid_mucpv_595 :

Char. 224: 0 --> 1

Char. 226: 1 --> 2

Char. 241: 1 --> 0

Char. 242: 1 --> 0

Char. 255: 0 --> 1

Char. 256: 0 --> 1

Char. 290: 1 --> 0

Char. 293: 0 --> 1

Char. 425: 1 --> 2

Char. 453: 1 --> 0

Char. 461: 1 --> 0

Char. 520: 1 --> 0

Char. 555: 1 --> 0

Char. 650: 1 --> 0

Mei_long :

Char. 0: 1 --> 0

Char. 3: 1 --> 0

Char. 15: 2 --> 3

Char. 16: 1 --> 0

Char. 23: 2 --> 1

Char. 32: 0 --> 1

Char. 45: 2 --> 3

Char. 49: 0 --> 1

Char. 95: 1 --> 2

Char. 101: 1 --> 0

Char. 108: 1 --> 0

Char. 198: 0 --> 2

Char. 215: 2 --> 0

Char. 327: 1 --> 0

Char. 334: 0 --> 1

Char. 343: 1 --> 0

Char. 347: 0 --> 1

Char. 388: 1 --> 0

Char. 391: 2 --> 1

Char. 398: 1 --> 0

Char. 457: 1 --> 0

Char. 467: 1 --> 0

Char. 469: 0 --> 1

Char. 503: 1 --> 0

Char. 535: 0 --> 2

Char. 553: 0 --> 1

Char. 592: 2 --> 0

Char. 613: 2 --> 0

Char. 614: 1 --> 2

Char. 615: 1 --> 0

Char. 630: 0 --> 1

Char. 635: 1 --> 0

Char. 667: 1 --> 0

Char. 741: 1 --> 0

Char. 753: 1 --> 0

Char. 756: 0 --> 1

Char. 777: 1 --> 0

Char. 787: 1 --> 0

Char. 803: 1 --> 0

Char. 836: 1 --> 0

Char. 912: 0 --> 1

Char. 936: 0 --> 1

Meraxes_gigas :

Char. 127: 2 --> 3

Char. 133: 0 --> 1

Char. 134: 0 --> 1

Char. 317: 0 --> 1

Char. 671: 0 --> 1

Char. 840: 1 --> 0

Char. 960: 1 --> 2

Metriacanthosaurus_parkeri :

Char. 558: 0 --> 1

Char. 791: 0 --> 1

Char. 873: 0 --> 1

Char. 889: 0 --> 1

Microraptor :

Char. 322: 1 --> 2

Char. 401: 0 --> 1

Char. 428: 1 --> 0

Char. 540: 1 --> 0

Char. 631: 0 --> 1

Char. 721: 1 --> 0

Char. 741: 1 --> 0

Char. 774: 0 --> 1

Char. 787: 1 --> 0

Char. 803: 1 --> 0

Char. 820: 1 --> 2

Microvenator_celer :

Char. 329: 1 --> 0

Char. 354: 1 --> 0

Char. 667: 1 --> 0

Char. 850: 0 --> 1

Mirischia_asymmetrica :

Char. 535: 0 --> 2

Char. 554: 1 --> 0

Char. 557: 0 --> 1

Char. 561: 0 --> 1

Char. 567: 0 --> 1

Char. 575: 0 --> 12

Char. 760: 0 --> 1

Char. 761: 0 --> 2

Char. 778: 0 --> 1

Char. 780: 0 --> 1

Char. 782: 0 --> 1

Char. 793: 1 --> 0

Char. 795: 0 --> 1

Char. 796: 1 --> 0

Char. 797: 1 --> 0

Char. 802: 0 --> 1

Char. 810: 0 --> 1

Char. 813: 0 --> 1

Char. 819: 1 --> 0

Char. 820: 0 --> 1

Char. 834: 0 --> 1

Char. 840: 1 --> 0

Char. 848: 0 --> 1

Char. 854: 1 --> 2

Char. 858: 0 --> 1

Char. 862: 0 --> 1

Char. 876: 1 --> 2

Monolophosaurus_jiangi :

Char. 15: 2 --> 1

Char. 25: 0 --> 1

Char. 40: 0 --> 1

Char. 88: 0 --> 1

Char. 96: 1 --> 0

Char. 104: 1 --> 0

Char. 115: 0 --> 1

Char. 129: 0 --> 1

Char. 153: 1 --> 0

Char. 173: 1 --> 0

Char. 178: 1 --> 2

Char. 180: 1 --> 0

Char. 194: 0 --> 1

Char. 195: 0 --> 1

Char. 222: 0 --> 1

Char. 231: 1 --> 0

Char. 238: 0 --> 2

Char. 247: 0 --> 1

Char. 342: 0 --> 1

Char. 399: 1 --> 0

Char. 404: 0 --> 1

Char. 454: 0 --> 1

Char. 479: 2 --> 3

Char. 539: 0 --> 1

Char. 760: 0 --> 1

Char. 781: 0 --> 1

Char. 806: 0 --> 1

Mononykus_olecranus :

Char. 285: 1 --> 2

Char. 484: 1 --> 0

Char. 543: 0 --> 1

Char. 560: 0 --> 1

Char. 688: 1 --> 0

Char. 952: 0 --> 1

Char. 953: 0 --> 1

Char. 955: 1 --> 2

Murusraptor_barrosaensis :

Char. 139: 0 --> 1

Char. 141: 1 --> 0

Char. 223: 0 --> 1

Char. 284: 0 --> 1

Char. 302: 0 --> 1

Char. 549: 1 --> 0

Char. 552: 0 --> 1

Char. 603: 0 --> 1

Char. 748: 0 --> 1

Char. 790: 0 --> 1

Char. 796: 1 --> 0

Neovenator_salerii :

Char. 4: 0 --> 1

Char. 16: 2 --> 1

Char. 20: 1 --> 0

Char. 33: 1 --> 2

Char. 34: 1 --> 0

Char. 63: 1 --> 0

Char. 76: 1 --> 2

Char. 85: 0 --> 1

Char. 188: 1 --> 0

Char. 192: 0 --> 1

Char. 395: 1 --> 2

Char. 470: 0 --> 1

Char. 479: 3 --> 2

Char. 498: 0 --> 1

Char. 534: 0 --> 1

Char. 549: 1 --> 0

Char. 552: 1 --> 0

Char. 560: 0 --> 1

Char. 598: 1 --> 0

Char. 606: 1 --> 0

Char. 806: 0 --> 1

Char. 819: 1 --> 0

Char. 827: 0 --> 1

Char. 835: 1 --> 0

Char. 858: 1 --> 0

Char. 876: 1 --> 2

Char. 882: 0 --> 1

Char. 887: 0 --> 1

Char. 900: 1 --> 2

Char. 938: 1 --> 0

Char. 953: 0 --> 1

Noasaurus_leali :

Char. 58: 1 --> 0

Char. 67: 1 --> 0

Char. 69: 2 --> 1

Char. 498: 1 --> 2

Char. 505: 1 --> 2

Char. 517: 1 --> 2

Char. 519: 0 --> 1

Nomingia_gobiensis :

Char. 562: 0 --> 1

Char. 575: 0 --> 2

Char. 585: 3 --> 2

Char. 617: 0 --> 1

Char. 759: 1 --> 2

Char. 761: 0 --> 1

Char. 772: 1 --> 0

Char. 803: 1 --> 2

Char. 811: 0 --> 1

Char. 848: 0 --> 1

Char. 855: 2 --> 1

Char. 905: 0 --> 1

Char. 913: 1 --> 0

Char. 919: 0 --> 1

Char. 922: 1 --> 0

Nothronychus :

Char. 641: 1 --> 0

Char. 646: 1 --> 0

Char. 759: 1 --> 2

Char. 770: 0 --> 1

Char. 772: 0 --> 1

Char. 825: 1 --> 2

Char. 897: 2 --> 3

Char. 901: 1 --> 2

Nqwebasaurus_thwazi :

Char. 49: 0 --> 1

Char. 63: 1 --> 0

Char. 79: 0 --> 1

Char. 104: 0 --> 1

Char. 121: 0 --> 1

Char. 201: 1 --> 0

Char. 203: 0 --> 1

Char. 422: 2 --> 1

Char. 502: 1 --> 0

Char. 548: 0 --> 2

Char. 693: 2 --> 1

Char. 703: 0 --> 1

Char. 707: 1 --> 0

Char. 719: 1 --> 0

Char. 746: 1 --> 0

Char. 847: 0 --> 1

Char. 873: 0 --> 1

Char. 902: 1 --> 0

Char. 913: 1 --> 0

Char. 917: 0 --> 1

Char. 954: 1 --> 2

Ornitholestes_hermanni :

Char. 80: 0 --> 1

Char. 88: 0 --> 1

Char. 127: 1 --> 0

Char. 133: 0 --> 1

Char. 215: 0 --> 2

Char. 230: 1 --> 0

Char. 246: 0 --> 1

Char. 329: 0 --> 1

Char. 331: 0 --> 1

Char. 360: 1 --> 0

Char. 397: 0 --> 1

Char. 398: 1 --> 2

Char. 399: 1 --> 2

Char. 412: 0 --> 1

Char. 418: 0 --> 1

Char. 512: 0 --> 1

Char. 554: 1 --> 0

Char. 609: 0 --> 3

Char. 657: 0 --> 1

Char. 772: 0 --> 1

Char. 818: 0 --> 1

Char. 870: 0 --> 1

Char. 874: 0 --> 1

Char. 902: 1 --> 0

Char. 914: 3 --> 2

Char. 932: 0 --> 1

Ornithomimus :

Char. 3: 0 --> 1

Char. 65: 01 --> 2

Char. 127: 1 --> 0

Char. 201: 1 --> 0

Char. 224: 0 --> 1

Char. 227: 0 --> 1

Char. 235: 0 --> 1

Char. 263: 1 --> 0

Char. 331: 0 --> 1

Char. 380: 0 --> 1

Char. 544: 0 --> 1

Char. 557: 0 --> 1

Char. 641: 1 --> 0

Char. 747: 1 --> 2

Char. 749: 0 --> 1

Char. 750: 0 --> 1

Char. 752: 0 --> 1

Char. 911: 0 --> 1

Char. 947: 1 --> 2

Oviraptor_philoceratops :

No autapomorphies

Patagonykus_puertai :

Char. 563: 0 --> 1

Char. 566: 0 --> 1

Char. 575: 0 --> 2

Char. 592: 1 --> 0

Char. 614: 1 --> 2

Char. 632: 0 --> 1

Char. 672: 1 --> 0

Char. 703: 0 --> 1

Char. 738: 1 --> 0

Char. 741: 0 --> 1

Char. 784: 1 --> 0

Char. 870: 0 --> 1

Char. 873: 0 --> 1

Char. 913: 1 --> 0

Char. 921: 1 --> 0

Char. 925: 0 --> 1

Char. 933: 0 --> 1

Pelecanimimus_polyodon :

Char. 19: 0 --> 1

Char. 22: 1 --> 0

Char. 30: 0 --> 1

Char. 33: 0 --> 1

Char. 52: 0 --> 1

Char. 53: 1 --> 0

Char. 91: 1 --> 0

Char. 114: 0 --> 1

Char. 125: 0 --> 1

Char. 126: 0 --> 1

Char. 127: 1 --> 0

Char. 165: 0 --> 1

Char. 186: 0 --> 1

Char. 251: 0 --> 1

Char. 275: 1 --> 0

Char. 328: 1 --> 0

Char. 330: 2 --> 0

Char. 394: 1 --> 0

Char. 408: 1 --> 0

Char. 434: 1 --> 3

Char. 435: 0 --> 1

Char. 451: 1 --> 0

Char. 506: 0 --> 1

Char. 532: 0 --> 1

Char. 536: 0 --> 1

Char. 557: 0 --> 1

Char. 571: 0 --> 1

Char. 637: 1 --> 0

Char. 642: 1 --> 0

Char. 672: 2 --> 1

Char. 690: 0 --> 1

Char. 714: 1 --> 0

Piatnitzkysaurus_floresi :

Char. 41: 0 --> 1

Char. 63: 1 --> 0

Char. 201: 0 --> 1

Char. 254: 1 --> 0

Char. 255: 1 --> 0

Char. 334: 0 --> 1

Char. 417: 0 --> 1

Char. 506: 1 --> 2

Char. 526: 1 --> 2

Char. 797: 1 --> 0

Char. 862: 1 --> 0

Char. 889: 0 --> 1

Proceratosaurus_bradleyi :

Char. 3: 1 --> 2

Char. 10: 0 --> 1

Char. 15: 1 --> 0

Char. 18: 1 --> 0

Char. 28: 0 --> 1

Char. 65: 1 --> 0

Char. 88: 0 --> 1

Char. 265: 0 --> 1

Char. 271: 0 --> 1

Char. 327: 0 --> 1

Char. 409: 1 --> 3

Char. 412: 0 --> 1

Rahonavis_ostromi :

Char. 327: 1 --> 0

Char. 331: 0 --> 1

Char. 535: 0 --> 2

Char. 536: 0 --> 2

Char. 548: 2 --> 0

Char. 553: 0 --> 1

Char. 554: 1 --> 0

Char. 562: 0 --> 1

Char. 575: 0 --> 1

Char. 578: 1 --> 0

Char. 593: 0 --> 1

Char. 655: 1 --> 2

Char. 686: 0 --> 1

Char. 755: 2 --> 3

Char. 759: 1 --> 2

Char. 763: 1 --> 0

Char. 771: 0 --> 1

Char. 774: 0 --> 1

Char. 783: 0 --> 1

Char. 787: 1 --> 0

Char. 796: 2 --> 1

Char. 806: 1 --> 0

Char. 807: 0 --> 1

Char. 819: 01 --> 2

Char. 820: 0 --> 1

Char. 824: 0 --> 1

Char. 835: 2 --> 1

Char. 836: 1 --> 2

Char. 853: 1 --> 2

Char. 874: 0 --> 1

Char. 886: 2 --> 1

Char. 905: 0 --> 1

Char. 927: 0 --> 1

Char. 934: 0 --> 1

Char. 935: 0 --> 1

Char. 941: 0 --> 1

Char. 942: 1 --> 0

Char. 948: 0 --> 1

Char. 963: 0 --> 1

Rajasaurus_narmadensis :

Char. 136: 0 --> 1

Char. 202: 0 --> 1

Rinchenia_mongoliensis :

Char. 333: 0 --> 1

Char. 759: 1 --> 2

Char. 762: 0 --> 1

Char. 766: 0 --> 1

Char. 783: 0 --> 1

Char. 839: 1 --> 2

Rugops_primus :

Char. 23: 1 --> 0

Char. 31: 0 --> 1

Char. 47: 1 --> 0

Char. 84: 1 --> 0

Char. 121: 1 --> 2

Char. 186: 1 --> 0

Char. 187: 2 --> 0

Char. 191: 0 --> 1

Char. 192: 0 --> 1

Santanaraptor_placidus :

Char. 606: 0 --> 1

Char. 609: 0 --> 4

Char. 825: 0 --> 1

Char. 867: 0 --> 1

Char. 873: 0 --> 1

Char. 882: 0 --> 1

Char. 892: 1 --> 0

Char. 919: 1 --> 0

Sapeornis_chaoyangensis :

Char. 34: 0 --> 1

Char. 111: 1 --> 0

Char. 128: 0 --> 1

Char. 140: 1 --> 0

Char. 203: 0 --> 1

Char. 214: 1 --> 0

Char. 320: 0 --> 1

Char. 328: 0 --> 1

Char. 363: 0 --> 1

Char. 591: 1 --> 0

Char. 630: 0 --> 1

Char. 787: 1 --> 0

Char. 821: 0 --> 1

Char. 824: 0 --> 1

Char. 946: 2 --> 1

Char. 959: 1 --> 2

Saurornithoides_mongoliensis :

Char. 1: 1 --> 2

Char. 3: 1 --> 0

Char. 15: 2 --> 1

Char. 16: 1 --> 2

Char. 19: 0 --> 1

Char. 52: 0 --> 1

Char. 58: 2 --> 0

Char. 63: 1 --> 0

Char. 70: 0 --> 1

Char. 80: 1 --> 0

Char. 81: 0 --> 1

Char. 97: 0 --> 1

Char. 99: 1 --> 0

Char. 280: 0 --> 1

Char. 289: 0 --> 1

Char. 291: 1 --> 0

Char. 292: 0 --> 2

Char. 304: 1 --> 0

Char. 327: 1 --> 2

Char. 337: 0 --> 1

Char. 343: 1 --> 0

Char. 347: 0 --> 1

Char. 427: 2 --> 0

Char. 434: 1 --> 3

Char. 435: 0 --> 1

Char. 817: 0 --> 2

Char. 821: 0 --> 1

Char. 825: 2 --> 1

Char. 836: 1 --> 0

Char. 838: 0 --> 1

Char. 855: 2 --> 1

Char. 936: 0 --> 1

Char. 949: 1 --> 0

Saurornitholestes :

Char. 1: 1 --> 2

Char. 23: 1 --> 0

Char. 29: 0 --> 1

Char. 40: 0 --> 1

Char. 57: 0 --> 1

Char. 104: 0 --> 1

Char. 108: 1 --> 2

Char. 118: 1 --> 0

Char. 126: 0 --> 1

Char. 133: 0 --> 1

Char. 135: 1 --> 2

Char. 145: 1 --> 0

Char. 175: 1 --> 0

Char. 192: 0 --> 1

Char. 194: 0 --> 1

Char. 195: 0 --> 1

Char. 196: 0 --> 1

Char. 333: 0 --> 1

Char. 335: 0 --> 1

Char. 363: 0 --> 2

Char. 405: 1 --> 0

Char. 407: 0 --> 1

Char. 425: 1 --> 0

Char. 503: 1 --> 0

Char. 575: 0 --> 1

Char. 620: 2 --> 1

Char. 658: 0 --> 1

Char. 681: 1 --> 0

Char. 686: 0 --> 1

Char. 759: 1 --> 2

Char. 773: 1 --> 0

Char. 780: 0 --> 1

Char. 783: 0 --> 1

Char. 787: 1 --> 0

Char. 906: 0 --> 1

Char. 922: 1 --> 0

Char. 938: 1 --> 0

Segnosaurus_galbinensis :

Char. 631: 0 --> 1

Char. 646: 1 --> 2

Char. 821: 0 --> 1

Char. 879: 1 --> 0

Char. 901: 1 --> 0

Char. 914: 3 --> 2

Shaochilong_maortuensis :

Char. 41: 0 --> 1

Char. 61: 2 --> 0

Char. 69: 12 --> 0

Char. 70: 0 --> 1

Char. 86: 1 --> 2

Char. 200: 0 --> 1

Char. 207: 0 --> 1

Char. 208: 0 --> 1

Char. 213: 0 --> 1

Char. 258: 2 --> 3

Char. 285: 1 --> 2

Char. 295: 1 --> 0

Char. 600: 0 --> 1

Shenzhousaurus_orientalis :

Char. 15: 12 --> 3

Char. 30: 0 --> 1

Char. 53: 1 --> 0

Char. 58: 12 --> 0

Char. 63: 1 --> 0

Char. 160: 0 --> 1

Char. 186: 0 --> 1

Char. 330: 2 --> 0

Char. 335: 0 --> 1

Char. 439: 0 --> 1

Char. 718: 1 --> 0

Char. 729: 1 --> 0

Char. 779: 0 --> 1

Char. 782: 0 --> 1

Char. 784: 1 --> 0

Char. 791: 1 --> 0

Char. 804: 0 --> 1

Char. 811: 1 --> 0

Char. 818: 0 --> 1

Shuvuuia_deserti :

Char. 503: 1 --> 0

Char. 513: 1 --> 0

Char. 526: 0 --> 1

Char. 592: 1 --> 2

Char. 886: 12 --> 0

Char. 954: 1 --> 0

Char. 955: 1 --> 0

Char. 956: 12 --> 0

Char. 959: 1 --> 0

Siamraptor_suwati :

Char. 22: 1 --> 0

Char. 85: 0 --> 1

Char. 127: 2 --> 3

Char. 402: 0 --> 2

Char. 512: 1 --> 0

Siats_meekerorum :

Char. 599: 1 --> 2

Char. 602: 0 --> 1

Sinocalliopteryx_gigas :

Char. 23: 1 --> 0

Char. 38: 0 --> 2

Char. 91: 1 --> 0

Char. 192: 0 --> 1

Char. 320: 0 --> 1

Char. 445: 0 --> 1

Char. 505: 0 --> 1

Char. 532: 1 --> 2

Char. 557: 0 --> 1

Char. 585: 3 --> 4

Char. 641: 1 --> 0

Char. 683: 1 --> 0

Char. 718: 1 --> 2

Char. 739: 1 --> 2

Char. 803: 1 --> 0

Char. 830: 0 --> 1

Char. 947: 1 --> 0

Char. 954: 1 --> 0

Sinornithomimus_dongi :

Char. 1: 2 --> 1

Char. 26: 0 --> 1

Char. 39: 0 --> 1

Char. 98: 1 --> 2

Char. 135: 0 --> 2

Char. 198: 0 --> 2

Char. 245: 1 --> 0

Char. 327: 1 --> 2

Char. 460: 1 --> 0

Char. 469: 0 --> 1

Char. 479: 0 --> 2

Char. 660: 0 --> 1

Char. 726: 1 --> 2

Char. 811: 1 --> 0

Char. 818: 0 --> 1

Sinornithoides_youngi :

Char. 404: 2 --> 0

Char. 418: 1 --> 0

Char. 427: 2 --> 0

Char. 435: 0 --> 1

Char. 487: 2 --> 1

Char. 613: 2 --> 0

Char. 635: 1 --> 0

Char. 650: 0 --> 1

Char. 667: 1 --> 0

Char. 672: 1 --> 2

Char. 753: 1 --> 0

Char. 939: 1 --> 0

Char. 949: 1 --> 0

Char. 958: 1 --> 0

Char. 960: 1 --> 2

Sinornithosaurus_millenii :

Char. 36: 0 --> 1

Char. 65: 1 --> 2

Char. 99: 1 --> 0

Char. 120: 1 --> 0

Char. 175: 1 --> 0

Char. 344: 0 --> 1

Char. 712: 0 --> 1

Char. 772: 1 --> 0

Char. 915: 0 --> 1

Sinosauropteryx_prima :

Char. 13: 1 --> 2

Char. 39: 0 --> 1

Char. 58: 2 --> 0

Char. 121: 0 --> 1

Char. 487: 2 --> 0

Char. 499: 0 --> 1

Char. 585: 3 --> 5

Char. 655: 1 --> 0

Char. 667: 1 --> 2

Char. 694: 0 --> 1

Char. 739: 1 --> 2

Char. 758: 1 --> 0

Char. 783: 1 --> 0

Char. 826: 0 --> 1

Char. 886: 2 --> 0

Sinovenator_changii :

Char. 13: 1 --> 2

Char. 17: 1 --> 0

Char. 34: 0 --> 1

Char. 97: 0 --> 1

Char. 98: 1 --> 2

Char. 104: 0 --> 1

Char. 108: 1 --> 0

Char. 127: 1 --> 0

Char. 130: 1 --> 0

Char. 230: 1 --> 0

Char. 262: 1 --> 0

Char. 277: 1 --> 0

Char. 278: 0 --> 1

Char. 337: 0 --> 1

Char. 368: 0 --> 1

Char. 381: 0 --> 1

Char. 393: 1 --> 0

Char. 427: 2 --> 0

Char. 435: 0 --> 1

Char. 458: 0 --> 1

Char. 461: 1 --> 0

Char. 467: 1 --> 0

Char. 540: 1 --> 0

Char. 580: 1 --> 0

Char. 613: 2 --> 0

Char. 614: 1 --> 2

Char. 620: 2 --> 1

Char. 641: 0 --> 1

Char. 753: 1 --> 2

Char. 820: 0 --> 1

Char. 824: 0 --> 1

Char. 880: 0 --> 1

Char. 911: 0 --> 1

Char. 941: 0 --> 1

Sinraptor_dongi :

Char. 3: 1 --> 0

Char. 9: 2 --> 3

Char. 63: 1 --> 0

Char. 116: 0 --> 1

Char. 135: 2 --> 1

Char. 157: 0 --> 1

Char. 402: 0 --> 1

Char. 451: 2 --> 1

Char. 540: 0 --> 1

Char. 558: 0 --> 1

Char. 564: 0 --> 1

Char. 719: 0 --> 1

Char. 762: 0 --> 1

Char. 775: 0 --> 1

Char. 791: 1 --> 0

Char. 806: 0 --> 1

Char. 819: 1 --> 0

Char. 821: 1 --> 0

Char. 840: 0 --> 1

Char. 867: 0 --> 1

Char. 873: 0 --> 1

Char. 884: 1 --> 2

Char. 908: 1 --> 0

Char. 914: 2 --> 1

Char. 915: 1 --> 2

Skorpiovenator_bustingorryi :

Char. 48: 0 --> 1

Char. 56: 0 --> 1

Char. 149: 1 --> 0

Char. 164: 0 --> 1

Char. 170: 0 --> 1

Char. 171: 0 --> 1

Char. 330: 0 --> 2

Char. 391: 1 --> 0

Char. 758: 1 --> 0

Char. 795: 1 --> 0

Spectrovenator_ragei :

Char. 110: 1 --> 0

Char. 115: 1 --> 0

Char. 120: 1 --> 0

Char. 150: 1 --> 0

Char. 161: 0 --> 1

Char. 166: 1 --> 0

Char. 173: 1 --> 0

Char. 200: 1 --> 0

Char. 213: 1 --> 0

Char. 215: 1 --> 0

Char. 263: 1 --> 0

Char. 338: 1 --> 0

Char. 343: 0 --> 1

Char. 347: 0 --> 1

Char. 348: 2 --> 1

Char. 381: 1 --> 0

Char. 797: 1 --> 0

Llukalkan_aliocranianus :

Char. 9: 23 --> 1

Char. 38: 1 --> 0

Char. 119: 1 --> 0

Char. 161: 0 --> 1

Char. 241: 1 --> 2

Char. 248: 1 --> 0

Char. 285: 0 --> 1

Char. 288: 0 --> 1

Char. 402: 0 --> 2

Spinosaurus_aegyptiacus :

Char. 330: 0 --> 1

Char. 333: 0 --> 1

Char. 340: 1 --> 0

Char. 350: 1 --> 2

Char. 411: 1 --> 2

Char. 438: 1 --> 2

Char. 488: 0 --> 1

Char. 498: 0 --> 1

Char. 506: 1 --> 2

Char. 513: 0 --> 1

Char. 544: 1 --> 0

Char. 547: 0 --> 1

Char. 549: 1 --> 2

Char. 556: 0 --> 1

Char. 564: 1 --> 0

Char. 569: 0 --> 1

Char. 586: 0 --> 1

Char. 592: 2 --> 0

Char. 603: 0 --> 1

MSNM_v4047 :

Char. 36: 0 --> 1

Char. 50: 0 --> 1

Char. 404: 0 --> 2

Char. 411: 1 --> 2

MNBH_TIG_6 :

Char. 494: 1 --> 0

Char. 506: 1 --> 2

Char. 573: 0 --> 1

Char. 584: 0 --> 1

Struthiomimus_altus :

Char. 15: 2 --> 1

Char. 19: 0 --> 1

Char. 59: 1 --> 0

Char. 95: 1 --> 2

Char. 186: 0 --> 1

Char. 232: 1 --> 0

Char. 244: 0 --> 1

Char. 253: 1 --> 0

Char. 258: 2 --> 3

Char. 262: 0 --> 1

Char. 291: 1 --> 0

Char. 295: 1 --> 2

Char. 333: 0 --> 1

Char. 467: 0 --> 1

Char. 510: 1 --> 0

Char. 549: 2 --> 1

Char. 613: 0 --> 1

Char. 806: 1 --> 0

Char. 808: 2 --> 0

Char. 811: 1 --> 0

Suchomimus_tenerensis :

Char. 34: 0 --> 1

Char. 229: 1 --> 0

Char. 409: 12 --> 3

Char. 425: 2 --> 1

Char. 505: 0 --> 1

Char. 514: 1 --> 0

Char. 515: 1 --> 0

Char. 531: 1 --> 0

Char. 548: 0 --> 1

Char. 658: 0 --> 1

Char. 823: 0 --> 1

Char. 854: 1 --> 2

"Szechuanosaurus"_zigongensis :

Char. 44: 1 --> 0

Char. 457: 0 --> 1

Char. 462: 0 --> 1

Char. 547: 0 --> 1

Char. 562: 1 --> 0

Char. 790: 1 --> 0

Char. 816: 1 --> 2

Char. 834: 0 --> 1

Tameryraptor_markgrafi :

Char. 605: 1 --> 0

Char. 819: 1 --> 0

Tanycolagreus :

Char. 15: 1 --> 2

Char. 19: 0 --> 1

Char. 147: 0 --> 1

Char. 172: 0 --> 1

Char. 541: 1 --> 0

Char. 552: 0 --> 1

Char. 567: 0 --> 1

Char. 618: 1 --> 0

Char. 631: 0 --> 1

Char. 648: 0 --> 1

Char. 661: 0 --> 2

Char. 673: 1 --> 0

Char. 676: 0 --> 1

Char. 689: 0 --> 1

Char. 710: 0 --> 1

Char. 720: 1 --> 0

Char. 729: 1 --> 0

Char. 744: 1 --> 0

Char. 746: 1 --> 0

Char. 813: 0 --> 1

Char. 851: 1 --> 0

Char. 870: 0 --> 1

Char. 884: 1 --> 2

Char. 897: 2 --> 3

Char. 901: 0 --> 1

Char. 903: 0 --> 1

Char. 908: 1 --> 0

Char. 922: 1 --> 0

Char. 930: 1 --> 0

Char. 947: 1 --> 0

Char. 954: 1 --> 0

Tarbosaurus_bataar :

Char. 0: 1 --> 2

Char. 16: 3 --> 2

Char. 35: 0 --> 1

Char. 53: 1 --> 0

Char. 67: 0 --> 1

Char. 110: 0 --> 3

Char. 119: 0 --> 1

Char. 160: 0 --> 1

Char. 178: 0 --> 1

Char. 199: 0 --> 1

Char. 224: 0 --> 1

Char. 292: 1 --> 0

Char. 305: 0 --> 1

Char. 368: 1 --> 0

Char. 600: 0 --> 1

Char. 741: 1 --> 0

Char. 747: 0 --> 1

Char. 949: 1 --> 0

Char. 952: 0 --> 1

Char. 959: 1 --> 0

MPCA_Pv_803 :

Char. 127: 2 --> 1

Char. 128: 1 --> 0

Char. 487: 0 --> 12

Char. 657: 1 --> 0

Char. 664: 2 --> 1

Char. 672: 1 --> 2

Char. 855: 0 --> 1

Char. 951: 1 --> 0

Torvosaurus_tanneri :

Char. 55: 0 --> 1

Char. 58: 2 --> 0

Char. 80: 0 --> 1

Char. 108: 1 --> 0

Char. 112: 1 --> 0

Char. 117: 0 --> 1

Char. 391: 2 --> 01

Char. 398: 0 --> 1

Char. 610: 0 --> 1

Troodon_formosus :

Char. 17: 1 --> 0

Char. 44: 1 --> 0

Char. 99: 1 --> 0

Char. 103: 0 --> 1

Char. 225: 1 --> 0

Char. 259: 1 --> 0

Char. 275: 0 --> 1

Char. 280: 0 --> 1

Char. 285: 1 --> 0

Char. 289: 0 --> 1

Char. 291: 1 --> 0

Char. 292: 0 --> 2

Char. 295: 0 --> 2

Char. 302: 1 --> 0

Char. 418: 1 --> 0

Char. 425: 2 --> 1

Char. 427: 2 --> 0

Char. 441: 0 --> 2

Char. 503: 1 --> 0

Char. 510: 1 --> 0

Char. 560: 0 --> 1

Char. 562: 0 --> 1

Char. 574: 3 --> 4

Char. 592: 2 --> 1

Char. 613: 2 --> 0

Char. 614: 1 --> 2

Char. 806: 1 --> 0

Char. 810: 1 --> 0

Char. 817: 0 --> 2

Char. 825: 2 --> 1

Char. 853: 1 --> 2

Char. 855: 2 --> 1

Char. 883: 1 --> 0

Char. 886: 2 --> 0

Char. 905: 0 --> 1

Char. 933: 0 --> 1

Char. 936: 0 --> 1

Char. 949: 1 --> 0

Char. 957: 0 --> 1

Char. 958: 1 --> 0

Char. 961: 1 --> 0

Tsaagan_mangas :

Char. 19: 1 --> 0

Char. 22: 1 --> 0

Char. 32: 0 --> 1

Char. 53: 1 --> 0

Char. 111: 1 --> 0

Char. 125: 0 --> 1

Char. 209: 1 --> 0

Char. 255: 0 --> 1

Char. 282: 1 --> 2

Char. 457: 1 --> 0

Tugulusaurus_faciles :

Char. 855: 1 --> 0

Char. 894: 1 --> 0

Char. 915: 0 --> 1

Tyrannosaurus_rex :

Char. 9: 1 --> 2

Char. 52: 0 --> 1

Char. 56: 1 --> 0

Char. 68: 0 --> 1

Char. 86: 1 --> 0

Char. 118: 0 --> 1

Char. 128: 1 --> 0

Char. 173: 1 --> 0

Char. 219: 1 --> 0

Char. 234: 1 --> 0

Char. 237: 0 --> 1

Char. 275: 0 --> 1

Char. 297: 0 --> 1

Char. 334: 0 --> 1

Char. 429: 0 --> 1

Char. 548: 1 --> 2

Char. 657: 0 --> 1

Char. 752: 0 --> 1

Char. 762: 0 --> 1

Char. 877: 0 --> 1

Tyrannotitan_chubutensis :

Char. 176: 1 --> 0

Char. 541: 1 --> 0

Char. 560: 0 --> 1

Char. 576: 0 --> 1

Char. 653: 1 --> 0

Char. 807: 0 --> 1

Char. 860: 1 --> 0

Char. 874: 0 --> 1

Velociraptor_mongoliensis :

Char. 108: 1 --> 2

Char. 126: 0 --> 1

Char. 164: 0 --> 1

Char. 244: 0 --> 1

Char. 257: 0 --> 1

Char. 291: 1 --> 0

Char. 509: 0 --> 1

Char. 624: 1 --> 0

Velocisaurus_unicus :

Char. 943: 0 --> 1

Vespersaurus_paranaensis :

Char. 210: 0 --> 1

Char. 486: 0 --> 1

Char. 491: 1 --> 0

Char. 502: 1 --> 0

Char. 511: 1 --> 0

Char. 513: 2 --> 3

Char. 517: 1 --> 0

Char. 609: 2 --> 0

Char. 654: 0 --> 1

Char. 670: 0 --> 1

Char. 674: 1 --> 0

Char. 793: 1 --> 0

Char. 820: 0 --> 1

Char. 940: 0 --> 1

Char. 941: 0 --> 1

Wiehenvenator_albati :

Char. 15: 2 --> 1

Char. 17: 1 --> 0

Char. 19: 1 --> 0

Char. 45: 2 --> 1

Char. 51: 0 --> 1

Char. 81: 0 --> 1

Char. 93: 1 --> 0

Char. 110: 0 --> 1

Char. 155: 1 --> 2

Char. 918: 0 --> 1

Char. 919: 1 --> 0

Xuanhanosaurus_qilixiaensis :

Char. 539: 0 --> 1

Char. 540: 0 --> 1

Char. 542: 0 --> 1

Char. 638: 0 --> 1

Yangchuanosaurus_hepingensis :

Char. 10: 0 --> 1

Char. 121: 1 --> 2

Char. 128: 0 --> 1

Char. 198: 1 --> 0

Char. 220: 0 --> 1

Char. 235: 0 --> 1

Char. 350: 0 --> 1

Char. 382: 1 --> 2

Char. 574: 2 --> 3

Char. 605: 1 --> 0

Char. 637: 1 --> 0

Char. 759: 0 --> 1

Char. 848: 0 --> 1

Char. 856: 0 --> 1

Yangchuanosaurus_shangyouensis :

Char. 109: 1 --> 0

Char. 167: 1 --> 0

Char. 170: 0 --> 1

Char. 192: 0 --> 1

Char. 438: 1 --> 0

Char. 549: 1 --> 2

Char. 764: 1 --> 0

Char. 773: 0 --> 1

Char. 862: 1 --> 0

Char. 877: 1 --> 0

Char. 921: 1 --> 0

Yixianornis_grabaui :

Char. 330: 2 --> 0

Char. 536: 0 --> 2

Char. 580: 1 --> 0

Char. 623: 1 --> 0

Char. 628: 1 --> 0

Char. 696: 1 --> 0

Char. 759: 1 --> 0

Char. 761: 1 --> 0

Char. 763: 0 --> 1

Char. 836: 1 --> 0

Yuanmouraptor_jinshajiangensis :

Char. 460: 1 --> 2

Char. 478: 1 --> 2

Char. 480: 1 --> 0

Char. 483: 1 --> 0

Char. 492: 0 --> 1

Char. 505: 1 --> 0

Char. 513: 0 --> 12

Char. 520: 1 --> 0

Yunyangosaurus_puanensis :

Char. 488: 0 --> 1

Char. 495: 1 --> 0

Char. 543: 0 --> 1

Char. 544: 0 --> 1

Char. 548: 0 --> 2

Yutyrannus_huali :

Char. 1: 1 --> 2

Char. 3: 1 --> 2

Char. 39: 0 --> 1

Char. 44: 0 --> 1

Char. 52: 0 --> 1

Char. 65: 0 --> 2

Char. 71: 1 --> 0

Char. 72: 1 --> 0

Char. 96: 1 --> 3

Char. 103: 1 --> 0

Char. 107: 0 --> 1

Char. 110: 0 --> 2

Char. 148: 1 --> 0

Char. 194: 0 --> 1

Char. 351: 0 --> 1

Char. 465: 1 --> 0

Char. 536: 1 --> 0

Char. 657: 0 --> 1

Char. 954: 1 --> 0

Zanabazar_junior :

Char. 16: 1 --> 3

Char. 19: 0 --> 1

Char. 23: 2 --> 1

Char. 33: 1 --> 2

Char. 58: 2 --> 0

Char. 63: 1 --> 0

Char. 70: 0 --> 1

Char. 94: 0 --> 1

Char. 95: 1 --> 0

Char. 97: 0 --> 1

Char. 103: 0 --> 1

Char. 155: 1 --> 2

Char. 212: 0 --> 1

Char. 225: 1 --> 0

Char. 231: 0 --> 1

Char. 255: 0 --> 1

Char. 263: 1 --> 0

Char. 275: 0 --> 1

Char. 280: 0 --> 1

Char. 289: 0 --> 1

Char. 292: 0 --> 2

Char. 298: 0 --> 1

Char. 302: 1 --> 0

Char. 337: 0 --> 1

Char. 347: 0 --> 1

Char. 402: 0 --> 2

Char. 423: 2 --> 3

Char. 427: 2 --> 0

Char. 434: 1 --> 3

Char. 435: 0 --> 1

Char. 613: 2 --> 0

Char. 614: 1 --> 2

Zuolong_salleei :

Char. 15: 1 --> 2

Char. 19: 0 --> 1

Char. 21: 1 --> 0

Char. 22: 1 --> 0

Char. 58: 2 --> 1

Char. 108: 1 --> 2

Char. 116: 0 --> 1

Char. 198: 0 --> 2

Char. 213: 0 --> 1

Char. 215: 0 --> 1

Char. 520: 1 --> 0

Char. 682: 1 --> 0

Char. 812: 1 --> 0

Char. 854: 1 --> 0

Char. 900: 1 --> 2

Char. 933: 0 --> 1

Char. 938: 0 --> 1

Char. 939: 0 --> 1

Char. 958: 0 --> 1

Zupaysaurus_rougieri :

Char. 1: 1 --> 2

Char. 44: 1 --> 0

Char. 79: 0 --> 1

Char. 103: 0 --> 1

Char. 105: 0 --> 1

Char. 165: 0 --> 1

Char. 170: 0 --> 1

Char. 342: 1 --> 0

Char. 343: 0 --> 1

Char. 409: 2 --> 3

Char. 434: 1 --> 2

Char. 534: 0 --> 1

Char. 631: 0 --> 1

Node 189 :

Char. 91: 0 --> 1

Char. 329: 1 --> 0

Char. 393: 1 --> 0

Char. 415: 1 --> 0

Char. 417: 2 --> 0

Char. 423: 12 --> 3

Char. 431: 1 --> 0

Char. 781: 1 --> 0

Node 190 :

No synapomorphies

Node 191 :

Char. 331: 0 --> 1

Char. 357: 0 --> 1

Char. 539: 0 --> 1

Char. 578: 0 --> 1

Node 192 :

Char. 88: 1 --> 0

Char. 117: 1 --> 0

Char. 140: 0 --> 1

Char. 203: 0 --> 1

Char. 223: 0 --> 1

Char. 266: 0 --> 1

Char. 436: 0 --> 1

Char. 650: 1 --> 0

Char. 676: 0 --> 1

Char. 678: 0 --> 1

Char. 755: 0 --> 1

Node 193 :

Char. 235: 0 --> 1

Char. 434: 1 --> 2

Char. 648: 0 --> 1

Char. 777: 0 --> 1

Char. 792: 0 --> 1

Char. 884: 1 --> 0

Node 194 :

Char. 69: 12 --> 0

Char. 322: 1 --> 2

Char. 404: 0 --> 1

Node 195 :

Char. 553: 1 --> 0

Char. 611: 0 --> 1

Char. 848: 2 --> 0

Char. 952: 0 --> 1

Node 196 :

Char. 58: 1 --> 2

Char. 266: 0 --> 1

Char. 444: 0 --> 1

Char. 592: 0 --> 2

Char. 808: 0 --> 1

Char. 844: 0 --> 1

Char. 845: 0 --> 1

Char. 885: 0 --> 1

Char. 898: 0 --> 1

Char. 908: 0 --> 1

Node 197 :

Char. 88: 1 --> 0

Char. 124: 0 --> 1

Char. 132: 1 --> 0

Char. 173: 1 --> 0

Char. 196: 1 --> 0

Char. 208: 1 --> 0

Char. 356: 1 --> 0

Char. 357: 0 --> 1

Char. 358: 0 --> 1

Char. 392: 0 --> 1

Char. 406: 1 --> 0

Char. 414: 1 --> 0

Char. 416: 12 --> 0

Char. 428: 1 --> 0

Char. 734: 0 --> 1

Char. 839: 1 --> 0

Char. 915: 1 --> 2

Char. 948: 1 --> 0

Node 198 :

Char. 209: 2 --> 0

Char. 250: 1 --> 0

Node 199 :

Char. 592: 2 --> 1

Char. 595: 0 --> 1

Char. 857: 1 --> 0

Node 200 :

Char. 59: 0 --> 1

Char. 91: 1 --> 2

Char. 102: 1 --> 0

Char. 108: 1 --> 0

Char. 213: 0 --> 1

Node 201 :

Char. 69: 12 --> 0

Char. 70: 0 --> 1

Char. 81: 0 --> 1

Char. 103: 0 --> 1

Char. 111: 0 --> 1

Char. 114: 0 --> 1

Char. 116: 0 --> 1

Char. 119: 0 --> 1

Char. 189: 0 --> 1

Char. 409: 1 --> 2

Char. 416: 0 --> 1

Char. 422: 3 --> 2

Node 202 :

Char. 3: 1 --> 0

Char. 9: 2 --> 3

Char. 15: 2 --> 1

Char. 16: 1 --> 2

Char. 33: 1 --> 2

Char. 40: 0 --> 1

Char. 86: 0 --> 1

Char. 141: 0 --> 1

Char. 166: 0 --> 1

Char. 177: 0 --> 1

Char. 185: 0 --> 1

Char. 198: 0 --> 1

Char. 222: 0 --> 1

Char. 285: 1 --> 0

Char. 300: 0 --> 1

Char. 311: 1 --> 0

Char. 363: 0 --> 1

Char. 391: 2 --> 01

Char. 420: 1 --> 0

Char. 454: 0 --> 1

Char. 457: 0 --> 1

Char. 795: 0 --> 1

Char. 854: 0 --> 1

Char. 937: 0 --> 2

Node 203 :

Char. 50: 0 --> 1

Char. 67: 0 --> 1

Char. 80: 0 --> 1

Char. 110: 0 --> 2

Char. 118: 0 --> 1

Char. 144: 0 --> 1

Char. 156: 0 --> 1

Char. 172: 1 --> 0

Char. 179: 1 --> 0

Char. 218: 0 --> 1

Char. 260: 1 --> 0

Char. 327: 0 --> 1

Char. 344: 0 --> 1

Char. 358: 1 --> 0

Char. 398: 0 --> 1

Char. 423: 3 --> 2

Char. 490: 0 --> 1

Char. 491: 0 --> 1

Char. 563: 0 --> 1

Char. 569: 0 --> 1

Char. 574: 2 --> 3

Char. 577: 0 --> 1

Char. 581: 0 --> 1

Char. 609: 0 --> 2

Char. 614: 1 --> 0

Char. 637: 0 --> 1

Char. 649: 0 --> 1

Char. 653: 1 --> 0

Char. 691: 2 --> 0

Char. 695: 1 --> 0

Char. 715: 1 --> 0

Char. 737: 0 --> 1

Char. 798: 0 --> 1

Char. 844: 1 --> 0

Char. 861: 0 --> 1

Char. 874: 0 --> 1

Char. 878: 0 --> 1

Char. 912: 0 --> 1

Char. 957: 0 --> 1

Char. 958: 0 --> 2

Node 204 :

Char. 8: 1 --> 0

Char. 113: 0 --> 1

Char. 117: 1 --> 0

Char. 153: 0 --> 1

Char. 178: 0 --> 1

Char. 192: 1 --> 0

Char. 843: 0 --> 1

Char. 919: 0 --> 1

Node 205 :

Char. 285: 0 --> 1

Char. 554: 0 --> 1

Char. 787: 1 --> 0

Char. 839: 0 --> 1

Char. 921: 0 --> 1

Node 206 :

Char. 15: 3 --> 2

Char. 16: 0 --> 1

Char. 19: 1 --> 0

Char. 28: 1 --> 0

Char. 45: 1 --> 0

Char. 75: 2 --> 0

Char. 172: 0 --> 1

Char. 241: 0 --> 1

Char. 250: 0 --> 1

Char. 260: 0 --> 1

Char. 391: 1 --> 2

Char. 513: 1 --> 0

Char. 514: 0 --> 1

Char. 553: 0 --> 1

Char. 562: 0 --> 1

Char. 585: 4 --> 3

Char. 672: 1 --> 2

Char. 703: 1 --> 0

Char. 862: 0 --> 1

Char. 906: 0 --> 1

Char. 946: 1 --> 2

Char. 947: 0 --> 1

Char. 956: 1 --> 2

Node 207 :

Char. 8: 0 --> 1

Char. 9: 1 --> 2

Char. 35: 1 --> 0

Char. 38: 0 --> 1

Char. 59: 1 --> 0

Char. 71: 0 --> 1

Char. 118: 1 --> 0

Char. 121: 0 --> 1

Char. 139: 1 --> 0

Char. 205: 0 --> 1

Char. 231: 0 --> 1

Char. 290: 1 --> 2

Char. 409: 2 --> 1

Char. 481: 0 --> 1

Char. 498: 0 --> 1

Char. 513: 2 --> 1

Char. 539: 0 --> 1

Char. 555: 0 --> 1

Char. 644: 0 --> 1

Char. 731: 1 --> 0

Char. 903: 1 --> 0

Node 208 :

Char. 7: 0 --> 1

Char. 17: 0 --> 1

Char. 102: 0 --> 1

Char. 104: 0 --> 1

Char. 112: 0 --> 1

Char. 128: 1 --> 0

Char. 245: 0 --> 1

Char. 296: 0 --> 1

Char. 549: 2 --> 1

Char. 598: 0 --> 1

Char. 599: 0 --> 1

Char. 797: 0 --> 1

Char. 877: 0 --> 1

Char. 892: 0 --> 1

Char. 902: 0 --> 1

Char. 917: 0 --> 1

Node 209 :

Char. 23: 0 --> 1

Char. 29: 0 --> 1

Char. 43: 0 --> 1

Char. 69: 1 --> 2

Char. 536: 0 --> 1

Char. 574: 1 --> 2

Char. 614: 0 --> 1

Char. 661: 0 --> 1

Char. 697: 0 --> 1

Char. 700: 0 --> 1

Char. 717: 1 --> 0

Char. 718: 0 --> 1

Char. 754: 0 --> 1

Char. 781: 0 --> 1

Char. 786: 0 --> 1

Char. 787: 0 --> 1

Char. 789: 1 --> 2

Char. 790: 0 --> 1

Char. 850: 0 --> 1

Char. 929: 1 --> 2

Node 210 :

Char. 15: 1 --> 2

Char. 71: 0 --> 1

Char. 399: 1 --> 2

Char. 429: 1 --> 0

Char. 463: 1 --> 0

Char. 539: 0 --> 1

Char. 562: 0 --> 1

Char. 565: 0 --> 1

Node 211 :

Char. 19: 0 --> 1

Char. 40: 0 --> 1

Char. 491: 0 --> 1

Char. 495: 0 --> 1

Node 212 :

Char. 128: 0 --> 1

Char. 486: 0 --> 1

Node 213 :

Char. 3: 1 --> 0

Char. 69: 2 --> 1

Char. 71: 1 --> 0

Char. 140: 0 --> 1

Char. 205: 1 --> 2

Char. 213: 0 --> 1

Char. 429: 0 --> 1

Char. 631: 0 --> 1

Char. 664: 0 --> 2

Char. 801: 1 --> 0

Char. 839: 1 --> 2

Char. 908: 1 --> 0

Char. 951: 0 --> 1

Node 214 :

Char. 144: 0 --> 1

Char. 147: 0 --> 1

Char. 161: 0 --> 1

Char. 163: 1 --> 2

Char. 168: 0 --> 1

Char. 292: 0 --> 1

Char. 307: 0 --> 1

Char. 363: 0 --> 2

Char. 417: 0 --> 1

Char. 422: 3 --> 2

Char. 423: 3 --> 2

Char. 452: 0 --> 1

Char. 473: 1 --> 0

Char. 479: 2 --> 3

Char. 709: 0 --> 1

Char. 711: 0 --> 1

Char. 775: 1 --> 0

Char. 844: 1 --> 0

Node 215 :

Char. 84: 1 --> 0

Char. 96: 1 --> 2

Char. 178: 1 --> 2

Char. 194: 0 --> 1

Char. 195: 0 --> 1

Char. 198: 0 --> 1

Char. 209: 0 --> 2

Char. 222: 0 --> 1

Char. 247: 0 --> 1

Char. 398: 0 --> 1

Char. 655: 1 --> 0

Char. 676: 0 --> 1

Char. 682: 1 --> 0

Char. 849: 0 --> 1

Char. 938: 0 --> 1

Char. 952: 0 --> 1

Node 216 :

Char. 562: 1 --> 0

Char. 832: 0 --> 1

Node 217 :

Char. 563: 0 --> 1

Char. 777: 0 --> 1

Char. 861: 0 --> 1

Node 218 :

Char. 45: 0 --> 12

Char. 72: 0 --> 1

Char. 126: 0 --> 1

Char. 141: 0 --> 1

Char. 172: 1 --> 0

Char. 385: 0 --> 1

Char. 457: 0 --> 1

Char. 637: 0 --> 1

Char. 672: 2 --> 1

Char. 806: 1 --> 0

Char. 840: 0 --> 1

Char. 854: 0 --> 1

Node 219 :

Char. 165: 0 --> 1

Char. 173: 0 --> 1

Char. 273: 1 --> 0

Char. 274: 1 --> 0

Char. 474: 0 --> 1

Char. 658: 1 --> 0

Char. 661: 1 --> 2

Char. 775: 0 --> 1

Char. 835: 0 --> 1

Node 220 :

Char. 32: 1 --> 0

Char. 86: 0 --> 2

Char. 253: 0 --> 1

Char. 343: 0 --> 1

Char. 376: 1 --> 0

Char. 424: 0 --> 1

Char. 467: 0 --> 1

Char. 473: 0 --> 1

Char. 488: 1 --> 0

Char. 517: 1 --> 0

Char. 539: 1 --> 0

Char. 541: 0 --> 1

Char. 564: 0 --> 1

Char. 682: 0 --> 1

Char. 707: 0 --> 1

Char. 756: 1 --> 0

Char. 886: 0 --> 2

Node 221 :

Char. 481: 1 --> 2

Char. 536: 1 --> 2

Char. 593: 0 --> 2

Char. 598: 1 --> 2

Char. 599: 1 --> 2

Char. 600: 0 --> 1

Char. 757: 0 --> 1

Char. 775: 12 --> 0

Char. 784: 0 --> 1

Node 222 :

Char. 886: 2 --> 1

Char. 887: 0 --> 1

Char. 893: 0 --> 1

Node 223 :

Char. 848: 0 --> 1

Char. 954: 1 --> 0

Node 224 :

Char. 663: 0 --> 1

Char. 680: 0 --> 1

Char. 908: 1 --> 0

Node 225 :

Char. 178: 1 --> 0

Char. 203: 0 --> 1

Char. 212: 0 --> 1

Char. 228: 0 --> 1

Char. 285: 1 --> 0

Char. 286: 0 --> 1

Char. 295: 1 --> 2

Char. 299: 0 --> 1

Node 226 :

Char. 80: 0 --> 1

Char. 180: 1 --> 0

Char. 215: 0 --> 2

Char. 217: 0 --> 1

Char. 454: 0 --> 1

Char. 491: 0 --> 1

Char. 614: 1 --> 0

Node 227 :

Char. 9: 2 --> 1

Char. 13: 0 --> 1

Char. 44: 1 --> 0

Char. 77: 0 --> 1

Char. 98: 0 --> 1

Char. 160: 1 --> 0

Char. 187: 1 --> 0

Char. 239: 0 --> 1

Char. 255: 1 --> 0

Char. 260: 1 --> 0

Char. 278: 0 --> 1

Char. 282: 2 --> 1

Char. 335: 1 --> 0

Char. 340: 1 --> 0

Char. 350: 0 --> 1

Char. 402: 0 --> 2

Char. 461: 0 --> 1

Char. 479: 2 --> 1

Char. 552: 1 --> 0

Char. 619: 0 --> 1

Char. 621: 1 --> 0

Char. 715: 1 --> 0

Char. 719: 0 --> 1

Char. 744: 0 --> 1

Char. 780: 0 --> 1

Char. 790: 1 --> 0

Char. 812: 0 --> 1

Char. 826: 1 --> 0

Char. 851: 0 --> 1

Char. 877: 1 --> 0

Char. 894: 0 --> 1

Char. 913: 0 --> 1

Char. 915: 1 --> 0

Char. 956: 2 --> 1

Node 228 :

Char. 44: 1 --> 0

Char. 58: 2 --> 1

Char. 63: 1 --> 0

Char. 140: 0 --> 1

Char. 343: 0 --> 1

Char. 349: 1 --> 0

Char. 460: 1 --> 2

Char. 478: 1 --> 2

Char. 479: 2 --> 3

Char. 480: 1 --> 0

Char. 501: 0 --> 1

Char. 509: 0 --> 1

Char. 539: 1 --> 0

Char. 689: 0 --> 1

Char. 832: 0 --> 1

Char. 862: 1 --> 2

Char. 888: 0 --> 1

Char. 931: 1 --> 2

Node 229 :

Char. 507: 1 --> 0

Char. 512: 1 --> 0

Char. 527: 1 --> 0

Char. 555: 1 --> 0

Node 230 :

Char. 513: 0 --> 2

Char. 520: 1 --> 0

Char. 549: 1 --> 2

Char. 554: 1 --> 0

Char. 561: 0 --> 1

Char. 562: 1 --> 0

Node 231 :

Char. 47: 1 --> 0

Char. 82: 1 --> 2

Char. 90: 0 --> 1

Char. 179: 1 --> 0

Char. 521: 0 --> 1

Char. 703: 0 --> 1

Char. 830: 0 --> 1

Char. 832: 0 --> 1

Node 232 :

Char. 797: 1 --> 0

Char. 873: 0 --> 1

Char. 906: 1 --> 0

Node 233 :

Char. 795: 0 --> 1

Char. 903: 0 --> 1

Node 234 :

Char. 91: 0 --> 1

Char. 143: 0 --> 1

Char. 197: 0 --> 1

Char. 228: 1 --> 0

Char. 270: 1 --> 0

Node 235 :

Char. 69: 1 --> 0

Char. 144: 0 --> 1

Char. 269: 1 --> 3

Char. 350: 1 --> 2

Char. 380: 0 --> 1

Char. 421: 0 --> 1

Char. 570: 1 --> 0

Char. 820: 1 --> 2

Char. 856: 0 --> 1

Node 236 :

Char. 6: 0 --> 1

Char. 10: 0 --> 2

Char. 41: 0 --> 1

Char. 53: 0 --> 1

Char. 155: 1 --> 2

Char. 170: 0 --> 1

Char. 342: 0 --> 1

Char. 362: 0 --> 1

Char. 461: 1 --> 0

Char. 536: 1 --> 2

Char. 936: 0 --> 1

Node 237 :

Char. 16: 2 --> 3

Char. 19: 0 --> 1

Char. 189: 0 --> 2

Char. 335: 0 --> 1

Char. 744: 1 --> 0

Node 238 :

Char. 182: 0 --> 1

Char. 672: 1 --> 2

Char. 694: 1 --> 0

Char. 879: 1 --> 0

Node 239 :

Char. 11: 0 --> 1

Char. 44: 1 --> 0

Char. 51: 0 --> 1

Char. 98: 0 --> 1

Char. 108: 1 --> 2

Char. 142: 0 --> 2

Char. 146: 0 --> 1

Char. 162: 0 --> 1

Char. 237: 0 --> 1

Char. 241: 1 --> 0

Char. 248: 1 --> 0

Char. 257: 1 --> 0

Char. 298: 1 --> 0

Char. 337: 1 --> 0

Char. 345: 1 --> 2

Char. 388: 1 --> 0

Char. 424: 1 --> 0

Char. 462: 0 --> 1

Char. 475: 2 --> 1

Char. 576: 0 --> 1

Char. 583: 0 --> 1

Char. 598: 1 --> 0

Char. 700: 2 --> 1

Char. 729: 1 --> 2

Char. 730: 0 --> 1

Char. 731: 0 --> 1

Char. 755: 1 --> 0

Char. 761: 2 --> 0

Char. 767: 0 --> 1

Char. 777: 1 --> 0

Char. 782: 0 --> 1

Char. 791: 0 --> 1

Char. 810: 0 --> 1

Char. 811: 1 --> 0

Char. 832: 1 --> 0

Char. 834: 0 --> 1

Char. 840: 1 --> 0

Char. 876: 1 --> 0

Char. 917: 0 --> 1

Char. 961: 0 --> 1

Node 240 :

Char. 38: 1 --> 0

Char. 63: 1 --> 0

Char. 110: 0 --> 1

Char. 192: 0 --> 1

Char. 318: 0 --> 1

Char. 370: 0 --> 1

Char. 657: 0 --> 1

Char. 659: 0 --> 1

Node 241 :

Char. 579: 0 --> 1

Char. 586: 0 --> 1

Char. 740: 0 --> 1

Char. 747: 0 --> 1

Char. 756: 0 --> 2

Char. 777: 1 --> 0

Node 242 :

Char. 98: 1 --> 0

Char. 273: 0 --> 2

Char. 288: 2 --> 0

Char. 335: 0 --> 1

Char. 363: 0 --> 1

Char. 380: 0 --> 1

Char. 388: 1 --> 0

Char. 553: 0 --> 1

Char. 554: 1 --> 0

Char. 661: 0 --> 1

Char. 675: 1 --> 0

Char. 676: 0 --> 1

Char. 686: 0 --> 1

Char. 728: 0 --> 1

Char. 739: 01 --> 2

Char. 742: 0 --> 1

Char. 860: 0 --> 1

Char. 900: 1 --> 2

Node 243 :

Char. 3: 1 --> 0

Char. 140: 1 --> 0

Char. 271: 0 --> 1

Char. 316: 1 --> 2

Char. 507: 1 --> 0

Char. 592: 2 --> 1

Char. 736: 0 --> 1

Char. 738: 0 --> 1

Char. 769: 0 --> 1

Node 244 :

Char. 175: 0 --> 1

Char. 201: 0 --> 1

Char. 206: 0 --> 1

Char. 242: 1 --> 0

Char. 250: 1 --> 0

Char. 278: 1 --> 0

Char. 298: 1 --> 0

Char. 302: 0 --> 1

Char. 348: 1 --> 0

Char. 418: 0 --> 1

Char. 427: 0 --> 2

Char. 442: 0 --> 1

Char. 520: 1 --> 0

Char. 526: 1 --> 0

Char. 532: 1 --> 0

Char. 589: 1 --> 0

Char. 613: 0 --> 2

Char. 628: 0 --> 1

Char. 682: 1 --> 0

Char. 844: 1 --> 0

Char. 903: 0 --> 1

Char. 938: 0 --> 1

Node 245 :

Char. 17: 0 --> 1

Char. 56: 0 --> 1

Char. 69: 1 --> 0

Char. 76: 1 --> 2

Char. 102: 1 --> 0

Char. 113: 1 --> 0

Char. 116: 0 --> 1

Char. 123: 1 --> 0

Char. 126: 1 --> 0

Char. 277: 0 --> 1

Char. 327: 0 --> 1

Char. 409: 1 --> 23

Char. 414: 0 --> 1

Char. 416: 0 --> 1

Char. 422: 3 --> 2

Char. 479: 1 --> 0

Char. 578: 0 --> 1

Char. 662: 0 --> 1

Char. 773: 0 --> 1

Char. 778: 0 --> 1

Char. 780: 1 --> 0

Char. 791: 0 --> 1

Char. 825: 0 --> 1

Char. 836: 0 --> 1

Char. 920: 0 --> 1

Node 246 :

Char. 540: 0 --> 1

Char. 565: 0 --> 1

Char. 592: 0 --> 2

Char. 675: 0 --> 1

Char. 694: 1 --> 0

Node 247 :

Char. 130: 0 --> 1

Char. 761: 2 --> 0

Node 248 :

Char. 69: 2 --> 1

Char. 72: 1 --> 0

Char. 398: 0 --> 1

Node 249 :

Char. 16: 2 --> 1

Char. 99: 0 --> 1

Char. 104: 1 --> 0

Char. 118: 0 --> 1

Char. 139: 0 --> 1

Char. 495: 0 --> 1

Char. 806: 0 --> 1

Char. 906: 1 --> 0

Node 250 :

Char. 7: 1 --> 0

Char. 38: 1 --> 0

Char. 96: 1 --> 0

Char. 127: 2 --> 1

Char. 141: 1 --> 0

Char. 205: 1 --> 0

Char. 231: 1 --> 0

Char. 263: 0 --> 1

Char. 467: 1 --> 0

Char. 510: 0 --> 1

Char. 536: 1 --> 0

Char. 605: 1 --> 0

Char. 801: 1 --> 0

Char. 810: 0 --> 1

Char. 818: 1 --> 0

Char. 837: 0 --> 1

Char. 892: 1 --> 0

Node 251 :

Char. 441: 0 --> 1

Char. 532: 1 --> 0

Char. 589: 1 --> 0

Char. 831: 0 --> 1

Char. 847: 0 --> 1

Char. 929: 2 --> 0

Char. 958: 0 --> 1

Node 252 :

Char. 328: 0 --> 1

Char. 329: 0 --> 1

Char. 341: 0 --> 1

Char. 393: 0 --> 1

Char. 618: 1 --> 0

Char. 761: 0 --> 1

Char. 783: 1 --> 0

Char. 796: 1 --> 2

Char. 816: 1 --> 2

Char. 820: 0 --> 1

Char. 839: 1 --> 2

Char. 891: 0 --> 1

Char. 940: 0 --> 1

Node 253 :

Char. 177: 0 --> 1

Char. 295: 1 --> 2

Char. 416: 1 --> 2

Char. 436: 0 --> 1

Char. 488: 0 --> 1

Char. 498: 1 --> 2

Char. 513: 1 --> 2

Char. 514: 1 --> 2

Char. 531: 0 --> 1

Char. 543: 0 --> 1

Char. 588: 0 --> 1

Char. 710: 0 --> 1

Char. 760: 0 --> 1

Char. 766: 0 --> 1

Char. 807: 0 --> 1

Char. 810: 1 --> 0

Char. 857: 1 --> 0

Char. 901: 0 --> 1

Char. 927: 0 --> 1

Char. 947: 1 --> 2

Node 254 :

Char. 9: 1 --> 0

Char. 44: 0 --> 1

Char. 100: 0 --> 1

Char. 105: 0 --> 1

Char. 166: 0 --> 1

Char. 170: 0 --> 1

Char. 186: 0 --> 1

Char. 216: 0 --> 1

Char. 251: 0 --> 1

Char. 256: 0 --> 2

Char. 259: 0 --> 1

Char. 262: 0 --> 1

Char. 266: 1 --> 0

Char. 273: 0 --> 1

Char. 335: 0 --> 1

Char. 345: 1 --> 0

Char. 414: 1 --> 0

Char. 416: 1 --> 0

Char. 422: 2 --> 3

Char. 455: 1 --> 0

Char. 499: 0 --> 1

Char. 588: 0 --> 1

Char. 591: 0 --> 1

Char. 598: 1 --> 0

Char. 609: 0 --> 4

Char. 617: 0 --> 1

Char. 620: 0 --> 2

Char. 629: 0 --> 1

Char. 632: 0 --> 1

Char. 635: 0 --> 1

Char. 648: 0 --> 1

Char. 673: 1 --> 0

Char. 681: 0 --> 1

Char. 721: 0 --> 1

Char. 753: 0 --> 1

Char. 761: 0 --> 1

Char. 770: 0 --> 1

Char. 779: 0 --> 1

Char. 825: 1 --> 2

Char. 848: 0 --> 1

Char. 939: 0 --> 1

Char. 950: 0 --> 1

Char. 954: 1 --> 0

Char. 955: 1 --> 0

Char. 956: 12 --> 0

Char. 958: 0 --> 1

Node 255 :

Char. 80: 0 --> 1

Char. 120: 0 --> 1

Char. 215: 0 --> 2

Char. 288: 2 --> 1

Char. 295: 1 --> 0

Char. 306: 0 --> 1

Char. 339: 0 --> 1

Char. 478: 1 --> 0

Char. 480: 1 --> 0

Char. 490: 1 --> 0

Char. 548: 0 --> 2

Char. 623: 0 --> 1

Char. 641: 1 --> 0

Char. 683: 1 --> 0

Char. 698: 0 --> 1

Char. 735: 0 --> 1

Char. 772: 0 --> 1

Char. 782: 0 --> 1

Char. 783: 1 --> 0

Char. 787: 0 --> 1

Char. 796: 1 --> 2

Char. 815: 0 --> 1

Char. 847: 0 --> 1

Char. 852: 2 --> 1

Char. 855: 1 --> 2

Char. 902: 1 --> 0

Char. 919: 1 --> 0

Char. 940: 0 --> 1

Char. 965: 0 --> 1

Node 256 :

Char. 125: 0 --> 1

Char. 238: 0 --> 1

Char. 339: 0 --> 1

Char. 574: 3 --> 4

Char. 631: 0 --> 1

Char. 664: 0 --> 1

Char. 712: 0 --> 1

Char. 714: 1 --> 0

Char. 748: 1 --> 2

Char. 860: 0 --> 1

Node 257 :

Char. 38: 0 --> 2

Char. 52: 0 --> 1

Char. 69: 0 --> 2

Char. 164: 0 --> 1

Char. 242: 0 --> 1

Char. 433: 0 --> 1

Char. 487: 2 --> 0

Char. 499: 0 --> 1

Char. 520: 0 --> 1

Char. 690: 0 --> 1

Char. 744: 1 --> 0

Char. 830: 1 --> 2

Char. 888: 0 --> 1

Char. 902: 1 --> 0

Char. 928: 0 --> 1

Char. 936: 0 --> 1

Node 258 :

Char. 180: 0 --> 1

Char. 214: 1 --> 0

Char. 408: 0 --> 1

Char. 492: 0 --> 1

Char. 514: 1 --> 2

Char. 540: 1 --> 0

Char. 565: 1 --> 0

Char. 592: 1 --> 0

Char. 613: 2 --> 0

Char. 656: 0 --> 1

Char. 658: 0 --> 1

Char. 666: 0 --> 1

Char. 667: 1 --> 0

Char. 672: 1 --> 2

Char. 675: 1 --> 0

Char. 678: 1 --> 0

Char. 747: 0 --> 1

Char. 810: 1 --> 0

Char. 854: 0 --> 1

Char. 919: 1 --> 0

Char. 933: 0 --> 1

Node 259 :

Char. 34: 01 --> 2

Char. 87: 0 --> 1

Char. 280: 0 --> 1

Char. 491: 0 --> 1

Char. 549: 1 --> 2

Char. 609: 0 --> 3

Char. 614: 1 --> 0

Char. 642: 0 --> 1

Char. 662: 1 --> 0

Char. 726: 2 --> 1

Char. 906: 0 --> 1

Char. 953: 0 --> 1

Char. 955: 1 --> 2

Char. 960: 1 --> 0

Char. 964: 0 --> 1

Node 260 :

Char. 14: 0 --> 1

Char. 33: 1 --> 0

Char. 39: 0 --> 1

Char. 56: 1 --> 0

Char. 65: 0 --> 12

Char. 70: 0 --> 1

Char. 195: 0 --> 1

Char. 326: 0 --> 1

Char. 345: 1 --> 2

Char. 354: 0 --> 1

Char. 356: 1 --> 0

Char. 360: 1 --> 0

Char. 372: 0 --> 1

Char. 394: 0 --> 1

Char. 534: 0 --> 1

Char. 536: 01 --> 2

Char. 593: 0 --> 2

Char. 618: 1 --> 0

Char. 631: 0 --> 1

Char. 838: 0 --> 1

Char. 860: 0 --> 1

Node 261 :

Char. 0: 1 --> 0

Char. 1: 1 --> 0

Char. 23: 2 --> 1

Char. 27: 0 --> 1

Char. 52: 0 --> 1

Char. 98: 1 --> 2

Char. 104: 0 --> 1

Char. 322: 1 --> 2

Char. 324: 0 --> 1

Char. 328: 0 --> 1

Char. 329: 0 --> 1

Char. 344: 0 --> 2

Char. 388: 1 --> 0

Char. 408: 0 --> 1

Char. 433: 0 --> 1

Char. 445: 0 --> 1

Char. 446: 0 --> 1

Char. 587: 0 --> 1

Char. 953: 0 --> 1

Char. 955: 1 --> 2

Char. 957: 0 --> 1

Char. 960: 1 --> 0

Node 262 :

Char. 326: 0 --> 1

Char. 447: 0 --> 1

Char. 635: 1 --> 0

Char. 805: 0 --> 1

Char. 836: 1 --> 2

Node 263 :

Char. 446: 0 --> 1

Char. 534: 0 --> 1

Char. 574: 4 --> 5

Char. 625: 0 --> 1

Char. 629: 1 --> 2

Char. 633: 0 --> 1

Char. 673: 0 --> 1

Char. 699: 2 --> 3

Char. 702: 0 --> 1

Char. 750: 0 --> 1

Char. 752: 0 --> 1

Char. 815: 1 --> 0

Char. 945: 0 --> 1

Char. 960: 1 --> 0

Node 264 :

Char. 14: 0 --> 1

Char. 15: 2 --> 3

Char. 17: 1 --> 0

Char. 19: 0 --> 1

Char. 20: 1 --> 0

Char. 22: 1 --> 0

Char. 24: 0 --> 1

Char. 93: 1 --> 0

Char. 181: 0 --> 1

Char. 339: 1 --> 0

Char. 348: 0 --> 1

Char. 408: 0 --> 1

Char. 460: 1 --> 0

Char. 571: 0 --> 1

Char. 753: 2 --> 3

Char. 793: 1 --> 0

Char. 916: 0 --> 1

Char. 917: 0 --> 1

Node 265 :

Char. 16: 1 --> 0

Char. 30: 0 --> 1

Char. 32: 0 --> 1

Char. 49: 0 --> 1

Char. 53: 1 --> 0

Char. 58: 2 --> 0

Char. 61: 2 --> 0

Char. 98: 1 --> 2

Char. 132: 0 --> 1

Char. 215: 2 --> 0

Char. 316: 1 --> 2

Char. 317: 1 --> 2

Char. 329: 0 --> 1

Char. 335: 1 --> 0

Char. 343: 1 --> 0

Char. 396: 1 --> 0

Char. 481: 0 --> 3

Char. 574: 3 --> 4

Char. 585: 3 --> 0

Char. 587: 0 --> 1

Char. 590: 0 --> 2

Char. 652: 1 --> 0

Char. 655: 1 --> 2

Char. 662: 1 --> 0

Char. 699: 0 --> 2

Char. 717: 0 --> 1

Char. 753: 1 --> 2

Char. 763: 1 --> 0

Char. 777: 1 --> 0

Char. 803: 1 --> 0

Char. 807: 0 --> 1

Char. 838: 0 --> 1

Char. 853: 1 --> 2

Char. 911: 0 --> 1

Char. 912: 0 --> 1

Char. 923: 0 --> 1

Char. 925: 0 --> 1

Char. 942: 1 --> 0

Char. 950: 1 --> 0

Node 266 :

Char. 877: 0 --> 1

Char. 884: 2 --> 1

Node 267 :

Char. 39: 0 --> 1

Char. 83: 0 --> 1

Char. 198: 0 --> 1

Char. 218: 0 --> 1

Char. 228: 0 --> 1

Char. 234: 0 --> 1

Char. 236: 0 --> 1

Char. 238: 0 --> 1

Char. 242: 1 --> 0

Char. 248: 1 --> 2

Char. 268: 2 --> 3

Char. 271: 1 --> 2

Char. 275: 0 --> 1

Char. 431: 0 --> 1

Char. 492: 0 --> 1

Char. 498: 1 --> 0

Char. 521: 0 --> 1

Char. 557: 0 --> 2

Char. 559: 0 --> 1

Char. 796: 0 --> 1

Node 268 :

Char. 61: 2 --> 0

Char. 271: 0 --> 1

Char. 505: 1 --> 0

Char. 515: 0 --> 1

Node 269 :

Char. 8: 0 --> 1

Char. 69: 1 --> 0

Char. 110: 0 --> 2

Char. 117: 0 --> 1

Char. 127: 2 --> 3

Char. 135: 2 --> 1

Char. 197: 0 --> 1

Char. 331: 0 --> 1

Char. 350: 0 --> 1

Char. 411: 0 --> 1

Char. 478: 1 --> 2

Char. 777: 0 --> 1

Node 270 :

Char. 19: 0 --> 1

Char. 772: 0 --> 1

Char. 824: 0 --> 1

Char. 834: 0 --> 1

Node 271 :

Char. 420: 1 --> 0

Char. 570: 1 --> 0

Char. 782: 0 --> 1

Node 272 :

Char. 193: 0 --> 1

Char. 204: 0 --> 1

Char. 299: 0 --> 1

Char. 300: 0 --> 1

Char. 424: 1 --> 0

Char. 548: 2 --> 0

Char. 608: 0 --> 1

Node 273 :

Char. 69: 1 --> 0

Char. 189: 0 --> 2

Char. 208: 0 --> 1

Char. 209: 2 --> 0

Char. 240: 0 --> 1

Char. 901: 0 --> 1

Char. 902: 1 --> 0

Node 274 :

Char. 127: 2 --> 3

Char. 191: 0 --> 1

Char. 209: 2 --> 0

Char. 241: 1 --> 0

Node 275 :

Char. 29: 0 --> 2

Char. 110: 2 --> 1

Char. 122: 0 --> 1

Char. 153: 1 --> 0

Char. 242: 1 --> 0

Char. 252: 0 --> 1

Char. 295: 0 --> 2

Node 276 :

Char. 13: 0 --> 1

Char. 140: 0 --> 1

Char. 149: 0 --> 1

Char. 155: 1 --> 0

Char. 338: 0 --> 1

Char. 417: 0 --> 1

Char. 569: 1 --> 0

Char. 649: 1 --> 0

Char. 657: 0 --> 1

Char. 665: 0 --> 1

Char. 667: 1 --> 2

Char. 669: 0 --> 1

Char. 683: 1 --> 0

Node 277 :

Char. 106: 0 --> 1

Char. 113: 1 --> 0

Char. 155: 1 --> 2

Char. 165: 0 --> 1

Char. 598: 1 --> 0

Char. 678: 0 --> 1

Char. 723: 0 --> 1

Char. 725: 0 --> 1

Char. 749: 0 --> 1

Char. 767: 0 --> 1

Char. 787: 0 --> 1

Char. 887: 0 --> 1

Char. 889: 0 --> 1

Char. 904: 0 --> 1

Node 278 :

Char. 42: 0 --> 1

Char. 63: 1 --> 0

Char. 108: 1 --> 0

Char. 109: 0 --> 12

Char. 332: 0 --> 1

Char. 447: 0 --> 1

Char. 575: 0 --> 1

Char. 670: 0 --> 1

Node 279 :

Char. 19: 0 --> 1

Char. 34: 1 --> 3

Char. 38: 0 --> 1

Char. 78: 0 --> 1

Char. 97: 0 --> 1

Char. 127: 1 --> 0

Char. 173: 0 --> 1

Char. 184: 1 --> 0

Char. 206: 1 --> 0

Char. 339: 1 --> 0

Char. 355: 0 --> 1

Char. 524: 1 --> 0

Char. 535: 0 --> 2

Char. 591: 0 --> 1

Char. 787: 1 --> 0

Char. 803: 1 --> 3

Char. 853: 1 --> 2

Char. 927: 0 --> 1

Char. 957: 1 --> 0

Node 280 :

Char. 58: 2 --> 0

Char. 103: 0 --> 1

Char. 137: 0 --> 1

Char. 342: 1 --> 0

Node 281 :

Char. 631: 0 --> 1

Char. 912: 0 --> 1

Char. 925: 0 --> 1

Node 282 :

Char. 642: 1 --> 0

Node 283 :

Char. 91: 1 --> 0

Char. 117: 1 --> 0

Char. 336: 1 --> 0

Char. 391: 1 --> 0

Char. 434: 1 --> 2

Char. 534: 0 --> 1

Char. 750: 0 --> 1

Node 284 :

Char. 752: 0 --> 1

Node 285 :

Char. 19: 0 --> 1

Char. 427: 0 --> 1

Char. 439: 0 --> 1

Char. 562: 0 --> 1

Char. 589: 1 --> 0

Char. 737: 0 --> 1

Char. 840: 1 --> 0

Node 286 :

Char. 44: 0 --> 1

Char. 98: 1 --> 0

Char. 532: 1 --> 2

Char. 617: 0 --> 1

Char. 705: 0 --> 1

Char. 708: 0 --> 1

Char. 748: 0 --> 1

Char. 919: 1 --> 0

Node 287 :

Char. 214: 1 --> 0

Char. 235: 0 --> 1

Char. 249: 0 --> 1

Char. 492: 0 --> 1

Char. 561: 0 --> 1

Char. 727: 0 --> 1

Char. 728: 0 --> 1

Node 288 :

Char. 14: 1 --> 0

Char. 94: 0 --> 1

Char. 135: 0 --> 1

Char. 175: 1 --> 0

Char. 529: 0 --> 1

Char. 748: 0 --> 1

Node 289 :

Char. 494: 1 --> 0

Node 290 :

Char. 473: 1 --> 0

Char. 498: 1 --> 0

Char. 521: 0 --> 1

Node 291 :

Char. 540: 0 --> 1

Char. 598: 1 --> 0

Node 292 :

Char. 41: 1 --> 0

Char. 175: 0 --> 1

Char. 203: 1 --> 0

Char. 232: 1 --> 0

Char. 351: 0 --> 1

Char. 385: 1 --> 0

Char. 803: 1 --> 2

Node 293 :

Char. 60: 0 --> 2

Char. 113: 1 --> 0

Char. 335: 1 --> 0

Char. 550: 0 --> 1

Char. 739: 1 --> 0

Node 294 :

Char. 3: 1 --> 0

Char. 6: 0 --> 1

Char. 19: 0 --> 1

Char. 23: 2 --> 1

Char. 34: 0 --> 2

Char. 38: 0 --> 1

Char. 206: 1 --> 0

Char. 207: 0 --> 1

Char. 290: 0 --> 1

Char. 340: 0 --> 1

Char. 343: 1 --> 0

Char. 404: 2 --> 0

Char. 417: 1 --> 0

Char. 423: 2 --> 3

Char. 470: 1 --> 0

Char. 524: 1 --> 0

Char. 536: 0 --> 2

Char. 549: 1 --> 0

Char. 555: 0 --> 1

Char. 560: 0 --> 1

Char. 562: 0 --> 1

Char. 635: 1 --> 0

Char. 675: 1 --> 0

Char. 821: 0 --> 1

Char. 825: 2 --> 1

Char. 833: 0 --> 1

Char. 855: 2 --> 1

Node 295 :

Char. 44: 1 --> 0

Char. 64: 0 --> 1

Char. 65: 0 --> 1

Char. 91: 1 --> 0

Char. 97: 0 --> 1

Char. 98: 1 --> 2

Char. 145: 0 --> 1

Char. 166: 1 --> 0

Char. 173: 0 --> 1

Char. 198: 0 --> 2

Char. 208: 1 --> 0

Char. 246: 1 --> 0

Char. 335: 1 --> 0

Char. 347: 0 --> 1

Char. 380: 0 --> 1

Char. 393: 1 --> 0

Char. 399: 1 --> 2

Char. 409: 23 --> 1

Char. 419: 0 --> 1

Char. 499: 1 --> 0

Char. 512: 0 --> 1

Char. 546: 1 --> 0

Char. 553: 0 --> 1

Char. 571: 0 --> 1

Char. 701: 1 --> 0

Char. 753: 1 --> 2

Char. 854: 0 --> 1

Char. 927: 0 --> 1

Char. 935: 0 --> 1

Char. 948: 0 --> 1

Char. 960: 1 --> 2

Node 296 :

Char. 398: 0 --> 1

Char. 713: 1 --> 0

Node 297 :

Char. 938: 0 --> 1

Node 298 :

Char. 548: 0 --> 1

Char. 646: 1 --> 0

Char. 725: 0 --> 1

Char. 731: 0 --> 1

Char. 854: 1 --> 2

Char. 960: 1 --> 0

Node 299 :

Char. 82: 2 --> 1

Char. 331: 0 --> 1

Node 300 :

Char. 86: 2 --> 1

Char. 562: 1 --> 0

Char. 682: 1 --> 0

Char. 772: 0 --> 2

Char. 937: 1 --> 0

Node 301 :

Char. 63: 1 --> 0

Char. 658: 0 --> 1

Char. 903: 1 --> 0

Node 302 :

Char. 499: 0 --> 1

Char. 510: 0 --> 1

Char. 514: 1 --> 2

Char. 534: 0 --> 1

Char. 563: 1 --> 0

Char. 592: 2 --> 0

Char. 636: 1 --> 0

Char. 641: 0 --> 1

Char. 644: 1 --> 2

Char. 657: 0 --> 1

Char. 826: 1 --> 0

Char. 907: 0 --> 1

Char. 919: 1 --> 0

Node 303 :

Char. 953: 0 --> 1

Char. 954: 1 --> 2

Node 304 :

Char. 60: 2 --> 0

Char. 180: 1 --> 0

Char. 381: 0 --> 1

Char. 565: 0 --> 1

Char. 854: 2 --> 1

Node 305 :

Char. 598: 1 --> 0

Char. 631: 1 --> 0

Char. 642: 1 --> 0

Char. 826: 1 --> 0

Node 306 :

Char. 133: 1 --> 0

Char. 330: 0 --> 1

Char. 333: 0 --> 1

Char. 482: 0 --> 1

Char. 549: 1 --> 0

Char. 605: 1 --> 0

Node 307 :

Char. 327: 2 --> 1

Char. 355: 1 --> 0

Char. 763: 1 --> 0

Node 308 :

Char. 381: 0 --> 1

Char. 534: 1 --> 0

Node 309 :

Char. 39: 1 --> 0

Char. 70: 1 --> 0

Node 310 :

Char. 388: 0 --> 1

Char. 753: 1 --> 2

Char. 793: 0 --> 1

Char. 811: 0 --> 1

Node 311 :

Char. 549: 2 --> 0

Char. 785: 0 --> 1

Char. 787: 0 --> 1

Node 312 :

Char. 28: 1 --> 0

Char. 69: 1 --> 0

Char. 123: 0 --> 1

Char. 127: 1 --> 2

Char. 140: 0 --> 1

Char. 165: 0 --> 1

Char. 637: 0 --> 1

Char. 693: 1 --> 0

Char. 756: 1 --> 0

Char. 809: 0 --> 1

Char. 905: 0 --> 1

Node 313 :

Char. 13: 1 --> 2

Char. 23: 1 --> 0

Char. 44: 0 --> 1

Char. 72: 1 --> 0

Char. 335: 0 --> 1

Char. 407: 0 --> 1

Node 314 :

Char. 124: 1 --> 0

Char. 154: 0 --> 1

Char. 376: 1 --> 0

Char. 595: 0 --> 1

Char. 808: 1 --> 0

Char. 809: 0 --> 1

Char. 912: 0 --> 1

Node 315 :

Char. 64: 1 --> 0

Char. 88: 0 --> 1

Char. 101: 1 --> 0

Char. 214: 1 --> 0

Char. 230: 1 --> 0

Char. 242: 0 --> 1

Char. 263: 1 --> 0

Char. 404: 0 --> 1

Char. 406: 1 --> 0

Char. 428: 1 --> 0

Char. 467: 1 --> 0

Node 316 :

Char. 452: 0 --> 1

Char. 531: 1 --> 0

Node 317 :

Char. 495: 1 --> 0

Char. 659: 0 --> 1

Char. 689: 0 --> 1

Node 318 :

Char. 16: 1 --> 0

Char. 49: 0 --> 1

Char. 57: 0 --> 1

Char. 127: 1 --> 0

Char. 345: 0 --> 1

Char. 389: 1 --> 2

Char. 392: 0 --> 1

Char. 804: 0 --> 1

Char. 820: 0 --> 1

Char. 824: 0 --> 1

Char. 936: 0 --> 1

Char. 949: 1 --> 2

Node 319 :

Char. 530: 1 --> 0

Char. 547: 0 --> 2

Char. 594: 0 --> 1

Char. 652: 1 --> 0

Char. 662: 1 --> 0

Char. 690: 0 --> 1

Char. 753: 12 --> 3

Char. 782: 0 --> 1

Char. 809: 1 --> 0

Char. 853: 1 --> 2

Char. 871: 0 --> 1

Char. 880: 0 --> 1

Char. 894: 1 --> 0

Char. 905: 0 --> 1

Char. 915: 0 --> 1

Char. 916: 0 --> 1

Char. 936: 0 --> 2

Node 320 :

Char. 912: 0 --> 1

Char. 919: 1 --> 0

Node 321 :

Char. 947: 2 --> 1

Char. 953: 1 --> 2

Node 322 :

Char. 598: 1 --> 2

Char. 600: 0 --> 1

Node 323 :

Char. 192: 0 --> 1

Char. 273: 0 --> 1

Char. 750: 0 --> 1

Node 324 :

Char. 19: 1 --> 0

Char. 21: 1 --> 0

Char. 198: 1 --> 0

Char. 200: 0 --> 1

Char. 227: 0 --> 1

Char. 244: 1 --> 0

Char. 254: 1 --> 0

Char. 256: 0 --> 1

Char. 268: 4 --> 3

Char. 277: 0 --> 1

Char. 327: 0 --> 1

Char. 417: 0 --> 1

Char. 438: 1 --> 2

Char. 441: 0 --> 1

Char. 784: 0 --> 1

Char. 870: 0 --> 1

Char. 933: 0 --> 1

Node 325 :

Char. 28: 0 --> 1

Char. 67: 0 --> 1

Char. 69: 1 --> 0

Char. 71: 1 --> 0

Char. 72: 1 --> 0

Char. 75: 0 --> 1

Char. 153: 1 --> 0

Synapomorphies for Consensus implied weight K=16

Tree 9 :

Silesaurus_opolensis :

No autapomorphies

Eoraptor_lunensis :

Char. 1: 1 --> 0

Char. 10: 0 --> 1

Char. 21: 0 --> 1

Char. 45: 1 --> 0

Char. 54: 0 --> 1

Char. 103: 0 --> 1

Char. 128: 1 --> 0

Char. 135: 1 --> 0

Char. 193: 0 --> 1

Char. 250: 0 --> 1

Char. 328: 0 --> 1

Char. 348: 0 --> 1

Char. 349: 1 --> 0

Char. 387: 0 --> 1

Char. 391: 1 --> 0

Char. 432: 1 --> 0

Char. 513: 2 --> 1

Char. 642: 1 --> 0

Char. 650: 1 --> 0

Char. 713: 1 --> 0

Char. 746: 0 --> 1

Char. 877: 0 --> 1

Char. 917: 0 --> 1

Bagualosaurus_agudoensis :

Char. 858: 1 --> 0

Buriolestes_schultzi :

Char. 36: 0 --> 1

Char. 43: 0 --> 1

Char. 52: 1 --> 0

Char. 112: 0 --> 1

Char. 116: 0 --> 1

Char. 179: 1 --> 0

Char. 398: 1 --> 0

Char. 406: 1 --> 0

Char. 412: 0 --> 1

Char. 414: 1 --> 0

Char. 416: 2 --> 0

Char. 417: 2 --> 01

Char. 598: 0 --> 1

Char. 599: 0 --> 1

Char. 668: 0 --> 1

Char. 673: 0 --> 1

Char. 852: 0 --> 1

Char. 900: 1 --> 2

Char. 905: 0 --> 1

Char. 908: 0 --> 1

Plateosaurus_trossingensis :

Char. 549: 2 --> 1

Char. 554: 0 --> 1

Char. 580: 0 --> 1

Char. 644: 2 --> 1

Char. 671: 0 --> 1

Char. 705: 0 --> 1

Char. 732: 1 --> 0

Char. 790: 0 --> 1

Char. 791: 0 --> 1

Char. 792: 1 --> 0

Char. 858: 1 --> 0

Char. 946: 1 --> 0

Char. 951: 0 --> 1

Pampadromaeus_barberenai :

Char. 91: 0 --> 1

Char. 95: 2 --> 1

Char. 127: 1 --> 0

Char. 305: 0 --> 1

Char. 367: 0 --> 1

Char. 436: 1 --> 0

Char. 549: 2 --> 1

Char. 606: 0 --> 1

Char. 635: 0 --> 1

Char. 842: 1 --> 0

Saturnalia_tupiniquim :

Char. 101: 0 --> 1

Char. 155: 1 --> 0

Char. 276: 1 --> 0

Char. 288: 0 --> 1

Char. 404: 0 --> 1

Char. 438: 0 --> 2

Char. 441: 0 --> 2

Char. 649: 0 --> 1

Char. 673: 0 --> 1

Char. 772: 0 --> 2

Char. 839: 1 --> 0

Char. 852: 0 --> 1

Guaibasaurus_candelariensis :

Char. 584: 2 --> 0

Char. 603: 0 --> 1

Char. 606: 0 --> 1

Char. 718: 0 --> 1

Char. 816: 1 --> 0

Char. 846: 1 --> 0

Char. 917: 0 --> 1

Eodromaeus_murphi :

Char. 38: 0 --> 1

Char. 45: 1 --> 0

Char. 47: 1 --> 0

Char. 72: 0 --> 1

Char. 76: 0 --> 1

Char. 114: 0 --> 1

Char. 228: 0 --> 1

Char. 288: 0 --> 1

Char. 391: 1 --> 2

Char. 409: 2 --> 1

Char. 432: 1 --> 0

Char. 475: 0 --> 2

Char. 649: 0 --> 1

Char. 664: 0 --> 1

Char. 692: 1 --> 0

Char. 803: 1 --> 0

Char. 862: 0 --> 1

Char. 909: 1 --> 0

Abelisaurus_comahuensis :

Char. 19: 0 --> 1

Char. 288: 0 --> 1

Acrocanthosaurus_atokensis :

Char. 15: 1 --> 2

Char. 22: 1 --> 0

Char. 49: 0 --> 1

Char. 62: 1 --> 0

Char. 74: 0 --> 1

Char. 91: 1 --> 2

Char. 185: 0 --> 1

Char. 194: 1 --> 0

Char. 236: 0 --> 1

Char. 251: 0 --> 1

Char. 252: 0 --> 1

Char. 261: 0 --> 1

Char. 348: 1 --> 2

Char. 373: 1 --> 0

Char. 382: 1 --> 2

Char. 386: 0 --> 1

Char. 399: 1 --> 2

Char. 487: 0 --> 2

Char. 516: 1 --> 0

Char. 543: 0 --> 1

Char. 562: 0 --> 1

Char. 671: 0 --> 1

Char. 672: 1 --> 0

Char. 677: 0 --> 1

Char. 679: 0 --> 2

Char. 687: 0 --> 1

Char. 767: 0 --> 1

Char. 801: 0 --> 1

Char. 839: 2 --> 1

Char. 841: 0 --> 1

Char. 879: 1 --> 0

Aerosteon_riocoloradensis :

Char. 155: 1 --> 0

Char. 157: 0 --> 1

Char. 558: 0 --> 1

Char. 566: 0 --> 1

Char. 631: 0 --> 1

Char. 637: 1 --> 0

Char. 803: 1 --> 2

Afromimus_tenerensis :

Char. 896: 0 --> 1

Afrovenator_abakensis :

Char. 40: 0 --> 1

Char. 54: 0 --> 1

Char. 59: 0 --> 1

Char. 63: 0 --> 1

Char. 94: 0 --> 1

Char. 96: 1 --> 2

Char. 113: 1 --> 0

Char. 115: 0 --> 1

Char. 467: 1 --> 0

Char. 532: 1 --> 2

Char. 668: 0 --> 1

Char. 706: 0 --> 1

Char. 711: 0 --> 1

Char. 803: 1 --> 2

Char. 809: 1 --> 0

Char. 860: 0 --> 1

Char. 903: 0 --> 1

Albertosaurus_sarcophagus :

Char. 127: 2 --> 3

Char. 149: 0 --> 2

Char. 191: 0 --> 1

Char. 271: 0 --> 1

Char. 278: 1 --> 0

Char. 368: 1 --> 0

Alioramus :

Char. 0: 1 --> 2

Char. 3: 1 --> 0

Char. 7: 1 --> 0

Char. 59: 0 --> 1

Char. 60: 0 --> 1

Char. 105: 0 --> 1

Char. 127: 2 --> 1

Char. 227: 0 --> 1

Char. 235: 0 --> 1

Char. 253: 1 --> 0

Char. 277: 0 --> 1

Char. 293: 0 --> 1

Char. 350: 1 --> 0

Char. 381: 1 --> 0

Char. 382: 1 --> 0

Char. 386: 0 --> 1

Char. 409: 1 --> 2

Char. 451: 2 --> 0

Char. 455: 1 --> 0

Char. 470: 1 --> 0

Char. 483: 1 --> 0

Char. 582: 0 --> 1

Char. 773: 0 --> 1

Char. 784: 0 --> 1

Char. 842: 1 --> 0

Alpkarakush_kyrgyzicus :

Char. 139: 0 --> 2

Char. 152: 0 --> 1

Char. 154: 1 --> 0

Char. 173: 1 --> 0

Char. 176: 1 --> 0

Char. 550: 0 --> 1

Char. 569: 0 --> 1

Char. 723: 0 --> 1

Char. 766: 0 --> 1

Char. 788: 0 --> 1

Char. 793: 1 --> 0

Char. 803: 1 --> 3

Char. 818: 1 --> 0

Char. 839: 1 --> 2

Char. 840: 1 --> 0

Char. 853: 1 --> 0

Char. 861: 1 --> 0

Char. 862: 1 --> 2

Char. 886: 2 --> 1

Char. 888: 0 --> 1

Char. 911: 0 --> 1

Char. 912: 0 --> 1

Char. 918: 0 --> 1

Char. 927: 0 --> 1

Allosaurus :

Char. 21: 1 --> 0

Char. 80: 0 --> 1

Char. 90: 0 --> 1

Char. 91: 1 --> 2

Char. 96: 2 --> 3

Char. 113: 1 --> 0

Char. 146: 0 --> 1

Char. 154: 0 --> 1

Char. 229: 1 --> 0

Char. 238: 0 --> 2

Char. 251: 0 --> 1

Char. 254: 1 --> 0

Char. 261: 0 --> 1

Char. 278: 0 --> 1

Char. 316: 1 --> 2

Char. 327: 0 --> 1

Char. 335: 1 --> 0

Char. 351: 0 --> 1

Char. 395: 1 --> 2

Char. 402: 0 --> 2

Char. 420: 01 --> 2

Char. 493: 1 --> 0

Char. 494: 1 --> 0

Char. 505: 1 --> 0

Char. 516: 1 --> 0

Char. 540: 0 --> 1

Char. 549: 1 --> 0

Char. 552: 1 --> 0

Char. 558: 0 --> 1

Char. 637: 1 --> 0

Char. 677: 0 --> 1

Char. 713: 1 --> 0

Char. 808: 2 --> 1

Char. 876: 1 --> 0

Alvarezsaurus_calvoi :

Char. 498: 1 --> 0

Char. 914: 3 --> 2

Char. 940: 0 --> 1

Char. 944: 0 --> 1

Alxasaurus_elesitaiensis :

Char. 436: 1 --> 0

Char. 613: 0 --> 2

Char. 735: 0 --> 1

Char. 748: 0 --> 1

Char. 853: 1 --> 2

Char. 960: 1 --> 0

Anchiornis_huxleyi :

Char. 57: 0 --> 1

Char. 69: 0 --> 2

Char. 91: 1 --> 0

Char. 145: 0 --> 1

Char. 201: 1 --> 0

Char. 254: 0 --> 1

Char. 362: 0 --> 1

Char. 761: 1 --> 2

Char. 816: 1 --> 0

Char. 924: 0 --> 1

Anserimimus_planinychus :

Char. 653: 1 --> 0

Char. 667: 0 --> 1

Char. 734: 2 --> 1

Char. 740: 0 --> 1

Char. 767: 1 --> 0

Char. 830: 2 --> 0

Char. 835: 1 --> 0

Char. 851: 1 --> 2

Char. 917: 0 --> 1

Anzu_wyliei :

Char. 496: 0 --> 1

Char. 529: 0 --> 1

Char. 539: 0 --> 1

Char. 543: 0 --> 1

Char. 587: 1 --> 0

Char. 610: 0 --> 1

Char. 819: 0 --> 1

Char. 906: 0 --> 1

Apsaravis_ukhaana :

Char. 342: 0 --> 1

Char. 343: 0 --> 1

Char. 571: 1 --> 0

Char. 587: 1 --> 0

Char. 659: 0 --> 1

Char. 773: 1 --> 0

Char. 819: 1 --> 0

Char. 829: 1 --> 0

Char. 848: 1 --> 0

Char. 860: 0 --> 1

Char. 954: 0 --> 1

Char. 955: 0 --> 1

Archaeopteryx :

Char. 18: 0 --> 1

Char. 76: 2 --> 1

Char. 98: 2 --> 1

Char. 104: 0 --> 1

Char. 108: 1 --> 0

Char. 120: 1 --> 0

Char. 160: 0 --> 1

Char. 170: 1 --> 0

Char. 196: 0 --> 1

Char. 224: 0 --> 1

Char. 247: 0 --> 1

Char. 253: 1 --> 0

Char. 287: 1 --> 0

Char. 290: 0 --> 1

Char. 325: 0 --> 1

Char. 330: 2 --> 0

Char. 402: 0 --> 1

Char. 444: 1 --> 0

Char. 675: 1 --> 0

Char. 820: 0 --> 2

Char. 830: 0 --> 3

Char. 836: 1 --> 2

Char. 899: 1 --> 0

Char. 913: 1 --> 0

Char. 963: 0 --> 1

Archaeornithomimus :

Char. 524: 0 --> 1

Char. 526: 0 --> 1

Char. 549: 2 --> 1

Char. 574: 3 --> 2

Char. 601: 0 --> 1

Char. 678: 0 --> 1

Char. 680: 0 --> 1

Char. 727: 0 --> 1

Char. 748: 1 --> 2

Char. 759: 1 --> 0

Char. 761: 0 --> 2

Char. 770: 0 --> 1

Char. 791: 1 --> 0

Char. 801: 0 --> 1

Char. 802: 0 --> 1

Char. 803: 1 --> 0

Char. 806: 1 --> 0

Char. 822: 1 --> 0

Char. 829: 0 --> 1

Char. 835: 1 --> 0

Char. 883: 1 --> 0

Char. 897: 2 --> 3

Char. 911: 0 --> 1

Char. 918: 1 --> 0

Char. 939: 0 --> 1

Asfaltovenator_vialidadi :

Char. 0: 1 --> 0

Char. 3: 1 --> 2

Char. 28: 0 --> 1

Char. 62: 1 --> 0

Char. 67: 0 --> 1

Char. 76: 1 --> 0

Char. 85: 0 --> 1

Char. 152: 0 --> 1

Char. 159: 0 --> 1

Char. 172: 0 --> 1

Char. 182: 0 --> 1

Char. 205: 1 --> 0

Char. 226: 1 --> 0

Char. 235: 0 --> 1

Char. 257: 1 --> 0

Char. 263: 0 --> 1

Char. 277: 0 --> 1

Char. 331: 0 --> 1

Char. 333: 0 --> 1

Char. 357: 1 --> 0

Char. 363: 0 --> 1

Char. 373: 1 --> 0

Char. 375: 0 --> 1

Char. 385: 1 --> 0

Char. 387: 1 --> 0

Char. 392: 0 --> 1

Char. 457: 1 --> 0

Char. 465: 1 --> 0

Char. 481: 2 --> 1

Char. 541: 1 --> 0

Char. 553: 0 --> 1

Char. 564: 0 --> 1

Char. 567: 0 --> 1

Char. 636: 2 --> 1

Char. 642: 1 --> 0

Char. 643: 0 --> 1

Char. 648: 0 --> 1

Char. 652: 1 --> 0

Char. 664: 0 --> 1

Char. 737: 0 --> 1

Char. 861: 1 --> 0

Char. 862: 1 --> 0

Char. 888: 0 --> 1

Char. 906: 1 --> 0

Aucasaurus_garridoi :

Char. 860: 0 --> 1

Char. 909: 0 --> 1

Australovenator_wintonensis :

Char. 726: 2 --> 1

Char. 729: 1 --> 0

Char. 734: 1 --> 0

Char. 735: 0 --> 1

Austroraptor_cabazai :

Char. 63: 1 --> 0

Char. 70: 0 --> 1

Char. 203: 0 --> 1

Char. 337: 0 --> 1

Char. 419: 1 --> 0

Char. 560: 0 --> 1

Char. 948: 1 --> 0

Char. 952: 0 --> 1

Char. 953: 0 --> 1

Char. 954: 0 --> 1

Char. 964: 0 --> 1

Avimimus :

Char. 10: 1 --> 2

Char. 15: 2 --> 1

Char. 33: 0 --> 2

Char. 179: 1 --> 0

Char. 228: 0 --> 1

Char. 232: 1 --> 0

Char. 255: 0 --> 1

Char. 298: 0 --> 1

Char. 300: 0 --> 1

Char. 330: 2 --> 0

Char. 346: 1 --> 0

Char. 496: 0 --> 1

Char. 498: 1 --> 0

Char. 513: 1 --> 2

Char. 539: 0 --> 1

Char. 545: 0 --> 1

Char. 565: 1 --> 0

Char. 637: 1 --> 0

Char. 685: 0 --> 1

Char. 699: 0 --> 1

Char. 749: 0 --> 1

Char. 750: 0 --> 1

Char. 752: 0 --> 1

Char. 778: 1 --> 0

Char. 803: 1 --> 0

Char. 836: 1 --> 0

Char. 839: 1 --> 2

Char. 855: 2 --> 1

Char. 883: 1 --> 0

Char. 900: 1 --> 2

Char. 906: 0 --> 1

Char. 925: 0 --> 1

Char. 926: 0 --> 1

Char. 936: 01 --> 2

Char. 949: 1 --> 0

Baryonyx_walkeri :

Char. 203: 0 --> 1

Char. 241: 1 --> 0

Char. 249: 0 --> 1

Char. 262: 0 --> 1

Char. 337: 1 --> 0

Char. 398: 0 --> 1

Char. 471: 0 --> 1

Char. 473: 1 --> 0

Char. 487: 0 --> 1

Char. 516: 1 --> 0

Char. 540: 0 --> 1

Char. 548: 0 --> 2

Char. 667: 1 --> 2

Char. 690: 0 --> 1

Char. 861: 0 --> 1

Beipiaosaurus_inexpectus :

Char. 47: 1 --> 2

Char. 56: 1 --> 0

Char. 330: 0 --> 1

Char. 637: 1 --> 0

Char. 664: 0 --> 12

Char. 716: 0 --> 1

Char. 853: 1 --> 2

Berthasaura_leopoldinae :

Char. 17: 1 --> 0

Char. 26: 0 --> 1

Char. 31: 0 --> 1

Char. 34: 0 --> 1

Char. 94: 0 --> 1

Char. 116: 0 --> 1

Char. 155: 1 --> 2

Char. 170: 0 --> 1

Char. 214: 1 --> 0

Char. 339: 0 --> 1

Char. 513: 2 --> 01

Char. 515: 0 --> 1

Char. 536: 1 --> 0

Char. 577: 1 --> 0

Char. 578: 0 --> 1

Char. 593: 0 --> 2

Char. 672: 2 --> 01

Char. 676: 0 --> 1

Char. 678: 0 --> 1

Char. 680: 0 --> 1

Char. 758: 1 --> 0

Char. 761: 0 --> 12

Char. 764: 1 --> 2

Char. 777: 0 --> 1

Char. 778: 0 --> 1

Char. 791: 0 --> 1

Char. 809: 1 --> 0

Char. 835: 0 --> 2

Char. 851: 0 --> 1

Char. 901: 0 --> 1

Bicentenaria_argentina :

Char. 28: 0 --> 1

Char. 178: 1 --> 0

Char. 276: 1 --> 0

Char. 404: 2 --> 1

Char. 536: 0 --> 1

Char. 539: 0 --> 1

Char. 564: 0 --> 1

Char. 565: 1 --> 0

Char. 580: 1 --> 0

Char. 603: 0 --> 1

Char. 614: 1 --> 0

Char. 675: 1 --> 0

Char. 694: 0 --> 1

Char. 840: 1 --> 0

Char. 842: 0 --> 1

Char. 848: 0 --> 12

Char. 849: 0 --> 1

Char. 883: 1 --> 0

Char. 911: 0 --> 1

Char. 915: 0 --> 1

Char. 917: 0 --> 1

Char. 919: 1 --> 0

Bistahieversor_sealeyi :

Char. 8: 0 --> 1

Char. 72: 1 --> 0

Char. 90: 0 --> 1

Char. 148: 1 --> 0

Char. 153: 0 --> 1

Char. 196: 0 --> 1

Char. 361: 0 --> 1

Char. 422: 3 --> 2

Buitreraptor_gonzalezorum :

Char. 208: 0 --> 1

Char. 499: 0 --> 1

Char. 506: 1 --> 0

Char. 512: 1 --> 0

Char. 655: 1 --> 2

Char. 803: 1 --> 0

Char. 936: 0 --> 1

Byronosaurus_jaffei :

Char. 30: 0 --> 1

Char. 38: 0 --> 2

Char. 47: 1 --> 2

Char. 62: 1 --> 0

Char. 76: 2 --> 1

Char. 84: 1 --> 0

Char. 100: 1 --> 0

Char. 254: 0 --> 1

Char. 287: 1 --> 0

Char. 327: 1 --> 0

Char. 334: 0 --> 1

Char. 414: 0 --> 1

Char. 852: 1 --> 2

Carcharodontosaurus :

Char. 109: 1 --> 0

Char. 116: 0 --> 1

Char. 134: 0 --> 1

Char. 145: 0 --> 1

Char. 220: 0 --> 1

Char. 222: 1 --> 0

Char. 250: 1 --> 0

Char. 292: 1 --> 0

Carnotaurus_sastrei :

Char. 1: 1 --> 0

Char. 8: 0 --> 1

Char. 10: 0 --> 1

Char. 13: 1 --> 0

Char. 23: 1 --> 0

Char. 47: 1 --> 0

Char. 90: 0 --> 1

Char. 134: 0 --> 1

Char. 153: 0 --> 2

Char. 155: 0 --> 1

Char. 158: 0 --> 1

Char. 175: 0 --> 1

Char. 185: 1 --> 0

Char. 229: 0 --> 1

Char. 247: 0 --> 1

Char. 350: 0 --> 1

Char. 399: 1 --> 0

Char. 409: 2 --> 1

Char. 519: 0 --> 1

Char. 541: 0 --> 2

Char. 595: 0 --> 1

Char. 761: 0 --> 1

Char. 768: 0 --> 1

Caudipteryx :

Char. 15: 2 --> 3

Char. 38: 0 --> 2

Char. 63: 1 --> 0

Char. 187: 0 --> 1

Char. 334: 0 --> 1

Char. 353: 0 --> 2

Char. 397: 0 --> 1

Char. 401: 0 --> 1

Char. 404: 2 --> 1

Char. 585: 3 --> 2

Char. 646: 12 --> 0

Char. 707: 1 --> 0

Char. 717: 0 --> 1

Char. 849: 0 --> 1

Char. 851: 1 --> 2

Char. 942: 0 --> 1

Ceratosaurus :

Char. 84: 1 --> 0

Char. 96: 1 --> 3

Char. 110: 2 --> 1

Char. 118: 1 --> 0

Char. 123: 1 --> 0

Char. 159: 0 --> 1

Char. 198: 1 --> 2

Char. 247: 0 --> 1

Char. 487: 1 --> 0

Char. 494: 1 --> 0

Char. 573: 0 --> 1

Char. 584: 0 --> 1

Char. 654: 0 --> 1

Char. 715: 0 --> 1

Char. 801: 1 --> 0

Char. 842: 0 --> 1

Char. 844: 0 --> 1

Char. 852: 2 --> 1

Char. 862: 1 --> 2

Char. 863: 1 --> 0

Char. 882: 0 --> 1

Char. 904: 1 --> 2

Char. 927: 0 --> 1

Char. 957: 1 --> 0

Citipati_osmolskae :

Char. 9: 1 --> 2

Char. 10: 1 --> 0

Char. 15: 2 --> 1

Char. 20: 1 --> 0

Char. 22: 1 --> 0

Char. 29: 1 --> 0

Char. 37: 1 --> 0

Char. 90: 0 --> 1

Char. 154: 0 --> 1

Char. 160: 0 --> 1

Char. 190: 0 --> 1

Char. 199: 0 --> 1

Char. 230: 0 --> 1

Char. 364: 1 --> 0

Char. 629: 0 --> 1

Coelophysis_bauri :

Char. 3: 0 --> 1

Char. 29: 1 --> 0

Char. 35: 1 --> 0

Char. 45: 2 --> 3

Char. 208: 0 --> 1

Char. 273: 1 --> 0

Char. 347: 0 --> 1

Char. 409: 2 --> 3

Char. 457: 0 --> 1

Char. 479: 2 --> 0

Char. 661: 1 --> 0

Char. 682: 0 --> 1

Char. 750: 1 --> 0

Megapnosaurus_rhodesiensis :

Char. 512: 1 --> 0

Char. 536: 1 --> 0

Char. 788: 0 --> 1

"Syntarsus"_kayentakatae :

Char. 79: 0 --> 1

Char. 112: 0 --> 1

Char. 124: 1 --> 0

Char. 139: 1 --> 0

Char. 140: 0 --> 1

Char. 173: 0 --> 1

Char. 198: 0 --> 1

Char. 199: 0 --> 1

Char. 257: 0 --> 1

Char. 389: 1 --> 2

Char. 412: 0 --> 1

Char. 492: 0 --> 1

Char. 513: 2 --> 1

Char. 577: 0 --> 1

Char. 853: 0 --> 1

Char. 872: 0 --> 1

Char. 926: 0 --> 1

Segisaurus_halli :

Char. 628: 0 --> 1

Char. 636: 1 --> 2

Char. 637: 0 --> 1

Char. 646: 0 --> 1

Char. 673: 0 --> 1

Char. 828: 0 --> 1

Char. 861: 0 --> 1

Char. 903: 1 --> 0

Coelurus_fragilis :

Char. 505: 0 --> 1

Char. 506: 1 --> 0

Char. 536: 0 --> 1

Char. 598: 1 --> 0

Char. 599: 1 --> 0

Char. 606: 0 --> 1

Char. 670: 0 --> 1

Char. 679: 1 --> 2

Char. 680: 0 --> 1

Char. 716: 1 --> 0

Char. 803: 1 --> 0

Char. 814: 0 --> 1

Char. 840: 1 --> 0

Char. 855: 1 --> 0

Char. 870: 0 --> 1

Char. 903: 1 --> 0

Char. 915: 0 --> 1

Compsognathus_longipes :

Char. 15: 1 --> 3

Char. 16: 1 --> 0

Char. 30: 0 --> 1

Char. 76: 1 --> 0

Char. 126: 1 --> 0

Char. 335: 0 --> 1

Char. 395: 1 --> 0

Char. 409: 1 --> 2

Char. 417: 12 --> 3

Char. 422: 3 --> 2

Char. 425: 2 --> 1

Char. 498: 1 --> 0

Char. 505: 0 --> 1

Char. 541: 1 --> 0

Char. 549: 1 --> 2

Char. 599: 1 --> 0

Char. 618: 1 --> 0

Char. 658: 0 --> 1

Char. 707: 1 --> 0

Char. 777: 1 --> 0

Char. 780: 1 --> 0

Char. 808: 2 --> 0

Char. 815: 0 --> 1

Char. 911: 0 --> 1

Char. 912: 0 --> 1

Concavenator_corcovatus :

Char. 127: 2 --> 1

Char. 561: 0 --> 1

Char. 562: 0 --> 1

Char. 788: 0 --> 1

Conchoraptor_gracilis :

Char. 261: 0 --> 1

Char. 457: 1 --> 0

Char. 529: 0 --> 1

Char. 562: 0 --> 1

Char. 744: 1 --> 0

Char. 748: 0 --> 1

Char. 764: 1 --> 2

Char. 951: 0 --> 1

Condorraptor_currumili :

Char. 498: 1 --> 0

Char. 540: 0 --> 1

Char. 546: 0 --> 1

Char. 567: 0 --> 1

Char. 788: 0 --> 1

Confuciusornis_sanctus :

Char. 0: 1 --> 2

Char. 9: 0 --> 1

Char. 19: 1 --> 2

Char. 124: 1 --> 0

Char. 322: 1 --> 2

Char. 334: 0 --> 1

Char. 355: 0 --> 1

Char. 553: 0 --> 1

Char. 631: 0 --> 1

Char. 739: 0 --> 1

Char. 759: 1 --> 2

Char. 918: 1 --> 0

Char. 927: 0 --> 1

Char. 934: 0 --> 1

Cryolophosaurus_ellioti :

Char. 96: 1 --> 3

Char. 105: 0 --> 1

Char. 109: 0 --> 1

Char. 155: 1 --> 2

Char. 165: 0 --> 1

Char. 194: 0 --> 1

Char. 205: 1 --> 0

Char. 219: 0 --> 1

Char. 223: 0 --> 1

Char. 226: 1 --> 0

Char. 262: 0 --> 1

Char. 270: 1 --> 0

Char. 290: 2 --> 0

Char. 487: 1 --> 2

Char. 550: 0 --> 2

Char. 565: 0 --> 1

Char. 615: 1 --> 0

Char. 664: 0 --> 1

Char. 678: 0 --> 1

Char. 852: 2 --> 1

Char. 873: 0 --> 1

Char. 877: 1 --> 0

Daspletosaurus :

Char. 72: 1 --> 0

Char. 76: 2 --> 1

Char. 142: 0 --> 1

Char. 154: 1 --> 0

Char. 191: 0 --> 1

Char. 196: 0 --> 1

Char. 221: 1 --> 0

Char. 353: 2 --> 1

Char. 363: 1 --> 2

Char. 409: 1 --> 2

Char. 454: 1 --> 0

Char. 463: 0 --> 1

Char. 617: 0 --> 1

Char. 659: 0 --> 1

Char. 837: 0 --> 1

Char. 858: 0 --> 1

Char. 953: 0 --> 1

Char. 955: 1 --> 2

Deinocheirus :

Char. 18: 0 --> 1

Char. 21: 0 --> 1

Char. 30: 0 --> 1

Char. 94: 0 --> 1

Char. 95: 1 --> 0

Char. 99: 1 --> 0

Char. 108: 1 --> 0

Char. 186: 0 --> 1

Char. 327: 1 --> 2

Char. 381: 0 --> 1

Char. 481: 0 --> 1

Char. 499: 0 --> 1

Char. 506: 0 --> 1

Char. 509: 0 --> 2

Char. 536: 0 --> 2

Char. 555: 1 --> 2

Char. 557: 0 --> 2

Char. 559: 0 --> 1

Char. 598: 1 --> 0

Char. 599: 1 --> 0

Char. 604: 0 --> 1

Char. 631: 0 --> 1

Char. 673: 1 --> 0

Char. 678: 0 --> 1

Char. 693: 2 --> 01

Char. 718: 1 --> 0

Char. 736: 1 --> 0

Char. 737: 1 --> 0

Char. 738: 1 --> 0

Char. 747: 1 --> 0

Char. 748: 1 --> 0

Char. 757: 0 --> 1

Char. 759: 1 --> 0

Char. 766: 0 --> 1

Char. 803: 1 --> 2

Char. 840: 1 --> 0

Char. 841: 0 --> 1

Char. 917: 0 --> 1

Char. 950: 0 --> 1

Char. 951: 0 --> 1

Char. 954: 1 --> 2

Deinonychus_antirrhopus :

Char. 22: 1 --> 0

Char. 65: 1 --> 2

Char. 99: 1 --> 0

Char. 120: 1 --> 0

Char. 127: 1 --> 2

Char. 186: 1 --> 0

Char. 357: 0 --> 1

Char. 409: 1 --> 2

Char. 419: 1 --> 0

Char. 510: 1 --> 0

Char. 540: 1 --> 0

Char. 708: 0 --> 1

Char. 779: 1 --> 0

Char. 913: 1 --> 0

Char. 946: 2 --> 1

Char. 954: 0 --> 1

Deltadromeus_agilis :

Char. 660: 0 --> 1

Char. 848: 0 --> 1

Char. 854: 1 --> 2

Char. 860: 0 --> 1

Char. 862: 0 --> 1

Char. 874: 0 --> 1

Char. 884: 1 --> 2

Char. 886: 2 --> 1

Char. 900: 1 --> 2

Char. 955: 2 --> 1

Dilong_paradoxus :

Char. 20: 1 --> 0

Char. 52: 0 --> 1

Char. 63: 1 --> 0

Char. 71: 1 --> 0

Char. 96: 0 --> 1

Char. 103: 0 --> 1

Char. 159: 0 --> 1

Char. 182: 0 --> 1

Char. 184: 0 --> 1

Char. 187: 0 --> 1

Char. 192: 0 --> 1

Char. 215: 0 --> 2

Char. 276: 1 --> 0

Char. 380: 0 --> 1

Char. 411: 0 --> 12

Char. 424: 1 --> 0

Char. 494: 1 --> 0

Char. 635: 0 --> 1

Char. 636: 2 --> 1

Char. 672: 1 --> 2

Char. 688: 1 --> 0

Char. 761: 2 --> 1

Char. 763: 1 --> 0

Char. 775: 1 --> 0

Char. 780: 1 --> 0

Char. 838: 0 --> 1

Char. 843: 1 --> 0

Char. 855: 1 --> 0

Char. 879: 1 --> 0

Sinosaurus_triassicus :

Char. 0: 1 --> 2

Char. 15: 2 --> 1

Char. 23: 1 --> 0

Char. 94: 0 --> 1

Char. 160: 1 --> 0

Char. 218: 0 --> 1

Char. 222: 0 --> 1

Char. 248: 1 --> 0

Char. 269: 1 --> 4

Char. 278: 0 --> 1

Char. 283: 0 --> 1

Char. 363: 0 --> 1

Char. 602: 0 --> 1

Char. 650: 0 --> 1

Char. 652: 1 --> 0

Char. 655: 1 --> 0

Char. 760: 0 --> 1

Char. 803: 1 --> 0

Char. 830: 0 --> 1

Char. 853: 0 --> 1

Char. 887: 0 --> 1

Char. 889: 0 --> 1

Char. 944: 0 --> 1

Char. 955: 1 --> 2

Dilophosaurus_wetherilli :

Char. 20: 1 --> 0

Char. 23: 1 --> 2

Char. 30: 0 --> 1

Char. 79: 0 --> 1

Char. 80: 0 --> 1

Char. 83: 0 --> 1

Char. 98: 0 --> 12

Char. 110: 0 --> 2

Char. 119: 0 --> 1

Char. 161: 0 --> 1

Char. 165: 0 --> 1

Char. 166: 0 --> 1

Char. 194: 0 --> 1

Char. 195: 0 --> 1

Char. 207: 0 --> 1

Char. 252: 0 --> 1

Char. 256: 0 --> 1

Char. 257: 0 --> 1

Char. 340: 0 --> 1

Char. 399: 1 --> 2

Char. 400: 1 --> 2

Char. 442: 0 --> 1

Char. 457: 0 --> 1

Char. 561: 0 --> 1

Char. 567: 0 --> 1

Char. 640: 02 --> 1

Char. 642: 1 --> 0

Char. 661: 1 --> 0

Char. 881: 1 --> 0

Char. 920: 0 --> 1

Dromaeosaurus_albertensis :

Char. 114: 0 --> 1

Char. 127: 1 --> 2

Char. 231: 0 --> 1

Char. 256: 0 --> 1

Char. 268: 4 --> 0

Char. 270: 1 --> 0

Char. 321: 0 --> 1

Char. 377: 1 --> 0

Char. 402: 0 --> 1

Char. 409: 1 --> 0

Char. 425: 1 --> 0

Char. 438: 1 --> 0

Dubreuillosaurus_valesdunensis :

Char. 54: 0 --> 1

Char. 67: 0 --> 1

Char. 112: 1 --> 0

Char. 203: 0 --> 1

Char. 252: 0 --> 1

Char. 291: 0 --> 1

Char. 295: 0 --> 1

Char. 606: 0 --> 1

Char. 608: 0 --> 1

Char. 637: 1 --> 0

Char. 839: 1 --> 0

Char. 844: 1 --> 0

Char. 868: 1 --> 0

Ekrixinatosaurus_novasi :

Char. 47: 1 --> 0

Char. 212: 0 --> 1

Char. 241: 1 --> 2

Char. 242: 1 --> 0

Char. 795: 1 --> 0

Char. 854: 1 --> 0

Char. 856: 0 --> 1

Char. 874: 1 --> 0

Char. 915: 2 --> 1

Elaphrosaurus_bambergi :

Char. 485: 1 --> 0

Char. 487: 12 --> 0

Char. 489: 1 --> 0

Char. 505: 1 --> 0

Char. 523: 0 --> 1

Char. 576: 0 --> 1

Char. 599: 1 --> 0

Char. 609: 2 --> 3

Char. 650: 0 --> 1

Char. 653: 0 --> 1

Char. 654: 0 --> 1

Char. 661: 0 --> 1

Char. 750: 0 --> 1

Char. 757: 0 --> 1

Char. 781: 0 --> 1

Char. 796: 0 --> 1

Char. 854: 0 --> 1

Char. 868: 0 --> 1

Char. 878: 1 --> 0

Char. 884: 1 --> 2

Char. 921: 1 --> 0

Char. 937: 0 --> 2

Char. 954: 1 --> 0

Eoabelisaurus_mefi :

Char. 93: 1 --> 0

Char. 96: 1 --> 0

Char. 126: 0 --> 1

Char. 153: 1 --> 0

Char. 179: 0 --> 1

Char. 212: 0 --> 1

Char. 213: 0 --> 1

Char. 238: 0 --> 1

Char. 242: 1 --> 0

Char. 252: 0 --> 1

Char. 276: 1 --> 0

Char. 284: 0 --> 1

Char. 514: 1 --> 0

Char. 592: 2 --> 0

Char. 595: 0 --> 1

Char. 640: 0 --> 1

Char. 683: 1 --> 2

Char. 695: 0 --> 1

Char. 768: 0 --> 1

Char. 799: 0 --> 1

Char. 809: 1 --> 0

Char. 843: 1 --> 0

Char. 851: 0 --> 1

Char. 853: 0 --> 1

Char. 868: 0 --> 1

Char. 895: 0 --> 1

Char. 896: 0 --> 1

Eotyrannus_lengi :

Char. 187: 0 --> 1

Char. 422: 3 --> 2

Char. 480: 1 --> 0

Char. 487: 0 --> 12

Char. 549: 1 --> 2

Char. 651: 0 --> 1

Char. 675: 0 --> 1

Char. 707: 1 --> 0

Char. 726: 2 --> 1

Char. 888: 0 --> 1

Erlikosaurus_andrewsi :

Char. 230: 0 --> 1

Char. 231: 0 --> 1

Char. 234: 1 --> 0

Char. 270: 1 --> 0

Char. 285: 1 --> 0

Char. 340: 0 --> 1

Char. 342: 1 --> 0

Char. 949: 1 --> 0

Eustreptospondylus_oxoniensis :

Char. 29: 0 --> 1

Char. 58: 2 --> 0

Char. 61: 2 --> 1

Char. 82: 1 --> 0

Char. 108: 1 --> 0

Char. 113: 1 --> 0

Char. 160: 1 --> 0

Char. 248: 1 --> 0

Char. 254: 1 --> 0

Char. 261: 0 --> 1

Char. 263: 0 --> 1

Char. 293: 0 --> 1

Char. 541: 1 --> 0

Char. 542: 0 --> 1

Falcarius_utahensis :

Char. 178: 1 --> 0

Char. 182: 0 --> 1

Char. 339: 0 --> 1

Char. 340: 0 --> 1

Char. 486: 0 --> 1

Char. 534: 0 --> 1

Char. 536: 0 --> 2

Char. 549: 1 --> 2

Char. 557: 0 --> 1

Char. 560: 0 --> 1

Char. 598: 1 --> 0

Char. 617: 0 --> 1

Char. 620: 0 --> 1

Char. 632: 0 --> 1

Char. 656: 0 --> 1

Char. 670: 0 --> 1

Char. 672: 1 --> 0

Char. 680: 0 --> 1

Char. 735: 0 --> 1

Char. 764: 1 --> 0

Char. 774: 0 --> 1

Char. 813: 0 --> 1

Char. 815: 0 --> 1

Char. 816: 1 --> 0

Char. 874: 0 --> 1

Char. 893: 0 --> 1

Char. 949: 1 --> 2

Char. 959: 1 --> 2

Fukuiraptor_kitadaniensis :

No autapomorphies

Fukuivenator_paradoxus :

Char. 51: 0 --> 1

Char. 63: 1 --> 0

Char. 68: 0 --> 1

Char. 70: 0 --> 1

Char. 93: 1 --> 0

Char. 104: 0 --> 1

Char. 108: 1 --> 0

Char. 177: 0 --> 1

Char. 209: 0 --> 1

Char. 238: 0 --> 1

Char. 271: 0 --> 1

Char. 420: 1 --> 2

Char. 427: 0 --> 2

Char. 445: 0 --> 1

Char. 461: 1 --> 0

Char. 464: 1 --> 0

Char. 478: 1 --> 2

Char. 486: 0 --> 1

Char. 488: 0 --> 1

Char. 496: 0 --> 1

Char. 506: 1 --> 2

Char. 531: 0 --> 1

Char. 543: 0 --> 1

Char. 549: 1 --> 2

Char. 567: 0 --> 1

Char. 577: 0 --> 1

Char. 578: 0 --> 2

Char. 588: 0 --> 1

Char. 590: 0 --> 1

Char. 599: 1 --> 2

Char. 606: 0 --> 1

Char. 610: 0 --> 1

Char. 635: 0 --> 1

Char. 636: 2 --> 1

Char. 657: 0 --> 1

Char. 670: 0 --> 1

Char. 671: 0 --> 1

Char. 680: 0 --> 1

Char. 710: 0 --> 1

Char. 734: 1 --> 2

Char. 806: 1 --> 0

Char. 848: 0 --> 2

Char. 893: 0 --> 1

Char. 917: 0 --> 1

Char. 958: 0 --> 1

Gallimimus_bullatus :

Char. 15: 2 --> 3

Char. 29: 0 --> 2

Char. 94: 0 --> 1

Char. 103: 0 --> 1

Char. 114: 0 --> 1

Char. 179: 1 --> 0

Char. 230: 0 --> 1

Char. 234: 0 --> 1

Char. 381: 0 --> 1

Char. 534: 0 --> 1

Char. 541: 1 --> 0

Char. 575: 0 --> 12

Char. 646: 1 --> 0

Char. 653: 1 --> 0

Char. 689: 0 --> 1

Char. 748: 1 --> 0

Char. 762: 1 --> 0

Char. 767: 1 --> 0

Gallus :

Char. 325: 0 --> 1

Char. 553: 0 --> 1

Char. 571: 1 --> 2

Char. 591: 1 --> 0

Char. 657: 0 --> 1

Char. 661: 0 --> 2

Char. 670: 0 --> 1

Char. 681: 1 --> 0

Char. 683: 0 --> 1

Char. 751: 0 --> 1

Char. 769: 1 --> 2

Char. 783: 0 --> 1

Char. 828: 0 --> 1

Char. 833: 0 --> 1

Char. 924: 0 --> 1

Char. 936: 0 --> 2

Char. 939: 1 --> 0

Char. 941: 0 --> 1

Char. 942: 0 --> 1

Garudimimus_brevipes :

Char. 20: 1 --> 0

Char. 65: 0 --> 2

Char. 158: 1 --> 0

Char. 159: 0 --> 1

Char. 255: 0 --> 1

Char. 256: 0 --> 2

Char. 322: 1 --> 0

Char. 333: 0 --> 1

Char. 334: 0 --> 1

Char. 360: 1 --> 0

Char. 457: 1 --> 0

Char. 598: 1 --> 0

Char. 760: 0 --> 1

Char. 806: 1 --> 0

Char. 854: 1 --> 2

Char. 860: 0 --> 1

Char. 868: 0 --> 1

Char. 937: 0 --> 2

Char. 940: 0 --> 1

Giganotosaurus_carolinii :

Char. 113: 1 --> 0

Char. 338: 0 --> 1

Char. 467: 1 --> 0

Char. 479: 3 --> 2

Char. 552: 1 --> 0

Char. 589: 1 --> 0

Char. 599: 1 --> 0

Char. 610: 1 --> 0

Char. 639: 0 --> 1

Char. 767: 0 --> 1

Char. 853: 1 --> 0

Char. 856: 0 --> 1

Char. 873: 0 --> 1

Gigantoraptor_erlianensis :

Char. 330: 2 --> 0

Char. 345: 2 --> 1

Char. 587: 1 --> 0

Char. 657: 0 --> 1

Char. 852: 1 --> 2

Char. 867: 0 --> 1

Char. 868: 0 --> 1

Gnathovorax_cabreirai :

Char. 22: 0 --> 1

Char. 96: 0 --> 1

Char. 101: 0 --> 1

Char. 108: 1 --> 0

Char. 136: 0 --> 1

Char. 158: 0 --> 1

Char. 241: 0 --> 2

Char. 275: 0 --> 1

Char. 309: 0 --> 1

Char. 376: 1 --> 0

Char. 395: 1 --> 0

Char. 407: 0 --> 1

Char. 425: 0 --> 1

Char. 430: 0 --> 1

Char. 586: 0 --> 1

Char. 605: 0 --> 1

Char. 789: 1 --> 0

Char. 877: 0 --> 1

Char. 895: 0 --> 1

Tawa_hallae :

Char. 2: 1 --> 0

Char. 3: 0 --> 1

Char. 10: 0 --> 2

Char. 11: 0 --> 2

Char. 23: 0 --> 1

Char. 29: 0 --> 1

Char. 30: 0 --> 1

Char. 36: 0 --> 1

Char. 43: 0 --> 1

Char. 80: 0 --> 1

Char. 116: 0 --> 1

Char. 128: 1 --> 0

Char. 170: 0 --> 1

Char. 231: 0 --> 1

Char. 232: 1 --> 0

Char. 290: 1 --> 2

Char. 342: 1 --> 0

Char. 360: 1 --> 0

Char. 409: 2 --> 1

Char. 505: 0 --> 1

Char. 636: 1 --> 2

Char. 683: 1 --> 0

Char. 700: 0 --> 1

Char. 703: 1 --> 0

Char. 713: 1 --> 0

Char. 803: 1 --> 0

Char. 848: 2 --> 0

Char. 868: 0 --> 1

Char. 920: 0 --> 1

Herrerasaurus_ischigualastensis :

Char. 17: 0 --> 1

Char. 117: 1 --> 0

Char. 247: 0 --> 1

Char. 343: 0 --> 1

Char. 416: 0 --> 1

Char. 441: 0 --> 1

Char. 797: 0 --> 1

Char. 828: 0 --> 1

Char. 876: 1 --> 0

Staurikosaurus_pricei :

Char. 381: 0 --> 1

Char. 385: 0 --> 1

Char. 819: 0 --> 1

Char. 883: 1 --> 0

Gobivenator :

Char. 97: 1 --> 0

Char. 98: 1 --> 2

Char. 208: 1 --> 0

Char. 214: 1 --> 0

Char. 285: 1 --> 0

Gorgosaurus_libratus :

Char. 3: 1 --> 0

Char. 13: 1 --> 0

Char. 105: 0 --> 1

Char. 197: 0 --> 1

Char. 381: 1 --> 0

Char. 400: 1 --> 2

Char. 629: 0 --> 1

Char. 764: 1 --> 2

Char. 782: 0 --> 1

Char. 831: 0 --> 1

Char. 911: 0 --> 1

Char. 954: 1 --> 0

Gualicho_shinyae :

Char. 655: 1 --> 0

Char. 666: 1 --> 2

Char. 673: 1 --> 0

Char. 806: 1 --> 0

Char. 851: 1 --> 0

Char. 861: 1 --> 0

Char. 873: 0 --> 1

Char. 882: 1 --> 0

Char. 900: 1 --> 0

Char. 903: 1 --> 0

Char. 937: 0 --> 1

Guanlong_wucaii :

Char. 1: 1 --> 2

Char. 82: 1 --> 2

Char. 85: 0 --> 1

Char. 278: 1 --> 0

Char. 400: 1 --> 2

Char. 425: 0 --> 1

Haplocheirus_sollers :

Char. 6: 0 --> 1

Char. 10: 1 --> 0

Char. 11: 1 --> 0

Char. 21: 0 --> 1

Char. 56: 1 --> 0

Char. 94: 0 --> 1

Char. 95: 1 --> 0

Char. 124: 1 --> 0

Char. 162: 0 --> 1

Char. 164: 0 --> 1

Char. 187: 0 --> 1

Char. 192: 0 --> 1

Char. 195: 0 --> 1

Char. 206: 1 --> 0

Char. 327: 1 --> 0

Char. 368: 0 --> 1

Char. 423: 12 --> 3

Char. 427: 2 --> 1

Char. 430: 0 --> 1

Char. 484: 1 --> 0

Char. 632: 0 --> 1

Char. 670: 0 --> 1

Char. 682: 0 --> 1

Char. 707: 1 --> 0

Char. 784: 1 --> 0

Char. 803: 1 --> 0

Char. 808: 2 --> 0

Char. 826: 0 --> 1

Char. 902: 1 --> 0

Harpymimus_okladnikovi :

Char. 199: 0 --> 1

Char. 334: 0 --> 1

Char. 382: 2 --> 1

Char. 433: 1 --> 0

Char. 503: 1 --> 0

Char. 562: 0 --> 1

Char. 664: 0 --> 1

Char. 667: 0 --> 1

Char. 704: 1 --> 0

Char. 721: 0 --> 1

Char. 726: 1 --> 2

Char. 746: 1 --> 0

Char. 758: 1 --> 0

Char. 762: 1 --> 0

Char. 914: 3 --> 2

Ichthyovenator_laosensis :

Char. 494: 1 --> 0

Char. 604: 0 --> 1

Incisivosaurus_gauthieri :

Char. 3: 1 --> 0

Char. 10: 1 --> 0

Char. 69: 0 --> 2

Char. 112: 0 --> 1

Char. 116: 1 --> 0

Char. 118: 1 --> 0

Char. 125: 0 --> 1

Char. 128: 0 --> 1

Char. 133: 0 --> 1

Char. 140: 1 --> 0

Char. 197: 0 --> 1

Char. 224: 0 --> 1

Char. 230: 0 --> 1

Char. 248: 2 --> 0

Char. 253: 1 --> 0

Char. 259: 0 --> 1

Char. 265: 0 --> 1

Char. 267: 1 --> 0

Char. 269: 0 --> 1

Char. 343: 1 --> 0

Irritator_challengeri :

Char. 80: 0 --> 1

Char. 187: 1 --> 0

Char. 420: 2 --> 1

Jianchangosaurus_yixianensis :

Char. 343: 1 --> 0

Char. 375: 0 --> 1

Char. 391: 2 --> 0

Char. 464: 1 --> 0

Char. 487: 1 --> 0

Char. 694: 0 --> 1

Char. 747: 0 --> 1

Char. 755: 1 --> 23

Char. 758: 1 --> 0

Char. 763: 1 --> 0

Char. 766: 1 --> 0

Char. 830: 0 --> 23

Char. 852: 2 --> 1

Char. 927: 1 --> 0

Juratyrant_langhami :

Char. 823: 1 --> 0

Char. 860: 0 --> 1

Char. 877: 0 --> 1

Char. 886: 2 --> 0

Char. 887: 0 --> 1

Char. 893: 0 --> 1

Khaan_mckennai :

Char. 95: 1 --> 2

Char. 155: 1 --> 2

Char. 164: 1 --> 0

Char. 246: 1 --> 0

Char. 529: 0 --> 1

Char. 694: 0 --> 1

Char. 748: 0 --> 1

Char. 783: 0 --> 1

Char. 917: 0 --> 1

Char. 955: 2 --> 1

Kileskus_aristotocus :

Char. 53: 0 --> 1

Char. 65: 1 --> 2

Char. 70: 0 --> 1

Char. 398: 1 --> 0

Char. 420: 1 --> 0

Char. 424: 1 --> 0

Kiyacursor_longipes :

Char. 481: 1 --> 0

Char. 652: 1 --> 0

Char. 659: 0 --> 1

Char. 886: 0 --> 2

Char. 928: 0 --> 1

Char. 957: 1 --> 0

Lajasvenator_ascheriae :

Char. 535: 0 --> 1

Char. 536: 2 --> 1

Char. 753: 0 --> 1

Leshansaurus_qianweiensis :

Char. 54: 0 --> 1

Char. 75: 0 --> 2

Char. 90: 0 --> 1

Char. 212: 0 --> 1

Char. 224: 0 --> 1

Char. 250: 1 --> 0

Char. 254: 1 --> 0

Char. 273: 0 --> 1

Char. 467: 1 --> 0

Char. 563: 0 --> 1

Char. 578: 0 --> 2

Char. 788: 0 --> 1

Char. 840: 1 --> 0

Liliensternus_liliensterni :

Char. 133: 0 --> 1

Char. 416: 0 --> 1

Notatesseraeraptor_frickensis :

Char. 75: 2 --> 1

Char. 238: 0 --> 1

Char. 252: 0 --> 1

Char. 357: 1 --> 0

Char. 366: 1 --> 0

Char. 578: 0 --> 1

Char. 683: 1 --> 0

Char. 727: 0 --> 1

Char. 777: 0 --> 1

Char. 803: 1 --> 0

Char. 832: 0 --> 1

Panguraptor_lufengensis :

Char. 10: 0 --> 1

Char. 11: 0 --> 1

Char. 44: 1 --> 0

Char. 322: 1 --> 0

Char. 487: 0 --> 1

Char. 554: 0 --> 1

Char. 655: 1 --> 0

Char. 693: 1 --> 2

Char. 848: 2 --> 1

Char. 900: 1 --> 2

Char. 908: 1 --> 0

Limusaurus_inextricabilis :

Char. 494: 1 --> 0

Char. 570: 0 --> 1

Char. 614: 0 --> 2

Char. 656: 0 --> 1

Char. 690: 0 --> 1

Char. 763: 1 --> 0

Char. 795: 0 --> 1

Char. 823: 0 --> 1

Char. 861: 1 --> 0

Char. 876: 1 --> 0

Char. 877: 1 --> 0

Char. 902: 1 --> 0

Linheraptor_exquisitus :

Char. 11: 1 --> 0

Char. 18: 0 --> 1

Char. 23: 1 --> 0

Char. 39: 0 --> 1

Char. 44: 0 --> 1

Char. 59: 1 --> 0

Char. 63: 1 --> 0

Char. 70: 0 --> 1

Char. 97: 1 --> 0

Char. 135: 1 --> 2

Char. 145: 1 --> 0

Char. 167: 0 --> 1

Char. 232: 1 --> 0

Char. 242: 0 --> 1

Char. 247: 0 --> 1

Char. 254: 0 --> 1

Char. 281: 2 --> 1

Char. 513: 1 --> 0

Char. 532: 0 --> 1

Char. 588: 1 --> 0

Char. 621: 0 --> 1

Char. 625: 0 --> 1

Char. 804: 0 --> 1

Lourinhanosaurus_antunesi :

Char. 582: 0 --> 1

Char. 777: 1 --> 0

Char. 797: 0 --> 1

Char. 856: 0 --> 1

Char. 874: 0 --> 1

Char. 881: 1 --> 0

Lucianovenator_bonoi :

Char. 504: 0 --> 1

Char. 781: 1 --> 0

Lusovenator_santosi :

Char. 795: 0 --> 1

Char. 801: 0 --> 1

Maip_macrothorax :

Char. 603: 0 --> 1

Majungasaurus_crenatissimus :

Char. 8: 0 --> 1

Char. 15: 1 --> 2

Char. 142: 0 --> 1

Char. 144: 1 --> 0

Char. 160: 1 --> 0

Char. 171: 0 --> 1

Char. 176: 0 --> 1

Char. 178: 1 --> 0

Char. 184: 0 --> 1

Char. 195: 0 --> 1

Char. 202: 0 --> 1

Char. 238: 0 --> 2

Char. 262: 0 --> 1

Char. 290: 2 --> 1

Char. 318: 0 --> 1

Char. 373: 1 --> 0

Char. 462: 0 --> 1

Char. 475: 2 --> 0

Char. 486: 0 --> 1

Char. 487: 1 --> 0

Char. 543: 0 --> 1

Char. 560: 0 --> 1

Char. 562: 1 --> 0

Char. 574: 3 --> 2

Char. 605: 1 --> 0

Char. 636: 1 --> 0

Char. 641: 0 --> 1

Char. 762: 1 --> 0

Char. 868: 0 --> 1

Char. 876: 1 --> 2

Char. 937: 2 --> 0

Char. 944: 0 --> 1

Char. 949: 1 --> 0

Char. 951: 0 --> 1

Mapusaurus_roseae :

Char. 474: 1 --> 0

Char. 478: 1 --> 0

Char. 566: 1 --> 0

Char. 827: 0 --> 1

Char. 830: 0 --> 1

Marshosaurus_bicentesimus :

Char. 481: 1 --> 2

Char. 544: 0 --> 1

Char. 781: 0 --> 1

Char. 796: 0 --> 1

Char. 801: 1 --> 0

Char. 803: 1 --> 0

Char. 818: 0 --> 1

Masiakasaurus_knopfleri :

Char. 41: 0 --> 1

Char. 70: 0 --> 1

Char. 84: 1 --> 0

Char. 430: 0 --> 1

Char. 512: 0 --> 1

Char. 513: 2 --> 1

Char. 517: 1 --> 0

Char. 944: 0 --> 1

Megalosaurus :

Char. 40: 0 --> 1

Char. 45: 12 --> 0

Char. 86: 2 --> 1

Char. 429: 0 --> 1

Char. 669: 0 --> 1

Char. 670: 0 --> 1

Char. 772: 0 --> 2

Char. 774: 0 --> 1

Char. 819: 0 --> 1

Char. 835: 1 --> 0

Char. 886: 2 --> 0

Char. 889: 0 --> 1

Char. 937: 1 --> 0

Megaraptor_namunhuaiquii :

Char. 715: 0 --> 1

Megaraptorid_mucpv_595 :

Char. 453: 1 --> 0

Char. 461: 1 --> 0

Char. 555: 1 --> 0

Char. 650: 1 --> 0

Mei_long :

Char. 0: 1 --> 0

Char. 15: 2 --> 3

Char. 32: 0 --> 1

Char. 45: 2 --> 3

Char. 95: 1 --> 2

Char. 215: 2 --> 0

Char. 334: 0 --> 1

Char. 388: 1 --> 0

Char. 391: 2 --> 1

Char. 457: 1 --> 0

Char. 469: 0 --> 1

Char. 535: 0 --> 2

Char. 753: 1 --> 0

Char. 777: 1 --> 0

Char. 803: 1 --> 0

Char. 836: 1 --> 0

Char. 912: 0 --> 1

Meraxes_gigas :

Char. 127: 2 --> 3

Char. 134: 0 --> 1

Char. 317: 0 --> 1

Char. 671: 0 --> 1

Char. 840: 1 --> 0

Char. 960: 1 --> 2

Metriacanthosaurus_parkeri :

Char. 562: 0 --> 1

Char. 777: 0 --> 1

Char. 844: 0 --> 1

Microraptor :

Char. 322: 1 --> 2

Char. 325: 0 --> 1

Char. 401: 0 --> 1

Char. 540: 1 --> 0

Char. 613: 2 --> 1

Char. 631: 0 --> 1

Char. 803: 1 --> 0

Char. 820: 1 --> 2

Char. 924: 0 --> 1

Char. 966: 0 --> 1

Microvenator_celer :

Char. 329: 1 --> 0

Char. 332: 1 --> 0

Char. 354: 1 --> 0

Char. 667: 1 --> 0

Char. 753: 1 --> 0

Char. 850: 0 --> 1

Mirischia_asymmetrica :

Char. 535: 0 --> 2

Char. 551: 1 --> 0

Char. 554: 1 --> 0

Char. 555: 1 --> 0

Char. 557: 0 --> 1

Char. 561: 0 --> 1

Char. 806: 0 --> 1

Char. 818: 1 --> 0

Char. 819: 1 --> 0

Char. 821: 1 --> 0

Char. 834: 0 --> 1

Char. 840: 1 --> 0

Char. 861: 1 --> 0

Char. 876: 1 --> 2

Monolophosaurus_jiangi :

Char. 4: 0 --> 1

Char. 25: 0 --> 1

Char. 40: 0 --> 1

Char. 88: 0 --> 1

Char. 96: 1 --> 0

Char. 104: 1 --> 0

Char. 115: 0 --> 1

Char. 129: 0 --> 1

Char. 153: 1 --> 0

Char. 178: 1 --> 2

Char. 180: 1 --> 0

Char. 194: 0 --> 1

Char. 195: 0 --> 1

Char. 222: 0 --> 1

Char. 231: 1 --> 0

Char. 238: 0 --> 2

Char. 247: 0 --> 1

Char. 342: 0 --> 1

Char. 357: 1 --> 0

Char. 392: 0 --> 1

Char. 399: 1 --> 0

Char. 404: 0 --> 1

Char. 454: 0 --> 1

Char. 479: 2 --> 3

Char. 539: 0 --> 1

Char. 760: 0 --> 1

Char. 781: 0 --> 1

Char. 790: 1 --> 0

Char. 806: 0 --> 1

Mononykus_olecranus :

Char. 285: 1 --> 2

Char. 484: 1 --> 0

Char. 543: 0 --> 1

Char. 560: 0 --> 1

Char. 688: 1 --> 0

Char. 952: 0 --> 1

Char. 953: 0 --> 1

Char. 955: 1 --> 2

Murusraptor_barrosaensis :

Char. 879: 1 --> 0

Neovenator_salerii :

Char. 4: 0 --> 1

Char. 15: 1 --> 2

Char. 16: 2 --> 1

Char. 20: 1 --> 0

Char. 33: 1 --> 2

Char. 34: 1 --> 0

Char. 63: 1 --> 0

Char. 76: 1 --> 2

Char. 188: 1 --> 0

Char. 395: 1 --> 2

Char. 399: 1 --> 2

Char. 470: 0 --> 1

Char. 478: 1 --> 2

Char. 479: 3 --> 2

Char. 526: 1 --> 2

Char. 534: 0 --> 1

Char. 549: 1 --> 0

Char. 552: 1 --> 0

Char. 560: 0 --> 1

Char. 562: 0 --> 1

Char. 578: 0 --> 1

Char. 598: 1 --> 0

Char. 806: 0 --> 1

Char. 819: 1 --> 0

Char. 827: 0 --> 1

Char. 876: 1 --> 2

Char. 882: 0 --> 1

Char. 887: 0 --> 1

Char. 900: 1 --> 2

Char. 938: 1 --> 0

Char. 953: 0 --> 1

Noasaurus_leali :

Char. 58: 1 --> 0

Char. 67: 1 --> 0

Char. 69: 2 --> 1

Char. 498: 1 --> 2

Char. 517: 1 --> 2

Char. 519: 0 --> 1

Nomingia_gobiensis :

Char. 562: 0 --> 1

Char. 585: 3 --> 2

Char. 617: 0 --> 1

Char. 848: 0 --> 1

Char. 855: 2 --> 1

Nothronychus :

Char. 641: 1 --> 0

Char. 646: 1 --> 0

Char. 759: 1 --> 2

Char. 770: 0 --> 1

Char. 772: 0 --> 1

Char. 825: 1 --> 2

Char. 897: 2 --> 3

Char. 901: 1 --> 2

Nqwebasaurus_thwazi :

Char. 49: 0 --> 1

Char. 79: 0 --> 1

Char. 104: 0 --> 1

Char. 121: 0 --> 1

Char. 201: 1 --> 0

Char. 203: 0 --> 1

Char. 422: 2 --> 1

Char. 502: 1 --> 0

Char. 548: 0 --> 2

Char. 693: 2 --> 1

Char. 707: 1 --> 0

Char. 746: 1 --> 0

Char. 847: 0 --> 1

Char. 873: 0 --> 1

Char. 902: 1 --> 0

Char. 913: 1 --> 0

Char. 954: 1 --> 2

Ornitholestes_hermanni :

Char. 80: 0 --> 1

Char. 88: 0 --> 1

Char. 127: 1 --> 0

Char. 133: 0 --> 1

Char. 215: 0 --> 2

Char. 230: 1 --> 0

Char. 246: 0 --> 1

Char. 329: 0 --> 1

Char. 331: 0 --> 1

Char. 357: 1 --> 0

Char. 360: 1 --> 0

Char. 397: 0 --> 1

Char. 398: 1 --> 2

Char. 399: 1 --> 2

Char. 412: 0 --> 1

Char. 418: 0 --> 1

Char. 512: 0 --> 1

Char. 554: 1 --> 0

Char. 609: 0 --> 3

Char. 657: 0 --> 1

Char. 772: 0 --> 1

Char. 818: 0 --> 1

Char. 870: 0 --> 1

Char. 874: 0 --> 1

Char. 902: 1 --> 0

Char. 914: 3 --> 2

Char. 932: 0 --> 1

Ornithomimus :

Char. 557: 0 --> 1

Char. 749: 0 --> 1

Char. 750: 0 --> 1

Char. 752: 0 --> 1

Oviraptor_philoceratops :

Char. 342: 1 --> 0

Patagonykus_puertai :

Char. 563: 0 --> 1

Char. 566: 0 --> 1

Char. 575: 0 --> 2

Char. 614: 1 --> 2

Char. 632: 0 --> 1

Char. 672: 1 --> 0

Char. 703: 0 --> 1

Char. 738: 1 --> 0

Char. 741: 0 --> 1

Char. 784: 1 --> 0

Char. 870: 0 --> 1

Char. 873: 0 --> 1

Char. 913: 1 --> 0

Char. 921: 1 --> 0

Char. 925: 0 --> 1

Char. 933: 0 --> 1

Pelecanimimus_polyodon :

Char. 22: 1 --> 0

Char. 52: 0 --> 1

Char. 91: 1 --> 0

Char. 97: 0 --> 1

Char. 111: 0 --> 1

Char. 114: 0 --> 1

Char. 125: 0 --> 1

Char. 126: 0 --> 1

Char. 127: 1 --> 0

Char. 165: 0 --> 1

Char. 251: 0 --> 1

Char. 435: 0 --> 1

Char. 536: 0 --> 1

Char. 557: 0 --> 1

Char. 571: 0 --> 1

Piatnitzkysaurus_floresi :

Char. 41: 0 --> 1

Char. 75: 0 --> 1

Char. 201: 0 --> 1

Char. 254: 1 --> 0

Char. 255: 1 --> 0

Char. 276: 1 --> 0

Char. 278: 0 --> 1

Char. 327: 0 --> 1

Char. 334: 0 --> 1

Char. 417: 0 --> 1

Char. 438: 1 --> 0

Char. 506: 1 --> 2

Char. 526: 1 --> 2

Char. 540: 0 --> 1

Char. 657: 0 --> 1

Char. 664: 0 --> 1

Char. 757: 0 --> 1

Char. 853: 0 --> 1

Char. 889: 0 --> 1

Char. 944: 0 --> 1

Proceratosaurus_bradleyi :

Char. 65: 1 --> 0

Char. 409: 2 --> 3

Char. 412: 0 --> 1

Rahonavis_ostromi :

Char. 331: 0 --> 1

Char. 535: 0 --> 2

Char. 536: 0 --> 2

Char. 548: 2 --> 0

Char. 554: 1 --> 0

Char. 562: 0 --> 1

Char. 575: 0 --> 1

Char. 578: 1 --> 0

Char. 593: 0 --> 1

Char. 655: 1 --> 2

Char. 755: 2 --> 3

Char. 759: 1 --> 2

Char. 763: 1 --> 0

Char. 783: 0 --> 1

Char. 806: 1 --> 0

Char. 807: 0 --> 1

Char. 819: 01 --> 2

Char. 835: 2 --> 1

Char. 836: 1 --> 2

Char. 853: 1 --> 2

Char. 874: 0 --> 1

Char. 886: 2 --> 1

Char. 905: 0 --> 1

Char. 934: 0 --> 1

Rajasaurus_narmadensis :

Char. 136: 0 --> 1

Char. 202: 0 --> 1

Rinchenia_mongoliensis :

Char. 116: 1 --> 0

Char. 124: 1 --> 0

Char. 331: 0 --> 1

Char. 381: 0 --> 1

Char. 534: 1 --> 0

Rugops_primus :

Char. 23: 1 --> 0

Char. 31: 0 --> 1

Char. 47: 1 --> 0

Char. 84: 1 --> 0

Char. 121: 1 --> 2

Char. 186: 1 --> 0

Char. 187: 2 --> 0

Char. 191: 0 --> 1

Char. 192: 0 --> 1

Santanaraptor_placidus :

Char. 606: 0 --> 1

Char. 609: 0 --> 4

Char. 867: 0 --> 1

Char. 873: 0 --> 1

Char. 919: 1 --> 0

Sapeornis_chaoyangensis :

Char. 4: 1 --> 0

Char. 17: 0 --> 1

Char. 19: 1 --> 0

Char. 34: 0 --> 1

Char. 128: 0 --> 1

Char. 136: 1 --> 0

Char. 203: 0 --> 1

Char. 328: 0 --> 1

Char. 363: 0 --> 1

Char. 591: 1 --> 0

Char. 630: 0 --> 1

Char. 670: 0 --> 1

Char. 821: 0 --> 1

Char. 824: 0 --> 1

Char. 946: 2 --> 1

Saurornithoides_mongoliensis :

Char. 1: 1 --> 2

Char. 23: 1 --> 2

Char. 80: 1 --> 0

Char. 327: 1 --> 2

Char. 409: 3 --> 2

Saurornitholestes :

Char. 23: 1 --> 0

Char. 29: 0 --> 1

Char. 40: 0 --> 1

Char. 57: 0 --> 1

Char. 104: 0 --> 1

Char. 126: 0 --> 1

Char. 135: 1 --> 2

Char. 145: 1 --> 0

Char. 192: 0 --> 1

Char. 194: 0 --> 1

Char. 195: 0 --> 1

Char. 333: 0 --> 1

Char. 335: 0 --> 1

Char. 425: 1 --> 0

Char. 503: 1 --> 0

Char. 620: 2 --> 1

Char. 681: 1 --> 0

Char. 759: 1 --> 2

Char. 773: 1 --> 0

Char. 780: 0 --> 1

Char. 783: 0 --> 1

Char. 787: 1 --> 0

Char. 906: 0 --> 1

Char. 922: 1 --> 0

Char. 938: 1 --> 0

Segnosaurus_galbinensis :

Char. 631: 0 --> 1

Char. 646: 1 --> 2

Char. 821: 0 --> 1

Char. 879: 1 --> 0

Char. 901: 1 --> 0

Char. 914: 3 --> 2

Shaochilong_maortuensis :

Char. 41: 0 --> 1

Char. 61: 2 --> 0

Char. 69: 12 --> 0

Char. 70: 0 --> 1

Char. 86: 1 --> 2

Char. 200: 0 --> 1

Char. 207: 0 --> 1

Char. 208: 0 --> 1

Char. 213: 0 --> 1

Char. 258: 2 --> 3

Char. 285: 1 --> 2

Char. 295: 1 --> 0

Char. 601: 0 --> 1

Shenzhousaurus_orientalis :

Char. 15: 2 --> 3

Char. 58: 12 --> 0

Char. 160: 0 --> 1

Char. 335: 0 --> 1

Char. 718: 1 --> 0

Char. 729: 1 --> 0

Char. 779: 0 --> 1

Char. 782: 0 --> 1

Char. 784: 1 --> 0

Char. 791: 1 --> 0

Char. 804: 0 --> 1

Char. 818: 0 --> 1

Shuvuuia_deserti :

Char. 503: 1 --> 0

Char. 513: 1 --> 0

Char. 526: 0 --> 1

Char. 886: 12 --> 0

Char. 954: 1 --> 0

Char. 955: 1 --> 0

Char. 956: 12 --> 0

Char. 959: 1 --> 0

Siamraptor_suwati :

Char. 127: 2 --> 3

Char. 512: 1 --> 0

Siats_meekerorum :

Char. 537: 0 --> 1

Char. 548: 0 --> 2

Char. 561: 0 --> 1

Char. 567: 0 --> 1

Char. 603: 0 --> 1

Char. 780: 1 --> 0

Char. 785: 0 --> 1

Sinocalliopteryx_gigas :

Char. 23: 1 --> 0

Char. 91: 1 --> 0

Char. 192: 0 --> 1

Char. 320: 0 --> 1

Char. 445: 0 --> 1

Char. 505: 0 --> 1

Char. 532: 1 --> 2

Char. 557: 0 --> 1

Char. 585: 3 --> 4

Char. 641: 1 --> 0

Char. 683: 1 --> 0

Char. 716: 1 --> 0

Char. 718: 1 --> 2

Char. 739: 1 --> 2

Char. 803: 1 --> 0

Char. 830: 0 --> 1

Char. 954: 1 --> 0

Sinornithomimus_dongi :

Char. 1: 2 --> 1

Char. 26: 0 --> 1

Char. 39: 0 --> 1

Char. 98: 1 --> 2

Char. 108: 1 --> 2

Char. 135: 0 --> 2

Char. 198: 0 --> 2

Char. 245: 1 --> 0

Char. 327: 1 --> 2

Char. 460: 1 --> 0

Char. 479: 0 --> 2

Char. 660: 0 --> 1

Char. 704: 1 --> 0

Char. 811: 1 --> 0

Char. 818: 0 --> 1

Sinornithoides_youngi :

Char. 404: 2 --> 0

Char. 409: 3 --> 2

Char. 614: 2 --> 1

Char. 753: 1 --> 0

Char. 939: 1 --> 0

Sinornithosaurus_millenii :

Char. 36: 0 --> 1

Char. 65: 1 --> 2

Char. 99: 1 --> 0

Char. 120: 1 --> 0

Char. 175: 1 --> 0

Char. 201: 1 --> 0

Char. 344: 0 --> 1

Char. 418: 1 --> 0

Char. 425: 2 --> 1

Char. 428: 0 --> 1

Char. 712: 0 --> 1

Char. 772: 1 --> 0

Char. 915: 0 --> 1

Sinosauropteryx_prima :

Char. 13: 1 --> 2

Char. 39: 0 --> 1

Char. 58: 2 --> 0

Char. 121: 0 --> 1

Char. 487: 12 --> 0

Char. 499: 0 --> 1

Char. 585: 3 --> 5

Char. 655: 1 --> 0

Char. 667: 1 --> 2

Char. 694: 0 --> 1

Char. 739: 1 --> 2

Char. 758: 1 --> 0

Char. 783: 1 --> 0

Char. 826: 0 --> 1

Char. 886: 2 --> 0

Sinovenator_changii :

Char. 13: 1 --> 2

Char. 17: 1 --> 0

Char. 34: 0 --> 1

Char. 104: 0 --> 1

Char. 127: 1 --> 0

Char. 278: 0 --> 1

Char. 347: 1 --> 0

Char. 540: 1 --> 0

Char. 580: 1 --> 0

Char. 620: 2 --> 1

Char. 641: 0 --> 1

Char. 753: 1 --> 2

Char. 880: 0 --> 1

Char. 911: 0 --> 1

Char. 941: 0 --> 1

Sinraptor_dongi :

Char. 725: 1 --> 0

Char. 775: 0 --> 1

Char. 791: 1 --> 0

Char. 806: 0 --> 1

Char. 821: 1 --> 0

Char. 867: 0 --> 1

Char. 884: 1 --> 2

Char. 908: 1 --> 0

Char. 914: 2 --> 1

Char. 915: 1 --> 2

Skorpiovenator_bustingorryi :

Char. 48: 0 --> 1

Char. 56: 0 --> 1

Char. 149: 1 --> 0

Char. 164: 0 --> 1

Char. 170: 0 --> 1

Char. 171: 0 --> 1

Char. 330: 0 --> 2

Char. 391: 1 --> 0

Char. 758: 1 --> 0

Char. 795: 1 --> 0

Spectrovenator_ragei :

Char. 110: 1 --> 0

Char. 115: 1 --> 0

Char. 120: 1 --> 0

Char. 150: 1 --> 0

Char. 161: 0 --> 1

Char. 166: 1 --> 0

Char. 173: 1 --> 0

Char. 200: 1 --> 0

Char. 213: 1 --> 0

Char. 215: 1 --> 0

Char. 263: 1 --> 0

Char. 338: 1 --> 0

Char. 347: 0 --> 1

Char. 348: 2 --> 1

Char. 381: 1 --> 0

Char. 797: 1 --> 0

Llukalkan_aliocranianus :

Char. 9: 23 --> 1

Char. 38: 1 --> 0

Char. 119: 1 --> 0

Char. 161: 0 --> 1

Char. 241: 1 --> 2

Char. 248: 1 --> 0

Char. 285: 0 --> 1

Char. 288: 0 --> 1

Char. 402: 0 --> 2

Spinosaurus_aegyptiacus :

Char. 488: 0 --> 1

Char. 498: 0 --> 1

Char. 513: 0 --> 1

Char. 544: 1 --> 0

Char. 549: 1 --> 2

Char. 556: 0 --> 1

Char. 569: 0 --> 1

Char. 586: 0 --> 1

Char. 603: 0 --> 1

MSNM_v4047 :

Char. 50: 0 --> 1

MNBH_TIG_6 :

Char. 494: 1 --> 0

Char. 506: 1 --> 2

Char. 573: 0 --> 1

Char. 584: 0 --> 1

Struthiomimus_altus :

Char. 15: 2 --> 1

Char. 59: 1 --> 0

Char. 186: 0 --> 1

Char. 244: 0 --> 1

Char. 253: 1 --> 0

Char. 262: 0 --> 1

Char. 333: 0 --> 1

Char. 467: 0 --> 1

Char. 510: 1 --> 0

Char. 549: 2 --> 1

Char. 613: 0 --> 1

Char. 683: 0 --> 1

Char. 806: 1 --> 0

Char. 808: 2 --> 0

Char. 811: 1 --> 0

Suchomimus_tenerensis :

Char. 34: 0 --> 1

Char. 229: 1 --> 0

Char. 505: 0 --> 1

Char. 514: 1 --> 0

Char. 515: 1 --> 0

Char. 531: 1 --> 0

Char. 548: 0 --> 1

"Szechuanosaurus"_zigongensis :

Char. 481: 2 --> 1

Char. 525: 0 --> 1

Char. 527: 2 --> 1

Char. 532: 1 --> 2

Char. 547: 0 --> 1

Char. 553: 0 --> 1

Char. 672: 1 --> 2

Char. 682: 0 --> 1

Char. 726: 2 --> 1

Char. 846: 1 --> 0

Tameryraptor_markgrafi :

Char. 188: 1 --> 0

Char. 190: 0 --> 1

Char. 195: 1 --> 0

Char. 237: 0 --> 1

Char. 248: 1 --> 0

Char. 416: 01 --> 2

Char. 417: 1 --> 2

Char. 423: 2 --> 1

Char. 486: 1 --> 0

Char. 514: 1 --> 0

Char. 592: 1 --> 0

Char. 593: 0 --> 2

Char. 598: 1 --> 2

Char. 600: 0 --> 1

Char. 793: 1 --> 0

Char. 816: 1 --> 0

Char. 819: 1 --> 0

Char. 901: 1 --> 2

Tanycolagreus :

Char. 851: 1 --> 0

Char. 855: 1 --> 0

Char. 870: 0 --> 1

Char. 897: 2 --> 3

Char. 922: 1 --> 0

Char. 930: 1 --> 0

Tarbosaurus_bataar :

Char. 0: 1 --> 2

Char. 16: 3 --> 2

Char. 35: 0 --> 1

Char. 67: 0 --> 1

Char. 110: 0 --> 3

Char. 119: 0 --> 1

Char. 160: 0 --> 1

Char. 178: 0 --> 1

Char. 199: 0 --> 1

Char. 224: 0 --> 1

Char. 292: 1 --> 0

Char. 305: 0 --> 1

Char. 368: 1 --> 0

Char. 600: 0 --> 1

Char. 741: 1 --> 0

Char. 747: 0 --> 1

Char. 949: 1 --> 0

Char. 952: 0 --> 1

Char. 959: 1 --> 0

MPCA_Pv_803 :

Char. 127: 2 --> 1

Char. 128: 1 --> 0

Char. 487: 0 --> 12

Char. 657: 1 --> 0

Char. 664: 2 --> 1

Char. 672: 1 --> 2

Char. 855: 0 --> 1

Char. 951: 1 --> 0

Char. 955: 2 --> 1

Torvosaurus_tanneri :

Char. 55: 0 --> 1

Char. 58: 2 --> 0

Char. 61: 2 --> 1

Char. 74: 0 --> 1

Char. 80: 0 --> 1

Char. 108: 1 --> 0

Char. 112: 1 --> 0

Char. 117: 0 --> 1

Char. 126: 1 --> 0

Char. 391: 2 --> 01

Char. 398: 0 --> 1

Char. 441: 0 --> 1

Char. 495: 0 --> 1

Char. 536: 1 --> 2

Char. 542: 0 --> 1

Char. 547: 0 --> 1

Char. 682: 0 --> 1

Char. 775: 1 --> 0

Char. 826: 1 --> 0

Char. 845: 1 --> 0

Char. 853: 0 --> 1

Char. 854: 1 --> 0

Char. 884: 1 --> 2

Troodon_formosus :

Char. 17: 1 --> 0

Char. 224: 1 --> 0

Char. 259: 1 --> 0

Char. 285: 1 --> 0

Char. 295: 0 --> 2

Char. 425: 2 --> 1

Char. 435: 1 --> 0

Char. 560: 0 --> 1

Char. 574: 3 --> 4

Char. 806: 1 --> 0

Char. 853: 1 --> 2

Tsaagan_mangas :

Char. 19: 1 --> 0

Char. 22: 1 --> 0

Char. 32: 0 --> 1

Char. 53: 1 --> 0

Char. 63: 1 --> 0

Char. 70: 0 --> 1

Char. 111: 1 --> 0

Char. 125: 0 --> 1

Char. 207: 1 --> 0

Char. 209: 1 --> 0

Char. 224: 0 --> 1

Char. 425: 1 --> 2

Char. 513: 1 --> 0

Tugulusaurus_faciles :

Char. 855: 1 --> 0

Char. 894: 1 --> 0

Char. 915: 0 --> 1

Tyrannosaurus_rex :

Char. 9: 1 --> 2

Char. 52: 0 --> 1

Char. 56: 1 --> 0

Char. 68: 0 --> 1

Char. 82: 2 --> 1

Char. 86: 1 --> 0

Char. 118: 0 --> 1

Char. 173: 1 --> 0

Char. 219: 1 --> 0

Char. 237: 0 --> 1

Char. 275: 0 --> 1

Char. 297: 0 --> 1

Char. 334: 0 --> 1

Char. 429: 0 --> 1

Char. 548: 1 --> 2

Char. 657: 0 --> 1

Char. 752: 0 --> 1

Char. 762: 0 --> 1

Char. 819: 0 --> 1

Char. 877: 0 --> 1

Tyrannotitan_chubutensis :

Char. 541: 1 --> 0

Char. 560: 0 --> 1

Char. 576: 0 --> 1

Char. 592: 1 --> 0

Char. 807: 0 --> 1

Char. 860: 1 --> 0

Char. 874: 0 --> 1

Velociraptor_mongoliensis :

Char. 59: 1 --> 0

Char. 126: 0 --> 1

Char. 203: 1 --> 0

Char. 244: 0 --> 1

Char. 257: 0 --> 1

Char. 291: 1 --> 0

Char. 509: 0 --> 1

Velocisaurus_unicus :

No autapomorphies

Vespersaurus_paranaensis :

Char. 491: 1 --> 0

Char. 502: 1 --> 0

Char. 517: 1 --> 0

Char. 674: 1 --> 0

Char. 940: 0 --> 1

Wiehenvenator_albati :

Char. 15: 2 --> 1

Char. 17: 1 --> 0

Char. 51: 0 --> 1

Char. 61: 2 --> 0

Char. 155: 1 --> 2

Char. 340: 1 --> 0

Char. 417: 0 --> 1

Char. 569: 0 --> 1

Char. 605: 1 --> 0

Char. 918: 0 --> 1

Char. 919: 1 --> 0

Xuanhanosaurus_qilixiaensis :

Char. 539: 0 --> 1

Char. 542: 0 --> 1

Char. 638: 0 --> 1

Yangchuanosaurus_hepingensis :

Char. 10: 0 --> 1

Char. 121: 1 --> 2

Char. 128: 0 --> 1

Char. 382: 1 --> 2

Char. 409: 2 --> 1

Char. 759: 0 --> 1

Char. 848: 0 --> 1

Char. 856: 0 --> 1

Yangchuanosaurus_shangyouensis :

Char. 109: 1 --> 0

Char. 167: 1 --> 0

Char. 170: 0 --> 1

Char. 438: 1 --> 0

Char. 549: 1 --> 2

Char. 862: 1 --> 0

Yixianornis_grabaui :

Char. 330: 2 --> 0

Char. 536: 0 --> 2

Char. 580: 1 --> 0

Char. 623: 1 --> 0

Char. 628: 1 --> 0

Char. 696: 1 --> 0

Char. 759: 1 --> 0

Char. 761: 1 --> 0

Char. 763: 0 --> 1

Char. 836: 1 --> 0

Yuanmouraptor_jinshajiangensis :

Char. 16: 2 --> 1

Char. 21: 1 --> 0

Char. 53: 0 --> 1

Char. 72: 1 --> 0

Char. 126: 1 --> 0

Char. 141: 1 --> 0

Char. 172: 0 --> 1

Char. 229: 1 --> 0

Char. 242: 1 --> 0

Char. 256: 0 --> 1

Char. 331: 0 --> 1

Char. 374: 0 --> 1

Char. 392: 0 --> 1

Char. 457: 1 --> 0

Char. 465: 1 --> 0

Char. 478: 1 --> 2

Char. 480: 1 --> 0

Char. 483: 1 --> 0

Char. 493: 1 --> 0

Char. 495: 0 --> 1

Char. 498: 1 --> 0

Char. 505: 1 --> 0

Char. 513: 0 --> 12

Char. 520: 1 --> 0

Char. 521: 0 --> 1

Yunyangosaurus_puanensis :

Char. 481: 2 --> 1

Char. 488: 0 --> 1

Char. 490: 0 --> 1

Char. 494: 1 --> 0

Char. 527: 1 --> 2

Char. 540: 0 --> 1

Char. 544: 0 --> 1

Yutyrannus_huali :

Char. 1: 1 --> 2

Char. 3: 1 --> 2

Char. 4: 0 --> 1

Char. 39: 0 --> 1

Char. 44: 0 --> 1

Char. 52: 0 --> 1

Char. 65: 0 --> 2

Char. 71: 1 --> 0

Char. 72: 1 --> 0

Char. 96: 1 --> 3

Char. 103: 1 --> 0

Char. 107: 0 --> 1

Char. 110: 0 --> 2

Char. 148: 1 --> 0

Char. 194: 0 --> 1

Char. 351: 0 --> 1

Char. 465: 1 --> 0

Char. 536: 1 --> 0

Char. 657: 0 --> 1

Char. 954: 1 --> 0

Zanabazar_junior :

No autapomorphies

Zuolong_salleei :

Char. 15: 1 --> 2

Char. 21: 1 --> 0

Char. 22: 1 --> 0

Char. 58: 2 --> 1

Char. 108: 1 --> 2

Char. 116: 0 --> 1

Char. 198: 0 --> 2

Char. 213: 0 --> 1

Char. 215: 0 --> 1

Char. 424: 1 --> 0

Char. 520: 1 --> 0

Char. 682: 1 --> 0

Char. 812: 1 --> 0

Char. 854: 1 --> 0

Char. 855: 1 --> 0

Char. 900: 1 --> 2

Char. 933: 0 --> 1

Char. 939: 0 --> 1

Char. 958: 0 --> 1

Zupaysaurus_rougieri :

Char. 44: 1 --> 0

Char. 79: 0 --> 1

Char. 165: 0 --> 1

Char. 342: 1 --> 0

Char. 343: 0 --> 1

Char. 434: 1 --> 2

Char. 534: 0 --> 1

Char. 631: 0 --> 1

Char. 650: 0 --> 1

Node 189 :

Char. 91: 0 --> 1

Char. 329: 1 --> 0

Char. 393: 1 --> 0

Char. 415: 1 --> 0

Char. 417: 2 --> 0

Char. 423: 12 --> 3

Char. 431: 1 --> 0

Char. 781: 1 --> 0

Node 190 :

No synapomorphies

Node 191 :

Char. 331: 0 --> 1

Char. 357: 0 --> 1

Char. 539: 0 --> 1

Char. 578: 0 --> 1

Node 192 :

Char. 88: 1 --> 0

Char. 117: 1 --> 0

Char. 140: 0 --> 1

Char. 203: 0 --> 1

Char. 223: 0 --> 1

Char. 266: 0 --> 1

Char. 436: 0 --> 1

Char. 650: 1 --> 0

Char. 676: 0 --> 1

Char. 678: 0 --> 1

Char. 755: 0 --> 1

Node 193 :

Char. 235: 0 --> 1

Char. 434: 1 --> 2

Char. 648: 0 --> 1

Char. 777: 0 --> 1

Char. 792: 0 --> 1

Char. 884: 1 --> 0

Node 194 :

Char. 69: 12 --> 0

Char. 322: 1 --> 2

Char. 404: 0 --> 1

Node 195 :

Char. 553: 1 --> 0

Char. 611: 0 --> 1

Char. 848: 2 --> 0

Char. 952: 0 --> 1

Node 196 :

Char. 58: 1 --> 2

Char. 266: 0 --> 1

Char. 444: 0 --> 1

Char. 592: 0 --> 2

Char. 808: 0 --> 1

Char. 844: 0 --> 1

Char. 845: 0 --> 1

Char. 885: 0 --> 1

Char. 898: 0 --> 1

Char. 908: 0 --> 1

Node 197 :

Char. 88: 1 --> 0

Char. 124: 0 --> 1

Char. 132: 1 --> 0

Char. 173: 1 --> 0

Char. 196: 1 --> 0

Char. 208: 1 --> 0

Char. 356: 1 --> 0

Char. 357: 0 --> 1

Char. 358: 0 --> 1

Char. 392: 0 --> 1

Char. 406: 1 --> 0

Char. 414: 1 --> 0

Char. 416: 12 --> 0

Char. 428: 1 --> 0

Char. 734: 0 --> 1

Char. 839: 1 --> 0

Char. 915: 1 --> 2

Char. 948: 1 --> 0

Node 198 :

Char. 209: 2 --> 0

Char. 250: 1 --> 0

Node 199 :

Char. 592: 2 --> 1

Char. 595: 0 --> 1

Char. 857: 1 --> 0

Node 200 :

Char. 59: 0 --> 1

Char. 91: 1 --> 2

Char. 102: 1 --> 0

Char. 108: 1 --> 0

Char. 213: 0 --> 1

Node 201 :

Char. 61: 1 --> 0

Char. 69: 12 --> 0

Char. 70: 0 --> 1

Char. 81: 0 --> 1

Char. 103: 0 --> 1

Char. 111: 0 --> 1

Char. 114: 0 --> 1

Char. 116: 0 --> 1

Char. 119: 0 --> 1

Char. 189: 0 --> 1

Char. 409: 1 --> 2

Char. 416: 0 --> 1

Char. 422: 3 --> 2

Node 202 :

Char. 3: 1 --> 0

Char. 9: 2 --> 3

Char. 15: 2 --> 1

Char. 16: 1 --> 2

Char. 33: 1 --> 2

Char. 40: 0 --> 1

Char. 86: 0 --> 1

Char. 166: 0 --> 1

Char. 177: 0 --> 1

Char. 185: 0 --> 1

Char. 198: 0 --> 1

Char. 222: 0 --> 1

Char. 269: 1 --> 0

Char. 285: 1 --> 0

Char. 300: 0 --> 1

Char. 311: 1 --> 0

Char. 363: 0 --> 1

Char. 391: 2 --> 01

Char. 453: 0 --> 1

Char. 454: 0 --> 1

Char. 795: 0 --> 1

Char. 854: 0 --> 1

Char. 937: 0 --> 2

Node 203 :

Char. 50: 0 --> 1

Char. 61: 2 --> 1

Char. 67: 0 --> 1

Char. 80: 0 --> 1

Char. 110: 0 --> 2

Char. 118: 0 --> 1

Char. 144: 0 --> 1

Char. 156: 0 --> 1

Char. 179: 1 --> 0

Char. 218: 0 --> 1

Char. 260: 1 --> 0

Char. 344: 0 --> 1

Char. 398: 0 --> 1

Char. 423: 3 --> 2

Char. 465: 0 --> 1

Char. 490: 0 --> 1

Char. 491: 0 --> 1

Char. 563: 0 --> 1

Char. 569: 0 --> 1

Char. 574: 2 --> 3

Char. 577: 0 --> 1

Char. 581: 0 --> 1

Char. 614: 1 --> 0

Char. 637: 0 --> 1

Char. 649: 0 --> 1

Char. 653: 1 --> 0

Char. 691: 2 --> 0

Char. 695: 1 --> 0

Char. 737: 0 --> 1

Char. 772: 0 --> 3

Char. 788: 0 --> 1

Char. 794: 0 --> 1

Char. 798: 0 --> 1

Char. 842: 1 --> 0

Char. 844: 1 --> 0

Char. 861: 0 --> 1

Char. 874: 0 --> 1

Char. 878: 0 --> 1

Char. 912: 0 --> 1

Char. 957: 0 --> 1

Char. 958: 0 --> 2

Node 204 :

Char. 8: 1 --> 0

Char. 113: 0 --> 1

Char. 117: 1 --> 0

Char. 153: 0 --> 1

Char. 178: 0 --> 1

Char. 192: 1 --> 0

Char. 532: 2 --> 1

Char. 843: 0 --> 1

Char. 919: 0 --> 1

Node 205 :

Char. 285: 0 --> 1

Char. 554: 0 --> 1

Char. 787: 1 --> 0

Char. 839: 0 --> 1

Char. 921: 0 --> 1

Node 206 :

Char. 15: 3 --> 2

Char. 16: 0 --> 1

Char. 19: 1 --> 0

Char. 28: 1 --> 0

Char. 45: 1 --> 0

Char. 75: 2 --> 0

Char. 241: 0 --> 1

Char. 250: 0 --> 1

Char. 260: 0 --> 1

Char. 391: 1 --> 2

Char. 392: 1 --> 0

Char. 513: 1 --> 0

Char. 514: 0 --> 1

Char. 553: 0 --> 1

Char. 562: 0 --> 1

Char. 585: 4 --> 3

Char. 672: 1 --> 2

Char. 703: 1 --> 0

Char. 862: 0 --> 1

Char. 906: 0 --> 1

Char. 946: 1 --> 2

Char. 956: 1 --> 2

Node 207 :

Char. 8: 0 --> 1

Char. 9: 1 --> 2

Char. 35: 1 --> 0

Char. 38: 0 --> 1

Char. 59: 1 --> 0

Char. 71: 0 --> 1

Char. 118: 1 --> 0

Char. 121: 0 --> 1

Char. 139: 1 --> 0

Char. 205: 0 --> 1

Char. 231: 0 --> 1

Char. 290: 1 --> 2

Char. 409: 2 --> 1

Char. 481: 0 --> 1

Char. 498: 0 --> 1

Char. 513: 2 --> 1

Char. 539: 0 --> 1

Char. 555: 0 --> 1

Char. 644: 0 --> 1

Char. 731: 1 --> 0

Char. 903: 1 --> 0

Node 208 :

Char. 7: 0 --> 1

Char. 17: 0 --> 1

Char. 102: 0 --> 1

Char. 104: 0 --> 1

Char. 112: 0 --> 1

Char. 245: 0 --> 1

Char. 296: 0 --> 1

Char. 549: 2 --> 1

Char. 598: 0 --> 1

Char. 599: 0 --> 1

Char. 650: 1 --> 0

Char. 797: 0 --> 1

Char. 877: 0 --> 1

Char. 892: 0 --> 1

Char. 902: 0 --> 1

Char. 917: 0 --> 1

Node 209 :

Char. 23: 0 --> 1

Char. 29: 0 --> 1

Char. 43: 0 --> 1

Char. 69: 1 --> 2

Char. 536: 0 --> 1

Char. 574: 1 --> 2

Char. 614: 0 --> 1

Char. 661: 0 --> 1

Char. 697: 0 --> 1

Char. 700: 0 --> 1

Char. 717: 1 --> 0

Char. 718: 0 --> 1

Char. 754: 0 --> 1

Char. 781: 0 --> 1

Char. 786: 0 --> 1

Char. 787: 0 --> 1

Char. 789: 1 --> 2

Char. 790: 0 --> 1

Char. 850: 0 --> 1

Char. 929: 1 --> 2

Node 210 :

Char. 86: 1 --> 2

Char. 498: 1 --> 0

Char. 537: 0 --> 1

Char. 618: 1 --> 0

Char. 803: 1 --> 3

Char. 955: 1 --> 2

Node 211 :

Char. 334: 0 --> 1

Char. 544: 0 --> 1

Char. 575: 0 --> 1

Char. 608: 0 --> 1

Char. 614: 1 --> 0

Char. 788: 0 --> 1

Node 212 :

Char. 40: 0 --> 1

Char. 491: 0 --> 1

Char. 495: 0 --> 1

Char. 536: 1 --> 2

Char. 558: 0 --> 1

Char. 565: 0 --> 1

Char. 592: 2 --> 1

Node 213 :

Char. 80: 0 --> 1

Char. 140: 0 --> 1

Char. 205: 1 --> 2

Char. 213: 0 --> 1

Char. 494: 1 --> 0

Char. 515: 0 --> 1

Char. 631: 0 --> 1

Char. 664: 0 --> 2

Char. 801: 1 --> 0

Char. 839: 1 --> 2

Char. 893: 0 --> 1

Node 214 :

Char. 144: 0 --> 1

Char. 161: 0 --> 1

Char. 163: 1 --> 2

Char. 168: 0 --> 1

Char. 219: 1 --> 0

Char. 292: 0 --> 1

Char. 307: 0 --> 1

Char. 363: 0 --> 2

Char. 473: 1 --> 0

Char. 479: 2 --> 3

Char. 527: 1 --> 2

Char. 661: 0 --> 1

Char. 775: 1 --> 0

Char. 842: 1 --> 0

Node 215 :

Char. 45: 2 --> 1

Char. 84: 1 --> 0

Char. 96: 1 --> 2

Char. 178: 1 --> 2

Char. 194: 0 --> 1

Char. 195: 0 --> 1

Char. 198: 0 --> 1

Char. 209: 0 --> 2

Char. 222: 0 --> 1

Char. 247: 0 --> 1

Char. 361: 0 --> 1

Char. 460: 1 --> 2

Char. 655: 1 --> 0

Char. 706: 0 --> 1

Char. 849: 0 --> 1

Char. 938: 0 --> 1

Char. 952: 0 --> 1

Node 216 :

Char. 16: 1 --> 2

Char. 34: 0 --> 1

Char. 62: 0 --> 1

Char. 76: 0 --> 1

Char. 86: 2 --> 1

Char. 295: 0 --> 1

Char. 377: 1 --> 0

Char. 531: 1 --> 0

Char. 564: 1 --> 0

Char. 616: 1 --> 0

Char. 618: 0 --> 1

Char. 636: 1 --> 2

Char. 661: 2 --> 0

Char. 686: 1 --> 0

Char. 796: 0 --> 1

Char. 853: 0 --> 1

Char. 861: 0 --> 1

Char. 862: 0 --> 1

Node 217 :

Char. 63: 0 --> 1

Char. 269: 1 --> 0

Char. 283: 0 --> 1

Char. 337: 0 --> 1

Char. 451: 0 --> 2

Char. 465: 0 --> 1

Char. 525: 1 --> 0

Char. 580: 1 --> 0

Char. 761: 0 --> 2

Char. 777: 0 --> 1

Char. 844: 1 --> 0

Char. 869: 0 --> 1

Char. 914: 1 --> 2

Node 218 :

Char. 45: 0 --> 2

Char. 268: 04 --> 2

Char. 481: 1 --> 2

Char. 637: 0 --> 1

Char. 672: 2 --> 1

Char. 818: 0 --> 1

Char. 840: 0 --> 1

Char. 854: 0 --> 1

Node 219 :

Char. 273: 1 --> 0

Char. 274: 1 --> 0

Char. 797: 1 --> 0

Char. 835: 0 --> 1

Char. 862: 1 --> 0

Node 220 :

Char. 32: 1 --> 0

Char. 86: 0 --> 2

Char. 253: 0 --> 1

Char. 376: 1 --> 0

Char. 424: 0 --> 1

Char. 467: 0 --> 1

Char. 473: 0 --> 1

Char. 488: 1 --> 0

Char. 516: 0 --> 1

Char. 517: 1 --> 0

Char. 525: 0 --> 1

Char. 541: 0 --> 1

Char. 564: 0 --> 1

Char. 756: 1 --> 0

Char. 868: 0 --> 1

Char. 886: 0 --> 2

Node 221 :

Char. 549: 1 --> 0

Char. 560: 0 --> 1

Char. 564: 0 --> 1

Char. 636: 2 --> 1

Char. 653: 0 --> 1

Node 222 :

Char. 601: 0 --> 1

Node 223 :

Char. 536: 1 --> 2

Char. 598: 1 --> 2

Char. 599: 1 --> 2

Char. 602: 0 --> 1

Node 224 :

Char. 178: 1 --> 0

Char. 203: 0 --> 1

Char. 212: 0 --> 1

Char. 228: 0 --> 1

Char. 285: 1 --> 0

Char. 286: 0 --> 1

Char. 295: 1 --> 2

Char. 299: 0 --> 1

Node 225 :

Char. 180: 1 --> 0

Char. 215: 0 --> 2

Char. 217: 0 --> 1

Char. 454: 0 --> 1

Char. 491: 0 --> 1

Node 226 :

Char. 13: 0 --> 1

Char. 44: 1 --> 0

Char. 77: 0 --> 1

Char. 98: 0 --> 1

Char. 160: 1 --> 0

Char. 187: 1 --> 0

Char. 239: 0 --> 1

Char. 255: 1 --> 0

Char. 260: 1 --> 0

Char. 278: 0 --> 1

Char. 282: 2 --> 1

Char. 335: 1 --> 0

Char. 340: 1 --> 0

Char. 402: 0 --> 2

Char. 479: 2 --> 1

Char. 493: 1 --> 0

Char. 516: 1 --> 0

Char. 569: 0 --> 1

Char. 619: 0 --> 1

Char. 621: 1 --> 0

Char. 653: 1 --> 0

Char. 705: 1 --> 0

Char. 719: 0 --> 1

Char. 741: 0 --> 1

Char. 780: 0 --> 1

Char. 826: 1 --> 0

Char. 851: 0 --> 1

Char. 855: 0 --> 1

Char. 894: 0 --> 1

Char. 913: 0 --> 1

Char. 915: 1 --> 0

Char. 956: 2 --> 1

Node 227 :

Char. 486: 0 --> 1

Char. 511: 1 --> 0

Char. 530: 1 --> 0

Char. 888: 0 --> 1

Char. 893: 0 --> 1

Char. 912: 1 --> 0

Char. 941: 0 --> 1

Char. 953: 0 --> 1

Node 228 :

Char. 460: 1 --> 2

Char. 478: 1 --> 2

Char. 479: 2 --> 3

Char. 480: 1 --> 0

Char. 501: 0 --> 1

Char. 509: 0 --> 1

Char. 656: 0 --> 1

Char. 832: 0 --> 1

Char. 862: 1 --> 2

Char. 931: 1 --> 2

Node 229 :

Char. 507: 1 --> 0

Char. 512: 1 --> 0

Char. 526: 1 --> 0

Char. 527: 1 --> 0

Char. 555: 1 --> 0

Node 230 :

Char. 513: 0 --> 2

Char. 520: 1 --> 0

Char. 549: 1 --> 2

Char. 554: 1 --> 0

Char. 561: 0 --> 1

Char. 562: 1 --> 0

Node 231 :

Char. 82: 1 --> 2

Char. 182: 0 --> 1

Char. 453: 0 --> 1

Char. 790: 1 --> 0

Char. 843: 1 --> 0

Node 232 :

Char. 47: 1 --> 0

Char. 179: 1 --> 0

Char. 521: 0 --> 1

Char. 639: 0 --> 1

Char. 667: 1 --> 2

Char. 906: 1 --> 0

Char. 927: 0 --> 1

Node 233 :

Char. 91: 0 --> 1

Char. 143: 0 --> 1

Char. 197: 0 --> 1

Char. 228: 1 --> 0

Char. 270: 1 --> 0

Node 234 :

Char. 69: 1 --> 0

Char. 144: 0 --> 1

Char. 269: 1 --> 3

Char. 350: 1 --> 2

Char. 380: 0 --> 1

Char. 421: 0 --> 1

Char. 570: 1 --> 0

Char. 820: 1 --> 2

Char. 821: 1 --> 0

Char. 856: 0 --> 1

Node 235 :

Char. 6: 0 --> 1

Char. 10: 0 --> 2

Char. 53: 0 --> 1

Char. 155: 1 --> 2

Char. 170: 0 --> 1

Char. 342: 0 --> 1

Char. 362: 0 --> 1

Char. 461: 1 --> 0

Char. 536: 1 --> 2

Char. 936: 0 --> 1

Node 236 :

Char. 16: 2 --> 3

Char. 189: 0 --> 2

Char. 335: 0 --> 1

Node 237 :

Char. 182: 0 --> 1

Char. 672: 1 --> 2

Char. 694: 1 --> 0

Char. 775: 1 --> 2

Char. 879: 1 --> 0

Node 238 :

Char. 583: 0 --> 1

Char. 737: 0 --> 1

Char. 870: 0 --> 1

Node 239 :

Char. 558: 0 --> 1

Char. 703: 0 --> 1

Char. 889: 0 --> 1

Node 240 :

Char. 460: 2 --> 1

Char. 790: 1 --> 0

Node 241 :

Char. 41: 0 --> 1

Char. 44: 1 --> 0

Char. 98: 0 --> 1

Char. 153: 1 --> 0

Char. 241: 1 --> 0

Char. 248: 1 --> 0

Char. 298: 1 --> 0

Char. 337: 1 --> 0

Char. 424: 1 --> 0

Char. 462: 0 --> 1

Node 242 :

Char. 146: 0 --> 1

Char. 154: 0 --> 1

Char. 162: 0 --> 1

Char. 385: 1 --> 0

Char. 388: 1 --> 0

Char. 475: 2 --> 1

Node 243 :

Char. 4: 0 --> 1

Char. 41: 0 --> 1

Char. 63: 1 --> 0

Char. 110: 0 --> 1

Char. 318: 0 --> 1

Char. 370: 0 --> 1

Char. 463: 0 --> 1

Char. 659: 0 --> 1

Char. 703: 0 --> 1

Node 244 :

Char. 579: 0 --> 1

Char. 586: 0 --> 1

Char. 740: 0 --> 1

Char. 747: 0 --> 1

Char. 756: 0 --> 2

Char. 777: 1 --> 0

Node 245 :

Char. 98: 1 --> 0

Char. 261: 0 --> 1

Char. 273: 0 --> 2

Char. 335: 0 --> 1

Char. 363: 0 --> 1

Char. 380: 0 --> 1

Char. 388: 1 --> 0

Char. 553: 0 --> 1

Char. 554: 1 --> 0

Char. 661: 0 --> 1

Char. 675: 1 --> 0

Char. 686: 0 --> 1

Char. 728: 0 --> 1

Char. 739: 01 --> 2

Char. 742: 0 --> 1

Char. 860: 0 --> 1

Char. 900: 1 --> 2

Node 246 :

Char. 3: 1 --> 0

Char. 53: 1 --> 0

Char. 68: 0 --> 1

Char. 108: 1 --> 2

Char. 140: 1 --> 0

Char. 271: 0 --> 1

Char. 316: 1 --> 2

Char. 694: 0 --> 1

Char. 727: 0 --> 1

Char. 736: 0 --> 1

Char. 738: 0 --> 1

Char. 769: 0 --> 1

Node 247 :

Char. 175: 0 --> 1

Char. 184: 0 --> 1

Char. 201: 0 --> 1

Char. 206: 0 --> 1

Char. 242: 1 --> 0

Char. 278: 1 --> 0

Char. 298: 1 --> 0

Char. 302: 0 --> 1

Char. 348: 1 --> 0

Char. 418: 0 --> 1

Char. 425: 01 --> 2

Char. 427: 0 --> 2

Char. 442: 0 --> 1

Char. 520: 1 --> 0

Char. 526: 1 --> 0

Char. 589: 1 --> 0

Char. 613: 0 --> 2

Char. 628: 0 --> 1

Char. 682: 1 --> 0

Char. 897: 2 --> 3

Char. 903: 0 --> 1

Char. 938: 0 --> 1

Node 248 :

Char. 17: 0 --> 1

Char. 126: 1 --> 0

Char. 277: 0 --> 1

Char. 377: 0 --> 1

Char. 414: 0 --> 1

Char. 422: 3 --> 2

Char. 578: 0 --> 1

Char. 662: 0 --> 1

Char. 773: 0 --> 1

Char. 791: 0 --> 1

Char. 920: 0 --> 1

Node 249 :

Char. 321: 1 --> 0

Char. 373: 1 --> 0

Char. 784: 0 --> 1

Char. 861: 1 --> 0

Node 250 :

Char. 130: 0 --> 1

Char. 165: 1 --> 0

Char. 234: 0 --> 1

Char. 404: 0 --> 2

Char. 417: 0 --> 12

Char. 423: 3 --> 12

Char. 503: 0 --> 1

Char. 643: 0 --> 1

Char. 761: 2 --> 0

Char. 775: 1 --> 0

Node 251 :

Char. 72: 1 --> 0

Node 252 :

Char. 16: 2 --> 1

Char. 104: 1 --> 0

Char. 118: 0 --> 1

Char. 495: 0 --> 1

Char. 741: 1 --> 0

Char. 806: 0 --> 1

Char. 868: 1 --> 0

Char. 906: 1 --> 0

Node 253 :

Char. 510: 0 --> 1

Char. 536: 1 --> 0

Char. 565: 0 --> 1

Char. 605: 1 --> 0

Char. 675: 0 --> 1

Char. 694: 1 --> 0

Char. 713: 1 --> 0

Char. 801: 1 --> 0

Char. 818: 1 --> 0

Char. 819: 1 --> 0

Node 254 :

Char. 96: 1 --> 0

Char. 141: 1 --> 0

Char. 467: 1 --> 0

Char. 483: 1 --> 0

Char. 580: 0 --> 1

Char. 716: 0 --> 1

Char. 837: 0 --> 1

Char. 842: 1 --> 0

Char. 857: 0 --> 1

Char. 892: 1 --> 0

Node 255 :

Char. 441: 0 --> 1

Char. 532: 1 --> 0

Char. 589: 1 --> 0

Char. 831: 0 --> 1

Char. 847: 0 --> 1

Char. 929: 2 --> 0

Char. 958: 0 --> 1

Node 256 :

Char. 328: 0 --> 1

Char. 329: 0 --> 1

Char. 341: 0 --> 1

Char. 393: 0 --> 1

Char. 618: 1 --> 0

Char. 761: 0 --> 1

Char. 783: 1 --> 0

Char. 796: 1 --> 2

Char. 816: 1 --> 2

Char. 820: 0 --> 1

Char. 839: 1 --> 2

Char. 891: 0 --> 1

Char. 940: 0 --> 1

Node 257 :

Char. 177: 0 --> 1

Char. 295: 1 --> 2

Char. 416: 1 --> 2

Char. 436: 0 --> 1

Char. 488: 0 --> 1

Char. 498: 1 --> 2

Char. 513: 1 --> 2

Char. 514: 1 --> 2

Char. 516: 0 --> 1

Char. 531: 0 --> 1

Char. 543: 0 --> 1

Char. 588: 0 --> 1

Char. 710: 0 --> 1

Char. 713: 0 --> 1

Char. 716: 1 --> 0

Char. 760: 0 --> 1

Char. 766: 0 --> 1

Char. 807: 0 --> 1

Char. 810: 1 --> 0

Char. 857: 1 --> 0

Char. 901: 0 --> 1

Char. 927: 0 --> 1

Char. 947: 01 --> 2

Char. 961: 0 --> 1

Node 258 :

Char. 17: 1 --> 0

Char. 30: 0 --> 1

Char. 32: 0 --> 1

Char. 37: 1 --> 0

Char. 184: 1 --> 0

Char. 228: 0 --> 1

Char. 320: 0 --> 1

Char. 397: 0 --> 1

Char. 440: 0 --> 1

Char. 853: 1 --> 2

Char. 949: 1 --> 2

Node 259 :

Char. 9: 1 --> 0

Char. 105: 0 --> 1

Char. 170: 0 --> 1

Char. 335: 0 --> 1

Char. 345: 1 --> 0

Char. 455: 1 --> 0

Char. 588: 0 --> 1

Char. 591: 0 --> 1

Char. 598: 1 --> 0

Char. 609: 0 --> 4

Char. 617: 0 --> 1

Char. 620: 0 --> 1

Char. 632: 0 --> 1

Char. 634: 0 --> 1

Char. 648: 0 --> 1

Char. 681: 0 --> 1

Char. 753: 0 --> 1

Char. 761: 0 --> 1

Char. 770: 0 --> 1

Char. 779: 0 --> 1

Char. 808: 2 --> 0

Char. 825: 1 --> 2

Char. 848: 0 --> 1

Char. 954: 1 --> 0

Char. 955: 1 --> 0

Char. 956: 12 --> 0

Char. 958: 0 --> 1

Node 260 :

Char. 98: 1 --> 2

Char. 120: 0 --> 1

Char. 215: 0 --> 2

Char. 295: 1 --> 0

Char. 306: 0 --> 1

Char. 330: 0 --> 2

Char. 339: 0 --> 1

Char. 373: 0 --> 1

Char. 478: 1 --> 0

Char. 480: 1 --> 0

Char. 490: 1 --> 0

Char. 548: 0 --> 2

Char. 623: 0 --> 1

Char. 683: 1 --> 0

Char. 698: 0 --> 1

Char. 772: 0 --> 1

Char. 782: 0 --> 1

Char. 815: 0 --> 1

Char. 847: 0 --> 1

Char. 852: 2 --> 3

Char. 855: 1 --> 2

Char. 879: 1 --> 0

Char. 899: 0 --> 1

Char. 919: 1 --> 0

Char. 940: 0 --> 1

Char. 965: 0 --> 1

Node 261 :

Char. 747: 1 --> 2

Node 262 :

Char. 65: 0 --> 1

Char. 111: 0 --> 1

Char. 261: 0 --> 1

Char. 304: 1 --> 0

Char. 334: 0 --> 1

Char. 363: 0 --> 1

Char. 748: 1 --> 2

Char. 868: 0 --> 1

Node 263 :

Char. 125: 0 --> 1

Char. 238: 0 --> 1

Char. 574: 3 --> 4

Char. 631: 0 --> 1

Char. 664: 0 --> 1

Char. 714: 1 --> 0

Char. 860: 0 --> 1

Node 264 :

Char. 487: 12 --> 0

Char. 499: 0 --> 1

Char. 520: 0 --> 1

Char. 823: 0 --> 1

Char. 888: 0 --> 1

Char. 928: 0 --> 1

Node 265 :

Char. 497: 01 --> 2

Char. 511: 0 --> 1

Char. 683: 1 --> 0

Char. 773: 1 --> 0

Char. 830: 1 --> 2

Char. 936: 0 --> 1

Node 266 :

Char. 2: 1 --> 2

Char. 9: 1 --> 0

Char. 12: 0 --> 1

Char. 38: 0 --> 2

Char. 902: 1 --> 0

Node 267 :

Char. 0: 1 --> 0

Char. 53: 0 --> 1

Char. 108: 2 --> 1

Char. 110: 0 --> 3

Char. 285: 1 --> 2

Char. 330: 0 --> 2

Char. 433: 0 --> 1

Node 268 :

Char. 33: 1 --> 0

Char. 328: 0 --> 1

Char. 394: 0 --> 1

Char. 408: 0 --> 1

Char. 437: 0 --> 1

Node 269 :

Char. 645: 0 --> 1

Char. 650: 0 --> 1

Char. 694: 1 --> 0

Char. 704: 0 --> 1

Char. 727: 1 --> 0

Char. 747: 0 --> 1

Node 270 :

Char. 658: 0 --> 1

Char. 666: 0 --> 1

Char. 667: 1 --> 0

Char. 675: 1 --> 0

Char. 810: 1 --> 0

Node 271 :

Char. 511: 1 --> 0

Char. 897: 3 --> 2

Node 272 :

Char. 491: 0 --> 1

Char. 549: 1 --> 2

Char. 609: 0 --> 3

Char. 614: 1 --> 0

Char. 662: 1 --> 0

Char. 906: 0 --> 1

Node 273 :

Char. 816: 1 --> 0

Char. 860: 0 --> 1

Node 274 :

Char. 371: 0 --> 1

Char. 383: 0 --> 1

Char. 536: 0 --> 2

Char. 575: 0 --> 1

Char. 592: 0 --> 2

Char. 593: 0 --> 2

Char. 773: 1 --> 0

Char. 852: 3 --> 1

Char. 899: 1 --> 0

Node 275 :

Char. 14: 0 --> 1

Char. 342: 0 --> 1

Char. 354: 0 --> 1

Char. 394: 0 --> 1

Node 276 :

Char. 33: 1 --> 0

Char. 39: 0 --> 1

Char. 56: 1 --> 0

Char. 65: 0 --> 12

Char. 70: 0 --> 1

Char. 195: 0 --> 1

Char. 326: 0 --> 1

Char. 360: 1 --> 0

Node 277 :

Char. 0: 1 --> 0

Char. 1: 1 --> 0

Char. 27: 0 --> 1

Char. 104: 0 --> 1

Char. 322: 1 --> 2

Char. 324: 0 --> 1

Char. 328: 0 --> 1

Char. 329: 0 --> 1

Char. 344: 0 --> 12

Char. 388: 1 --> 0

Char. 445: 0 --> 1

Char. 446: 0 --> 1

Char. 587: 0 --> 1

Char. 953: 0 --> 1

Char. 955: 1 --> 2

Char. 957: 0 --> 1

Char. 960: 1 --> 0

Node 278 :

Char. 326: 0 --> 1

Char. 447: 0 --> 1

Char. 635: 1 --> 0

Char. 805: 0 --> 1

Char. 836: 1 --> 2

Node 279 :

Char. 446: 0 --> 1

Char. 534: 0 --> 1

Char. 574: 4 --> 5

Char. 625: 0 --> 1

Char. 629: 1 --> 2

Char. 633: 0 --> 1

Char. 673: 0 --> 1

Char. 699: 2 --> 3

Char. 702: 0 --> 1

Char. 750: 0 --> 1

Char. 752: 0 --> 1

Char. 815: 1 --> 0

Char. 819: 2 --> 1

Char. 945: 0 --> 1

Char. 949: 2 --> 1

Char. 960: 1 --> 0

Node 280 :

Char. 14: 0 --> 1

Char. 22: 1 --> 0

Char. 24: 0 --> 1

Char. 93: 1 --> 0

Char. 181: 0 --> 1

Char. 320: 1 --> 0

Char. 339: 1 --> 0

Char. 348: 0 --> 1

Char. 408: 0 --> 1

Char. 460: 1 --> 0

Char. 571: 0 --> 1

Char. 753: 2 --> 3

Char. 916: 0 --> 1

Char. 917: 0 --> 1

Node 281 :

Char. 53: 1 --> 0

Char. 58: 2 --> 0

Char. 61: 2 --> 0

Char. 317: 1 --> 2

Char. 329: 0 --> 1

Char. 335: 1 --> 0

Char. 343: 1 --> 0

Char. 574: 3 --> 4

Char. 585: 2 --> 0

Char. 587: 0 --> 1

Char. 590: 0 --> 2

Char. 652: 1 --> 0

Char. 662: 1 --> 0

Char. 699: 0 --> 2

Char. 763: 1 --> 0

Char. 911: 0 --> 1

Char. 912: 0 --> 1

Char. 923: 0 --> 1

Node 282 :

Char. 45: 2 --> 3

Char. 56: 1 --> 0

Char. 70: 0 --> 1

Char. 127: 1 --> 0

Char. 132: 0 --> 1

Char. 166: 0 --> 1

Char. 215: 2 --> 0

Char. 396: 1 --> 0

Char. 409: 1 --> 0

Char. 416: 01 --> 2

Char. 655: 1 --> 2

Char. 753: 1 --> 2

Char. 819: 0 --> 2

Char. 966: 0 --> 1

Node 283 :

Char. 684: 0 --> 1

Char. 685: 0 --> 1

Char. 737: 0 --> 1

Char. 738: 0 --> 2

Char. 741: 1 --> 0

Node 284 :

Char. 782: 0 --> 1

Node 285 :

Char. 0: 1 --> 2

Char. 1: 1 --> 2

Char. 155: 1 --> 2

Char. 156: 0 --> 1

Char. 409: 1 --> 3

Char. 430: 0 --> 1

Char. 486: 0 --> 1

Char. 496: 0 --> 1

Char. 527: 1 --> 0

Node 286 :

Char. 6: 0 --> 1

Char. 214: 1 --> 0

Char. 825: 2 --> 1

Char. 855: 2 --> 1

Node 287 :

Char. 330: 2 --> 0

Char. 641: 0 --> 1

Char. 854: 0 --> 1

Node 288 :

Char. 592: 0 --> 2

Char. 879: 0 --> 1

Char. 927: 0 --> 1

Char. 935: 0 --> 1

Char. 948: 0 --> 1

Char. 961: 0 --> 1

Char. 963: 0 --> 1

Node 289 :

Char. 100: 0 --> 1

Char. 216: 0 --> 1

Char. 305: 0 --> 1

Char. 347: 0 --> 1

Char. 365: 1 --> 0

Char. 368: 0 --> 1

Char. 381: 0 --> 1

Char. 462: 1 --> 0

Char. 620: 1 --> 2

Char. 852: 3 --> 1

Char. 950: 0 --> 1

Char. 960: 1 --> 2

Node 290 :

Char. 83: 0 --> 1

Char. 198: 0 --> 1

Char. 218: 0 --> 1

Char. 228: 0 --> 1

Char. 234: 0 --> 1

Char. 236: 0 --> 1

Char. 238: 0 --> 1

Char. 242: 1 --> 0

Char. 248: 1 --> 2

Char. 268: 2 --> 3

Char. 271: 1 --> 2

Char. 275: 0 --> 1

Char. 431: 0 --> 1

Char. 492: 0 --> 1

Char. 498: 1 --> 0

Char. 521: 0 --> 1

Char. 557: 0 --> 2

Char. 559: 0 --> 1

Node 291 :

Char. 515: 0 --> 1

Char. 906: 1 --> 0

Node 292 :

Char. 799: 0 --> 1

Node 293 :

Char. 8: 0 --> 1

Char. 20: 1 --> 0

Char. 69: 12 --> 0

Char. 110: 0 --> 2

Char. 117: 0 --> 1

Char. 127: 2 --> 3

Char. 185: 0 --> 1

Char. 190: 0 --> 1

Char. 197: 0 --> 1

Char. 331: 0 --> 1

Char. 411: 0 --> 1

Char. 478: 1 --> 2

Char. 795: 0 --> 1

Node 294 :

Char. 772: 0 --> 1

Char. 824: 0 --> 1

Char. 834: 0 --> 1

Node 295 :

Char. 141: 1 --> 0

Char. 335: 1 --> 0

Char. 342: 1 --> 0

Char. 394: 0 --> 1

Char. 408: 0 --> 1

Char. 433: 0 --> 1

Char. 552: 1 --> 0

Node 296 :

Char. 262: 0 --> 1

Char. 280: 0 --> 1

Char. 291: 1 --> 0

Char. 393: 0 --> 1

Char. 418: 1 --> 0

Char. 434: 1 --> 3

Node 297 :

Char. 101: 0 --> 1

Char. 427: 2 --> 0

Char. 787: 0 --> 1

Node 298 :

Char. 409: 1 --> 3

Char. 467: 1 --> 0

Char. 468: 0 --> 1

Char. 503: 1 --> 0

Char. 613: 2 --> 0

Char. 614: 1 --> 2

Char. 667: 1 --> 0

Char. 924: 0 --> 1

Node 299 :

Char. 193: 0 --> 1

Char. 204: 0 --> 1

Char. 299: 0 --> 1

Char. 300: 0 --> 1

Char. 424: 1 --> 0

Node 300 :

Char. 60: 0 --> 2

Char. 69: 1 --> 0

Char. 71: 1 --> 0

Char. 189: 0 --> 2

Char. 208: 0 --> 1

Char. 209: 2 --> 0

Char. 240: 0 --> 1

Char. 409: 2 --> 1

Char. 429: 0 --> 1

Char. 901: 0 --> 1

Node 301 :

Char. 127: 2 --> 3

Char. 191: 0 --> 1

Char. 209: 2 --> 0

Char. 241: 1 --> 0

Node 302 :

Char. 29: 0 --> 2

Char. 110: 2 --> 1

Char. 122: 0 --> 1

Char. 153: 1 --> 0

Char. 242: 1 --> 0

Char. 252: 0 --> 1

Char. 295: 0 --> 2

Node 303 :

Char. 13: 0 --> 1

Char. 140: 0 --> 1

Char. 149: 0 --> 1

Char. 155: 1 --> 0

Char. 338: 0 --> 1

Char. 417: 0 --> 1

Char. 569: 1 --> 0

Char. 649: 1 --> 0

Char. 657: 0 --> 1

Char. 665: 0 --> 1

Char. 667: 1 --> 2

Char. 669: 0 --> 1

Char. 683: 1 --> 0

Node 304 :

Char. 106: 0 --> 1

Char. 113: 1 --> 0

Char. 155: 1 --> 2

Char. 165: 0 --> 1

Char. 598: 1 --> 0

Char. 678: 0 --> 1

Char. 705: 1 --> 0

Char. 723: 0 --> 1

Char. 725: 0 --> 1

Char. 749: 0 --> 1

Char. 767: 0 --> 1

Char. 787: 0 --> 1

Char. 887: 0 --> 1

Char. 889: 0 --> 1

Char. 904: 0 --> 1

Node 305 :

Char. 326: 1 --> 0

Char. 634: 0 --> 1

Node 306 :

Char. 467: 1 --> 0

Char. 787: 1 --> 0

Char. 803: 2 --> 3

Node 307 :

Char. 42: 0 --> 1

Char. 63: 1 --> 0

Char. 109: 0 --> 1

Char. 670: 0 --> 1

Char. 758: 1 --> 0

Node 308 :

Char. 29: 0 --> 1

Char. 355: 0 --> 1

Char. 535: 0 --> 2

Char. 779: 0 --> 1

Char. 957: 1 --> 0

Node 309 :

Char. 58: 2 --> 0

Char. 103: 0 --> 1

Char. 137: 0 --> 1

Char. 342: 1 --> 0

Node 310 :

Char. 631: 0 --> 1

Char. 912: 0 --> 1

Char. 925: 0 --> 1

Node 311 :

Char. 642: 1 --> 0

Node 312 :

Char. 91: 1 --> 0

Char. 117: 1 --> 0

Char. 336: 1 --> 0

Char. 391: 1 --> 0

Char. 434: 1 --> 2

Char. 534: 0 --> 1

Char. 750: 0 --> 1

Node 313 :

Char. 752: 0 --> 1

Node 314 :

Char. 427: 0 --> 1

Char. 439: 0 --> 1

Char. 562: 0 --> 1

Char. 589: 1 --> 0

Char. 737: 0 --> 1

Char. 840: 1 --> 0

Node 315 :

Char. 44: 0 --> 1

Char. 98: 1 --> 0

Char. 532: 1 --> 2

Char. 617: 0 --> 1

Char. 705: 0 --> 1

Char. 708: 0 --> 1

Char. 748: 0 --> 1

Char. 919: 1 --> 0

Node 316 :

Char. 184: 0 --> 1

Char. 235: 0 --> 1

Char. 249: 0 --> 1

Char. 425: 01 --> 2

Char. 492: 0 --> 1

Char. 561: 0 --> 1

Char. 727: 0 --> 1

Char. 728: 0 --> 1

Char. 898: 1 --> 0

Node 317 :

Char. 71: 1 --> 0

Char. 429: 0 --> 1

Node 318 :

Char. 494: 1 --> 0

Char. 558: 0 --> 1

Node 319 :

Char. 60: 0 --> 2

Char. 82: 1 --> 2

Char. 113: 1 --> 0

Char. 335: 1 --> 0

Char. 550: 0 --> 1

Char. 819: 1 --> 0

Node 320 :

Char. 97: 0 --> 1

Char. 657: 1 --> 0

Char. 734: 2 --> 1

Char. 947: 1 --> 2

Node 321 :

Char. 10: 1 --> 0

Char. 15: 2 --> 1

Char. 64: 0 --> 1

Char. 88: 1 --> 0

Char. 428: 0 --> 1

Char. 778: 1 --> 0

Char. 942: 1 --> 0

Node 322 :

Char. 9: 0 --> 1

Char. 285: 1 --> 2

Char. 381: 1 --> 0

Char. 421: 1 --> 0

Node 323 :

Char. 203: 0 --> 1

Char. 207: 0 --> 1

Char. 357: 1 --> 0

Char. 418: 1 --> 0

Char. 425: 2 --> 1

Char. 442: 1 --> 0

Node 324 :

Char. 184: 1 --> 0

Char. 201: 1 --> 0

Char. 206: 1 --> 0

Char. 209: 0 --> 1

Char. 339: 1 --> 0

Char. 343: 1 --> 0

Char. 427: 2 --> 0

Char. 899: 1 --> 0

Node 325 :

Char. 631: 0 --> 1

Char. 649: 0 --> 1

Char. 652: 1 --> 0

Char. 839: 1 --> 2

Char. 867: 0 --> 1

Char. 870: 0 --> 1

Char. 879: 1 --> 0

Char. 938: 1 --> 0

Node 326 :

Char. 65: 0 --> 1

Char. 98: 1 --> 0

Char. 185: 0 --> 1

Char. 190: 0 --> 1

Char. 195: 0 --> 1

Char. 226: 1 --> 0

Char. 356: 1 --> 0

Char. 441: 0 --> 1

Char. 609: 0 --> 3

Char. 614: 1 --> 2

Char. 913: 1 --> 0

Node 327 :

Char. 154: 0 --> 1

Char. 156: 0 --> 1

Char. 331: 0 --> 1

Char. 487: 01 --> 2

Node 328 :

Char. 499: 0 --> 1

Char. 514: 1 --> 2

Char. 592: 2 --> 0

Char. 636: 1 --> 0

Char. 657: 0 --> 1

Char. 688: 0 --> 1

Char. 788: 1 --> 0

Char. 794: 1 --> 0

Char. 826: 1 --> 0

Node 329 :

Char. 953: 0 --> 1

Char. 954: 1 --> 2

Node 330 :

Char. 884: 1 --> 2

Node 331 :

Char. 854: 2 --> 1

Node 332 :

Char. 593: 0 --> 1

Char. 650: 1 --> 0

Char. 803: 3 --> 1

Node 333 :

Char. 44: 1 --> 0

Char. 77: 0 --> 1

Char. 229: 1 --> 0

Char. 285: 1 --> 0

Node 334 :

Char. 327: 2 --> 1

Char. 342: 1 --> 0

Char. 355: 1 --> 0

Node 335 :

Char. 388: 0 --> 1

Char. 753: 1 --> 2

Char. 793: 0 --> 1

Char. 811: 0 --> 1

Node 336 :

Char. 549: 2 --> 0

Char. 785: 0 --> 1

Char. 787: 0 --> 1

Node 337 :

Char. 28: 1 --> 0

Char. 69: 1 --> 0

Char. 123: 0 --> 1

Char. 127: 1 --> 2

Char. 140: 0 --> 1

Char. 165: 0 --> 1

Char. 637: 0 --> 1

Char. 693: 1 --> 0

Char. 756: 1 --> 0

Char. 809: 0 --> 1

Char. 905: 0 --> 1

Node 338 :

Char. 95: 1 --> 0

Node 339 :

Char. 19: 0 --> 1

Char. 58: 2 --> 0

Node 340 :

Char. 289: 0 --> 1

Char. 292: 0 --> 2

Char. 825: 2 --> 1

Char. 855: 2 --> 1

Node 341 :

Char. 149: 0 --> 2

Char. 235: 0 --> 1

Char. 236: 0 --> 1

Char. 278: 1 --> 0

Char. 423: 3 --> 2

Char. 479: 12 --> 3

Node 342 :

Char. 13: 1 --> 2

Char. 23: 1 --> 0

Char. 72: 1 --> 0

Char. 335: 0 --> 1

Char. 407: 0 --> 1

Char. 725: 0 --> 1

Node 343 :

Char. 425: 1 --> 2

Char. 506: 1 --> 2

Char. 564: 1 --> 0

Char. 592: 2 --> 0

Node 344 :

Char. 256: 0 --> 1

Char. 260: 1 --> 0

Char. 261: 0 --> 1

Char. 291: 0 --> 1

Char. 390: 0 --> 1

Char. 425: 0 --> 1

Char. 465: 1 --> 0

Char. 534: 0 --> 1

Char. 535: 0 --> 1

Char. 551: 1 --> 0

Char. 819: 0 --> 1

Node 345 :

Char. 782: 0 --> 1

Char. 795: 0 --> 1

Char. 796: 1 --> 0

Char. 820: 0 --> 1

Char. 882: 0 --> 1

Node 346 :

Char. 15: 1 --> 0

Char. 409: 1 --> 2

Node 347 :

Char. 570: 1 --> 0

Char. 782: 0 --> 1

Node 348 :

Char. 438: 1 --> 2

Node 349 :

Char. 154: 0 --> 1

Char. 376: 1 --> 0

Char. 595: 0 --> 1

Char. 808: 1 --> 0

Char. 809: 0 --> 1

Node 350 :

Char. 583: 0 --> 1

Node 351 :

Char. 505: 1 --> 2

Char. 943: 0 --> 1

Node 352 :

Char. 72: 1 --> 0

Char. 85: 0 --> 1

Char. 498: 1 --> 0

Char. 593: 0 --> 1

Char. 914: 1 --> 2

Node 353 :

Char. 495: 1 --> 0

Char. 659: 0 --> 1

Char. 689: 0 --> 1

Node 354 :

Char. 57: 0 --> 1

Char. 127: 1 --> 0

Char. 345: 0 --> 1

Char. 389: 1 --> 2

Char. 392: 0 --> 1

Char. 427: 2 --> 1

Char. 804: 0 --> 1

Char. 936: 0 --> 1

Char. 949: 1 --> 2

Node 355 :

Char. 868: 1 --> 0

Node 356 :

Char. 530: 1 --> 0

Char. 547: 0 --> 2

Char. 594: 0 --> 1

Char. 652: 1 --> 0

Char. 662: 1 --> 0

Char. 690: 0 --> 1

Char. 753: 12 --> 3

Char. 782: 0 --> 1

Char. 809: 1 --> 0

Char. 853: 1 --> 2

Char. 871: 0 --> 1

Char. 880: 0 --> 1

Char. 894: 1 --> 0

Char. 905: 0 --> 1

Char. 915: 0 --> 1

Char. 916: 0 --> 1

Char. 936: 0 --> 2

Node 357 :

Char. 912: 0 --> 1

Char. 919: 1 --> 0

Node 358 :

Char. 947: 2 --> 1

Char. 953: 1 --> 2

Node 359 :

Char. 39: 1 --> 0

Char. 70: 1 --> 0

Char. 190: 0 --> 1

Node 360 :

Char. 884: 1 --> 2

Node 361 :

Char. 192: 0 --> 1

Char. 273: 0 --> 1

Char. 750: 0 --> 1

Node 362 :

Char. 19: 1 --> 0

Char. 21: 1 --> 0

Char. 198: 1 --> 0

Char. 200: 0 --> 1

Char. 227: 0 --> 1

Char. 244: 1 --> 0

Char. 268: 4 --> 3

Char. 277: 0 --> 1

Char. 327: 0 --> 1

Char. 438: 1 --> 2

Char. 441: 0 --> 1

Char. 784: 0 --> 1

Char. 870: 0 --> 1

Char. 873: 0 --> 1

Char. 933: 0 --> 1

Node 363 :

Char. 242: 0 --> 1

Char. 243: 0 --> 1

Char. 248: 2 --> 1

Node 364 :

Char. 4: 0 --> 1

Char. 16: 2 --> 3

Char. 40: 0 --> 1

Char. 70: 0 --> 1

Char. 189: 0 --> 1

Char. 361: 1 --> 0

Char. 840: 1 --> 0

**Figure S1**. *Mirischia asymmetrica* (SMNK 2349). Vertebral series in (a) left lateral and (b) right lateral views. Abbreviations: ccdl, caudal centrodiapophyseal lamina; cdf centrodiapophyseal fossa; cpl, centroparapophyseal lamina; cpzl, centropostzygapophyseal lamina; d, diapophysis; ivf, intervertebral foramen; ncs, neurocentral suture; ns, neural spine; nslc, neural spine longitudinal concavity; poz, postzygapophysis; psl, postspinal lamina; przcdf, prezygapophyseal centrodiapophyseal fossa; przdl, prezygodiapophyseal lamina; prz, prezygapophysis; pzcdf, postzygapophyseal centrodiapophyseal fossa; pzcpf, postzygapophyseal centroparapophyseal fossa; pzdl postzygodiapophyseal lamina; r, rib; s, sacral vertebra; spzl, spinopostzygapophyseal lamina; sr, sacral rib; t, trunk vertebra. Scale bar equals 5 cm.

**Figure S2**. *Mirischia asymmetrica* (SMNK 2349). Details of the pelvic girdle in (a) right and (b) left views, showing the probable breakage in (c–d) caudal views. Arrows indicate the potential area where the pubic flange was broken. Not to scale.
